# Proceedings from the 12^th^ Annual Conference on the Science of Dissemination and Implementation: Arlington, VA, USA. 4-6 December 2019

**DOI:** 10.1186/s13012-020-00985-1

**Published:** 2020-05-08

## Conference website

### I1 Introduction

#### Gila Neta^1^, David A. Chambers^1^, Lisa Simpson^2^

##### ^1^National Cancer Institute, Rockville, MD, USA; ^2^AcademyHealth, Washington, DC, USA

###### **Correspondence:** Gila Neta (Gila.Neta@nih.gov)

“Raising the bar on the rigor, relevance, and rapidity of dissemination and implementation science” was the theme of the 12^th^ Annual Conference on the Science of Dissemination and Implementation (D&I) in Health in December 2019, co-hosted by the National Institutes of Health (NIH) and AcademyHealth. We continued to see record-breaking attendance with over 1,400 researchers, practitioners, and policymakers convening in Arlington, VA to reflect on advancing the science. With each year of our partnership between NIH and AcademyHealth, we have seen tremendous growth in the field demonstrated by ever increasing numbers of attendees as well as a substantial percentage of newcomers to the conference series. As in prior years, attendees were from a diversity of employment settings: 43 percent in universities, 17 percent government, 14 percent in hospitals, provider organizations, or health plans, 10 percent in non-university research or policy organizations, and 18 percent from a mix of other settings. This reflects that D&I research draws upon a variety of contexts.

In this twelfth iteration of the conference, the two-and-a-half-day agenda included a keynote speaker, three plenary panels, concurrent paper sessions, and hundreds of posters, all touching upon the conference theme of improving the rigor, relevance, and rapidity of our science. This supplement contains the abstracts from the concurrent paper sessions, reflecting the variety of dissemination and implementation science supported and disseminated by our conference sponsors, which include the National Institutes of Health (NIH), AcademyHealth, the Agency for Healthcare Research and Quality (AHRQ), the Patient Centered Outcomes Research Institute (PCORI), the Robert Wood Johnson Foundation (RWJF), and the US Department of Veterans Affairs (VA). Not included here are 432 papers which were presented in poster format and can be viewed at https://academyhealth.confex.com/academyhealth/2019di/meetingapp.cgi/ModulePosterSessions/0.

In this sixth year of our partnership between the NIH and AcademyHealth in co-hosting the conference, we continued to be assisted by a multidisciplinary program planning committee which informed the plenary session development, recruited key speakers, and helped to develop the topics for workshops and discussion forums. We organized concurrent sessions across nine tracks, with two to three leads per track developing the call for abstracts and chairing review panels to select thematic sessions. We also convened a scientific advisory panel to advise on the overall conference, particularly highlighting the theme of improving the rigor, relevance, and rapidity of dissemination and implementation science domestically and abroad. We were able to bolster attendance from low and middle income countries by planning in advance, advertising, and offering a number of travel scholarships for the first time this year, thanks to AcademyHealth, the National Cancer Institute’s Center for Global Health and the Fogarty International Center, enabling 14 attendees from low and middle-income countries, bringing our total of non US based registrants to 65.

Our conference program began with a phenomenal keynote address by Dr. Amy Edmondson, Novartis Professor of Leadership and Management at the Harvard Business School, on “The Fearless Health Care Organization: Creating Psychological Safety for Learning and Implementation.” Dr. Edmondson described her discovery of a critical construct, psychological safety, in organizational behavior and its influence on effective learning and implementation within healthcare organizations. Moreover, her talk highlighted the importance of rigorous and relevant determinant and outcome measures to best understand drivers of effective implementation. These themes were further reflected across the three plenary panel sessions, which focused on fostering natural laboratories in D&I research, as well as two addressing methods for rapid, relevant, and rigorous study designs and measures. Each plenary panel enabled active dialogue between presenters and with the audience, further deepening the discussion and surfacing many of the nuances and issues in D&I science.

Concurrent sessions were organized into nine thematic tracks, including Behavioral Health, Clinical Care Settings (separated into two tracks: Patient-Level Interventions and System-Level Interventions), Global Dissemination and Implementation Science, Promoting Health Equity and Eliminating Disparities, Health Policy Dissemination and Implementation Science, Prevention and Public Health, and Models, Measures and Methods, and Building the Future of D & I Science: Training, Infrastructure, and Emerging Research Areas. This supplement is once again organized by track themes.

The call for abstracts generated 832 submissions, including individual paper presentations, individual posters, and panel presentations spread across the nine tracks. Over one hundred reviewers from multiple disciplines, sectors, settings and career stages devoted their time to ensuring a comprehensive review, and reviews were conducted within each track and coordinated by the track leads.

For the final program, 134 papers across 31 oral abstract sessions, 52 papers across 15 panels, and 432 posters were presented over the three-day meeting. Slides for the oral presentations and panels (with the agreement of the authors) were posted on the conference website and all abstracts were included on the conference webapp (https://academyhealth.confex.com/academyhealth/2019di/meetingapp.cgi/Home/0). For the fourth year in a row, we hosted a poster slam to enable the nine top scoring posters from each of the nine tracks to be presented in rapid succession, sharing key findings in five minutes each. And for the second time we hosted a Best of D&I Session which brought together four papers presented during the conference across four different tracks to highlight major lessons learned and their value to the field. Additionally, we offered poster walks with leading experts in the field and selected a Best Poster award for which the top ranked poster in each track competed. New this year, we promoted a novel poster format (https://www.youtube.com/watch?v=1RwJbhkCA58&feature=youtu.be) to be more engaging for both the presenter and audience. Several presenters used the format and informal feedback from them and conference attendees was that the format spurred greater interaction and discussion during the poster session. Finally, we continued to offer a limited number of scholarships for patients to attend the conference in addition to the aforementioned scholarships for participants from low- and middle- income countries, contributing greatly to the quality and relevance of conference discussions.

This supplement has compiled the abstracts for presented papers and panel sessions from the 12th Annual Conference on the Science of Dissemination and Implementation in Health: Raising the bar on the rigor, relevance, and rapidity of dissemination and implementation science. We are pleased to have the combined proceedings from the conference together in one volume once again. And new this year, we anticipate three debate style papers in the coming months highlighting the issues raised in each of our plenary panels to be added to this collection. We look forward to the 13th Annual meeting, scheduled for December 14-16, 2020 in Washington, DC.

## Behavioral Health

### S1 Main findings of the substance abuse treatment to HIV care (SAT2HIV) project: A type 2 effectiveness-implementation hybrid trial

#### Bryan Garner^1^, Stephen Tueller^1^, Steve Martino^2^, Heather Gotham^3^, Kathryn Speck^4^, Mike Chaple^5^, Denna Vandersloot^6^, Michael Bradshaw^1^, Liz Ball^1^, Alyssa Toro^1^, Marianne Kluckman^1^, Mat Roosa^7^, James Ford^8^

##### ^1^RTI International, Research Triangle Park, NC, USA; ^2^Connecitcut Healthcare System, Veterans Health Administration, West Haven, CT, USA; ^3^Stanford University, Palo Alto, CA, USA; ^4^University of Nebraska, Lincoln, Lincoln, NE, USA; ^5^Columbia University, New York, NY, USA; ^6^University of Washington, Portland, OR, USA; ^7^Onondaga County, Syracuse, NY, USA; ^8^University of Wisconsin - Madison, Madison, WI, USA

###### **Correspondence:** Bryan Garner (bgarner@rti.org)

**Background:** Improving the integration of substance use services within HIV service settings is an important public health concern. To help understand how best to improve the integration of substance use services within HIV service settings, the National Institute on Drug Abuse funded a type 2 effectiveness-implementation hybrid trial entitled the Substance Abuse Treatment to HIV Care (SAT2HIV) Project. This presentation focuses on the SAT2HIV Project’s main findings.

**Methods:** Using a cluster-randomized design, 39 HIV service organizations and their staff were randomized to either implementation-as-usual (IAU) or IAU plus Implementation & Sustainment Facilitation (IAU+ISF). As part of the IAU condition, staff received training, feedback, and coaching in a motivational interviewing-based brief intervention (BI) for substance use. As part of the IAU+ISF, staff received the IAU strategy, as well as participated in external facilitation meetings with an ISF coach. Within each HIV service organization, eligible and consenting clients were randomized to usual care (UC) or UC plus BI (UC+BI). The analytic sample included 678 clients (82% follow-up rate), nested within 78 BI staff, nested within the 39 HIV service organizations. The preparation-phase outcome was staff time-to-proficiency (i.e., a staff-level measure of the number of days between completing the initial training and demonstrating BI proficiency). Implementation-phase outcomes were: staff implementation effectiveness (i.e., a staff-level measure of the consistency and quality of BI implementation) and client substance use at follow-up (i.e., a client-level measure of past-28 day primary substance use).

**Findings:** The ISF strategy reduced time-to-proficiency (β = - .66), but this reduction was not significantly less (*p* < .05) than what was achieved by staff in the IAU condition. However, the ISF did significantly improved implementation effectiveness (β = .73, *p* < .001) beyond what was achieved in the IAU condition. Moreover, the ISF strategy did significantly improve the BI’s effectiveness for reducing client substance use (β = -2.25, *p* < .05).

**Implications for D&I Research:** Training, feedback, and coaching was sufficient for helping staff demonstrate proficiency in a motivational interviewing-based BI for substance use. However, the ISF strategy was found to help significantly improve implementation effectiveness and help significantly reduce client substance use.

**Primary Funding Source**

National Institutes of Health

### S2 A theory-informed, rapid cycle, human-centered model for improving collaborative care sustainability

#### Nathalie Moise^1^, Luis Blanco^1^, Rachel Monane^1^, Yenilshia Firpo-Greenwood^2^, Sara Greenberg^2^, Diane Wilson^2^, Nancy Chang^1^, Sun Yi^3^, DepCare Advisory Board^2^

##### ^1^Columbia University Medical Center, New York, NY, USA; ^2^New York Presbyterian Hospital, New York, NY, USA; ^3^NightOwl Interactive LLC, New York, NY, USA

###### **Correspondence:** Nathalie Moise (nm2562@cumc.columbia.edu)

**Background:** Expert consensus exists around implementation strategies, but few studies explore sustainability strategies or incorporate rigorous, adaptive, rapid cycle models to address barriers over time. Recent research suggests collaborative care (CC) sustainability hinges on patient and provider engagement. We describe a theory-informed, rapid cycle implementation team approach for improving patient and provider engagement in primary care settings seeking to sustain CC programs.

**Methods:** We created a 5-step, theory-informed, implementation team-based model: (1) We applied the Behavior Change Wheel framework, a multi-step process for mapping barriers to theory-informed, feasible/practical behavior change techniques and modes-of-delivery to interviews conducted with national CC experts (n=10), stakeholder interviews with psychiatrists, providers, administrators, and CC managers from 6 healthcare systems sustaining CC (n=24), and patient focus groups (n=12) examining barriers to sustainability and patient engagement; (2) a multidisciplinary team of providers and human-centered design experts translated techniques into a candidate interface for improving patient engagement; (3) a CC provider/patient team revised the interface and problem-solved implementation processes; (4) 8-12 naïve patients and providers user-tested the tool and (5) an advisory board of statistical and behavioral experts synthesized findings into actionable improvements to design and implementation. We designed, user-tested and iteratively adapted (e.g., to competing IT initiatives) until we maximized usability, effectiveness, acceptability, and feasibility.

**Findings:** We identified barriers related to Capability (CC knowledge, literacy/language), Opportunity (stigma/culture, referral processes subject to error, unyielding clinician/mental health provider workflow limiting patient rapport/diagnosis, limited resources), and motivation (treatment beliefs, self-efficacy). A theory-informed multi-component intervention addressing barriers includes (1) a 10-minute, waiting room administered, tablet-delivered, video-assisted, automated shared decision-making tool with depression screening, personalized psychoeducation/treatment options, and behavioral activation (2) direct self-referral to care managers (3) real-time treatment preference reports for providers and care manager (4) automated, ongoing care manager/provider psychoeducation and (4) personalized nontraditional options (e.g., telepsychiatry). User-testing demonstrated marked improvement in usability and effectiveness (e.g., reduced stigma). The intervention is currently being tested in a stepped wedge design across our healthcare system.

**Implications for D&I Research:** We demonstrate how employing theory-informed user-centered design principles within implementation team strategies can engage healthcare systems at multiple levels in building adaptive sustainability strategies.

**Primary Funding Source**

Agency for Healthcare Research and Quality

### S3 Organizational capacity and readiness for providing co-AUD treatment in public mental health settings

#### Katherine Watkins^1^, Allison Ober^1^, Brian Hurley^2^, Sarah Hunter^1^, Catherine Cohen^1^, Sarika Bharil^1^, Michael McCreary^3^

##### ^1^RAND Corporation, Santa Monica, CA, USA; ^2^Division of General Internal Medicine and Health Services Research, Los Angeles, CA, USA; ^3^UCLA, Los Angeles, CA, USA

###### **Correspondence:** Katherine Watkins (kwatkins@rand.org)

**Background:** Despite the availability of effective pharmacotherapies for alcohol use disorders (AUD), most individuals with co-occurring serious mental illness and AUD (Co-AUD) never receive treatment—treatment that could significantly improve outcomes. Public mental health organizations are an opportune setting in which to increase access to medications for AUD, because individuals with Co-AUD are more likely to access mental health care than substance use treatment. This study sought to assess organizational capacity and readiness for providing Co-AUD treatment in public mental health settings, prior to developing an implementation intervention.

**Methods:** We conducted a concurrent mixed methods evaluation of organizational capacity and readiness for integrating Co-AUD treatment into 8 publicly funded outpatient mental health clinics in Los Angeles County. Clinics were purposively selected to ensure diversity in clinic size, ethnicities served and geography. We conducted surveys of all staff (N=334), interviewed administrators from each site (N=39) and conducted 16 provider focus groups. We used two theoretical frameworks to develop our data collection tools, and quantitative and qualitative methods to analyze the data.

**Findings:** Despite general agreement that delivering Co-AUD care matched organizational priorities, staff across the 8 clinics identified gaps in organizational capacity and readiness to provide Co-AUD treatment. Gaps included policy barriers related to reimbursement, lack of knowledge about whether upper management supported the effort, a lack of fit with existing workflows and the absence of standardized workflow guidelines, difficulty with intra-organizational communication, and lack of patient readiness. Readiness for integrating care varied; while prescribing providers generally were willing to prescribe AUD pharmacotherapy, therapists and case managers had concerns about the time needed to provide psychosocial treatment and recovery support. Behavioral readiness was low.

**Implications for D&I Research:** Although most staff endorsed the importance of integrating and delivering Co-AUD treatment, there were gaps in organizational capacity to deliver care and variations in psychological and behavioral readiness by provider type. Implementation interventions that address system- provider- and patient-level barriers are needed. Next steps are to address these findings using a web-based implementation toolkit to help public mental health clinic leaders and providers integrate and deliver Co-AUD care.

**Primary Funding Source**

National Institutes of Health

### S4 Application of the ‘what matters most’ framework to examine socio-cultural aspects of the stigmatization process for women with obstetric fistula in Ghana: An ethnographic approach to inform selection of an implementation strategy

#### Nessa Ryan^1^, Gabriel Ganyaglo^2^, Alison El Ayadi^3^, Lawrence Yang^1^, Bernadette Boden-Albala^4^

##### ^1^NYU College of Global Public Health, New York, NY, USA; ^2^Korle Bu Teaching Hospital, Accra, Ghana; ^3^UCSF School of Medicine, San Francisco, CA, USA; ^4^New York University, New York, NY, USA

###### **Correspondence:** Nessa Ryan (ryann01@nyu.edu)

**Background:** Health-related stigma challenges an individual’s ability to seek, engage in, and adhere to prevention efforts and treatment for numerous global conditions, including HIV, substance use, and obstetric fistula (OF), a stigmatizing maternal morbidity causing urinary incontinence. For women with OF, the socio-cultural determinants of stigma that challenge meaningful aspects of daily life, or ‘what matters most’ (WMM), have not been examined and thus remain a challenge to implementing a stigma-reduction intervention. Therefore, the objective: to examine the stigma process and identify how stigma challenges WMM for women with OF in Ghana, thus informing selection of implementation strategies.

**Methods:** This convergent mixed methods study collected 38 semi-structured interviews among women with OF living in two communities in Ghana. Using an ethnographic approach, the interviews were comprised of closed-ended questions on socio-demographics, clinical characteristics, and stigma severity, while open-ended questions examined stigma experience and social support. Qualitative findings were developed using thematic analysis; quantitative results were generated using descriptive statistics; meta-inferences evolved from joint analyses.

**Findings:** OF-related stigma threatens women’s ability to fulfill social expectations and roles as woman, partner, and mother. Women able to *keep themselves very well*, or maintain a neat appearance and home, experienced less stigma. This was related to expectations of labor that allowed for *giving to others* and *getting/keeping a partner*, making childbearing more likely. In the agrarian, patriarchal North, stigma was more severe as fistula additionally challenged the ability to farm and respect male family. Women with a living child and supportive partner were less stigmatized. This work builds on previous applications of WMM framework regarding HIV and mental health stigma to develop culturally informed measures and stigma-reduction interventions. Ultimately, findings informed selection of culturally informed implementation strategies: for communities in northern Ghana, male heads of household will be key stakeholders; while in the south, female traditional leaders will champion the planned intervention.

**Implications for D&I Research:** This mixed methods ethnographic approach provides a transferable model for rapidly identifying socio-cultural factors to target within a theory-driven stigma-reduction intervention and implementation strategy. This team-based approach could be adapted to improve behavioral health outcomes among diverse, vulnerable, and underserved populations around the globe.

**Primary Funding Source**

National Institutes of Health

### S5 Hybrid type-3 RCT of virtual learning collaboratives vs. technical assistance in implementing health promotion cardiovascular risk reduction for individuals with mental illness and obesity in mental health organizations

#### Stephen Bartels^1^, Kelly Aschbrenner^2^, Souvik Banerjee^3^

##### ^1^Massachusetts General Hospital, Boston, MA, USA; ^2^Geisel School of Medicine at Dartmouth, Lebanon, NH, USA; ^3^MGH-Harvard, Boston, MA, USA

###### **Correspondence:** Stephen Bartels (SJBARTELS@mgh.harvard.edu)

**Background:** People with serious mental illness, about 3-4% of the US population, have among the greatest health disparities in the nation with a life expectancy up to 25 years less than the general population associated with obesity and sedentary lifestyles. InSHAPE, is an evidence-based practice associated with clinically significant cardiovascular risk reduction in approximately half of participants. This study addresses a major gap in the research literature by testing the effectiveness of Virtual Learning Collaboratives (VLC) compared to individual site Technical Assistance (TA) as a health promotion implementation strategy.

**Methods:** Hybrid-Type-3 effectiveness-implementation cluster randomized trial of 49 behavioral health provider organizations: Following an in-person group training, organizations received 18-months of web-delivered VLC monthly learning collaborative sessions (n=23) or telephone-delivered individual technical assistance (n=26) of four scheduled conference calls over 18 months and as-needed follow-up. Implementation outcomes were assessed at 3, 6, 12, and 24 months and participant-level outcomes were measured every 3 months after enrollment.

**Findings:** For program participants, (overweight and/or obese individuals with serious mental illness), VLC vs. TA was associated with a similar *proportion* of individuals achieving clinically significant cardiovascular risk-reduction at 12 months (5% weight loss and/or increased fitness) 62.2% vs. 61.7%. However, at the organization-level, VLC was associated with over 50% greater *number* of individuals receiving InSHAPE; greater number of health mentor sessions in their first 6 months in the program (VLC=16.2 vs TA=13.7), and 1.76 times greater number of participants receiving 15 or more health mentor sessions (P<0.0001). VLC was also associated with a greater InSHAPE fidelity score at 12 months (90.5 vs. 79.1, p=.002) (maximum score==110) and over double the proportion with good fidelity VLC (73.9%) vs. TA (34.8%) p=.009) (score (88-110).

**Implications for D&I Research:** Virtual learning collaboratives, compared to individual site technical assistance, achieved similar clinically-significant cardiovascular risk reduction at the participant-level, and superior implementation outcomes at the organization-level with respect to fidelity of the intervention and greater program reach. Virtual learning collaboratives appear to be an effective and scalable implementation strategy when implementing a new evidence-based practice, including for practices beyond the organization’s usual mission, scope of practice, type of services delivered, and financing.

**Primary Funding Source**

National Institutes of Health

### S6 Depression screening among coronary heart disease patients: Measuring fidelity when guidelines are simultaneously over- and under-implemented.

#### Nathalie Moise, Othanya Garcia, Todd Jones, Jennifer Mizhquiri Barbecho, Andrea Duran, Jessica Singer, Siqin Ye

##### Columbia University Medical Center, New York, NY, USA

###### **Correspondence:** Nathalie Moise (nm2562@cumc.columbia.edu)

**Background:** Depressive symptoms in coronary heart disease (CHD) predict recurrent cardiovascular events and mortality. Expert groups recommend systematic depression screening (i.e., Patient Health Questionnaires (PHQ)), but few studies have assessed fidelity (i.e., the extent to which intervention delivery adheres to originally developed protocols (e.g., dose/frequency)). Few also seek to simultaneously assess under *and* over-screening, of particular concern in multi-morbid patients.

**Methods:** We conducted a mixed methods study in a large academic, integrated behavioral primary care setting serving ≥ 12,000 unique patients/year. We employed a fidelity measurement framework (Carroll, 2008) to assess content (use of PHQ2/PHQ9), coverage (% of CHD patients with ≥1 screen/year) and frequency (median screens/individual/year) using electronic health record (EHR) data analysis. To measure quality and complexity, we conducted structured observations/time motion analyses and screened a random sample of CHD patients by phone. We employed descriptive statistics for quantitative responses.

**Findings:** Of 1170 CHD patients, 896 (77.9%) received ≥1 PHQ2 and/or PHQ9 (median 2.0 [IQR=3]/patient/year) of whom 839 (93.6%) w/PHQ=0 and 52 (5.8%) w/PHQ9≥10 (moderately depressed). Of 115 participants screened by phone, only 15 (8.6%) had PHQ=0 but 29 (25%) PHQ9≥10 of whom 37.9% were screened/recognized or in treatment. Observations revealed PHQ2s routinely administered with vitals, but suboptimal screening quality (e.g., prompting “you’re not depressed right?” in lieu of validated questions), PHQ2 administration in specialty settings (e.g., neurology) and suboptimal PHQ9 administration. Key observed facilitators included electronic algorithms for excluding those already screened, tablet-administered PHQ2s/PHQ9s, and patient/provider education/activation to promote treatment.

**Implications for D&I Research:** Both over-screening and poor screening quality may contribute to suboptimal identification of high-risk depressed CHD patients, with implications for broader primary care populations. While contingent on EHR data entry/quality, this study provides key facilitators for improving implementation efforts but also prompts consideration for de-implementation given over-screening, marked false-negative rates and low treatment uptake even in integrated primary care settings.

**Primary Funding Source**

National Institutes of Health

### S7 Patient perceptions of data visualizations and utilizing multiple mobile health technologies to support diabetes self-management

#### Allison Lewinski^1^, Jacqueline Vaughn^2^, Anna Diane^2^, Ryan Shaw^2^

##### ^1^Center of Innovation to Accelerate Discovery and Practice Transformation, Durham Veterans Affairs Health Care System, Veterans Health Administration, Durham, NC, USA; ^2^Duke University, Durham, NC, USA

###### **Correspondence:** Allison Lewinski (allison.lewinski@duke.edu)

**Background:** Mobile health (mHealth) devices can facilitate the collection of real-time, in situ patient-generated health data (e.g., physical activity, blood glucose, weight, and medication adherence). These tools: (1) help patients monitor their biophysical and behavioral health and thus can improve the ability to engage in self-management; and (2) enable researchers and clinicians to deliver real-time targeted self-management interventions. Thus, understanding patient perceptions of using mHealth in support of diabetes self-management is necessary to increase the likelihood of implementation.

**Methods:** We conducted a mixed methods exploratory study of 60 patients with type 2 diabetes (T2DM). Patients used 4 mobile health devices (i.e., wireless glucometer, cellular scale, wearable accelerometer, and smartphone delivered text message surveys) for 6 months to monitor and track their blood glucose, weight, physical activity, and medication adherence. We completed semi-structured interviews with 20 participants to obtain patient perceptions of: (1) using mHealth technologies to collect and monitor their diabetes related health data; and (2) data visualizations (e.g., charts/graphs of the patient’s data) generated from these technologies during the study. As part of the study, the research team developed individual data visualizations for these 20 patients.

**Findings:** We identified two themes related to the patients’ perceptions of utilizing mHealth devices in diabetes self- management: (1) *feasibility*, described how easy or difficult the patients found each device to use; and (2) *utility*, described how they used each of the three devices over the six-month study period. We identified two themes related to the patients’ perceptions of the data visualizations: (1) *meaningfulness of the data visualization*, described how patients made sense of their healthcare data in relation to their self-management behaviors; and (2) *construction of the data visualization*, described patient perceptions of the design and structure of the data visualization.

**Implications for D&I Research:** Developing optimal health interventions using mHealth technologies requires understanding patient perceptions on how, why, and when patients use these technologies and their data. Our project lays an important foundation for understanding how patients use mHealth data for T2DM self-management and how to best present information to patients about their patient-generated healthcare data.

**Primary Funding Source**

National Institutes of Health

### S8 Cost-benefit analysis of team-based collaborative care implementation in outpatient general mental health

#### Christopher Miller^1^, Kevin Griffith^2^, Kelly Stolzmann^1^, Mark Bauer^3^

##### ^1^VA Boston Healthcare System, Veterans Health Administration, Boston, MA, USA; ^2^Boston University School of Public Health, Boston, MA, USA; ^3^VA Boston Healthcare System, Center for Healthcare Organization and Implementation Research (CHOIR), Veterans Health Administration, Boston, MA, USA

###### **Correspondence:** Christopher Miller (christopher.miller8@va.gov)

**Background:** Collaborative Chronic Care Models (CCMs) represent an evidence-based way to structure care for chronic mental health conditions, but there is limited evidence regarding their feasibility, effectiveness, or cost in routine practice settings. We previously reported results from a randomized hybrid II stepped wedge trial that used blended facilitation to implement CCM-based care in nine outpatient mental health teams in US Department of Veterans Affairs (VA) medical centers. We concluded that CCM implementation resulted in mixed clinical effects, but may have important implications for future health service utilization. In this presentation, we describe an economic analysis of our CCM implementation.

**Methods:** We undertook a cost-benefit analysis from the health system perspective, accounting for CCM implementation costs and direct medical costs attributable to mental health disorders. In sensitivity analyses, we checked for spillover effects of CCM onto costs for other medical conditions. We estimated implementation costs through salary data for internal and external facilitators. We calculated costs for inpatient stays, outpatient visits, and pharmacy using the VA Health Economics Resource Center’s (HERC) Average Cost Datasets, which estimate reimbursements Medicare would provide for similar services. Our treatment group comprised patients treated by the nine teams that participated in our stepped wedge trial during the year of CCM implementation. For comparison, we investigated treatment costs for patients treated by other outpatient mental health teams at the same facilities. We conducted one-way sensitivity analyses to determine robustness of results to variations +/-20% in individual model parameters, along with probabilistic sensitivity analysis using Monte Carlo simulation.

**Findings:** Initial analyses suggest that CCM implementation for a typical outpatient mental health team treating 1,000 patients was associated with about 70 fewer acute mental health hospitalization days, and about 600 fewer total hospitalization days, than for similar teams at the same medical centers during the facilitation year. The CCM intervention was found to be cost-saving in the base case and across several sensitivity analyses.

**Implications for D&I Research:** To our knowledge this is one of the first studies to apply cost-benefit analysis to real-world implementation of CCM-based outpatient mental health care. Our findings strengthen the business case for CCM implementation in these settings.

**Primary Funding Source**

Department of Veterans Affairs

### S9 The sustained patient-centered alcohol-related care (SPARC) trial—an implementation trial across 22 primary care sites—findings regarding alcohol screening and brief intervention (Aim 1)

#### Carol Achtmeyer^1^, Amy Lee^2^, Jennifer Bobb^2^, Julie Richards^2^, Evette Ludman^2^, Gwen Lapham^2^, Malia Oliver^2^, Ryan Caldeiro^3^, Rebecca Parrish^3^, Emily Williams^4^, Joseph Glass^2^, Paula Lozano^3^, Katharine Bradley^5^

##### ^1^VA Puget Sound, Veterans Health Administration, Seattle, WA, USA; ^2^Kaiser Permanente Washington Health Research Institute, Seattle, WA, USA; ^3^Kaiser Permanente Washington, Seattle, WA, USA; ^4^HSR&D, Veterans Health Administration, Seattle, WA, USA; ^5^Kaiser Permanente, Seattle, WA, USA

###### **Correspondence:** Carol Achtmeyer (carol.achtmeyer@va.gov)

**Background:** SPARC was a stepped-wedge, cluster-randomized implementation trial in 22 primary care (PC) sites testing state-of-the-art strategies to implement evidence-based care for the spectrum of unhealthy alcohol use. This report presents results of SPARC trial Aim 1: to increase the proportion of PC patients who have brief intervention (BI) for unhealthy alcohol use documented consistent with US Preventive Services Task Force recommendations.

**Methods:** The trial was conducted in Kaiser Permanente Washington (KPWA), a medium-sized integrated health system. The trial sample included all patients seen for PC across 22 sites, January 2015-July 2018. KPWA leaders partnered on the trial and asked to implement Behavioral Health Integration for depression and other drug use as part of the trial. Implementation strategies addressing Aim 1 included: 1) practice coaching to address stigma, knowledge gaps and support quality improvement, 2) electronic health record (EHR) decision support for screening and a BI handout, and 3) performance monitoring and feedback on screening and DSM-5 assessment—but not BI. The main Aim 1 outcome was documented BI within 14 days of a positive AUDIT-C alcohol screen. BI was measured from EHRs using natural language processing and procedure codes. Analyses used general linear mixed models accounting for correlation of measures within patients and sites, adjusted for covariates including calendar time, and followed CONSORT standards.

**Findings:** 255,789 patients made 953,402 PC visits pre-implementation; 228,258 patients made 615,515 visits post-implementation. Comparing pre- and post-implementation, alcohol screening increased from 2,018 to 8,319 per 10,000 PC patients with visits/month; positive screens increased from 502 to 1802 per 10,000 PC patients (p< 0.0001). Documented BI after a positive alcohol screen increased five-fold post-implementation (11 versus 57 per 10,000 PC patients with visits/month, p< 0.0001). However, only 5% of 52,263 patients who screened positive for unhealthy alcohol use had documented BI post-implementation.

**Implications for D&I Research:** While alcohol screening was successfully implemented (83% of patients), and BI increased 5-fold, 95% of patients with a positive screen for unhealthy alcohol use had no documented BI post-implementation. Although BI may have been provided without documentation, increased documentation of BI will likely require monitoring and feedback on BI performance and EHR prompts for BI documentation.

**Primary Funding Source**

Agency for Healthcare Research and Quality

### S10 The sustained patient-centered alcohol-related care (SPARC) trial—an implementation trial across 22 primary care sites—findings regarding diagnosis and treatment of alcohol use disorders (AUD)

#### Katharine Bradley^1^, Amy Lee^2^, Jennifer Bobb^2^, Julie Richards^2^, Evette Ludman^2^, Carol Achtmeyer^3^, Gwen Lapham^2^, Malia Oliver^2^, Ryan Caldeiro^4^, Rebecca Parrish^4^, Emily Williams^5^, Joseph Glass^2^, Paula Lozano^4^

##### ^1^Kaiser Permanente, Seattle, WA, USA; ^2^Kaiser Permanente Washington Health Research Institute, Seattle, WA, USA; ^3^VA Puget Sound, Veterans Health Administration, Seattle, WA, USA; ^4^Kaiser Permanente Washington, Seattle, WA, USA; ^5^HSR&D, Veterans Health Administration, Seattle, WA, USA

###### **Correspondence:** Katharine Bradley (katharine.a.bradley@kp.org)

**Background:** SPARC was a stepped-wedge, cluster-randomized implementation trial in 22 primary care (PC) clinics testing state-of-the-art strategies to implement evidence-based care for the spectrum of unhealthy alcohol use. This report presents results of main SPARC trial Aim 2: to increase the proportion of PC patients who have AUD recognized and treated.

**Methods:** The trial was conducted in Kaiser Permanente Washington (KPWA), a medium-sized integrated health system. KPWA leaders partnered on the trial and asked to implement Behavioral Health Integration for depression, anxiety and other drug use in addition to alcohol-related care. The trial sample included all patients seen for PC across 22 sites, January 2015-July 2018. Implementation strategies addressing Aim 2 included: 1) practice coaching to address stigma, knowledge gaps and support quality improvement, 2) electronic health record (EHR) decision support to prompt for assessment of DSM-5 AUDs and initiation of AUD treatment, and 3) performance monitoring and feedback on DSM-5 AUD assessment. The main Aim 2 outcome was a new AUD diagnosis followed by initiation and engagement in AUD treatment (initiation within 14 days, and engagement requiring 2 more visits in the following 30 days, based on ICD codes used for HEDIS measures). Analyses used general linear mixed models accounting for correlation of measures from the same person or site, adjusted for covariates including calendar time, and followed CONSORT standards.

**Findings:** 255,789 patients made 953,402 PC visits pre-implementation; 228,258 patients made 615,515 visits post-implementation. Comparing the main AUD treatment outcome per 10,000 PC patients with visits/month pre- versus post-implementation, revealed no significant change (pre- 1.8 versus post-1.4, p=0.30), despite significant increases from pre- to post-implementation in other process measures: DSM-5 AUD symptom assessment (4.1 versus 81, p < 0.0001), new AUD diagnosis (29 versus 34, p 0.003), and new AUD diagnoses with treatment initiation (6.1 versus 7.8, p 0.0.042). Among 2,719 patients with new AUD diagnoses, 899 (33%) initiated and 394 (14%) engaged in treatment.

**Implications for D&I Research:** Evidence-based implementation strategies increased DSM-5 AUD assessment and AUD treatment initiation as part of Behavioral Health Integration, but most patients with new AUDs did not initiate treatment. Increasing engagement in AUD treatment will require additional strategies.

**Primary Funding Source**

Agency for Healthcare Research and Quality

### S11 Sustainment of alcohol-related care following implementation of a multi-faceted intervention in primary care

#### Julie Richards^1^, Amy Lee^1^, Jennifer Bobb^1^, Evette Ludman^1^, Carol Achtmeyer^2^, Gwen Lapham^1^, Malia Oliver^1^, Ryan Caldeiro^3^, Rebecca Parrish^3^, Emily Williams^4^, Joseph Glass^1^, Paula Lozano^3^, Katharine Bradley^5^

##### ^1^Kaiser Permanente Washington Health Research Institute, Seattle, WA, USA; ^2^VA Puget Sound, Veterans Health Administration, Seattle, WA, USA; ^3^Kaiser Permanente Washington, Seattle, WA, USA; ^4^HSR&D, Veterans Health Administration, Seattle, WA, USA; ^5^Kaiser Permanente, Seattle, WA, USA

###### **Correspondence:** Julie Richards (Julie.E.Richards@kp.org)

**Background:** Implementation of evidence-based care often fails to be sustained when resources supporting implementation are removed. The Sustained Program of Alcohol-related Care (SPARC) trial was designed by researchers and clinical leaders to enhance sustainability of the integration of alcohol-related care for primary care [PC] patients. This presentation describes sustainment of alcohol-related prevention and treatment. Specifically, this report compares SPARC implementation outcomes—rates of brief intervention [BI] for unhealthy alcohol use, and treatment of new alcohol use disorders [AUDs]—across implementation phases (pre-implementation, active implementation, and sustainment phases).

**Methods:** Three implementation strategies supported integration of alcohol-related care in Kaiser Permanente Washington PC clinics (N=22) using a stepped-wedge design (1/1/2015-7/30/2018): 1) practice coaching, 2) electronic health record [EHR] decision support, and 3) performance monitoring and feedback. Two main trial outcomes included prevalence of 1) documented BI following positive screens for unhealthy alcohol use and 2) initiation and engagement of AUD treatment among patients newly diagnosed with AUDs based on EHR data. Generalized linear models [GLM] adjusted for calendar time described the prevalence of outcomes per 10,000 patients with visits/month during three implementation phases for each clinic: pre-implementation (1/1/2015 until each clinic began implementation preparation), active implementation (4 months of practice coaching following each clinic’s randomly assigned start date), and sustainment (following active implementation until study end 7/30/2018). To allow statistical comparisons across presentations (abstracts 1-3) generalized linear mixed model [GLMM] analyses accounting for person and site-level correlation (now in progress) will be presented during the symposium.

**Findings:** The prevalence of documented BI following positive screening for unhealthy alcohol use, was 12 per 10,000 PC patients/month during the pre-implementation phase, compared to 98 during active implementation and 69 during the sustainment phase. The prevalence of AUD treatment among patients newly diagnosed with AUDs was 1.9 per 10,000 PC patients/month during the pre-implementation phase, compared to 1.4 during active implementation and 1.6 during the sustainment phase.

**Implications for D&I Research:** The multi-faceted implementation intervention was associated with an increased prevalence of documented BI during active implementation that appears to be partially sustained. AUD treatment did not meaningfully change across the 3 periods.

**Primary Funding Source**

Agency for Healthcare Research and Quality

### S12 Engaging front-line staff to implement a child mental health intervention in child welfare services

#### Geetha Gopalan^1^, Kerry-Ann Lee^2^, Caterina Pisciotta^1^

##### ^1^City University of New York, New York, NY, USA; ^2^University of Maryland, Baltimore, Baltimore, MD, USA

###### **Correspondence:** Geetha Gopalan (ggopalan@hunter.cuny.edu)

**Background:** Despite disproportionately high child behavioral difficulties, families involved in child welfare services (CWS) frequently struggle to access and utilize needed treatment in traditional child mental health settings. This study used a task-shifting approach to implement a child mental health evidence-based intervention (EBI; 4 Rs and 2 Ss for Strengthening Families Program) delivered by CWS caseworker in placement prevention services. Given the importance of implementation success on staff engagement or “buy-in”, we explored those factors which promoted or hindered frontline CWS staff buy-in.

**Methods:** Three cohorts of the modified EBI were implemented CWS placement prevention services, facilitated by CW caseworkers. Following completion of each cohort, qualitative data were collected from CW staff (n = 6 caseworkers, n = 4 superviors, and n = 2 administrators) via audio-recorded in-depth interviews (60-90 minutes). Qualitative analysis of written transcripts involved directed content analysis, compiling and applying *a priori* and emergent codes, and resolving coding discrepancies via discussion. Focused coding identified multiple themes within larger umbrella codes. For this paper, we focused on the theme of staff buy-in.

**Findings:** Factors promoting buy-in included the desire among caseworkers to increase their clinical skills, work with families in a non-coercive manner, and provide services known to help families. Additional factors included compensation for new tasks (financial, flexible hours). Finally aspects of the organization which promoted frontline staff engagement included buy-in from administration to reduce caseloads and provide additional manpower when needed, support from supervisors, and the ability share information about prior effort to support informed decision-making. CW frontline staff engagement was often hindered by the difficulty managing crisis-oriented workload tasks in addition to delivering the modified EBI, “negativity” and unpredictability of the organizational environment, concerns about fidelity assessment, and inertia against change. Notably, younger, less experienced caseworkers were perceived as easier to engage than older caseworkers likely to be more exhausted and overwhelmed.

**Implications for D&I Research:** Strategies to promote engagement include appeals to increasing clinical skills and the “mission” of helping families, as well as concrete incentives and organizational supports. Issues with workload management underscore the need for more strategies to improve efficiency while also attending to the impact of worker burn-out.

**Primary Funding Source**

National Institutes of Health

### S13 Navigating leadership changes in real-world implementation: Examples from three state child welfare systems

#### Lisa Saldana, Patricia Chamberlain, Naamith Heiblum, Jason Chapman

##### Oregon Social Learning Center, Eugene, OR, USA

###### **Correspondence:** Lisa Saldana (lisas@oslc.org)

**Background:** Child welfare systems (CWSs) employ thousands of social service staff and providers across states, to serve a highly vulnerable population—families that are system-involved for myriad social determinants of health. The CWS workforce is under-resourced, over-extended, and in need of evidence-based support. The R^3^ Supervisor Strategy was developed at the request of the New York City (NYC) CWS to help infuse the use of evidence-based strategies throughout the frontline staff interacting with CWS-involved families. Supervisors, as the primary point of ongoing training and quality assurance of caseworkers, are trained, coached, and certified to supervise using reinforcement of: (1) effort, (2) relationships and roles, and (3) small steps.

**Methods:** Originally implemented across all five boroughs in NYC, R^3^ now has been implemented in twelve counties in Tennessee, and four counties in Oregon. All three CWSs are run systematically different with NYC being city-administered, Tennessee state-administered, and Oregon county- administered. A HIPAA compliant-observation system was used to monitor fidelity of the workforce over time. Measures of organizational climate, citizenship, leadership, and attitudes were collected longitudinally. Qualitative interviews were conducted.

**Findings:** All three systems engaged in training and fidelity monitoring of frontline staff, with statistically significant improvements across time, but were influenced by outer context political factors which impacted system leader turnover and decision-making. NYC leadership change at the Mayorial level influenced who held the position of CWS Commissioner-- whose direction disrupted the implementation. Tennessee underwent three system leadership changes, yielding both negative and positive impacts. Under litigation, Oregon’s state leadership experienced slow turnover, causing disengagement between state and county leadership, subsequently impacting county progression through the implementation process. All three scenarios lead to disruption in sustainment. Nevertheless, fidelity measures and organizational surveys suggest the CWS staffs’ desire for and acceptance of a workforce intervention, and the positive impact of R^3^ on staff exposed to the model. Thus, lack of sustainment was not related to intervention success, but rather leadership decision-making.

**Implications for D&I Research:** Often driven by uncontrollable political changes, this presentation will describe the R^3^ team’s lessons learned for using implementation strategies to overcome barriers and make recommendations for tailoring of implementation strategies for system leadership.

**Primary Funding Source**

National Institutes of Health

### S14 Linking systems to implement substance use interventions in child welfare

#### Alicia Bunger^1^, Erica Magier^1^, Emmeline Chuang^2^, Amanda Girth^1^, Kathryn Lancaster^1^, Fawn Gadel^3^, Marla Himmeger^3^, Elinam Dellor^1^, Karla Shockley-McCarthy^1^, Christy Kranich^1^

##### ^1^Ohio State University, Columbus, OH, USA; ^2^UCLA Fielding School of Public Health, Los Angeles, CA, USA; ^3^Public Children Services Association of Ohio, Columbus, OH, USA

###### **Correspondence:** Alicia Bunger (bunger.5@osu.edu)

**Background:** Fueled by the opioid crisis, states are experiencing an increase in the number of children entering the child welfare system because of caregivers’ substance use concerns. Interventions like START (Sobriety Treatment and Recovery Teams) expedite substance use screening and referrals, and integrate child welfare and substance use services. However, START implementation is difficult in rural areas where there are few substance use treatment providers. This study examines contextual features associated with START implementation fidelity. Drawing on the Exploration, Preparation, Implementation, Sustainment (EPIS) framework, we expect stronger START fidelity in counties with treatment availability and formal cross-system partnerships.

**Methods:** We used a multiple holistic case study design, with participating counties as the unit of analysis. Of the 17 counties in southeastern Ohio implementing START since 2018, all but 5 are rural and/or Appalachian. Data included agency documents (e.g. contracts between child welfare agencies and substance use treatment providers), longitudinal surveys of child welfare staff (to assess changes in referral practices), and publicly available data on local substance use treatment availability. Fidelity was measured as average days between case intake and substance use screening within each county (as recorded in administrative data), with fewer days reflecting stronger fidelity. We identified high performing counties as those with better than average fidelity.

**Findings:** To date, 256 families received START, and were screened within 14 days on average. High performing counties (n=10) screened caregivers within seven days on average, whereas the other counties (n=7) screened within 18 days on average. Counties with strong implementation had fewer substance use treatment providers (an average of 5) compared to the other counties (an average of 11). Also, higher performing counties had more informal, referral-based partnerships compared to the other counties which had more formal partnerships. Results suggest that some child welfare agencies can leverage informal partnerships to overcome challenging system and organizational conditions during initial implementation.

**Implications for D&I Research:** While informal relationships may facilitate service coordination, our findings suggest formal partnerships may hinder implementation and expedited care, contrary to expectations. Understanding why, and whether this pattern holds as implementation matures can inform strategies for implementing START and requires future study.

**Primary Funding Source**

National Institutes of Health

### S15 Development of and model for the support center: Operationally-partnered internal facilitation to increase opioid use disorder treatment in VA primary care

#### Carol Achtmeyer^1^, Madeline Frost^2,3^, Anissa Danner^4^, Aline Lott^4^, Carly Hood^4^, Carol Malte^4,5^, Andrew Saxon^4^, Christopher Vanderwarker^6^, Eric Hawkins^7^, Emily Williams^2^

##### ^1^VA Puget Sound, Veterans Health Administration, Seattle, WA, USA; ^2^HSR&D, Veterans Health Administration, Seattle, WA, USA; ^3^Health Services Research & Development (HSR&D) Center of Innovation for Veteran-Centered and Value-Driven Care, Veterans Affairs (VA) Puget Sound Health Care System, Veterans Health Administration, Seattle, WA, USA; ^4^VA Center of Excellence in Substance Addiction Treatment and Education, Seattle, WA, USA; ^5^Health Services Research and Development (HSR&D), Veterans Affairs (VA) Puget Sound Health Care System, Seattle, WA, USA; ^6^VA Puget Sound Health Care System, Seattle, WA, USA; ^7^Department of Psychiatry and Behavioral Sciences, University of Washington, Seattle, WA, USA

###### **Correspondence:** Carol Achtmeyer (carol.achtmeyer@va.gov)

**Background:** Buprenorphine and injectable naltrexone are first-line medications to treat opioid use disorder (MOUD) in outpatient settings and associated with reductions in both overdose and suicide but are substantially underused outside of specialty-care. The Veterans Health Administration’s (VA) National Center for Patient Safety (NCPD) funds Patient Safety Centers of Inquiry to develop, disseminate and implement clinical innovations that improve patient safety. In 2017, opioid safety, and prevention of adverse drug events and suicide were highlighted as top NCPD priorities. We describe how we capitalized on these national operational priorities to create local clinical partnerships and obtain operational funding to support an internal facilitation approach to implement MOUD in primary care.

**Methods:** We partnered with local clinical leaders from primary care and addictions treatment to identify needs and priorities of the affiliated primary care clinic regarding opioid safety. In response to identified needs we obtained NCPD funding for the **SU**pporting **P**rimary care **P**roviders in **O**pioid **R**isk reduction and **T**reatment (**SUPPORT**) Center. Funding supports dedicated internal facilitators to support primary care providers in identifying patients with OUD and provide direct patient-centered care, including OUD assessment and buprenorphine initiation and maintenance using a stepped care approach rooted in the Chronic Care Model. Internal facilitators include an ARNP buprenorphine-waivered prescriber and a licensed social worker with addictions expertise who provide MOUD and behavioral support to patients and consult with providers. Internal facilitators also facilitate implementation of MOUD in primary care with strategies borrowed from the practice coaching literature, specifically employing humble inquiry to link and align themselves with and train interested clinical staff and create an environment of collaboration toward shared goals.

**Findings:** Capitalizing on an opportunity to obtain operational funding for quality improvement efforts which aligned with local and national clinical priorities supported embedded internal facilitation to support implementation of OUD assessment and provision of MOUD.

**Implications for D&I Research:** Implementation efforts may be bolstered when supported by clinical care dollars, focused on implementing innovations that are priorities for national and local clinical leaders, and facilitated internally with direct clinical care supplemented by practice coaching concepts.

**Primary Funding Source**

Department of Veterans Affairs

### S16 Rapid mixed-methods formative evaluation to identify and act on implementation barriers and facilitators and optimize integration of opioid use disorder treatment in primary care

#### Emily Williams^1^, Madeline Frost^1,2^, Anissa Danner^3^, Aline Lott^3^, Carol Achtmeyer^4^, Carly Hood^3^, Carol Malte^3^, Andrew Saxon^3^, Christopher Vanderwarker^5^, Eric Hawkins^6^

##### ^1^Department of Health Services, University of Washington School of Public Health, Seattle, WA, USA; ^2^Health Services Research & Development (HSR&D) Center of Innovation for Veteran-Centered and Value-Driven Care, Veterans Affairs (VA) Puget Sound Health Care System, Veterans Health Administration, Seattle, WA, USA; ^3^VA Center of Excellence in Substance Addiction Treatment and Education, Seattle, WA, USA; ^4^Health Services Research & Development; Primary and Specialty Medical Care Service, VA Puget Sound, Veterans Health Administration, Seattle, WA, USA; ^5^VA Puget Sound Health Care System, Seattle, WA, USA; ^6^Department of Psychiatry and Behavioral Sciences, University of Washington, Seattle, WA, USA

###### **Correspondence:** Emily Williams (Emily.Williams3@va.gov)

**Background:** Formative evaluation (FE) is a rigorous assessment process enabling greater understanding of implementation contexts and the experiences of those directly affected by implementation. The VA’s operationally-funded **SU**pporting **P**rimary care **P**roviders in **O**pioid **R**isk reduction and **T**reatment (**SUPPORT**) Center uses rapid mixed-methods FE to understand user context and experiences, identify barriers and facilitators, and refine internal facilitation implementation strategies to increase provision of first-line medications for opioid use disorder (MOUD). We describe FE data sources, processes and outcomes, and resulting iterative refinements to internal facilitation during the initial six months of implementation.

**Methods:** FE processes include weekly meetings between implementation/evaluation and clinical teams and ongoing rapid mixed-methods data collection and analysis. Three data sources are used: 1) EHR and patient tracking data, 2) surveys and semi-structured interviews with primary care providers, and 3) minutes from weekly meetings. EHR and patient tracking data are updated weekly and reviewed in weekly meetings. Qualitative interviews and meeting minutes are iteratively coded using the Consolidated Framework for Implementation Research. Results are used to inform and adapt internal facilitation implementation strategies.

**Findings:** FE results suggest that organizational context (required opioid tapering and incentives to prescribe MOUD); dedicated SUPPORT clinical staff providing consultation and delivering timely patient-centered care; delivery of buprenorphine waiver and informal trainings by addictions experts that build provider engagement and knowledge; and primary care providers sharing success stories have facilitated implementation. Patient complexity (mental health and medical comorbidities), challenges to obtaining prescribing privileges, and provider knowledge gaps (e.g., how to identify and diagnose OUD) and beliefs (e.g., stigma) have been barriers. While planned internal facilitation strategies included providing consultation and clinical services, buprenorphine waiver trainings, and development and provision of EHR templates, early FE findings refined these strategies to include additional educational services (e.g., weekly office hours), direct marketing of OUD treatment availability to patients, facilitation of credentialing/privileging processes, and timely electronic consults with SUPPORT clinical staff.

**Implications for D&I Research:** Rapid FE using triangulated qualitative and quantitative data can identify and address implementation barriers and capitalize on implementation facilitators in real time to facilitate increased capacity for and interest in MOUD treatment in primary care.

**Primary Funding Source**

Department of Veterans Affairs

### S17 Early implementation outcomes from the supporting primary care providers in opioid risk reduction and treatment (SUPPORT) center

#### Eric Hawkins^1,2^, Madeline Frost^3,4^, Anissa Danner^1^, Aline Lott^1^, Carol Achtmeyer^5^, Carly Hood^1^, Carol Malte^1^, Andrew Saxon^1^, Christopher Vanderwarker^6^, Emily Williams^3^

##### ^1^VA Center of Excellence in Substance Addiction Treatment and Education, Veterans Health Administration, Seattle, WA, USA; ^2^Department of Psychiatry and Behavioral Sciences, University of Washington, Seattle, WA, USA; ^3^Department of Health Services, University of Washington School of Public Health, Seattle, WA, USA; ^4^Health Services Research & Development (HSR&D) Center of Innovation for Veteran-Centered and Value-Driven Care, Veterans Affairs (VA) Puget Sound Health Care System, Veterans Health Administration, Seattle, WA, USA; ^5^Health Services Research & Development; Primary and Specialty Medical Care Service, VA Puget Sound, Veterans Health Administration, Seattle, WA, USA; ^6^VA Puget Sound Health Care System, Veterans Health Administration, Seattle, WA, USA

###### **Correspondence:** Eric Hawkins (Eric.Hawkins@va.gov)

**Background:** The **SU**pporting **P**rimary care **P**roviders in **O**pioid **R**isk reduction and **T**reatment (**SUPPORT**) Center uses internal facilitation with a stepped care approach based on the Chronic Care Model to support primary care providers in identifying patients at high risk for adverse opioid-related outcomes (e.g., those with opioid use disorder, or OUD) and providing medication treatment for OUD (MOUD). Services offered include buprenorphine waiver training, referrals for consultation, assessment and office-based MOUD, psychosocial services and ongoing support in MOUD maintenance after patients are stabilized by the Center. We describe the number of waiver trainings, number of providers waivered to prescribe buprenorphine and are prescribing, and the number of referrals, buprenorphine initiations and transfers back to primary care providers over the initial six months of implementation.

**Methods:** This prospective evaluation used provider training and VA pharmacy data to measure training and prescribing outcomes, as well as patient activity reports to measure referrals to the SUPPORT Center and patient transfers back to primary care providers for ongoing buprenorphine treatment. Training-related measures included number of waiver trainings and prescribers obtaining DEA waivers, and a referral measure reflected the number of total referrals to the SUPPORT Center. Prescribing measures included number of patients who initiated buprenorphine, number of patient transfers to primary care and number of primary care providers who prescribed buprenorphine during the initial six months.

**Findings:** Prior to the model’s launch in January 2019, a total of seven providers in primary care were waivered to prescribe buprenorphine for OUD, none of whom prescribed buprenorphine. Since launch, the SUPPORT Center has held two buprenorphine waiver trainings; 15 primary care providers have obtained DEA waivers since the initial training. The CCM team has received 44 referrals, and offered patient assessment, provider consultation, and care coordination. The CCM team initiated buprenorphine for 14 patients; 3 were stabilized and transferred back to primary care providers. Four of 15 providers have prescribed buprenorphine to one or more patients over the initial six months of implementation.

**Implications for D&I Research:** Internal facilitation with a stepped care model can substantially increase access to office-based MOUD in primary care. Identification of OUD remains challenging.

**Primary Funding Source**

Department of Veterans Affairs

## Building the Future of D&I Science: Training, Infrastructure, and Emerging Research Areas

### S18 Promoting d&I capacity with a safety net focus through the development of a center for implementation and improvement sciences

#### Erika Crable^1^, Allan Walkey^2^, Mari-Lynn Drainoni^3^

##### ^1^Boston University School of Medicine, Boston, MA, USA; ^2^Boston University, Boston, MA, USA; ^3^Boston University School of Public Health, Boston, MA, USA

###### **Correspondence:** Erika Crable (ecrable@bu.edu)

**Background:** The Evans Center for Implementation and Improvement Sciences (CIIS) was established in 2016 to promote scientific rigor in new and ongoing projects aimed at increasing the use of evidence and improving patient outcomes within an urban, academic, safety net medical center. Funded by and housed within the School of Medicine’s Department of Medicine, CIIS is a resource for researchers and practitioners interested in building capacity to conduct D&I research. CIIS is run by two co-Directors and one Research Fellow.

**Methods:** CIIS activities include: (1) conducting original D&I research with a safety net focus; (2) promoting D&I education through free, monthly seminars; (3) providing free technical assistance concerning implementation frameworks, study design and outcome measures; (4) providing pilot grant funding and mentoring to support investigators new to D&I. After 3 years of operation, CIIS conducted a self-evaluation to summarize the center’s delivery of core activities, and overall ability to build capacity for D&I research within an academic medical center.

**Findings:** Major challenges to increasing D&I capacity include: (1) physician preference for quality improvement approaches and general resistance to studying implementation processes; (2) limited funding to enhance center staffing at a level that meets demand for D&I expertise. Center successes include: (1) submission of 21 grant proposals (13 funded, 7 under review) built on collaborative research partnerships across diverse clinical settings; (2) development of 2 separate skills-building seminar series focused on foundational D&I concepts and bridging the D&I and quality improvement divide; (2) conducting 183 technical assistance consultations across schools of medicine, public health, dentistry, and social work; (3) fostering the development of a safety net hospital medical intensive care unit as a learning health system; (4) mentoring 3 cohorts of new D&I investigators via pilot and professional development studies.

**Implications for D&I Research:** There is increasing demand to support post-graduate and practitioner training in D&I approaches across a broad spectrum of medical and public health content areas. Summarizing the challenges and opportunities of D&I centers housed within academic medical centers will promote shared understanding of how to enhance workforce capacity within the broader D&I field.

### S19 An emerging implementation science core to build capacity among psychiatrists and psychologists at brown university

#### A. Rani Elwy (rani_elwy@brown.edu)

##### Alpert Medical School of Brown University, Providence, RI, USA; Alpert Medical School of Brown University, Providence, MA, USA

**Background:** The Department of Psychiatry and Human Behavior (DPHB) at Brown University launched a department-funded Implementation Science (IS) Core in July 2018, to foster the translation, spread, and scale-up of evidence-based practices into routine clinical care.

**Methods:** The IS Core works with psychiatry and psychology faculty and trainees across the six Brown-affiliated hospitals to 1) provide consultation on dissemination and implementation (D&I) science models, theories and frameworks to guide studies, 2) develop qualitative and quantitative methods specific to D&I science research questions, and 3) serve as investigators on implementation science projects. The IS Core is based on 8 essential elements for building D&I capacity in an academic setting, identified through a scoping review undertaken prior to launching this emerging program: mentorship, in-person workshops, institutional funding, learning communities, methodological training, competency-driven curricula, practice partnerships, and evaluation of its efforts. We adapted the knowledge translation survey by Park and colleagues (2018) to evaluate participants’ pre-post self-efficacy for practicing D&I science and knowledge of D&I during the first year of the IS Core.

**Findings:** Since July 2018, the IS Core has led 14 seminars and 4 in-depth workshops on key D&I topics, reaching 103 faculty and trainees. Thirty-five federal grant proposals with a D&I focus have been submitted, 10 of these have been funded, and 16 are under review. Eight NIH K or VA CDA proposals have also been submitted, each with primary or secondary D&I aims. Seventy-four one-on-one D&I mentoring sessions have occurred in this first year. The IS Core was represented at 11 invited D&I presentations at national or regional meetings, and as faculty on national D&I training programs. Knowledge translation of 69 faculty and trainees over the 1 year evaluation period indicated a more prepared academic workforce for D&I science activities.

**Implications for D&I Research:** DPHB at Brown University made an institutional investment in building capacity in implementation science, which has resulted in a substantial increase in D&I grant and career development proposal submissions, as well as an improvement in self-efficacy for and knowledge of D&I science. Further evaluation efforts will track changes in funding levels over time, as an indicator of return on investment.

### S20 Dissemination and implementation science program at the university of Colorado

#### Jodi Holtrop^1^, Russell Glasgow^2^

##### ^1^University of Colorado School of Medicine, Aurora, CO, USA; ^2^Family Medicine, University of Colorado School of Medicine, Aurora, CO, USA

###### **Correspondence:** Jodi Holtrop (jodi.holtrop@cuanschutz.edu)

**Background:** The University of Colorado houses the Dissemination and Implementation (D&I) Science Program within the Adult and Child Consortium for Health Outcomes Research and Delivery Science (ACCORDS). ACCORDS is a health services outcomes research center supported by the School of Medicine, Children’s Hospital Colorado and research funding. The program operates with a leader (Russell Glasgow), co-leader (Jodi Holtrop), five core faculty and a program staff support person. Weekly open meetings are sponsored to discuss key topics, recent publications, and critique research projects.

**Methods:** The D&I program provides educational services including: free 1-2 time consultations with a D&I scientist (currently five D&I faculty provide consultations) regarding methods, design, framework or other advice; seminars and workshops including a pragmatic research national conference in spring 2020; a graduate certificate program to begin January 2020; three graduate courses; and an NHLBI-funded K12 training program. Research funding currently comes from NIH, AHRQ, PCORI and foundations. A suite of online and interactive resources supports our focus on pragmatic D&I measures, methods and models. Key focus areas include shared decision making, primary care, geriatrics, qualitative and mixed methods, and health behavior change. In addition, D&I scientists serve as D&I experts for other’s research on a wide variety of topic areas.

**Findings:** The structure of having the D&I Program within ACCORDS provides for a free interchange with other methods and program areas across schools and disciplines. This makes it easy for researchers to find help with D&I. Although not housed within the University’s clinical translational science institute (called CCTSI at Colorado), its close affiliation facilitates graduate school offerings and provides a greater array of programs and resources. The structure of ACCORDS also makes it such that the support needed for center and training grants are possible, such as the K12 program. Current program challenges include the need for more senior D&I scientists because demand is outstripping availability for mentoring and co-I roles.

**Implications for D&I Research:** Enhanced D&I research capacity is needed to advance health-related interventions. Having a program structured within a larger health services delivery center is an effective model for interchange of ideas and access to D&I expertise.

### S21 The university of North Carolina’s approach to advancing implementation science: The dissemination and implementation methods unit

#### Jennifer Leeman^1^, Catherine Rohweder^2^, Sarah Birken^3^, Christopher Shea^4^

##### ^1^School of Nursing, University of North Carolina, Chapel Hill, NC, USA; ^2^University of North Carolina, Chapel Hill, NC, USA; ^3^Gillings School of Global Public Health, Chapel Hill, NC, USA; ^4^UNC Chapel Hill, Chapel Hill, NC, USA

###### **Correspondence:** Jennifer Leeman (jleeman@email.unc.edu)

**Background:** The current pool of implementation scientists is too small to meet the demand. Existing programs provide initial training and mentorship in implementation science. However, the graduates of these programs often have limited opportunities to collaborate with and learn from other implementation scientists. Thus, a pressing need exists to continue to mentor these new investigators while also introducing additional researchers to the field. We describe how the University of North Carolina (UNC) has worked to fill this gap, with a focus on its CTSA-funded Dissemination and Implementation (D&I) Methods Unit.

**Methods:** Members of the D&I Methods Unit traced UNC’s history with D&I and created an inventory of the organizational structures and strategies used to support implementation science. They also mapped how the D&I Unit bridges to other implementation science initiatives on campus and reviewed findings from the Unit’s annual evaluation of its processes and impact.

**Findings:** The D&I Methods Unit provides expert consultation and trainings, maintains an online portal of D&I resources, and conducts collaborative research to advance implementation science. Measures of unit processes and impact include number and types of consultations and trainings, new collaborations, publications, and submitted and funded research proposals. The Unit works closely with UNC’s Implementation Science Student Group and the dissemination and implementation arms of three of UNC’s multidisciplinary centers. We also will present investigators’ lessons learned from 20 years working to build capacity for implementation science.

**Implications for D&I Research:** We describe the strategies one university has used to meet the growing demand for expertise in implementation science.

### S22 Building capacity for dissemination and implementation research through stakeholder engagement in a clinical and translational science award institution: The university of Wisconsin story

#### Andrew Quanbeck^1^, Jane Mahoney^2^, Kim Kies^3^, Maureen Smith^4^

##### ^1^Department of Family Medicine and Community Health, University of Wisconsin – Madison, Madison, WI, USA; ^2^Department of Medicine, University of Wisconsin-Madison, Madison, WI, USA; ^3^University of Wisconsin Madison, Madison, WI, USA; ^4^Dept. of Population Health Sciences, University of Wisconsin-Madison, Madison, WI, USA

###### **Correspondence:** Andrew Quanbeck (arquanbe@wisc.edu)

**Background:** We report results of an 8-year process of stakeholder engagement aimed at building capacity in Dissemination and Implementation (D&I) research at the University of Wisconsin through the National Institutes of Health’s Clinical and Translational Science Award (CTSA). The stakeholder engagement process was guided by 1) the “Wisconsin Idea,” which holds that the university’s research should improve the health and quality of life of the citizenry, and 2) designing for dissemination, a concept stressing the importance of engaging stakeholders throughout the translational research spectrum to optimize the likelihood of successful dissemination.

**Methods:** Starting in 2011, annual individual interviews were held with leaders of the Wisconsin CTSA’s community engagement core for strategic planning purposes. Interviews were followed by annual in-person meetings that employed a facilitated group decision making process aimed at identifying and prioritizing gaps in the translational research spectrum.

**Findings:** The stakeholder engagement process has identified D&I as a primary gap limiting overall impact of the science deriving from the institution’s translational research spectrum. This finding led to inclusion of a dedicated D&I aim in the institution’s 2012 CTSA renewal application, and expansion of that aim in the 2017 renewal application. To build D&I capacity, the Wisconsin CTSA has created an array of resources falling into 6 categories: 1) Network building, with the community engagement core engaging 65 of the state’s 72 counties; 2) D&I grant funding, including 45 CTSA funded pilot grants; 3) Training & education, including a D&I short course averaging 80+ attendees annually; 4). Research support, including D&I consults with more than 500 researchers leading to 26 federal grants; 5) Support for translational activities to maximize likelihood of impact through provision of business and D&I services; and 6) Collection of data for evaluation and measurement of impact. The systematic process of stakeholder engagement has been repeated annually since 2011 and provides a method to continuously identify gaps and opportunities to increase impact through tailoring D&I resources to the institution’s needs.

**Implications for D&I Research:** This presentation highlights a model for building D&I capacity that CTSAs nationally could emulate by partnering with their Community Engagement cores, which are common to all CTSAs.

**Primary Funding Source**

National Institutes of Health

### S23 Beyond early adopters: Strategies for success in reaching and engaging diverse communities in implementation trials from the adaptive school-based implementation of CBT (ASIC) trial

#### Amy Rusch^1^, Seoyoun Choi^1^, Elizabeth Koschmann^2^, Celeste Liebrecht^1^, Kristen Miner^1^, Michael Prisbe^1^, Jennifer Vichich^1^, Amy Kilbourne^1^, Shawna Smith^1^

##### ^1^University of Michigan, Ann Arbor, MI, USA; ^2^University of Michigan Medical School, Ann Arbor, MI, USA

###### **Correspondence:** Amy Rusch (amyrusch@med.umich.edu)

**Background:** Implementation trials are often criticized for primarily engaging early adopter sites, thus limiting study generalizability. Better implementation science requires developing tactics for engaging stakeholders across the diffusion continuum in implementation research. Adaptive School-based Implementation of CBT (ASIC) is a large-scale randomized trial designed to optimize implementation strategies supporting school professional (SP) delivery of cognitive-behavioral therapy (CBT) in over 100 high schools across Michigan. Methods to recruit SPs for ASIC participation were analyzed and successful tactics for engaging participants beyond early adopters were identified.

**Methods:** ASIC schools were recruited over a 6-month period, and a post-hoc process evaluation was conducted. Metrics collected include quantitative measures (e.g., number of recruitment attempts) and qualitative feedback from recruiters. Following recruitment, data were analyzed to identify patterns in successful recruitment efforts and codify effective strategies for recruiting SPs to ASIC.

**Findings:** With a recruitment goal of 100 schools, recruiters reached out to SPs and administrators at 272 schools over 6 months; ultimately engaging 115 schools. The average SP required 5 contacts before consenting (range: 1-16). To help engage nonresponsive communities and harder to reach SPs, the study team mobilized seven mental health clinicians with experience working with school and community-based CBT and 30+ members of a statewide CBT coaching network. Leveraging these community partnerships significantly increased recruitment pace and diversity, increasing school recruitment numbers from 6 per month to 39 per month after these individuals were utilized. Community partners reported that SPs responded most favorably to recruitment efforts that assuaged potential barriers to CBT implementation in schools, such as heavy SP burden and limited professional benefit. Clinicians addressed these barriers by discussing past participant experiences, describing empirical effectiveness of CBT, and emphasizing program flexibility. Notably, SPs were less persuaded by study incentives ($330 over 18 months).

**Implications for D&I Research:** Applying strategies for effectively recruiting consumers to engage with EBPs is a critical step in disseminating and implementing novel practices in real world settings. Efforts to refine and be transparent about successful study recruitment strategies may improve the representation of community-based sites engaged in implementation studies beyond early adopters, and in turn, expand the generalizability of such studies’ findings.

**Primary Funding Source**

National Institutes of Mental Health

### S24 Global implementation research capacity building to address cardiovascular disease: An assessment of efforts in eight countries

#### Ana Baumann^1^, Annette Fitzpatrick^2^, Meredith Ford^3^, Mary Beth Weber^4^, Constantine Akwanalo^5^, Duc Ha^6^, Hoa Nguyen^7^, Mina Hosseinipour^8^, Jacob Plange-Rhule^9^, Helen Cox^10^

##### ^1^Washington University in St. Louis, St. Louis, MO, USA; ^2^Departments of Epidemiology, Family Medicine, Global Health, School of Public Health, University of Washington, Seattle, WA, USA; ^3^Colorado School of Public Health, Aurora, CO, USA; ^4^Emory University, Atlanta, GA, USA; ^5^Moi Teaching and Referral Hospital, Eldoret, Kenya; ^6^Vietnam Ministry of Health, Hanoi, Viet Nam; ^7^Baylor Scott & White, Dallas, TX, USA; ^8^Institute for Global Health and Infectious Diseases, University of North Carolina, Chapel Hill, NC, USA; ^9^Kwame Nkrumah University of Science and Technology, Khumasi, Ghana; ^10^National Heart, Lung, and Blood Institute ,National Institutes of Health Bethesda, MD, USA

###### **Correspondence:** Ana Baumann (abaumannwalker@wustl.edu)

**Background:** Cardiovascular disease (CVD) is the number one cause of death globally; more people die of CVDs than from any other cause, with most of these death occurring in low and middle income countries (LMICs). Hypertension (HTN) is a major risk factor for CVD and, while effective evidence-based interventions for control of HTN exist, they are not being implemented in LMICs. There is an acute need to build capacity in LMICs for implementation and dissemination of proven HTN management tools. To address this gap, a cohort of eight sites funded by the National Heart, Lung and Blood Institute, are collaborating to establish a research and training infrastructure in dissemination and implementation (D&I) to improve hypertension care.

**Methods:** The sites are: Ghana, Guatemala, India, Kenya, Malawi, Nepal, Rwanda and Vietnam. Using a capacity building framework developed by Potter and Brough (2004), we mapped the formal and informal activities that each site is doing to develop (a) structures, systems and roles, (b), staff and infrastructure, (c) skills, and (d) tools. In addition, we captured information from each site about their needs assessments and metrics.

**Findings:** All sites collaborate with the Ministry of Health and other local agencies, involving them at different levels and plan to engage stakeholders throughout the duration of the projects. Needs assessment were implemented in all countries, although the specific stakeholders integrated into projects varied. The training for D&I and the activities to develop skills in HTN research is variable across sites ranging from formal fellowships and courses to informal on-the job training. Five of the eight countries relied on Implementation Science frameworks including PRECEDE-PROCEED, RE-AIM, and CFIR.

**Implications for D&I Research:** While there is variability in the activities across sites in the skills and tools utilized to develop capacity to address CVD, sites seem to have more consistency in the activities related to developing structure, system and roles. The observation of activities of eight ongoing studies will allow us to develop a strategic plan to, in the short term, evaluate the best strategies to support capacity building for developing D&I skills and addressing the HTN care gap in LMICs.

**Primary Funding Source**

National Institutes of Health

### S25 Organizational communication: Public health program adoption, implementation, and fidelity

#### Oyinda Osibanjo (Oyinda.Osibanjo@oregonstate.edu)

##### Oregon State University, Hillsboro, OR, USA

**Background:** Organizational communication plays an important role in the translation of evidence-based interventions to real-world settings. However, few studies have examined communication processes within public health organizations implementing behavioral prevention programs. We examined patterns of participatory communication (PC) within Departments of Public Health (DPHs) and Community-Based Organizations (CBOs) implementing the HIV RESPECT program.

**Methods:** Using a maximum variation case-study design, ten organizations were sampled from a national survey of agencies implementing the RESPECT HIV program. Fifty organizational personnel were interviewed to identify PC patterns in specific program translation contexts such as program adoption and problem-solving contexts. Approximately 294 RESPECT clients were surveyed to assess program fidelity. Qualitative methods were used to analyze associative patterns between structural and contextual variables in relation to program fidelity.

**Findings:** We identified three PC patterns across agencies (full-, partial-, and non-PC). Small and medium sized CBOs that adopted RESPECT voluntarily, relative to DPHs or organizations mandated RESPECT, more often evidenced full PC across contexts. Full PC agencies consistently evidenced high program fidelity specifically in the implementation problem-solving context.

**Implications for D&I Research:** There is limited research that addresses the effect of specific program translation contexts on the patterns of PC and program fidelity; yet understanding the influence of specific program translation contexts on internal communication might contribute to effective program translation. Developing interventions to improve the quality of internal communication that takes into account specific program translation contexts has the potential to not only ensure positive program outcomes, but to also contribute to effective program adoption, implementation, and even the sustainability of EBIs. In addition, future intervention strategies should be directed at increasing PC, as well as promoting horizontal communication by improving the channel of internal communication within organizations.

**Primary Funding Source**

National Institutes of Health

### S26 Clinical routines as an under-explored yet critical component of context in implementation science

#### Miriam Bender^1^, Deborah Lefkowitz^2^

##### ^1^Sue & Bill Gross School of Nursing, University of California, Irvine, Irvine, CA, USA; ^2^School of Social Ecology, University of California, Irvine, Irvine, CA, USA

###### **Correspondence:** Miriam Bender (miriamb@uci.edu)

**Background:** Context is a fundamental concept in implementation science and considered a major source of facilitators and barriers to implementation. Yet context has been neither consistently nor comprehensively conceptualized in implementation science.

**Methods:** An implementation-effectiveness study examined the role of context in a complex nursing care delivery intervention delivered in 11 hospitals across 5 states. The intervention consisted in changing the nurse staffing model to incorporate Clinical Nurse Leaders (CNL). CNLs are Registered Nurses utilizing evidence-based competencies in clinical leadership, care environment management, and clinical outcomes management to improve the quality of frontline clinical processes. Interviews were conducted 2016-2019 with clinicians and administrators (n=380) along with 2-22 hours of observation of the implementation process per hospital. Data were analyzed using deductive and inductive qualitative content analyses.

**Findings:** One of the most consistent contextual components influencing implementation across settings was the clinical routine. In the organization science literature routines are defined as repetitive, recognizable patterns of interdependent actions, carried out by multiple actors. Although CNL interventions were developed based on evidence-informed competencies and agreed-on process gaps, they often clashed with pre-existing and well-functioning routines in unforeseen ways when implemented. For example, in some settings CNL facilitation of patient discharge unexpectedly impeded the routine for medical residents conferring with attending physicians. CNLs and residents worked on a day-of-discharge intervention that successfully improved patient flow. However, this intervention inadvertently changed the timing of the resident-attending conferral routine, which caused further problems by incrementally delaying other routines, such as patient rounding. These unintended consequences ultimately led to abandoning the discharge intervention so the resident-attending conferral routine could return to its original timing. While successful for patient discharge, the CNL intervention interfered with existing clinical routines in ways that had nothing to do with the original scope of the intervention.

**Implications for D&I Research:** Context matters. These findings suggest a complex causality between interventions and contexts that manifests via unanticipated intersections among existing multi-professional clinical routines. However, clinical routines are not listed as a component in existing context determinant frameworks. Further investigation and conceptualization is needed to advance knowledge about the causal significance of clinical routines for healthcare interventions in implementation science.

**Primary Funding Source**

Commission on Nursing Certification

### S27 Using a systematic continuous quality improvement process to improve the provision of medication-assisted treatment for opioid use disorder within rural primary care practices

#### Jack Warwick^1^, Ellen DiDomenico^2^, David Kelley^3^, Evan Cole^1^, Daniel Lomauro^1^, Adam Gordon^4^, Gerald Cochran^5^, Julie Donohue^6^, Walid Gellad^7^, Julie Kmiec^8^, Janice Pringle^1^

##### ^1^University of Pittsburgh, Pittsburgh, PA, USA; ^2^Pennsylvania Department of Drug and Alcohol Programs, Harrisburg, PA, USA; ^3^Pennsylvania Department of Human Services, Harrisburg, PA, USA; ^4^University of Utah, Salt Lake City, UT, USA; ^5^University of Utah School of Medicine, Salt Lake City, UT, USA; ^6^University of Pittsburgh Graduate School of Public Health, Pittsburgh, PA, USA; ^7^University of Pittsburgh Department of Medicine, Pittsburgh, PA, USA; ^8^University of Pittsburgh Department of Psychiatry, Pittsburgh, PA, USA

###### **Correspondence:** Jack Warwick (jswarwick@pitt.edu)

**Background:** The quality of substance use disorder (SUD) and mental health (MH) treatment varies widely. Continuous Quality Improvement (CQI) processes can be used to improve evidence-based practice delivery and quality. The Rural Access to MAT in Pennsylvania (RAMP) Project, an AHRQ-funded demonstration grant to support MAT for OUD in rural Pennsylvania, developed and utilized a systematic CQI process based in the Principles of Lean. The CQI process was implemented within practice sites using a framework for implementation called the Systems Transformation Framework (STF). The CQI process performance criteria were selected based on the most current HEDIS measures, clinical treatment guidelines, and published literature. This presentation will describe the RAMP CQI process and review preliminary findings related to MAT implementation.

**Methods:** The CQI process with recruited rural primary care practices in Pennsylvania involved a cycle of four steps occurring in quasi-real-time: (1) collection of encounter data on a pragmatic set of performance measures; (2) analysis and synthesis of performance data; (3) regular reporting of treatment engagement, adherence, and retention data compared to performance benchmarks and other participating providers; and (4) routine collaborative discussion of performance illustrated by the data and identified areas for improvement supported via concierge technical assistance. Qualitative data was collected through provider interviews and quantitative data was obtained through patient encounter data.

**Findings:** Based on quantitative and qualitative findings, we identified and provided feedback to improve MAT implementation in quasi-real-time in several key areas: (1) coordination of care; (2) adherence to clinical prescribing guidelines, workflows, and policies; and (3) awareness of patient engagement in and adherence to treatment. Preliminary findings from four practice sites participating in CQI suggest a 12% to 83% change on average in MAT/SUD/MH treatment engagement before and after a quality improvement intervention. We expect this trend in engagement to continue and to have statistically significant results analyzed by October 2019.

**Implications for D&I Research:** Utilization of a novel quasi-real-time, systematic performance evaluation process, like the RAMP CQI process, can provide valuable insight in quasi-real-time into areas for quality improvement during implementation, allow for process adaptation and enhancement in a timely manner, and ultimately may lead to improved treatment outcomes.

**Primary Funding Source**

Agency for Healthcare Research and Quality

### S28 Predictors of mis-implementation of chronic disease control programs in state health departments

#### Margaret Padek^1^, Peg Allen^2^, Stephanie Mazzucca^1^, Emily Weno^3^, Ross Brownson^4^

##### ^1^Prevention Research Center, Washington University in St. Louis, St. Louis, MO, USA; ^2^Brown School, Prevention Research Center, Washington University in St. Louis, St. Louis, MO, USA; ^3^Washington University in St. Louis, St Louis, MO, USA; ^4^Division of Public Health Sciences, Department of Surgery, Washington University in St. Louis, St. Louis, MO, USA

###### **Correspondence:** Margaret Padek (mpadek@wustl.edu)

**Background:** Much of the disease burden in the United States is preventable through optimal application of existing knowledge. State-level funders and practitioners are in ideal positions to affect programs and policies related to chronic disease but it is unknown the extent to which mis-implementation is occurring with these programs. Mis-implementation refers to ending effective programs and policies prematurely or continuing ineffective ones. Previous research has suggested that approximately 58% of public health programs are evidence-based.

**Methods:** We developed and tested a comprehensive survey to assess the extent to which mis-implementation was occurring in state health departments (SHDs) and to examine what individual, organizational, and external factors are related to mis-implementation. Questions were developed from previous literature and cognitive testing. Surveys were emailed out to 1,329 randomly selected state health department employees across the Unites States working in prevention and control of non-communicable conditions.

**Findings:** The survey received a 50% response rate with representation from every state. Half (50.7%) were program managers or unit directors and only 36% of the state health department respondents possessed a formal public health education. Forty-nine percent reported that their SHD sometimes, often or always continued ineffective programs. Over 50% also reported that their SHD sometimes or often ended effective programs. The strongest predictors of mis-implementation were at the organizational level. For example, the number of organizational layers that impede decision-making (OR 2.9, CI 1.3-6.6, p=0.01) or an agency’s willingness to change to use evidence-based interventions (OR 0.59, CI 0.38-0.91, p=0.02). An individual level factor that predicted mis-implementation was the respondent’s ability to modify interventions between populations (OR 3.95, CI 1.325-11.751, p=0.01).

**Implications for D&I Research:** The data suggest that agency practices can be addressed to maximize the effectiveness and efficiency of programs. Further research will focus on adding context to these issues and helping agencies engage in appropriate decision-making through developing case studies and agent-based models. Through future collaborative studies, D&I researchers can further public health practitioner understanding of how to de-implement ineffective interventions and sustain implementation of effective programs and policies. Thus countering mis-implementation and ultimately improving health outcomes.

**Primary Funding Source**

National Institutes of Health

### S29 I-corps@ncats: A novel designing-for-dissemination learning laboratory for clinical and translational researchers to increase intervention relevance and speed dissemination

#### Kathryn Nearing^1,2^, Julie Rainwater^3^, Elaine Morrato^4,5^, Stacey Neves^3^, Pamela Bhatti^6^, Nathaniel Hafer^7^, Suhrud Rajguru^8^, Bruce Conway^9^, Kevin Harter^10^, Molly Wasko^11^

##### ^1^University of Colorado Anschutz Medical Campus, Aurora, CO, USA; ^2^Eastern Colorado Geriatric Research Education and Clinical Center, Veterans Health Administration, Aurora, CO, USA; ^3^University of California, Davis, Sacramento, CA, USA; ^4^Colorado Clinical and Translational Sciences Institute, Aurora, CO, USA; ^5^Colorado School of Public Health, University of Colorado Anschutz Medical Campus, Aurora, CO, USA; ^6^Georgia Institute of Technology, Atlanta, GA, USA; ^7^UMMS, Worcester, MA, USA; ^8^University of Miami, Miami, FL, USA; ^9^The Rockefeller University, New York, NY, USA; ^10^Penn State University, Hershey, PA, USA; ^11^University of Alabama Birmingham, BIRMINGHAM, AL, USA

###### **Correspondence:** Kathryn Nearing (kathryn.nearing@ucdenver.edu)

**Background:** Research innovations that are technology-driven/feature-focused may fail to demonstrate strong value (relevance) if not also responsive to adopters’/stakeholders’ problems and implementation contexts. The federal *ROI Initiative for Unleashing American Innovation* endorsed expansion of NSF’s Innovation Corps™ (I-Corps™) program to train researchers in a rigorous, repeatable customer discovery process for studying, and designing for, implementation ecosystems. In 2018, NIH’s National Center for Advancing Translational Sciences (NCATS) funded the adaptation and implementation of I-Corps at six clinical translational science award (CTSA) sites (UC-Davis, UC-Denver, UAB/Georgia Tech, Miami, Penn State, UMass) to accelerate research translation into clinical-/community-based practice.

**Methods:** The 5-week I-Corps@NCATS training (8 cohorts, 62 teams, 150 individuals) included in-person didactics (“Kick-off”); national/regional instructors delivered presentations on core concepts, how to identify and prioritize key adopters/stakeholders and conduct customer discovery (target=30 interviews). In the immersive learning-laboratory, teams completed interviews while receiving weekly coaching. Participants reconvened for a “Finale” where they presented the value of their innovation for specific end-users in a defined ecosystem and modifications for enhanced dissemination. Mixed-methods evaluation assessed program implementation, reach, effectiveness. Evaluators observed Kick-offs, completing detailed observation protocols/fidelity checklists. Teams completed surveys following the Finale (55 teams) and 3-12 months post training (34 teams). All instructors participated in virtual focus groups to reflect on program implementation and identify feasibility/sustainability issues to inform broader CTSA dissemination.

**Findings:** Teams pursued medical devices (33%), drugs/biologics (20%), software applications (16%) and diagnostics (8%). All teams rated customer discovery very important (mean=4.7 on a 5-point scale); respondents reported that I-Corps helped them hone innovation relevance (product-market fit) and identify requirements for successful dissemination. Teams reported increased readiness for implementation and commercialization over time; 39% of teams reported meeting with tech transfer to pursue licensing/patents; 24% reported pursuing venture capital/investor funding.

**Implications for D&I Research:** I-Corps@NCATS training anchors intervention development in deep understanding of specific adopters’ needs and ecosystem requirements for D&I success. I-Corps builds D&I capacity by educating researchers to respond to issues affecting the individual adopter (e.g., How does this fit into my workflow? How is it better than what I do currently? How much will it “cost” me – time, money?), thus maximizing the impact of innovation.

**Primary Funding Source**

National Institutes of Health

### S30 Training implementation research in Rwanda: Evaluation, challenges and lessons learned

#### Ana Baumann^1^, Cole Hooley^1^, Vincent Mutabazi^2^, Angela Brown^1^, Dominic Reeds^1^, W. Todd Cade^1^, Lisa de las Fuentes^1^, Enola Proctor^3^, Stephen Karengera^2^, Kenneth Schechtman^1^, Charles W. Goss^1^, Pascal Launois^4^, Eugene Mutimura^2^, Victor G Davila-Roman^1^

##### ^1^Washington University in St. Louis, St. Louis, MO, USA; ^2^Regional Alliance for Sustainable Development (RASD) Rwanda, Kigali, Rwanda; ^3^George Warren Brown School of Social Work, Washington University in St. Louis, St. Louis, MO, USA; ^4^World Health Organization, Geneva, MO, Switzerland

###### **Correspondence:** Ana Baumann (abaumannwalker@wustl.edu)

**Background:** Cardiovascular disease (CVD) is the leading cause of morbidity and mortality in low- and middle-income countries (LMIC), and hypertension (HTN) is a major risk factor for CVD. Although effective evidence-based interventions for control of HTN exist, implementation of these in low- and middle-income countries is challenging due to limited capacity and infrastructure. In Rwanda, CVD is the third most common cause of mortality. Suboptimal infrastructure hinders research and community knowledge for HTN control. We report on a project aiming to develop research capacity in D&I to support implementation of interventions to control HTN-CVD in Rwanda.

**Methods:** First, a weeklong training program in HTN-CVD, biostatistics, and D&I was conducted in Rwanda in August 2018. Pre- and post-D&I training competency questionnaires were administered to participants. Second, stakeholders participated in a massive open online course (MOOC) developed by the Special Programme for Research and Training in Tropical Diseases at the World Health Organization to train in implementation research. Participants answered pre and post D&I competencies. D&I competencies were measured by a scale developed for a US-based training program, with the change in competency scores assessed by paired t-test. Feasibility was measured by use of MOOC training completion data (i.e., completion of homework assignments, quizzes, final exam and final project), and analyzed using descriptive statistics.

**Findings:** Questionnaires from the weeklong training in 2018 showed a statistically significant increase in D&I knowledge and skills (full scale pre- to post-test scores:2.12 ± 0.78 vs. 3.94 ± 0.42; p < 0.0001). Of the 92 trainees enrolled, 38% completed all MOOC components. D&I competency scores showed statistically significant improvements from pre- to post-test.

**Implications for D&I Research:** In the context of LMIC training, the weeklong training session and the MOOC course were feasible and showed improvement in D&I competencies. While the program was designed with a focus on training for tropical diseases (e.g., malaria), there is potential for scalability to a wider audience of stakeholders interested in D&I.

**Primary Funding Source**

National Institutes of Health

### S31 Transferring & sustaining implementation science knowledge and skills through a multifaceted training experience

#### JoAnn Kirchner (JoAnn.Kirchner@va.gov)

##### Central Arkansas Veterans Healthcare System (North Little Rock), Veterans Health Administration, North Little Rock, AR, USA

**Background:** Knowledge transfer of evidence-based implementation strategies supports the uptake of clinical innovations in research and quality improvement initiatives and can lead to sustained practice change. We developed an implementation facilitation (IF) training program guided by i-PARIHS to transfer IF knowledge/skills to operations leaders, researchers and implementation practitioners within and outside the VA. This training blends didactic and interactive learning (including flipped classrooms and interactive role play) to reinforce key concepts. In-person and video teleconference options are available to increase the reach of the training program.

**Methods:** An ongoing independent mixed-methods evaluation assesses training outcomes and informs ongoing refinements/adaptations. Evaluation includes pre-/post-surveys approximately 2 weeks before and 2 weeks and 6 months after each training, and qualitative interviews at 6 months. Surveys collect demographic information and assess IF training domains. To analyze training effectiveness, mean self-reported IF knowledge of and confidence in applying IF skills are compared to baseline. Qualitative interviews identify the application and/or adaptation of IF skills and areas for training enhancement.

**Findings:** Of 169 trainees since 2015, 104 (62% response rate) completed both pre-/post-surveys; 59 (67% response rate) completed 6-month interviews since 2017. Trainees reported increased knowledge of factors influencing innovation adoption, roles/activities of external and internal facilitators and site champions and best practices in providing facilitation through virtual platforms. Knowledge of and confidence in applying IF skills significantly increased and were sustained at 6-months. There were no significant differences in knowledge change scores for virtual trainees. Trainees report direct (i.e., on a specific project) and/or indirect applications of IF skills to their work. Recommendations for improvement include increasing applicability of the training to non-VA settings, adding time for project consultation, and additional training on evaluation.

**Implications for D&I Research:** Implementation scientists are increasingly encouraged to handoff evidence-based strategies to implementation practitioners including front line managers, policymakers and providers so these skills can be incorporated into clinical practice. This evaluation shows how our multifaceted training effectively transfers IF knowledge/skills to stakeholders with sustainability. IF training can be provided through virtual platforms without loss of knowledge transfer. This IF training program provides an example how scale-up and spread of evidence-based strategies has been accomplished.

**Primary Funding Source**

Department of Veterans Affairs

### S32 Designing for facilitator resilience in implementation science: A conceptual framework

#### Tanya Olmos-Ochoa^1^, David Ganz^2^, Jenny Barnard^1^, Lauren Penney^3,4^, Erin Finley^5^, Alison Hamilton^6,7^, Neetu Chawla^8^

##### ^1^VA Greater Los Angeles Healthcare System, Veterans Health Administration, Los Angeles, CA, USA; ^2^Center for the Study of Healthcare Innovation, Implementation & Policy (CSHIIP), V.A. Greater Los Angeles Healthcare System, Veterans Health Administration, Los Angeles, CA, USA; ^3^Medicine, University of Texas Health San Antonio, San Antonio, TX, USA; ^4^South Texas Veterans Affairs Healthcare System, Veterans Health Administration, San Antonio, TX, USA; ^5^VERDICT, South Texas Veteran Health Care System, Veterans Health Administration, San Antonio, TX, USA; ^6^Department of Veterans Affairs, Veterans Health Administration, Los Angeles, CA, USA; ^7^University of California Los Angeles, Los Angeles, CA, USA; ^8^Veterans Affairs Greater Los Angeles Healthcare System, Veterans Health Administration, Los Angeles, CA, USA

###### **Correspondence:** Tanya Olmos-Ochoa (tanya.olmos@va.gov)

**Background:** Practice facilitation is increasingly used by healthcare systems as an effective strategy to enable staff to identify and capitalize on factors associated with implementation success. Extant literature has focused on defining the facilitator role (skills/attributes, training) and the facilitation process (internal/external, duration and frequency of facilitation encounters) in relation to implementation outcomes in one-time facilitation efforts. Consequently, limited work addresses how implementation success and facilitation effectiveness are impacted when facilitators become embedded in the healthcare system and work on multiple projects over time. Our preliminary framework identifies factors that affect facilitators over time, including their ability to cope with the intensity of the facilitation process and to sustain their effectiveness across implementation projects.

**Methods:** Our framework was informed by: a review of the published facilitation literature, particularly articles from the facilitator’s perspective; and empirical data (templated facilitator reflections and debrief session notes) from the Coordination Toolkit and Coaching project, a VA-funded initiative to improve patient experience of care coordination in primary care. Since August 2017, two facilitators have conducted weekly, one-hour coaching calls with each of six clinics implementing self-selected quality improvement projects. Facilitation is ongoing, with 245 calls completed to date.

**Findings:** Our framework builds on the i-PARIHS constructs for successful implementation –innovation, recipients, context, and facilitation– and adds *facilitation resilience* and *facilitator intensity* to capture the dynamic and relational aspects of facilitation over time. We define *facilitator resilience* as facilitators’ ability to cope and adapt to the complexities of facilitation to effectively engage and motivate staff while nurturing and sustaining their own hope, self-efficacy, and adaptive coping behaviors. We define *facilitation intensity* as a quantitative and/or qualitative measure of the volume of tasks and activities needed to engage and motivate recipients in implementation, and the psychological impact on the facilitator of delivering these facilitation tasks and activities.

**Implications for D&I Research:** Facilitators who can withstand the demands of facilitation acquire vital institutional and facilitation knowledge. Accounting for the impact of facilitation intensity on facilitator resilience in the design of implementation efforts may help support continued improvement, prevent facilitator burnout, and sustain a skilled facilitator workforce, which is critical to the future of D&I research.

**Primary Funding Source**

Department of Veterans Affairs

### S33 Transforming innovations into market-ready products: An entrepreneurial approach to d&I science

#### Roberto Martinez^1^, Darcy Freedman^2^, Victoria Avi^1^, Haley Tolerton^1^, Rachel Sommer^1^

##### ^1^Case Western Reserve University, Cleveland, OH, USA; ^2^Case Western Reserve University, Mary Ann Swetland Center for Environmental Health, Cleveland, OH, USA

###### **Correspondence:** Roberto Martinez (ram249@case.edu)

**Background:** In the current public health discovery-to-practice process, internal validity of interventions takes priority over external validity due to funding and publication priorities, and input from users of the interventions is seldom sought. SPeeding Research-tested INterventions Training (SPRINT), sponsored by the National Cancer Institute, is a training program for researchers with the goal of transforming interventions into “market-ready products” to increase uptake. During their participation in SPRINT, the authors applied business research theory to the FreshLink Ambassador (FLA) program, which trains community members to promote use of nutrition incentives by SNAP recipients at farmers’ markets. The program was implemented during 2017-2018 at markets in Ohio and was then prepared for dissemination throughout the US through a “train-the-trainer” program. This abstract describes the results and illustrates how the application of business research theory may improve D&I science, producing interventions which are more context-specific and financially self-sustaining.

**Methods:** Using the Business Model Canvas (BMC) methodology, 70 “customer discovery interviews” (qualitative unstructured interviews) were performed with stakeholders relevant to the implementation of the FLA program, such as SNAP-ED state directors, farmers’ market managers, health department staff, and USDA leaders. The interviews were used to test “value propositions” (assumptions inherent to the planned intervention), identify “customers” (potential participants), and ultimately develop a “Minimal Viable Product,” or effective intervention most suitable to the context of potential “customers.”

**Findings:** Stakeholders recommended that the intervention be offered to any organization accepting SNAP and providing nutrition incentives, not just farmers’ markets. Additionally, some potential “customer” organizations, such as health departments, reported that the program did not align with their strategic priorities. Stakeholders also gave feedback regarding the logistics (i.e. location, duration) of the proposed training. Overall, the data continues to be used in an iterative process to significantly revise the plan for D&I, including A. modifying the “product” (intervention) design, and B. developing financial projections of the “product.”

**Implications for D&I Research:** Applying the BMC to the FLA program prior to D&I allowed for stakeholder input that will produce an intervention more relevant to the context and needs of the stakeholders, overall increasing the likelihood of success of the intervention.

**Primary Funding Source**

National Cancer Institute

### S34 Scaling up and disseminating faith in action: Discoveries from the sprint training

#### Elva Arredondo, Jessica Haughton, Jacqueline Montañez

##### San Diego State University, San Diego, CA, USA

###### **Correspondence:** Elva Arredondo (earredon@sdsu.edu)

**Background:***Faith in Action* is an evidence-based intervention (EBI) implemented by community health workers (i.e., *promotoras*) that increases the physical activity of churchgoing Latinas. The current presentation highlights the information and skills gained from participating in the National Cancer Institute’s SPeeding Research-tested INTerventions (SPRINT) training program. SPRINT provides training on the use of business models, including the Value Proposition Canvas and Business Model Canvas, to gain insights about the needs of community partners and customers (i.e., payers) that health programs serve.

**Methods:** Our team conducted in-person and phone interviews with 58 key stakeholders including pastors, study participants, community health workers *(i.e., promotoras)*, organizational leaders, denominational leaders, and physical activity advocates.

**Findings:** Key findings from the SPRINT interviews suggest that: 1) pastors need to be empowered to implement program activities and facilitate connections with outside organizations, 2) hospitals, federally qualified health centers (FQHCs), the local health department, and community organizations (e.g., Catholic Charities, YMCA) see value in and expressed interest in supporting health prevention programs that engage underserved communities as these programs increase their visibility and align with their fiscal priorities, 3) building strategic partnerships with national networks and associations (physical activity, faith-based, parks and recreational centers) can support national scale up, 4) it is important to know the training, implementation, and maintenance costs prior to scale up, and 5) involving the denominational leadership can optimize program implementation, dissemination and sustainment.

**Implications for D&I Research:** In the presentation, we discuss how these discoveries inform the scale up and dissemination of *Faith in Action*. Understanding the value that *Faith in Action* adds to community partners and customers will help transform this EBI into a market ready product. The SPRINT training can help behavioral scientists move behavioral interventions into practice more rapidly.

**Primary Funding Source**

National Cancer Institute

### S35 The customer discovery “sprint”: Lessons from spreading play streets to all

#### Keshia Pollack Porter^1^, M. Renée Umstattd Meyer^2^, Tyler Prochnow^2^

##### ^1^John Hopkins University, School of Public Health, Baltimore, MD, USA; ^2^Baylor University, Waco, TX, USA

###### **Correspondence:** Keshia Pollack Porter (kpollac1@jhu.edu)

**Background:** Speeding Research-tested Interventions Training (SPRINT) is a National Cancer Institute training program for researchers with the goal of transforming interventions into “market-ready products” to increase uptake and dissemination. We participated in SPRINT 2019 to support the spread of Play Streets -- place-based interventions that are typically organized by community organizations and involve temporarily activating public spaces, often closing streets, to create safe places and free opportunities for physically active play. Prior to SPRINT, we planned to spread Play Streets to every community and hypothesized that decision-makers in community-based organizations would value promoting child health and physical activity and choose to implement Play Streets to do so.

**Methods:** We conducted 57 customer discovery interviews (72% in-person and 28% conducted via phone or video) with stakeholders from around the world. Stakeholders included key partners, competitors, and potential customers to learn about their values, possible interest in implementing interventions that promote active play for youth, resources needed to support implementation, and their ability to pay for interventions or technical assistance to support implementation. Notes were taken during the interviews and findings were synthesized to determine customer segments, value propositions, and the best model to support uptake and sustainability of relevant interventions.

**Findings:** Rather than spread our intervention to all communities, we narrowed our focus to rural U.S. communities. We initially considered 9 different customer segments and through our customer discovery interviews realized several of these customer segments are partners or potential competitors. Based on the data, we determined it was best to narrow our focus to two customer segments within rural communities – local health departments and Agri-Life Extensions. We also learned these customers would not be able to pay for consultation to support implementation, and that we need to seek funds to support a re-granting model to provide seed money to support implementation and technical assistance.

**Implications for D&I Research:** Customer discovery interviews generate important information critical to the dissemination process, more than is normally collected during traditional formative research. The information generated will support optimal implementation and long-term sustainability of a physical activity intervention highly relevant to the needs and values of organizations in rural communities.

**Primary Funding Source**

National Cancer Institute

## Clinical Care Settings: Patient-level Interventions

### S36 Preventing diabetes with benefit-based tailored treatment: Implementation of an individualized risk calculator in electronic health records at two u.s. health systems

#### Elizabeth Ciemins^1^, John Cuddeback^1^, Jason Nelson^2^, Jill Powelson^1^, Francis Colangelo^3^, Carolyn Koenig^4^, Anastassios Pittas^2^, David Kent^2^

##### ^1^AMGA, Alexandria, VA, USA; ^2^Tufts Medical Center, Boston, MA, USA; ^3^Premier Medical Associates, Pittsburgh, PA, USA; ^4^Mercy Health, St. Louis, MO, USA

###### **Correspondence:** Elizabeth Ciemins (eciemins@amga.org)

**Background:** With 1/3 of U.S. adults at risk for diabetes, health systems need to target patients most likely to benefit from proven interventions. Providing patients their individual estimated risk of developing diabetes presents an opportunity for shared decision making, which may improve treatment adherence. In electronic health records (EHRs) at two health systems, we implemented a predictive model that provides persons at risk for diabetes with individualized benefit estimates for taking metformin or participating in the Diabetes Prevention Program (DPP) intensive lifestyle intervention (DPP-ILI).

**Methods:** The calculator was implemented at 10 primary care clinics. Provider and patient focus groups informed the implementation process. The model estimates risk of progression to diabetes with usual care, the DPP-ILI, or metformin, based on 11 demographic, biometric and diagnosis variables available in the EHR. The implementation was evaluated using the RE-AIM framework for Reach, Efficacy, Adoption and Maintenance of the intervention. Balance measures, e.g., preventive care screening rates, were also assessed.

**Findings:** The predictive model was used on 65% (n=2,425) and 49% (n=710) of patients with prediabetes at two health systems between 5/1/18 and 5/31/19. Treatment – either referrals for DPP-ILI or prescriptions for metformin – was provided for 69% and 29% of high-risk, 3% and 21% of moderate-risk, and 2% and 6% of low-risk patients, respectively, at the two systems. DPP-ILI referrals and metformin prescriptions for these high-risk patients increased substantially after implementation of the calculator (DPP: 0% to 52% and 0% to 12%; Metformin: 6% to 16% and 3% to 17%) at the two study sites, respectively. At one site, 97 new patients were diagnosed with diabetes.

**Implications for D&I Research:** Successful implementation of predictive risk calculators is challenging. The processes followed to implement at two health systems had many similarities but important differences, underscoring the necessity of recognizing and exploring the context in which implementation occurs. The shared decision making with patients who are aware of their specific probabilities of developing diabetes with and without treatment may increase adherence to proven interventions, especially to intensive options such as the DPP-ILI.

**Primary Funding Source**

Patient-Centered Outcomes Research Institute

### S37 Implementation of diabetes group visits and text messaging in midwestern community health centers: Diabetes messages study (medical care, education, social support, and goal-setting to empower self-management)

#### Erin Staab^1^, Jefferine Li^1^, Cynthia Schaefer^2^, Amanda Campbell^3^, Sara Siddiqui^1^, Michael Quinn^1^, Arshiya Baig^1^

##### ^1^University of Chicago, Chicago, IL, USA; ^2^University of Evansville, Evansville, IN, USA; ^3^Midwest Clinicians’ Network, East Lansing, MI, USA

###### **Correspondence:** Erin Staab (estaab@medicine.bsd.uchicago.edu)

**Background:** Diabetes group visits (GVs) have been shown to improve outcomes but research in the community health center (CHC) setting is limited.

**Methods:** Teams from 6 CHCs implemented 6 monthly diabetes GVs including the “core 4” elements of medical visits, education, social support, and goal setting. GV patients were also enrolled in a self-management text-messaging program. HC teams attended 2 in-person training sessions and 9 monthly webinars before and during implementation to learn about the GV model, discuss barriers, and share experiences. Session logs completed after each GV tracked patient attendance and GV content. Pre- and post-implementation surveys assessed staff preparedness, satisfaction, and perceptions of the potential impact of GVs and text messaging on quality of care, patient outcomes, and costs.

**Findings:** All teams completed the 6 GVs and enrolled patients in text messaging. All GVs included education and most included individual medical visits (85%), goal setting (78%), and goal follow-up (63%). GVs were 60-180 minutes long (median 120 minutes). HCs adjusted the order of activities to fit space, personnel, and time available and chose education topics based on patient feedback. The number of patients at each GV ranged from 2 to 12 (median 5.5). Cold weather and transportation were reported as barriers to attendance at all CHCs. Still, 61% of patients attended 3 or more GVs. Teams shared strategies to improve retention during training sessions. Training significantly improved staff preparedness, motivation, and knowledge. After implementing GVs, staff reported greater satisfaction providing care to patients with diabetes (4.33 vs. 3.77, p<0.001) and rated the potential impact of GVs higher for improving quality of diabetes care (4.87 vs. 4.42, p=0.01) and reducing racial/ethnic disparities in diabetes outcomes (4.20 vs. 3.85, p=0.01). Ratings of text messaging were lower after implementation. CHCs encountered technical difficulties using the text messaging program and desired more customization.

**Implications for D&I Research:** Training and co-learning with other CHC teams supported successful implementation of GVs. The model was adapted to local resources and patient needs but retained its “core 4” elements. Staff reported enjoying GVs and expected they would improve care. Future analyses will compare processes of care and outcomes for GV and control patients.

**Primary Funding Source**

HHS Office of Minority Health

### S38 Testing implementation strategies for the autism support checklist: A pilot study

#### Coral Bays-Muchmore, Belinda Tanuwijaya, Lauren Bartolotti, Shari King, Marilyn Augustyn

##### Boston Medical Center, Boston, MA, USA

###### **Correspondence:** Coral Bays-Muchmore (coralbm@uw.edu)

**Background:** To better serve patients with Autism Spectrum Disorder (ASD), we implemented the Autism Support Checklist (ASC), a summary memo in patients’ electronic medical record (EMR). ASCs were obtained by interviewing caregivers about their youth’s communication, sensory, and safety needs in healthcare settings. This is an ongoing hybrid pilot study designed to (1) evaluate two implementation strategies (active/passive communication) to achieve uptake and (2) determine the acceptability of the ASC and its impact on improving patient and caregiver experience by modifying clinician behaviors.

**Methods:** We employed an embedded mixed methods design (QUAN + qual) to evaluate individual-level (e.g. satisfaction, acceptability) and structural-level (e.g. fidelity) outcomes. We searched for upcoming appointments for patients who had ASCs and randomly assigned their clinicians to either an active (in-person training) or passive (email only) approach. Clinicians completed a web-based quantitative survey with embedded qualitative measures following the active/passive intervention and patient appointment. We also collected quantitative and qualitative data from patient caregivers via a phone survey following their appointment. Thematic analysis was conducted to help explain quantitative findings.

**Findings:** To date we have enrolled 16 clinicians and 12 families and we will continue collecting data through September 2019. Compared to email-only clinicians, in-person training clinicians were more likely to report modifying their behavior and the difference was statistically significant. In-person training clinicians also reported higher rates of utilization, satisfaction, and likelihood to recommend ASC to other clinicians and patients, although none of these were statistically significant. Email-only clinicians reported being unable to find the information in the EMR, whereas this was not a reported barrier by the in-person training clinicians. Other common barriers reported by both groups included information being too long and lack of time to read it. Patient caregivers reported similarly satisfied outcomes across both clinician groups regarding avoidance of identified sensory triggers and ability for clinician and patient to communicate.

**Implications for D&I Research:** Findings support prior research highlighting the value of a multifaceted strategy including an active training component to improve uptake of a clinical tool. Findings also identify key barriers to successful uptake and implementation of individualized patient-needs information.

**Primary Funding Source**

Center for Implementation and Improvement Sciences (CIIS)

### S39 Exploring barriers and facilitators to implementing text messaging to facilitate communication between healthcare providers and patients living with HIV in South Carolina

#### Virginia A. Fonner, Samuel Kennedy, Christie Eichberg, Lisa Martin, Eric G. Meissner

##### Medical University of South Carolina, Charleston, SC, USA

###### **Correspondence:** Virginia A. Fonner (fonner@musc.edu)

**Background:** As falling out of HIV-related care can have dire health consequences for patients and public health implications for HIV transmission, retaining patients in care is critical. We hypothesize that expanding patient-provider communication to allow for text messaging in our HIV clinic in South Carolina will result in greater patient satisfaction and retention, as staff are currently restricted to communicating with patients using landline telephones. To address this hypothesis and guide intervention development, we conducted formative interviews with patients and providers to understand texting preferences and barriers/facilitators to implementation.

**Methods:** Semi-structured in-depth interviews were conducted with 12 individuals receiving HIV-related care and 14 clinic providers, including case managers and pharmacists. Interviews were recorded and transcribed verbatim. The Consolidated Framework for Implementation Research (CFIR) shaped development of interview guides, coding structure, and data analysis, which included thematic comparisons across CFIR domains between patients and providers.

**Findings:** Most patients (n=11/12) and providers (n=12/14) supported adding text message communication. Both groups felt text messaging would help meet patients’ needs and increase convenience and efficiency but differed in perceptions of complexity and compatibility. Providers expressed concern about potential overuse by patients and creating false expectations of instant availability, thus adding burdens to existing workflows. Providers also worried about the consequences of patients texting about protected health information (e.g., compliance violations), whereas patients trusted providers to discretely deliver information and felt comfortable with the risks of having sensitive information on their phones, provided it was delivered through a secure texting application (10/12 preferred a secure application to plain texting). Some providers perceived text messaging as redundant given existing platforms, such as the online patient portal, whereas patients viewed the patient portal as too difficult to use and perceived texting as a convenient, less-cumbersome alternative.

**Implications for D&I Research:** While patients and providers mostly agreed on implementation barriers and facilitators, important differences emerged. Taking both perspectives into account when utilizing implementation frameworks such as CFIR is critical for developing successful interventions, especially for those requiring active participation from providers and patients. Findings are currently being used to design the text messaging intervention to address provider and patient needs.

**Primary Funding Source**

Viiv Healthcare

### S40 Healthy hearts for Oklahoma: Impact of implementing quality improvement intervention bundle on cardiovascular care

#### Ann Chou^1^, Ji Li^1^, Juell Homco^2^, Xi Chen^1^, Zsolt Nagykaldi^1^, James Mold^1^, Daniel Duffy^2^, Steven Crawford^3^, Julie Stoner^1^

##### ^1^University of Oklahoma Health Science Center, Oklahoma City, OK, USA; ^2^University of Oklahoma School of Community Medicine, Tulsa, OK, USA; ^3^Univeristy of Oklahoma, Oklahoma City, OK, USA

###### **Correspondence:** Ann Chou (ann-chou@ouhsc.edu)

**Background:** Preventing cardiovascular disease (CVD) has been challenging in many medical underserved areas (MUA). While many initiatives promote the implementation of ABCS (aspirin therapy, blood pressure control, cholesterol management, and smoking cessation) measures, primary care practices lack quality improvement (QI) support and resources to achieve meaningful targets. The Healthy Hearts for Oklahoma (H2O) Study implemented a bundled QI intervention to increase and evaluate adherence to ABCS measures in primary care practices across Oklahoma.

**Methods:** H2O partnered with public health agencies and communities to implement a bundled QI intervention to improve ABCS performance in 263 primary care practices. The intervention bundle included: (1) physician-led academic detailing, (2) performance feedback, (3) practice facilitation by trained and certified facilitators, (4) health information technology and exchange support, and (5) a listserv for sharing best practices and delivering educational material. The study employed a stepped-wedge design where intervention was rolled-out in four waves and quarterly ABCS outcomes data were collected. Changes in ABCS performance was estimated using mixed-effects linear regression modeling over time post-intervention relative to control period.

**Findings:** Compared to baseline, all ABCS measures improved over time. Performance for aspirin therapy increased by 0.063 (95% CI: 0.044-0.081, p<0.0001) 12-18 months, and by 0.087 (95% CI: 0.037-0.138, p=0.0008) 24-30 months under intervention. Performance for blood pressure control increased by 0.043 (95% CI: 0.03-0.056, p<0.0001) 12-18 months, and by 0.049 (95% CI: 0.015-0.084, p=0.005) 24-30 months under intervention. Performance for cholesterol management increased by 0.09 (95% CI: 0.015-0.166, p=0.02) 12-18 months, and by 0.157 (95% CI: 0.034-0.280, p=0.02) 24-30 months under intervention. Performance for smoking cessation increased by 0.167 (95% CI: 0.14-0.193, p<0.0001) 12-18 months, and by 0.165 (95% CI: 0.093-0.236, p<0.0001) 24-30 months under intervention.

**Implications for D&I Research:** The H2O program was designed to improve cardiovascular health and outcomes for more than 1.25 million Oklahomans. The QI bundle developed and deployed increased organizational capacity to improve ABCS performance and can serve as critical resources to assist small, rural practices in adapting to the ever-changing healthcare environment and delivering quality care to their communities. Lessons learned from this project can guide future strategies for dissemination and implementation of evidence-based practices.

**Primary Funding Source**

Agency for Healthcare Research and Quality

### S41 Effects of a decision aid on increasing clinicians’ discussions of cost among patients with atrial fibrillation

#### Celia Kamath, Mrs. Kasey Boehmer, Rachel Giblon, Aaron Leppin, Juan Pablo Brito Campana, Nilay Shah, Victor Montori

##### Mayo Clinic, Rochester, MN, USA

###### **Correspondence:** Celia Kamath (kamath.celia@mayo.edu)

**Background:** Achieving patient-centered care for patients with atrial fibrillation requires that clinicians engage in discussions with patients about the cost of various treatment options. Unfortunately cost conversations (CC) between clinicians and patients are not routine and little is known about how to implement them in practice. As part of an ongoing hybrid effectiveness-implementation trial, we sought to examine the effect of a decision aid on the rate of cost conversations relative to standard care. We also sought to examine additional individual and contextual factors that facilitated or hindered CC.

**Methods:** We reviewed 658 video recorded clinical encounters at 3 of the 5 sites enrolled in the Anticoagulation Choice Trial (R01HL131535). We identified the presence or absence of a CC between patients and clinicians in each video. We then conducted logistic regression with presence/absence of CC as our dependent variable, and DA use, contextual (practice location) and patient (demographic, risk factors, social determinants) factors as our independent variables. Independent variables were prospectively collected as part of the trial. We accounted for clinician clustering in our model.

**Findings:** CC were observed in 203 (65.3%) and 273 (86.4%) encounters in standard care and DA arms respectively. DA use was associated with greater likelihood of CC (DA arm: β =1.37 (95% CI 0.90 to 1.85) versus standard care; p<.0001); Practice setting also mattered (site1: β =0.91 (95% CI: 0.11 to1.72) versus site 2; p=0.02). Qualitative analyses of CC themes are ongoing and the full mixed methods analyses of this data will be available by October 2019.

**Implications for D&I Research:** Decision aids have long been used to induce shared decision making. When appropriately designed, our findings suggest they might be effective strategies for influencing clinician behavior more broadly, including facilitating conversations about treatment costs. Our findings suggest that contextual factors like practice culture may also influence these discussions. D&I researchers should consider the potential role of encounter decision aids as implementation strategies for facilitating diverse clinician behaviors in clinical encounters and explore innovative designs to “nudge” conversations in patient-centered directions. They may also explore the role of context in supporting these novel strategies.

**Primary Funding Source**

National Institutes of Health

### S42 “I learned a lot, it was very eye opening”: Nursing home provider perspectives on implementing a person-centered communication intervention

#### Katherine Abbott^1^, Alexandra Heppner^1^, Kimberly Van Haitsma^2^

##### ^1^Miami University, Oxford, OH, USA; ^2^The Pennsylvania State University, University Park, PA, USA

###### **Correspondence:** Katherine Abbott (abbottkm@miamioh.edu)

**Background:** The purpose of this study was to assess the implementation of a novel person-centered care (PCC) communication tool in Ohio nursing homes (NH). PCC is a philosophy that recognizes “knowing the person” and honoring individual preferences. The communication tool is based on an assessment of NH resident likes and dislikes via the *Preferences for Everyday Living Inventory* (PELI), which is an evidenced-based, validated instrument used to enhance the delivery of PCC. The Ohio Department of Medicaid mandated NHs use the PELI as one of the factors that determine their daily Medicaid reimbursement rate.

**Methods:** The Preferences for Activity and Leisure (PAL) Card was developed to communicate important resident preferences across care team members and approved by the Ohio Department of Aging as a Quality Improvement Project. Providers were recruited to create PAL cards for 15-20 residents. To understand the intervention characteristics and processes associated with effective implementation, telephone interviews were conducted with providers who completed the project. Calls were recorded, transcribed verbatim and checked for accuracy. The Consolidated Framework for Implementation Research (CFIR) was utilized as an a priori coding scheme to identify factors associated with effective implementation.

**Findings:** A total of *n=43* providers registered and *n=26* (60%) providers completed the project, which involved monthly coaching calls and an end of project telephone interview. Participating providers were not for profit 46% (*n=12*), for profit 46% (*n=12*), and government owned 8% (*n=2*). Participants attempted *n=439* PAL card interviews with residents and completed *n=414* (94.31%) PAL Cards. Major themes emerging from the data related to the evidence strength and quality of the intervention as well as the relative advantage to not assessing preferences (“turns out she doesn't even like TV and we have just been having her watch TV”), adaptability (tailoring for their needs), trialability (expanding offering of intervention after initial success), and complexity of the intervention (sharing the work across departments, difficulty using unfamiliar technology).

**Implications for D&I Research:** The majority of providers were successful in implementing PAL Cards for residents with substantial support from the project manager. Providers reported positive intervention characteristics of the PAL cards, however, barriers remain that require additional strategies to successfully implement.

**Primary Funding Source**

Ohio Department of Medicaid

### S43 Implementing integrated reproductive health care into a school based health center setting

#### Lisa Masinter^1^, Megan Erskine^2^, Molly Klare^3^, Feinglass Joe^3^

##### ^1^AllianceChicago, Chicago, IL, USA; ^2^Heartland Health Center, Chicago, IL, USA; ^3^Northwestern University, Chicago, IL, USA

###### **Correspondence:** Lisa Masinter (lmasinter@alliancechicago.org)

**Background:** Many efforts are underway to facilitate improved utilization of high quality sexual and reproductive health services (SRHS) among adolescents in the United States. In Chicago, one network of school-based health centers (SBHCs) chose to implement an integrated model of reproductive healthcare, emulating integrated behavioral healthcare, rather than uptrain primary care providers (PCPs) in SRHS. The aim of this project was to assess changes in contraceptive provision after implementation.

**Methods:** This model includes a certified nurse midwife (CNM) staffing the SBHCs intermittently and receiving “warm handoffs” from PCPs to provide longer counseling sessions and dedicated procedure time. Electronic medical records were utilized from before (2016) and after (2018) implementation to assess change in contraceptive prescriptions, using chi-square tests of significance. A Poisson regression model was utilized to estimate the incidence rate ratio (IRR) for the likelihood of receiving long acting reversible contraception (LARC; intrauterine devices and subdermal implants).

**Findings:** The results revealed significant differences in prescription types between 2016 and 2018. The percentage of LARC prescriptions (implants and IUDs) rose from 0.6% to 3.9% (p<0.001). The percentage of prescriptions for emergency contraception and oral contraceptives increased from 9.8% to 24.1% and 14.5% to 30.2%, respectively (p<0.001). The percentage of patch prescriptions rose from 2% to 7.2% (p<0.001), while there was a significant decrease in injections, falling from 43.7% of prescriptions in 2016 to 27.1% in 2018 (p<0.001) and no change in prescriptions for the contraceptive ring. A significant increase in number of prescriptions was also noted in patients under 18 after implementation of this model, and the regression model indicated that students were 17.4 times more likely to get LARC in 2018 as compared to 2016 (p<0.001).

**Implications for D&I Research:** Access to high quality SRHS, specifically LARC, remains an implementation challenge in primary care settings, and these preliminary data suggest that the integrated reproductive health care model may facilitate the uptake of a wider variety of contraceptive methods, at a younger age, in a SBHC setting. However, further studies are needed to rigorously evaluate the impact on both health and patient reported outcomes, including patient satisfaction and measures of patient centered care.

### S44 Best practices for implementing complex healthcare interventions: A cluster-analysis of implementation strategies used during medication reconciliation implementation in acute care hospitals.

#### Deonni Stolldorf, Kemberlee Bonnet, David Schlundt

##### Vanderbilt University, Nashville, TN, USA

###### **Correspondence:** Deonni Stolldorf (deonni.stolldorf@vanderbilt.edu)

**Background:** Medication reconciliation (MedRec), the process whereby patients’ medication orders are documented and verified, reduces unintentional medication discrepancies (UMDs). Implementation strategies, which are challenging in real-world settings, can help to translate clinically efficacious multi-component interventions into local settings. Using data from a multi-site implementation of MedRec interventions, our goal was to identify and describe the combinations of implementation strategies used by the sites.

**Methods:** Interviews were conducted with implementation teams from hospitals (n=18) that participated in an AHRQ-funded study of MedRec implementation to reduce UMDs. Interview transcripts were coded using a hierarchical coding system developed employing an inductive/deductive approach. The frequencies of each implementation strategy at each participating site were calculated and used in a hierarchical cluster analysis (Ward’s method with squared Euclidian distances) to group strategies across sites.

**Findings:** Implementation strategies clustered into seven groups which were named: Role Preparation, Metrics and Methods, Adaptive Problem Solving, Role Adaptation, Engagement, Consensus Building, and Quality Improvement (Table 1). Cluster analysis of the 18 hospitals indicated three clusters: process focused, procedural focused, people focused.

**Implications for D&I Research:** Hospitals implementing MedRec interventions can use these strategic clusters to guide their efforts. Hospital implementation teams can focus their implementation on processes, procedures, or people to select the group of strategies most suited to their organizations.

**Primary Funding Source**

Agency for Healthcare Research and Quality

**Table 1 (abstract S44).** Hospital clusters, Implementation Strategy Clusters, and Implementation Strategies

Clusters	Implementation Strategies	Hospital Clusters		
		Process	Procedure	People Focused
**Role Prep**	Identify and prepare champions	X		
	Facilitate relay of clinical data to providers			
**Metrics and Methods**	Recruit, designate, and train staff		X	
	Change records systems			
	Change workflow systems			
**Adaptive Problem Solving**	Tailor strategies to overcome barriers and honor preferences			
	Assess for readiness and identify barriers			
	Use mass media			
	Use a quality improvement / implementation advisor			
**Role Adaptation**	Use advisory boards and workgroups		X	
	Adaptation of existing interventions			
	Revise professional roles			
**Engagement**	Involve executive boards and/or sponsors	X	X	X
	Build a coalition			
	Conduct educational meetings			
**Consensus Building**	Conduct local consensus discussions			X
	Individualized training sessions			
	Inform local opinion leaders			
**Quality Improvement**	Audit and provide feedback	X		X

*Note:* X= Most often used implementation strategy clusters by hospital clusters

### S45 Reducing variability in discharge communication reduces administrative burden

#### Stephanie Lumpkin, Ian Kratzke, Clark Howell, Nicole Chaumont

##### University of North Carolina - Chapel Hill, Chapel Hill, NC, USA

###### **Correspondence:** Stephanie Lumpkin (stephanie.lumpkin@unchealth.unc.edu)

**Background:** Streamlined discharge communication in transitions of care improvement models improve patient satisfaction, improve patient self-efficacy, and reduce readmissions. Yet, significant variability and quality exists in the After Visit Summary (AVS) which is used to guide patients, caregivers, and non-surgical providers about the post-discharge needs of the patient. Notably, a poor quality AVS often leads to increased confusion, administrative burden, and worse team dynamics. We sought assess the feasibility, fidelity, acceptability, and effectiveness of a standardized, patient-centered AVS.

**Methods:** Our stakeholder team included surgical interns, advanced practice providers, patient-advisors, nurse managers, floor-nurses, quality improvement coaches, health literacy experts, and a project manager. This one-year Gastrointestinal Surgery Readmission Reduction Program was led by two physicians and had significant institutional support. We employed a tailored, hybrid implementation strategy, blending components from Lean methodology, the Institute for Healthcare Model for Improvement, and the Consolidated Framework for Implementation Research. We assessed the feasibility (e.g. quality of AVS, usability of template), fidelity (e.g. template utilization rates), and acceptability (e.g. direct stakeholder feedback, paging-system audit, patient call logs), and effectiveness (e.g. 30-day readmission rates and patient-centeredness) of our intervention (e.g. standardized, patient-centered AVS). We used a seasonally-adjusted interrupted time series to assess the effect of the intervention on the number of weekly pages.

**Findings:** Our AVS template was written at a third grade reading level but retained all clinically-relevant information. Providers and patients approved of the overall content, format, and utility, with 66% mean template utilization (range 41% to 80%). After our intervention, we observed decreased 30-day readmissions (19.7% to 16.0%). Notably, after introducing the AVS template, pages sent decreased weekly at a rate of 3.67 pages (95% CI = [−15.7, −14.0]), but this was not significant. As a balancing measure, the number of patient calls in the post-intervention period remained manageable and clinically-appropriate.

**Implications for D&I Research:** Reducing variability and improving the quality of discharge communication, not only improves patient outcomes, but may also improve workflow for providers. These improved team dynamics may lead to improved buy-in, pull, and readiness for change and are thus important to assess when considering an intervention that is operationalized by time and resource deprived-trainees and employees.

**Primary Funding Source**

University of North Carolina Institute for Healthcare Quality Improvement

### S46 How do patients respond to de-implementing a medication that is potentially harmful?

#### Toral Parikh^1,2^, Krysttel Stryczek^3^, Christopher Gillespie^4^, George Sayre^2,5^, Barbara Majerczyk^1^, Edmunds Udris^1^, Laura Feemster^1,2^, Seppo Rinne^6^, Renda Wiener^4^, Christian Helfrich^7^, David Au^7^

##### ^1^VA Puget Sound Healthcare System, Veterans Health Administration, Seattle, WA, USA; ^2^University of Washington, Seattle, WA, USA; ^3^VA Northeast Ohio Healthcare System, Veterans Health Administration, Cleveland, OH, USA; ^4^Center for Healthcare Organization and Implementation Research (CHOIR), Veterans Health Administration, Bedford, MA, USA; ^5^Seattle-Denver Center of Innovation (COIN), Center for Veteran-Centered and Value-Driven Care, Veterans Health Administration, Seattle, WA, USA; ^6^VA Center for Healthcare Organization and Implementation Research, Veterans Health Administration, Bedford, MA, USA; ^7^VA Puget Sound Health Care System, Veterans Health Administration, Seattle, WA, USA

###### **Correspondence:** Toral Parikh (parikht@uw.edu)

**Background:** De-implementing medications presents challenges for providers and patients, potentially distressing patients or creating mistrust. However, little research has assessed such efforts for inhaled corticosteroids (ICS): commonly prescribed to patients with mild/moderate COPD but not recommended. We sought to understand how patients responded when de-implementing an ICS was recommended.

**Methods:** We conducted a mixed-methods analysis as part of a provider-randomized quality improvement project testing a proactive e-consultation from pulmonologists recommending to discontinue ICS among patients with mild/moderate COPD if appropriate. PCPs (n=137) at 13 VHA primary care clinics were included. We completed 20 interviews with PCPs: 14 who were unexposed to the intervention and 6 who were exposed. We interviewed 8 patients within 3 months of a visit where a recommendation to de-implement their ICS had been made. We conducted inductive and deductive thematic analysis of the qualitative data. Forty-eight PCPs returned surveys (24 exposed and 24 unexposed, response rate: 35%).

**Findings:** Unexposed PCPs perceived patients to be resistant to discontinuing ICS and were reluctant to discontinue or change an ICS if the patient perceived a benefit. PCPs also had misgivings about making a change when there was no perceived problem, “*if someone is doing well, why rock the boat?*” Intervention exposed PCPs reported that patients were receptive to the medication change, especially in the context of potential harm. However, some PCPs did report hesitation from patients who felt the ICS was helping. Patients mostly reported deferring to their PCPs for medication changes. However, one patient described ICS as “*a security blanket”* that they preferred to hold on to. In surveys, 54% of PCPs reported having proposed discontinuing or reducing an ICS for a patient in the past 6 months. Of this group, 90% reported that their patients responded somewhat or very receptively, and 80% discontinued the ICS or switched to another medication.

**Implications for D&I Research:** While PCPs were concerned with how patients would respond to discontinuing an ICS, interviews with exposed PCPs and surveys suggested patients were receptive to this change. For future de-implementation strategies, it may be possible to use these findings to address provider concerns regarding anticipated patient reactance.

**Primary Funding Source**

Department of Veterans Affairs

### S47 Development of a self-assessment tool to measure fidelity to a pharmacy service to optimize medication use

#### Carrie Blanchard^1^, Melanie Livet^1^, Kelly Sheppard^1^, Todd Sorensen^2^, Mary McClurg^1^

##### ^1^University of North Carolina - Chapel Hill, Chapel Hill, NC, USA; ^2^University of Minnesota College of Pharmacy, Minneapolis, MN, USA

###### **Correspondence:** Carrie Blanchard (carriebm@email.unc.edu)

**Background:** A consistent approach to delivery of pharmacy services is critical to achieve expected clinical outcomes and facilitate scale up. To ensure these services are being delivered as intended, fidelity should be measured as a key implementation outcome. The purpose of this study is to develop and validate a tool to evaluate pharmacists’ self-reported adherence to the comprehensive medication management (CMM) patient care process, a patient-centered approach to optimizing medication use.

**Methods:** As part of a larger study, a well-defined protocol for CMM was developed and formed the basis for the CMM Patient Care Process Fidelity Assessment (CMM PCPFA). The CMM PCPFA was initially vetted for content validity with seven pharmacists using Rubio el al.’s (1) methodology. A pretest of the tool was conducted with 42 pharmacists, which was unable to establish validity due to lack of variance. Cognitive interviews were conducted, and the measure was refined based on those findings. To ensure broader respondent type, a pilot test was conducted with 134 pharmacists within 78 care settings to establish reliability and construct validity.

**Findings:** Results from the content validity analysis supported the survey items’ relevance, clarity, and alignment for each domain measured. Interrater agreement was 0.96. A multi-level Cascade Model was conducted on the pilot data to account for the nesting. The model fit the data well (*χ*^2^ (1) = 0.10, *p* = 0.77; RMSEA = 0.00; CFI = 1.00; TLI = 1.00; SRMR = 0.002) generally indicating that the domains built on each other as designed. Cronbach’ alphas were above 0.90 for all except the last domain (0.40). The final CMM PCPFA consisted of 32 items across 5 domains (i.e., collect and analyze information, assess the information and formulate a medication therapy problem list, develop the care plan, implement the care plan, and follow up and monitor) with a 5-point scale ranging from 0 to 100% of CMM visits.

**Implications for D&I Research:** Reliability, content validity, and construct validity were found for the CMM PCPFA. The creation of this fidelity assessment is novel in pharmacy practice and provides pharmacists, pharmacy leadership, and researchers a pragmatic way to measure adherence to the CMM patient care process.

**Primary Funding Source**

American College of Clinical Pharmacy and the American College of Clinical Pharmacy Research Institute

**Reference**

1. Rubio DM, Berg-Weger M, Tebb SS, Lee ES, Rauch S (2003). “Objectifying content validity: Conducting a content validity study in social work research”. *Social Work Res*. 27: 94–104.

### S48 The care of high-need patient populations: Findings from a 2 year study of high-performing hospitals

#### John McHugh^1^, Emmanuelle Belanger^2^, David Manning^2^, Emily Gadbois^2^, Renee Shield^2^, Vincent Mor^2^

##### ^1^Columbia University, Mailman School of Public Health, New York, NY, USA; ^2^Brown University School of Public Health, Providence, RI, USA

###### **Correspondence:** John McHugh (jpm2192@cumc.columbia.edu)

**Background:** Addressing the needs of older, complex, co-morbid patients is essential to reducing costs in the U.S. healthcare system. However, the structure of our healthcare system presents barriers to finding novel approaches to care for this cohort. Pockets of excellence across the country in high-performing hospitals are evident, however, and their efforts provide meaningful lessons.

**Methods:** We utilized a mixed method, positive deviance approach to identify 92 hospitals in the U.S. that performed in the top 20% across three metrics: 90-day mortality rate, 90-day readmission rate, and percent of days in community (absent of institutional care). From the list of 92, we chose 6 hospitals based on geographic dispersion and greater than 500 high-need patients served. In visits to the hospitals between May and October 2018, we conducted 136 leadership and staff interviews at the 6 high-performing hospitals. Following the site visits and analysis of the interviews, we convened a one-day in-person meeting in June 2019 with 9 representatives from the 6 high performing sites to obtain participant validation of our research findings and brainstorm innovative solutions to the challenges of implementing change.

**Findings:** High-performing hospitals report that they create structure to support change while working within the local and regional market context. They explicitly define characteristics of high-need older patients and expand the scope of care beyond hospital boundaries. By extending their efforts into the community, these hospitals are able to leverage their expertise and programs to support their patients. Follow-up with the high-performing hospitals has revealed the dynamic nature of change within these hospitals: While alignment across provider groups remains elusive in many settings, high-performing hospitals prioritize a balance between standardizing and individualizing care strategies and solutions. The high-performing hospitals also found significant benefit to being part of a larger system; however, they report the systems do not optimize full efficiency, especially relating to innovation dissemination.

**Implications for D&I Research:** Strategies to address the care requirements of high-need older patient populations remain elusive for many hospitals. Findings from this study provide important insights to the challenges hospitals face and suggest practical solutions for the care of this high-need population.

**Primary Funding Source**

Peterson Center on Healthcare

### S49 Fidelity to individual components of a standardized labor induction protocol and association with improved obstetric outcomes: A simplification strategy prior to large-scale implementation

#### Rebecca Hamm^1^, Rinad Beidas^2^, Sindhu Srinivas^1^, Lisa Levine^1^

##### ^1^University of Pennsylvania Perelman School of Medicine, Philadelphia, PA, USA; ^2^University of Pennsylvania Leonard Davis Institute of Health Economics, Philadelphia, PA, USA

###### **Correspondence:** Rebecca Hamm (rebecca.feldmanhamm@uphs.upenn.edu)

**Background:** Standardized labor induction protocols improve obstetric outcomes. However, these protocols are complex and difficult to implement. The Consolidated Framework for Implementation Research promotes strategies to simplify execution as a part of the planning process. To best prepare our protocol for implementation success, we aimed to identify the individual induction protocol components most associated with effectiveness. The overarching goal was to simplify our induction protocol prior to multi-site implementation.

**Methods:** This is a secondary analysis of an RCT comparing time to delivery among four labor induction methods. All patients enrolled in the trial had their labor managed with a multidisciplinary-developed, evidence-based labor induction protocol. For each patient’s induction, we assessed fidelity to 7 protocol components. Primary effectiveness outcomes were cesarean delivery, maternal morbidity, and neonatal morbidity. Bivariate analyses assessed association of each fidelity measure with each primary effectiveness outcome. Multivariable logistic regression determined independent predictors of each outcome while controlling for demographic and clinical factors known to impact our outcomes.

**Findings:** The 491 patients enrolled in the randomized trial were included in this analysis. While multiple fidelity measures were associated with each outcome in bivariate analysis, only 1-2 were found to be independent predictors of effectiveness outcomes in multivariable analysis. For cesarean delivery, only (1) “if active labor was reached, all active exams were performed ≤2.5 hours apart” was an independent predictor. For maternal morbidity, (1) “if a foley catheter was utilized, it was removed by 12 hours from placement” remained significant. For neonatal morbidity (1) “all latent exams were performed ≤4.5 hours apart” and (2) “if active labor was reached, all active exams were performed ≤2.5 hours apart” remained independent predictors. Of the independent predictors of effectiveness, most reflected the overarching concept “frequent exams in labor will allow for more frequent intervention when no change is made”.

**Implications for D&I Research:** Studies determining the relationship between fidelity to individual protocol components and improved effectiveness can simplify interventions prior to large-scale implementation, a strategy with potential to increase implementation success. Specifically, these data will be used to streamline our protocol to best target cesarean rate and maternal/neonatal morbidity prior to a planned type I hybrid implementation-effectiveness trial.

**Primary Funding Source**

National Institutes of Health

### S50 Acceptability of innovative treatments for cervical pre-cancer in global settings

#### Miriam Cremer^1^, Montserrat Soler^1^, Ana C. Uriarte^2^, Raul Murillo^3^, Suhui Wu^4^, Xinfeng Qu^5^, Rachel Masch^5^, Nicole Campos^6^, Jane Kim^6^, Karla Alfaro^5^

##### ^1^Cleveland Clinic, Cleveland, OH, USA; ^2^Instituto Salvadoreño del Seguro Social, San Salvador, El Salvador; ^3^Hospital Universitario San Ignacio, Bogotá, Colombia; ^4^Shanxi Dayi Hospital, Taiyuan, Shanxi Province, China; ^5^Basic Health International, New York, NY, USA; ^6^Harvard University, Cambridge, MA, USA

###### **Correspondence:** Montserrat Soler (solerm@ccf.org)

**Background:** Cervical cancer is a leading cause of death for women in low and middle-income countries (LMICs). Secondary prevention is possible through treatment of high-grade cervical pre-cancer. In LMICs, the most common treatment for pre-cancer is gas-based cryotherapy, which poses significant accessibility challenges due to the ongoing expense of gas and the difficulty of storing and transporting heavy gas cylinders. A randomized trial is currently underway to compare the efficacy of gas-based cryotherapy with two alternatives designed for use in LMICs: CryoPen® (non-gas cryotherapy) and thermal ablation with a handheld device. Patient acceptability of these new methods is assessed in the context of this trial, and cost-effectiveness analyses will be carried out across sites.

**Methods:** Data in El Salvador, Colombia, and China have been collected from 528 women randomized to three treatment arms: CO_2_-gas cryotherapy, CryoPen®, and thermal ablation. Acceptability assessments included pain before, during, and after treatment, side-effects, and satisfaction with treatment at a follow-up visit. Percentages and chi square tests were used to describe and compare findings across the study treatments.

**Findings:** There were no differences in pain levels before and after treatment, but women who underwent thermal ablation reported more pain during treatment than those in other treatment arms (cryotherapy = 2.31±2.57, CryoPen 2.97± 2.70, and thermoablation = 3.41±2.8, *p*= .004). Pain during treatment also varied significantly across sites (China = 1.56±1.99, Colombia = 2.65±2.64, El Salvador = 4.38±2.65, *p*<.001). At a 6-week follow-up visit, most participants (96%) reported experiencing at least one side effect, the most common being fluid discharge (94.4%). Most women (89%) reported they would recommend the treatment they received to a friend, citing as reasons that it was “non-surgery”, “not complicated”, “quick”, and “not very uncomfortable”. To date, 90% of women report being very satisfied with the treatment, 7% somewhat satisfied, and 3% neutral.

**Implications for D&I Research:** Innovative treatments are urgently needed in global healthcare. However, to meaningfully reduce the burden of disease, development of new technologies must be accompanied with multi-level contextual assessments. One strategy is to include implementation research in the design of efficacy trials. The present study offers an example of this approach.

**Primary Funding Source**

National Institutes of Health

### S51 Implementation of stride, an office-based intervention to prevent falls and fall-related injuries

#### Jennifer Reckrey^1^, Albert Siu^1^, David Reuben^2^, Nancy Latham^3^, Siobhan McMahon^4^, Priscilla Gazarian^3^, Fred Ko^1,5^

##### ^1^Icahn School of Medicine at Mount Sinai, New York, NY, USA; ^2^University of California, Los Angeles, Los Angeles, CA, USA; ^3^Brigham and Women’s Hospital, Boston, MA, USA; ^4^University of Minnesota, Minneapolis, MN, USA; ^5^James J. Peters VA Medical Center, Bronx, NY, USA

###### **Correspondence:** Jennifer Reckrey (jennifer.reckrey@mountsinai.org)

**Background:** Despite the public health burden of fall-related injuries, evidence-based fall-prevention programs are rarely effectively integrated into primary care. The national STRIDE study, a multisite cluster randomized pragmatic trial funding by the NIA and PCORI, aimed to reduce serious fall-related injuries among non-institutionalized older adults by embedding a falls care manager (FCM) within trial practices to create individualized fall-reduction care plans for high risk older adults. To better understand how fall prevention interventions might be implemented in primary care, we conducted this implementation study to accompany the effectiveness trial to form a hybrid type 1 effectiveness-implementation study.

**Methods:** We developed and piloted an interview guide based on the Consolidated Framework for Implementation Research (CFIR) and conducted telephone semi-structured interviews with key personnel involved with the STRIDE intervention: FCMs (n=13), groups of 2-3 study staff from each site (n=10), a group of 3 informants from the National Patient Stakeholder Council (n=1), and a group of 3 informants from the STRIDE Central Project Management (n=1). A codebook based on the CFIR was developed and iteratively refined using both inductive and deductive approaches. Code were applied to FCM interviews and the research team reviewed all coded data to determine themes. The remaining interviews will be coded and analyzed by November 2019.

**Findings:** Analysis identified themes related three major CFIR constructs that impacted STRIDE study implementation. Themes related to “Intervention Characteristics” included variable partnerships with providers, financial burden of intervention, and particularly challenging portions of the intervention protocol. Themes related to “Characteristics of Individuals” included individual readiness of change, declining patient health, and challenges of transportation. Themes related to “Inner Setting” included practice climate, limited integration of STRIDE workflow into the practice, and variability in available practice resources.

**Implications for D&I Research:** Successful clinical interventions to support the health of the rapidly growing population of older adults with complex health needs often entail highly comprehensive assessments and complex, individualized interventions. This may limit implementation and effectiveness. Our findings indicate several important characteristics of the intervention, individuals, and practice setting that may pose barriers to implementation. Implementation strategies and adaptations may be considered to address these determinants.

**Primary Funding Source**

National Institutes of Health

### S52 Patient navigators and their activities in the colorectal cancer control program (CRCCP): A national survey

#### Wendy Barrington^1^, Thuy Vu^1^, Amy DeGroff^2^, Stephanie Melillo^2^, Allison Cole^1^, Cam Escoffery^3^, Natoshia Askelson^4^, Laura Seegmiller^4^, Sarah Koopman Gonzalez^5^, Peggy Hannon^1^

##### ^1^University of Washington, Seattle, WA, USA; ^2^Centers for Disease Control and Prevention, Atlanta, GA, USA; ^3^Rollins School of Public Health, Emory University, Atlanta, GA, USA; ^4^University of Iowa, Iowa City, IA, USA; ^5^Case Western Reserve University, Cleveland, OH, USA

###### **Correspondence:** Wendy Barrington (wendybar@uw.edu)

**Background:** Patient navigation is a promising approach to decrease disparities in cancer screening. The Colorectal Cancer Control Program (CRCCP) is a federally-funded program that reached over 600 clinics serving over 1 million medically underserved patients, ages 50-75, from 2015-2018. Scant data directly from patient navigators (PNs) exist describing their environments and how they help patients complete colorectal cancer (CRC) screening. We assessed PN characteristics and their activities in the CRCCP to inform optimal PN training and support going forward.

**Methods:** We conducted a cross-sectional online survey among PNs affiliated with CRCCP-funded awardees to assess their demographic characteristics, professional training, navigation settings, patient barriers, and activities delivered. CRCCP awardees included state, university, and tribal entities; all (30 of 30) provided valid email contact information for their PNs (N=847). A total of 236 (27.9%) PNs completed the survey.

**Findings:** One hundred and seventy-two respondents (72.3%) reported they currently navigate for CRC screening. Most CRC PNs were female (93%), high-school educated (90%), and helped patients manage conditions in addition to cancer (83%). Many PNs were non-White (47%), relatively new to navigation (54% <5 years in role), not clinically trained (36%), and employed by government agencies (46%). Patient barriers reported by ≥75% PNs included: lack of knowledge about cancer, cancer screening procedures, and the benefit of screening; lack of motivation to get screened; transportation challenges; and lack of health insurance. Navigation activities reported by ≥75% of PNs included: talking to patients in clinics about screening; providing one-on-one education; and assessing patient barriers to screening. Clinically-trained PNs were more likely to help patients understand the bowel prep process (68.8%) and access associated materials (56.3%) compared to PNs without clinical training (40.3% and 29.0%, respectively; *P* value<0.001 for all).

**Implications for D&I Research:** This study is the first to examine PN-reported navigation environments and activities delivered within the CRCCP. PN activities focused on patient education and barrier assessment. PNs can play key roles in several evidence-based strategies to increase CRC screening, including patient reminders and reducing structural barriers; our findings suggest these are potential areas for additional PN training and support in the CRCCP.

**Primary Funding Source**

Centers for Disease Control and Prevention

### S53 Implementing and adapting a promising patient navigation intervention to increase colonsocopy completion

#### Allison Cole^1^, Thuy Vu^1^, Marlana Kohn^1^, Gloria Coronado^2^, Amy DeGroff^3^, Dara Schlueter^3^, Peggy Hannon^1^

##### ^1^University of Washington, Seattle, WA, USA; ^2^Kaiser Permanente Center For Health Research, Portland, OR, USA; ^3^Centers for Disease Control and Prevention, Atlanta, GA, USA

###### **Correspondence:** Allison Cole (acole2@uw.edu)

**Background:** Colorectal cancer (CRC) is a leading cause of cancer death in the United States. The U.S. Preventive Services Task Force recommends a variety of colorectal cancer screening tests, including colonoscopy. Patients experience several barriers to completing colonoscopy, and low rates of colonoscopy completion contribute to worse CRC health outcomes, particularly in populations at-risk for health disparities. A promising patient navigation program increased colonoscopy completion rates among patients served by the Centers for Disease Control and Prevention’s Colorectal Cancer Control Program (CRCCP) in New Hampshire. The purpose of the present study was to evaluate implementation and effectiveness of an adapted version of this patient navigation program in a new setting.

**Methods:** We partnered with a CRCCP grantee implementing a state-wide cancer screening program providing no-cost colonoscopy to low- income and un/under-insured clients to implement and evaluate the patient navigation program. Core elements of the adapted program include a six-topic protocol delivered telephonically by a registered nurse patient navigator (PN) to patients before and after colonoscopy, and an electronic system to collect and monitor participant data. We provided training and implementation technical assistance (TA), assessed the degree of implementation, and evaluated effectiveness using a randomized controlled trial. TA topics highlighted implementation adaptations and challenges.

**Findings:** The CRCCP grantee randomized 402 participants to intervention and screened them for eligibility; 347 participants were randomized to control. The PN was unable to contact 54% of intervention participants. Of those reached, 45% were eligible, and, of those, 97% enrolled in the intervention. Among enrolled participants, 74% were Hispanic/Latino and 46% required an interpreter. Fifty-four (75%) enrolled participants completed colonoscopy, compared to only 40 (12%) participants in the control group.

**Implications for D&I Research:** The majority of patients enrolled in navigation completed colonoscopy; however, implementation challenges highlight opportunities for dissemination and implementation research to optimize navigation. Program adaptations, including use of a non-nurse navigator, impact feasibility and could reduce program implementation costs. Difficulty reaching participants by phone may diminish the potential public health impact of this intervention with some populations. Future research that examines offering navigation to risk-stratified populations and alternative contact methods may enhance navigation reach and effectiveness, and facilitate program sustainability.

**Primary Funding Source**

Centers for Disease Control and Prevention

### S54 Patient randomized trial of a targeted navigation program to improve rates of colonoscopy after positive FIT in community health centers

#### Gloria Coronado^1^, Eric Johnson^2^, Michael Leo^1^, Jennifer Schneider^1^, David Smith^1^, Amanda Petrik^1^, Jamie Thompson^1^, Ricardo Jimenez^3^

##### ^1^Kaiser Permanente Center For Health Research, Portland, OR, USA; ^2^Northwest Permanente, Portland, OR, USA; ^3^SeaMar Community Health Centers, Seattle, WA, USA

###### **Correspondence:** Amanda Petrik (Amanda.F.Petrik@kpchr.org)

**Background:** Colorectal cancer screening by annual fecal immunochemical test (FIT) is an accessible and cost-effective strategy to lower colorectal cancer incidence and mortality. However, this mode of screening depends on follow-up colonoscopy after an abnormal FIT result to prevent cancer or find it in early, treatable forms. Unfortunately, nearly one-half of FIT-positive patients fail to complete this essential screening component, negating any benefit of FIT screening. Health-system-level interventions, such as patient navigation, may improve follow-up colonoscopy adherence, but such interventions are costly. To deliver patient navigation cost-effectively, systems could target navigation to patients who are unlikely to complete the procedure on their own.

**Methods:** Two studies contribute to this work. First, our team developed a colonoscopy adherence risk prediction model, using demographic and health care utilization variables commonly found in the electronic health record of Kaiser Permanente Northwest (KP NW) patients. The **Evaluating the Adoption and Implementation of an Evidence-Based Patient Navigation Intervention for Colonoscopy Screening (CDC SIP 16-001)** study piloted the use of the risk prediction model at KPNW to prioritize FIT-positive patients who needed navigation and tested the effectiveness of navigation for follow-up colonoscopy completion. The ongoing **Predicting and Addressing Colonoscopy in Safety Net Settings (PRECISE; NIH R01CA218923)** patient-randomized trial will validate the risk prediction model in a large and diverse community health center. PRECISE will determine whether patient navigation raises rates of colonoscopy adherence overall and among patients in each probability stratum (low, moderate, and high probability of adherence without intervention). We will also report cost-effectiveness of the program.

**Findings:** A total of 458 KP NW patients were randomized to receive patient navigation or usual care. Among patients in the lower probability groups (<65%), patient navigation increased colonoscopy completion by 9 percentage points and decreased days to colonoscopy by more than two weeks. The re-development of the risk prediction model for a large, diverse community health center will be discussed.

**Implications for D&I Research:** These clinical trials showcase an innovative and data-informed way to select patients for patient navigation, with the overarching goal of lowering implementation costs and promoting maintenance and broad adoption of patient navigation programs.

**Primary Funding Source**

National Institutes of Health

### S55 Message testing recruitment videos for a smoking cessation trial

#### Jordan Neil^1^, Nancy Rigotti^1^, Jennifer Haas^2^, Saif Hawari^1^, Caylin Marotta^1^, Efren Flores^2^, Elyse Park^1^

##### ^1^Massachusetts General Hospital/Harvard Medical School, Boston, MA, USA; ^2^Massachusetts General Hospital, Boston, MA, USA

###### **Correspondence:** Jordan Neil (jmneil@mgh.harvard.edu)

**Background:** Digital recruitment messages offer promise to improve the reach, success, and personalization of recruitment efforts for clinical trials. Screen ASSIST, a smoking cessation trial that offers cessation resources to smokers undergoing lung cancer screening (LCS), utilizes a novel video platform to create and send brief recruitment videos to eligible smokers. However, little empirical research has identified which video message components are most relevant and effect intent to participate in clinical trials.

**Methods:** In January 2019, study recruitment messages were tested in an online experiment of current smokers eligible for LCS (*N*=281). Participants were randomized to a 2x2 factorial design to test two recruitment message components:1) risks of continued smoking vs. benefits of quitting;2) cost of not participating vs. benefits of participating in the study. The aims of the study were to:1) test successful dissemination of the recruitment messages;2) determine which recruitment message components yielded greater perceived relevance and/or intent to participate in a trial;3) explore the relationship between message relevance and intent to participate. Message relevance was measured on a 2-item, 5-point scale (*M*=3.70,*SD*=1.05) and intent to participate was measured on a 5-item, 7-point scale (*M*=4.25,*SD*=1.70).

**Findings:** In total, 82% of participants (n=230) watched their entire recruitment message (*Mdn*=3:01mins). Participants randomized to the risks of continued smoking factor reported greater message relevance (*M*=3.71,*SD*=1.00,*p*=.88) but lower intent to participate (*M*=4.11,*SD*=1.75 *p*=.22). Participants randomized to the cost of not participating factor reported lower message relevance (*M*=3.67,*SD*=1.09,*p*=.56) but greater intent to participate (*M*=4.29,*SD*=1.76*,p*=.63). Overall, participants who watched a message containing the benefits of cessation and cost of not participating reported the greatest intent to participate in a cessation trial(*M*=4.41,*SD*=1.66,*p*=.63). Controlling for message condition and participant sociodemographic and smoking factors, greater perceptions of message relevance significantly predicted greater intention to participate in a cessation trial (*b*=.44,*p*<.001).

**Implications for D&I Research:** Digital recruitment strategies using a brief video have the potential to be low-burden, high-yield methods for improving study recruitment to clinical trials. Findings from this study determined the optimal digital recruitment message for Screen ASSIST, and offer insight on how to develop, rigorously evaluate, and disseminate recruitment messages for future smoking cessation interventions.

**Primary Funding Source**

National Institutes of Health

### S56 Monitoring acceptability of and engagement with SMART, an ehealth stepped-care HIV prevention intervention for adolescent men who have sex with men

#### Dennis Li^1,2^, Rana Saber^1^, David Moskowitz^1,2^, Brian Mustanski^1,2^

##### ^1^Institute for Sexual and Gender Minority Health and Wellbeing, Northwestern University, Chicago, IL, USA; ^2^Northwestern University Feinberg School of Medicine, Chicago, IL, USA

###### **Correspondence:** Dennis Li (dennis@northwestern.edu)

**Background:** One of the benefits of eHealth interventions is the ability to continuously monitor acceptability of the intervention by end users. Given the rapid pace of sociotechnical evolution—i.e., changes in individuals’ perceptions and use of technology as well as the technology itself—this feature is critical for implementation and sustainment. However, there are few examples of how this feature has been deployed in research trials. This presentation describes our ongoing tracking of acceptability and engagement in SMART, an online, stepped-care HIV prevention program for adolescent men who have sex with men (AMSM) aged 13–18, for whom no tailored evidence-based HIV prevention programs currently exist despite a substantial health disparity.

**Methods:** The SMART Program comprises a suite of three self-paced, multimedia health education interventions (SMART Sex Ed [SSE], SSE 2.0, SMART Squad) and one videoconferencing motivational interviewing protocol (SMART Sessions), currently being tested nationally in a year-long, sequential, multiple-assignment, randomized trial. Recruitment is ongoing, with 749 enrolled as of July 15, 2019.

We assessed acceptability and engagement via three mixed-methods sources: (a) the Intervention Acceptability and Tolerance (IAT) scale administered after each component and at the 12-month follow-up, (b) user rating and feedback pages throughout the intervention application, and (c) user meta-data (e.g., page clicks, device).

**Findings:** IAT scores for each component are consistently high (>90% agree or strongly agree) across all constructs (e.g., helpful, relevant, appropriate, convenient) except for *fun* in SSE (88.2%) and *convenience* in Sessions (79.3%). Similarly, 97.2% of the nearly 7,800 in-application ratings were positive on a thumbs-up/down scale. AMSM who completed SSE and Squad spent on average 107 and 110 minutes in the respective interventions. Most AMSM accessed SMART at night, and iOS was the most popular operating system (45.8%). Qualitative responses from the IATs and the SSE/Squad feedback pages will be presented. Results will be refreshed with data collected through November 2019.

**Implications for D&I Research:** eHealth interventions offer a plethora of data that can be used to monitor intervention engagement and acceptability over time to inform implementation, ongoing adaptation, and sustainment. Future directions include developing new methods to process and use these data in a timely manner.

**Primary Funding Source**

National Institutes of Health

### S57 Implementing a multi-level intervention to improve tobacco use treatment for cancer patients: A mixed-methods study

#### Ryan Theis^1^, Jennifer LeLaurin^1^, Deandra Chetram^2^, Jesse Dallery^2^, Natalie Silver^1^, Merry-Jennifer Markham^1^, Stephanie Staras^1^, Matthew Gurka^1^, Ramzi Salloum^1^

##### ^1^University of Florida College of Medicine, Gainesville, FL, USA; ^2^University of Florida, Gainesville, FL, USA

###### **Correspondence:** Ryan Theis (rtheis@ufl.edu)

**Background:** Cancer survivors who use tobacco face increased risk of poor treatment outcomes, additional primary cancers, and mortality. While practice guidelines recommend routine screening and referral to tobacco use treatment (TUT), competing demands of cancer care pose challenges in implementing these practices. To address this gap, we evaluated the feasibility of a multi-level intervention that incorporates: (1) mhealth-based cognitive behavioral therapy (mCBT), telephone counseling, or in-person group counseling options for patients; (2) an electronic health record (EHR)-based TUT referral system; and (3) strategies to connect clinics with a state-funded TUT program.

**Methods:** We used mixed-methods to understand acceptability and feasibility of the intervention to patients and providers and to inform tailored changes to the intervention. Focus groups with providers and staff in medical and surgical oncology clinics were conducted prior to implementation (n = 14). Qualitative interviews with 13 physicians specializing in medical, radiation, or surgical oncology were conducted after implementation of the EHR-based referral system. Structured surveys with patients were conducted at baseline (n = 87) and at 12-week follow-up (n = 22).

**Findings:** Focus groups revealed concerns about low capacity for screening patients for tobacco use and limited space in clinics for in-person counseling. In qualitative interviews, physicians considered the EHR-based referral system to be burdensome, and expressed a preference for sending lists of patients who use tobacco to the state-funded TUT program, which would not interrupt clinical workflow. Physicians indicated that timing of in-person counseling depended on the patient’s stage of recovery, as patients recently diagnosed with cancer may be less receptive to counseling. Preliminary data on patient acceptability of treatment options complement these findings. The majority of patients (80%) opted for either mCBT or telephone counseling, which most of these patients (>60%) chose because the intervention modality was convenient and could be done from home.

**Implications for D&I Research:** This study demonstrates the acceptability and feasibility of an evidence-based, multi-level TUT intervention from the perspective of both patients and providers, and points toward refinements to facilitate its implementation in cancer care settings. Further data collected in this ongoing study will reveal opportunities to address barriers related to clinic space, workflow, and patient-level factors.

**Primary Funding Source**

University of Florida Health Cancer Center

### S58 Effectiveness of participatory community solutions strategy on improving household and provider health care behaviors and practices: A mixed method evaluation

#### Gizachew Tiruneh^1^, Nebreed Zemichael^1^, Wuleta Betemariam^2^, Ali Karim^3^

##### ^1^JSI Research and Training Institute Inc., Addis Ababa, Ethiopia; ^2^John Snow, Inc, Addis Ababa, Ethiopia; ^3^NGO, Addis Ababa, Ethiopia

###### **Correspondence:** Gizachew Tiruneh (gizachew_tadele@et.jsi.com)

**Background:** We implemented a participatory quality improvement strategy in eight primary health care units of Ethiopia to improve utilization and quality of maternal and newborn health services.

**Methods:** We evaluated the effects of this strategy using a mixed methods research. The changes in maternal and newborn health (MNH) care indicators between 39 communities that received the intervention and 148 communities that did not receive the intervention were compared using before-and-after cross-sectional surveys of women with children 0 to 11 months of the 187 kebeles in March 2016 and November 2017.Propensity scores were used to match the intervention with the comparison communities at baseline and difference-in-difference analyses were used to estimate intervention effects. The qualitative method included 51 in-depth interviews of community volunteers, health extension workers, health center director, health center staff and project specialists.

**Findings:** The difference-in-difference analyses indicated that 7.6 %-points (95% confidence interval [CI]: -0.0-15.3 %) increase of receiving early antenatal care between baseline and follow-up surveys in the intervention area is attributable to the strategy. Similarly, the intervention effect on skilled delivery and postnatal care in 48 hours of the mother, were respectively, 7.9 % (95% CI: 1.8-13.9 %), 15.3 % (95% CI: 7.4-23.2). However, there was no evidence that the strategy affected the seven other maternal and newborn health care indicators considered. Interview participants expressed that the participatory design and implementation strategy helped them to realize gaps, identify real problems, and design appropriate solutions, as well as create ownership and shared responsibilities to implement innovations.

**Implications for D&I Research:** Community participation in planning and monitoring maternal and newborn health service delivery is effective in improving utilization of some of the high impact maternal and newborn health services. The study supports the notion that participatory community strategies should be considered to foster community-responsive health systems.

**Primary Funding Source**

BMGF

## Clinical Care Settings: System-level Interventions

### S59 Adaptation and fidelity of serious illness care program across primary care settings in the US and Canada

#### Seiko Izumi^1^, Danielle Caron^2^, Angela Combe^3^, Patrick Archambault^2^, Nicholas Shankle^1^, LeAnn Michaels^1^, David Dorr^1^, Annette Totten^1^

##### ^1^Oregon Health & Science University, Portland, OR, USA; ^2^Université Laval, Quebec City, QC, Canada; ^3^Oregon Health & Science University, La Grand, OR, USA

###### **Correspondence:** Seiko Izumi (izumis@ohsu.edu)

**Background:** The Serious Illness Care Program (SICP) intends to encourage Advance Care Planning with patients who are living with serious illness. Although evidence shows effectiveness of SICP, little is known how to implement SICP in primary care settings. As part of PCORI funded trial comparing effectiveness of two different approaches for SICP, we examine the implementation process of SICP in 42 primary care settings in the US and Canada. We will present the challenges, strategies, and outcomes of SICP implementation in multiple primary care settings.

**Methods:** We planned a type 2 hybrid design blending clinical effectiveness trial and implementation research.^1^ As the interventions of the trial, we have randomly assigned and implemented clinician-focused versus team-focused SICP in equal arms of 21 clinics. Study facilitators assigned to a group of clinics work closely with key stakeholders (e.g., clinicians, team members, staffs, administrators) providing SICP training and integrating the program into their practice, and collect information regarding implementation process. Investigators interviewed the facilitators about the implementation experience and process of adapting SICP. Qualitative interview data are analyzed using the adaptation framework.^2^

**Findings:** The trial began December 2017 and is ongoing. A major challenge has been balancing adaptation and fidelity of the intervention. To allow modification to suit the local context of each setting while ensuring fidelity, we provided a list of key aspects of the intervention that can and cannot be modified. Facilitators found that there were not many modifications in SICP training, and great variation in implementation process by clinics: e.g., team members to be involved, decision making process, and how to adapt SICP into workflow. The majority of variations were due to the clinic structure, team membership, clinic culture, and population served.

**Implications for D&I Research:** Interventions that involve interprofessional team members add complexity and require coordinated tailoring of the intervention, yet may contribute to more engagement and sustainability depending on the culture of the clinic. Our strategies and lessons learned from this study could inform implementation of similar interventions across a broad range of clinical settings.

**Primary Funding Source**

Patient-Centered Outcomes Research Institute

### S60 Longitudinal assessment of expert recommendations for implementing change (ERIC) strategies in the uptake of evidence-based practices for hepatitis c treatment in the veterans administration: Year four

#### Vera Yakovchenko^1^, Matthew Chinman^2^, Rachel Gonzalez^3^, Angela Park^4^, Timothy Morgan^5^, Maggie Chartier^6^, David Ross^6^, Shari Rogal^7^

##### ^1^Center for Healthcare Organization and Implementation Research, VA, BridgeQUERI & CHOIR, Veterans Health Administration, Bedford, MA, USA; ^2^Veterans Integrated Service Network 4 Mental Illness Research, Education and Clinical Center, VA Pittsburgh Healthcare System, Veterans Health Administration, Pittsburgh, PA, USA; ^3^Department of Research, VA Long Beach Healthcare System, Long Beach, CA, USA; ^4^Boston VA Healthcare System, New England Veterans Engineering Resource Center, Veterans Health Administration, Boston, MA, USA; ^5^Department of Gastroenterology, VA Long Beach Healthcare System, Veterans Health Administration, Long Beach, CA, USA; ^6^Office of Patient Care Services; HIV, Hepatitis, and Public Health Pathogens Programs, Veterans Healthcare Administration, Washington, DC, USA; ^7^Division of Gastroenterology, Hepatology, and Nutrition, University of Pittsburgh School of Medicine, Pittsburgh, PA, USA

###### **Correspondence:** Vera Yakovchenko (vera.yakovchenko@va.gov)

**Background:** The Department of Veterans Affairs (VA) established a national collaborative (HIT) composed of regional teams of interdisciplinary and interspecialty providers, leaders, and staff tasked with supporting local implementation strategies to increase curative treatments for hepatitis C (HCV). The primary aim of this longitudinal evaluation was to assess how site-level implementation strategies were associated with HCV treatment initiation over time.

**Methods:** HIT representatives from each VA site (N=130) were asked over four consecutive fiscal years (FYs) to complete a survey examining the use of 73 implementation strategies organized into 9 clusters as described by the Expert Recommendations for Implementing Change study. The number of Veterans initiating HCV treatment at each site was captured using administrative data. Descriptive, nonparametric, and multivariate analyses were conducted on the respondents in FY15 (N=80), FY16 (N=105), FY17 (N=109), and FY18 (N=88).

**Findings:** Of 130 sites, 127 (98%) responded at least once and 54 (42%) responded all 4 years. The mean number of strategies endorsed in each year were FY15: 25±14, FY16: 28±14, FY17: 26±15, and FY18: 35±26. The most commonly endorsed strategies across all years were: data warehousing techniques, tailoring strategies to deliver HCV care, and intervening with patients to promote uptake and adherence to HCV treatment. In FY15, the strategies associated with treatment starts (“successful strategies”) focused predominantly on developing interrelationships between stakeholders. In subsequent years, successful sites utilized different strategies appropriate to the project lifecycle: evaluative and iterative strategies in FY16, training and educating stakeholders in FY17, and providing interactive assistance in FY18. One strategy (“identify early adopters to learn from their experiences”) was significantly associated with HCV initiation treatment in each year.

**Implications for D&I Research:** We found that while the number of implementation strategies remained relatively stable over time, successful sites focused on different implementation methods over time. These results add to our understanding of changes in implementation strategies over time and across stages of planning, implementation, and sustainability in a national HCV quality improvement initiative. Understanding the shifts in the strategies associated with successful treatment initiation over time can provide important information for similar programs.

**Primary Funding Source**

Department of Veterans Affairs

### S61 Increasing diabetic eye screening rates by adapting the niatx model for stakeholder-engaged teleophthalmology implementation in primary care

#### Yao Liu^1^, Alejandra Torres Diaz^1^, Julia Carlson^1^, Nicholas Zupan^1^, Todd Molfenter^1^, Kyle McDaniel^1^, Jane Mahoney^1^, Mari Palta^1^, Deanne Boss^1^, Ronald Klein^1^, Timothy Bjelland^2^, Maureen Smith^1^

##### ^1^University of Wisconsin-Madison, Madison, WI, USA; ^2^Mile Bluff Medical Center, Mauston, WI, USA

###### **Correspondence:** Yao Liu (yaoliumd@gmail.com)

**Background:** Teleophthalmology is a greatly underutilized, evidence-based method of diabetic eye screening recommended by the American Diabetes Association. Diabetic eye disease is the leading cause of blindness among working-age U.S. adults. Teleophthalmology has tremendous potential to prevent avoidable blindness, but has been challenging to successfully implement in U.S. primary care clinics. We tested the hypothesis that the NIATx Model, a systematic behavioral healthcare process improvement framework, could be adapted to increase teleophthalmology use and diabetic eye screening rates in a rural primary care clinic.

**Methods:** Patients and clinical stakeholders (i.e., primary care providers, patient care staff, and administrators) were recruited in March 2017 from the Mile Bluff Medical Center, a rural U.S. health system where a teleophthalmology program was established in 2015 for all primary care clinics, but the program was very underutilized. We adapted the NIATx Model to guide stakeholder meetings and used PDSA (plan-do-study-act) cycles to test strategies for increasing teleophthalmology use at one (Mauston) of five Mile Bluff primary care clinics. Our approach aimed to tailor implementation strategies, developed and selected by our stakeholders, to an individual clinic’s needs and resources. The primary outcome measures were teleophthalmology use and diabetic eye screening rates, which were assessed with a patient phone survey and medical chart review. Differences in screening rates were tested using chi-square tests.

**Findings:** Nine patients and 23 clinical stakeholders participated in 22 meetings from May 2017-October 2018. Strategies tested at the Mauston clinic included a patient rooming checklist, quarterly provider performance reports, provider performance-based financial incentives, and patient reminder calls. Teleophthalmology use increased 5-fold at the Mauston clinic compared to 0.4-fold at the other clinics (p = 0.03). Overall diabetic eye screening rates at Mile Bluff increased from a baseline of 47.4% in 2015 to 64.0% in 2018. We created an implementation toolkit to facilitate dissemination of our teleophthalmology implementation package to other U.S. health systems.

**Implications for D&I Research:** We adapted the NIATx Model to provide stakeholder-engaged, tailored implementation of a complex intervention to increase teleophthalmology use in primary care. Our freely-available online toolkit may serve as a guide for implementing other complex preventive care interventions in primary care settings.

**Primary Funding Source**

National Institutes of Health

### S62 Implementation outcomes of humanwide: A pilot project of integrated precision health in team-based primary care

#### Cati Brown-Johnson^1^, Nadia Safaeinili^1^, Juliana Baratta^1^, Sarah Morris^1^, Latha Palaniappan^1^, Megan Mahoney^2^, Lisa Rosas^1^, Marcy Winget^1^

##### ^1^Stanford University, Stanford, CA, USA; ^2^Stanford Health Care, Stanford, CA, USA

###### **Correspondence:** Cati Brown-Johnson (catibj@stanford.edu)

**Background:** A learning healthcare system approach that focuses on pragmatic implementation evaluation of early pilots has been recommended for precision medicine to support implementation, future adaptation and spread.

**Methods:** The Humanwide precision health initiative in primary care included: 1) health coaching; 2) four digital health tools for blood-pressure, weight, glucose, and activity; 3) pharmacogenomic testing; and 4) genetic screening/testing. We conducted semi-structured interviews with a sample of patients, providers, and staff; and chart reviews for all 50 participating patients. We analyzed interview transcripts and observational meeting notes using a consensus approach, relying on multiple coders and input from research team and stakeholders. Emergent themes were mapped to implementation science constructs *penetration/reach*, *acceptability*, *feasibility*, *sustainability*.

**Findings:** 42% of participating patients received all four components of Humanwide, representing overall reach/penetration. Reach/penetration for individual components ranged from 94% (pharmacogenomics) to 64% (digital health). Patients and providers reported Humanwide was acceptable and engaged them holistically, supported faster medication titration, and strengthened patient-provider relationships. All patients clinically benefitted from participation in Humanwide. Challenges to feasibility included: low provider self-efficacy for supporting genetics and pharmacogenomics; difficulties with data integration; patient technology challenges; and lack of staffing support. Additional concerns around financial burden for patients surfaced with respect to sustainability.

**Implications for D&I Research:** Precision health embedded in primary care can be a success; patients are both enthusiastic and receive high quality care. Barriers to implementation are straightforward, though not easily overcome. To facilitate implementation of precision health in primary care, we recommend health systems invest in training and solutions that embed smart EHR solutions in well-mapped workflows and pay a small early tax by staffing and integrating additional lower-wage specialists/staff (e.g., medical assistants) in team-based care. We contend that successful integration into primary care also hinges on using the learnings from implementation science to inform integration of cutting-edge technology into healthcare workflows. This will support systems in addressing the responsibility of meeting our patients at the crossroads of innovative science and existing clinical systems, while also providing the resources needed for primary care teams to practice medicine of the future.

**Primary Funding Source**

Stanford Health Care

### S63 Economic costs of implementing virtual care for patients with post-traumatic stress disorder

#### Edwin Wong^1,2^, Suparna Rajan^3^, Chuan-Fen Liu^4^, John Fortney^1,3^

##### ^1^University of Washington, Seattle, WA, USA; ^2^Center of Innovation for Veteran-Centered and Value-Driven Care, VA Puget Sound Health Care System, Veterans Health Administration, Seattle, WA, USA; ^3^VA Puget Sound Healthcare System, Veterans Health Administration,, Seattle, WA, USA; ^4^Department of Health Services, University of Washington, Seattle, WA, USA

###### **Correspondence:** Edwin Wong (eswong@uw.edu)

**Background:** Rural veterans with post-traumatic stress disorder (PTSD) comprise 9.2% of all patients enrolled in the Veterans Affairs Health Care System (VA). Prior research indicates these patients experience little to no improvement in symptoms over time. Trauma-focused psychotherapy is the first-line treatment for patients with PTSD, primarily provided at larger urban VA Medical Centers. However, travel barriers including lengthy driving times limit rural patients’ access to these services. Telemedicine Outreach for PTSD (TOP) is a virtual evidence-based practice involving telephone care management and telepsychology that engages rural patients’ in trauma-focused psychotherapy. This study examined the costs of implementing TOP from a health system perspective.

**Methods:** Implementation costs were ascertained as part of a stepped wedge cluster randomized trial. All five sites initially received a **standard implementation** strategy, which included internal facilitation, funded care managers, dissemination of an operations guide, training materials and technical support. A subset of clinics subsequently received **enhanced implementation**, which included external facilitation and the incorporation of clinical processes of TOP into the existing clinic workflow. We measured clinic-level costs for each implementation strategy using project records and structured activity logs tracking personnel-level time devoted to implementation activities. Activities were tracked by site principal investigators, care managers, and external facilitators. We monetized time devoted to implementation activities by applying an opportunity cost approach using wage data from VA administrative databases and the Bureau of Labor Statistics. We conducted descriptive analyses of strategy-specific implementation costs across the five sites.

**Findings:** Over the 40-month study period, four of five sites received enhanced implementation (two after month 11 and two after month 22). Across all clinics, the mean clinic-level cost of standard implementation was $817 (SD=$493) per month. For the four clinics receiving enhanced implementation, the mean clinic-level cost was $2,647 (SD=$1,493) per month.

**Implications for D&I Research:** This study provides the first set of estimates informing the costs of implementing an evidence-based practice for PTSD into routine clinical care. Health systems considering broader use of virtual care for PTSD should account for implementation costs as well as clinical costs.

**Primary Funding Source**

Department of Veterans Affairs

### S64 Costs and effectiveness of interventions implemented to increase colorectal cancer screening in eight cdc colorectal cancer control program awardees

#### Florence Tangka^1^, Sujha Subramanian^2^, Sonja Hoover^3^, Christen Lara^4^, Casey Eastman^5^, Becky Glaze^6^, Mary Ellen Conn^7^, Melissa Barajas^8^

##### ^1^Centers for Disease Control and Prevention, Atlanta, GA, USA; ^2^RTI International, Waltham, MA, USA; ^3^Social Policy, Health and Economics Research, RTI International, North Waltham, MA, USA; ^4^Colorado Department of Public Health & Environment, Denver, CO, USA; ^5^Office of Health Communities, Washington State Department of Health, Olympia, WA, USA; ^6^HealthPoint, Renton, WA, USA; ^7^WVU Cancer Institute, West Virginia University, Morgantown, WV, USA; ^8^Neighborhood Healthcare, Escondido, CA, USA

###### **Correspondence:** Florence Tangka (ftangka@cdc.gov)

**Background:** The Colorectal Cancer Control Program (CRCCP) is a national program funded by the Centers for Disease Control & Prevention (CDC) to promote and increase colorectal cancer (CRC) screening uptake among individuals aged 50-75. Thirty awardees have been funded, and they consist of state health departments, academic medical centers, and tribal organizations. A key objective of CRCCP is supporting health systems to implement patient and provider evidence-based interventions (EBIs). Awardees typically implemented at least one EBI recommended by The Community Guide with the option of implementing supporting activities as well. The purpose of this analysis is to examine the costs and the effectiveness of the interventions implemented.

**Methods:** The Colorado Department of Public Health & Environment (CO); Washington State Department of Health (WASDOH); West Virginia University; and the California Department of Public Health provided cost and effectiveness data for the analysis. We report results from 8 federally qualified health centers (FQHCs). Tools for data collection were designed specifically for each awardee, as each implemented different combinations of EBIs and supporting activities. Data on staff, salaries, and time spent conducting various activities specific to the intervention and nonlabor costs (e.g., software, travel) were provided. Data on CRC screening uptake and process measures were included.

**Findings:** FQHCs were located in geographically diverse areas across the country. Number of patients eligible for CRCCP and patient sociodemographic characteristics varied by FQHC. EBIs included multicomponent interventions (e.g., patient and provider reminders, patient assessment and feedback) and provider incentives. The implementation period ranged from 10-36 months. The total number of additional screens conducted ranged from 42 at an FQHC implementing provider reminders to 2,045 at an FQHC implementing multicomponent interventions. Incremental intervention cost per person successfully screened ranged from $12.21 to $288.45.

**Implications for D&I Research:** Awardees and their partner FQHCs vary in the types of interventions implemented and the intensity with which interventions are implemented; thus, outcomes, such as additional persons screened and costs differ. Potential factors that impact cost-effectiveness include FQHC geographic location, size of the patient population served, and type of EBI implemented. Lessons learned from the analysis may be used to inform future program implementation to ensure sustainability.

**Primary Funding Source**

Centers for Disease Control and Prevention

### S65 Costs of recruitment and adoption of a diabetes prevention program- a population health management approach

#### Tzeyu Michaud^1^, Kathryn Wilson^1^, Fabio Almeida^1^, Jeffrey Katula^2^, Paul Estabrooks^1^

##### ^1^University of Nebraska Medical Center, Omaha, NE, USA; ^2^Wake Forest University, Winston-salem, NC, USA

###### **Correspondence:** Tzeyu Michaud (tzeyu.michaud@unmc.edu)

**Background:** Reach is an understudied dissemination and implementation outcome and is often overlooked in justifying the economic feasibility of implementing evidence-based interventions. This is especially important for evidence-based approaches for health promotion and disease prevention. The purpose of this study was to retrospectively assess the costs of a population management approach to identify, engage, and enroll patients in a type 1 hybrid effectiveness-implementation (HEI), diabetes prevention trial and estimated the potential costs of the approach if sustained in typical practice with modifications that reflect population health management approaches typical for chronic disease.

**Methods:** We used descriptive analyses and activity-based costing to estimate the recruitment costs of a population health management approach integrated within a randomized controlled trial. This included the creation of a process map to capture the recruitment process by outlining each activity from initial electronic health record identification of potentially eligible patients in a Midwest healthcare system to post-screening assessment. Measures included total costs and costs per participant recruited. We took the perspective of a healthcare system which may adopt the recruitment process for a diabetes prevention program, and further estimated the replication costs based on the eligibility/process for referral to a CDC-recognized lifestyle change program by type of target population (i.e. Medicare beneficiaries) and/or by input parameters associated with adoption in the scenario and sensitivity analyses (SA).

**Findings:** The total recruitment costs were $485,638, for an average of $811 per participant. Primary costs included lab HbA1c testing ($219,000) recruitment phone calls ($47,157), and personnel ($73,650). SA results indicated that this could be reduced to approximately $300 per participant by removing assessment-associated activities, operational service and supplies for a trial, and varied from $223 to $240 by the type of blood testing if based on the Medicare reimbursement rates.

**Implications for D&I Research:** The project provides a methodology to cost recruitment activities within HEI trials and examine the potential for adaptation to increase ongoing reach for prevention programs in healthcare settings. By using sensitivity analysis salient cost information can be provided to inform future clinical systems changes to improve the reach of existing evidence-based health promotion and disease prevention interventions.

**Primary Funding Source**

Commercial partner

### S66 Dynamics of change agent engagement during implementation of complex interventions

#### Xi Zhu^1^, Jure Baloh^2^, Marcia Ward^1^

##### ^1^University of Iowa, Iowa City, IA, USA; ^2^University of Arkansas for Medical Sciences, Little Rock, AR, USA

###### **Correspondence:** Xi Zhu (xi-zhu@uiowa.edu)

**Background:** Change agents (CAs) are often utilized in implementing complex interventions. The role of CAs in initiating, facilitating, and sustaining change processes is well described in the implementation literature. However, the normative view overlooks the complexity in CAs’ response to change, especially when the implementation process is complex and prolonged. The objective of this study is to examine how CAs’ response to change evolve over time and what factors contribute to their sustained engagement during implementation of complex interventions.

**Methods:** The study applied a longitudinal qualitative design. We followed 14 rural hospitals’ implementation of TeamSTEPPS, a complex quality-improvement (QI) intervention, for two years; and conducted 351 interviews during quarterly visits to the hospitals. The interview focused on implementation activities and CAs’ cognitive, emotional, and behavioral responses to change. Four coders independently coded interview transcripts to extract quotes related to key study constructs. Applying the set-theoretical approach, three investigators calibrated each case’s degree of membership in the following sets (constructs): initial motivation, ongoing implementation activities, level of engagement (quarterly), sustained engagement, and CA work background. Descriptive analysis was used to demonstrate trends of CA engagement in a team context. Fuzzy-set qualitative comparative analysis (QCA) was used to examine factors contributing to sustained engagement.

**Findings:** CAs demonstrated a variety of engagement patterns over time. The common patterns included 1) declined engagement and 2) stable engagement at a high or low level. In the team context, three types of dynamics emerged: sustained high engagement, overall declining engagement, and diminished engagement soon after the onset. QCA revealed that two configurations of conditions have led to sustained CA engagement. For CAs whose work focuses on QI, ongoing implementation activities sustained engagement. For CAs whose work does not focus on QI, strong initial motivation must be present in order to sustain engagement. Unsustained CA engagement occurred when CAs lacked initial motivation and are frontline staff or when CAs had initial motivation, but lacked ongoing implementation activities.

**Implications for D&I Research:** CA engagement in implementation is a dynamic process that warrants future research. CA initial motivation and ongoing implementation activities are important for sustaining CA engagement.

**Primary Funding Source**

Agency for Healthcare Research and Quality

### S67 VA rapidly reduces prescribing after randomized clinical trial shows harm.

#### Heather Campbell, Allison Murata, Todd Conner

##### VA Cooperative Studies Program, Veterans Health Administration, Albuquerque, NM, USA

###### **Correspondence:** Heather Campbell (heather.campbell@va.gov)

**Background:** Traditionally, results from clinical trials are integrated into clinical practice guidelines (CPGs), then adopted within the clinical community. We sought to determine if the Veterans Affairs Nephropathy in Diabetes (VA NEPHRON-D) trial had an impact on prescribing within the VA, which has policies directing communication between Research and the Pharmacy Benefits Manager (PBM), and PBM and clinicians. VA NEPHRON-D indicated patients randomized to combination angiotensin-converting enzyme inhibitor (ACEI) and angiotensin receptor blocker (ARB) therapy were at substantially increased harm for acute kidney injury and hyperkalemia compared to patients receiving ARB monotherapy. We assessed prescribing patterns before and after dissemination of VA NEPHRON-D findings.

**Methods:** The present study is a retrospective database analysis of the population who had type 2 diabetes, chronic kidney disease stages 1-3, and moderately- or severely- increased albuminuria. Combination use was noted when the data indicated deliberate overlap in ACEIs and ARBs. We assessed pooled cross-sectional data from the Corporate Data Warehouse by utilizing interrupted time series analysis. We identified a structural break in July 2008, which served as the starting point; analysis ended November 2017. We assessed the impact in the population and stratified by patient subgroups. We used segmented regression with monthly intervals and accounted for serial autocorrelation. Since there were four dissemination strategies, one of which occurred before trial publication, the timing of impact was of particular interest. We obtained IRB approval.

**Findings:** The first dissemination strategy (i.e., PBM communication) was the only one that occurred before the start of the change in the observed data. Following the PBM communication there were 331.94 fewer combination therapy users per 100,000 (95% CI: -500.27, -163.32, p<0.001) in the first month and 14.84 more combination therapy users per 100,000 (95% CI: 10.27, 19.42, p<0.001) per month compared to the counterfactual. This translates into a 29.54%, 23.49%, and 13.28% relative decrease at six, twelve, and eighteen months, respectively.

**Implications for D&I Research:** The apparent speed and impact of this reduction is encouraging due to the aforementioned safety issues and that it can take seventeen years for trial results to be implemented into practice. VA’s communication policies may be a model for other healthcare organizations.

**Primary Funding Source**

Department of Veterans Affairs

### S68 Clinical redesign strategies for multi-site implementation and quality improvement initiatives at a large academic healthcare system

#### Luming Li^1^, Scott Sussman^2^, Brittany Branson^1^, Melissa Davis^1^, Stephanie Amport^2^, Ian Schwartz^2^

##### ^1^Yale University, New Haven, CT, USA; ^2^Yale New Haven Health, New Haven, CT, USA

###### **Correspondence:** Luming Li (luming.li@yale.edu)

**Background:** Dissemination and implementation (D&I) of evidence-based practices can be difficult at large, multi-site academic hospital systems. Sites operate variably in delivering care due to variable stakeholders and de-centralized local practices. In 2015, a clinical redesign program was formed at Yale New Haven Health. Consisting of medical directors and project managers, the clinical redesign team applied a D&I framework utilizing a 90-day quick turnaround model for driving quality improvement changes within the system. Over the last 4 years, a total of 220 projects were completed using the same methodology, across 5 hospitals and multiple practice settings. The framework has led to the dissemination of multiple evidence-based practices, including rational opioid prescriptions and reductions in the number of unnecessary labs ordered.

**Methods:** The authors used a project tracker to identify high-value case studies within the database of completed clinical redesign projects. They retrospectively analyzed these case studies to identify key factors that contributed to the successful adoption of evidence-based practices in clinical settings. In addition, electronic healthcare record data reports were generated to study the effect of the clinical redesign program on metrics such as length of stay, readmissions, and cost per case.

**Findings:** Key factors required for optimal D&I projects at large academic healthcare systems include interdisciplinary team collaboration, supportive sponsors, focused methods with a target metric, data visualization through clinical dashboards, and strong project management. Early findings show that the program has contributed toward bending the cost curve throughout the time course of the program.

**Implications for D&I Research:**Creating value in healthcare delivery requires a mature infrastructure that incorporates multidisciplinary engagement, leadership support, and data expertise. The clinical redesign is an adaptive framework has yielded significant benefits for a multi-hospital healthcare system. Similar approaches can likely be used in other academic settings with benefit.

### S69 A national evaluation of the veterans health administration diffusion of excellence: Understanding successes and failures in gold status practice implementation and sustainability

#### Andrea Nevedal^1^, Caitlin Reardon^2^, George Jackson^3,4^, Brandolyn White^3^, Sarah Cutrona^5,6^, Allen Gifford^7,8,9^, Elizabeth Orvek^5,6^, Kathryn DeLaughter^6,8^, Lindsay White^8^, Heather King^3,4^, Blake Henderson^10^, Ryan Vega^10^, Laura Damschroder^2^

##### ^1^Center for Innovation to Implementation, VA Palo Alto Health Care System, Veterans Health Administration, Menlo Park, CA, USA; ^2^VA Ann Arbor Healthcare System, Veterans Health Administration, Ann Arbor, MI, USA; ^3^Durham Veterans Affairs Health Care System, Veterans Health Administration, Durham, NC, USA; ^4^Duke University, Durham, NC, USA; ^5^Bedford VA Medical Center, Veterans Health Administration, Bedford, MA, USA; ^6^University of Massachusetts Medical School, Worcester, MA, USA; ^7^Divison of General Internal Medicine, Boston University, Boston, MA, USA; ^8^Bedford & Boston VA Medical Centers, Veterans Health Administration, Bedford, MA, USA; ^9^Department of Health Law, Policy & Management, Boston University, Boston, MA, USA; ^10^United States Veterans Health Administration Innovation Ecosystem, Veterans Health Administration, Washington, DC, USA

###### **Correspondence:** Andrea Nevedal (Andrea.Nevedal@va.gov)

**Background:** The Veterans Health Administration (VHA) Diffusion of Excellence (DoE) provides a model of diffusion for learning health systems. The DoE hosts an annual “Shark Tank” competition to identify and diffuse best practices across facilities. 1,676 innovative practices were submitted by frontline staff in the first four “Shark Tank” cohorts; 48 were designated Gold Status Practices and were rapidly implemented in 63 facilities with 6-months of external facilitation. An aim of this national evaluation is to describe factors contributing to successful Gold Status Practice implementation and sustainability.

**Methods:** We conducted semi-structured telephone interviews with 88 out of 115 invited Gold Status Practice implementation team members, including external facilitators, implementing fellows, and key-stakeholders. At least one representative from 30 out of 31 teams from “Shark Tank” cohorts 2 and 3 participated in an interview. Surveys were emailed 1-2 years after implementation was completed to determine if teams sustained their Gold Status Practice. The Consolidated Framework for Implementation Research (CFIR) informed data collection and directed content analysis to understand factors influencing Gold Status Practice implementation success and sustainability.

**Findings:** About 50% of teams successfully (n=17) implemented their Gold Status Practice in 6-months, but key barriers related to implementation readiness (not having the necessary bureaucratic approvals, infrastructure, staff, or resources in place at the time of implementation) led some teams to partial (n=6) or unsuccessful (n=7) implementation. Despite variation in implementation success, teams reported a high level of practice sustainability overall; 1-2 years after facilitated implementation, 70% of successful (n=12) implementation teams reported practice sustainment and 50% of partial (n=3) or unsuccessful (n=4) implementation teams reported sustainment. Out of 30 teams, few reported partial sustainment (n=5), no sustainment (n=3), or did not respond to the sustainment survey (n=3). Most of the less successful teams completed implementation after the 6-month facilitated implementation period and sustained their practice 1-2 years later.

**Implications for D&I Research:** The VHA DoE is an innovative large-scale model for health systems seeking to identify, diffuse, and sustain best practices. Findings contribute to the literature by describing strengths and weaknesses in the VHA DoE, which can serve as a model of diffusion for other learning health systems.

**Primary Funding Source**

Department of Veterans Affairs

### S70 Factors that contribute to successful adoption of primary palliative care for emergency medicine (PRIM-ER): A mixed-methods study using RE-AIM

#### Sarah Turecamo, Allison Cuthel, Frank Chung, Corita Grudzen

##### New York University, New York, NY, USA

###### **Correspondence:** Sarah Turecamo (sarah.turecamo@nyulangone.org)

**Background:** Half of Americans 65 years and older are seen in the Emergency Department (ED) in the last month of life, and three-quarters visit the ED in the six months before their death. The Primary Palliative Care for Emergency Medicine (PRIM-ER) intervention is a pragmatic, stepped-wedge clinical trial aimed at improving palliative care outcomes in the ED through the integration of provider education (physicians and nurses) and a clinical decision support tool. We present results from the pilot of PRIM-ER using the RE-AIM framework to explore the factors that contributed to successful adoption at two unique EDs.

**Methods:** A mixed methods approach informed by the RE-AIM framework was used to assess adoption. Semi-structured interviews were conducted with six interviewees of varying roles (e.g. nurse champion, data analyst) employed at both EDs. Interviews were audio-recorded and transcribed. Deductive and inductive (grounded theory) approaches were used to code and identify themes, and RE-AIM was applied during the analysis. Quantitative data came from implementation adoption outcomes and a baseline survey assessing provider’s attitudes on Palliative Care.

**Findings:** Both pilot sites successfully implemented all components of the intervention and achieved a high level (>75%) of provider adoption. At baseline 91% of providers (N=189) agreed/strongly agreed that “Many patients would benefit if hospice care were initiated earlier in the course of their illness.” Two themes emerged as facilitators to successful adoption of PRIM-ER: 1) institutional leadership support and 2) established quality improvement (QI) processes. Institutional support included leveraging leadership with authority to a) mandate trainings; b) substitute PRIM-ER education for normally scheduled programming; c) provide protected time for champions to implement intervention components. Interviewees also expressed they were able to adopt the complex intervention successfully through capitalizing on existing QI processes which included a) leveraging interdisciplinary partnerships and communication plans and b) monitoring performance improvement data.

**Implications for D&I Research:** The use of RE-AIM qualitatively to assess adoption is largely understudied. Institutional leadership support and leveraging established QI processes are important facilitators for successful adoption of complex interventions. PRIM-ER researchers will use these findings as they scale up the intervention in the remaining 33 EDs.

**Primary Funding Source**

National Institutes of Health

### S71 Comparing strategies for helping chcs implement guideline-concordant care: Trial results

#### Rachel Gold^1^, Arwen Bunce^2^, Stuart Cowburn^3^, James Davis^1^, Joan Nelson^4^, Deborah Cohen^5^, James Dearing^6^, Michael Horberg^7^, Gerard Melgar^8^

##### ^1^Kaiser Permanente Center for Health Research, Portland, OR, USA; ^2^OCHIN, Inc., Portland, OR, USA; ^3^Practice Based Research Network, OCHIN, Inc., Portland, OR, USA; ^4^Kaiser Permanente, Portland, OR, USA; ^5^Family Medicine, Oregon Health & Science University, Portland, OR, USA; ^6^Communication, Michigan State University, East Lansing, MI, USA; ^7^Mid-Atlantic Permanente Medical Group, Kaiser Permanente, Rockville, MD, USA; ^8^Cowlitz Family Health Center, Cowlitz, WA, USA

###### **Correspondence:** Rachel Gold (rachel.gold@kpchr.org)

**Background:** Statins can reduce cardiovascular disease (CVD) risk in patients with diabetes (DM), but prescribing often lags behind recommendations. We compared how three increasingly intensive implementation support strategies impacted community health centers’ (CHCs) adoption of electronic health record (EHR) clinical decision support tools targeting guideline-concordant statin prescribing in DM. The tools (the ‘CVD Bundle’) were adapted from a previously successful intervention.

**Methods:** In this mixed methods, pragmatic trial, 29 CHCs with a shared EHR were randomized to 3 Arms that received implementation support: 1) Implementation Toolkit (CVD Bundle use instructions; Quality Improvement practice change techniques); 2) Toolkit + in-person training with follow-up webinars; or, 3) Toolkit, training, webinars, + offered practice facilitation. All study CHCs also identified a Champion to oversee related clinic activities. Statin prescription rates were compared across Arms, and with those in >300 additional CHCs which received no implementation support, a non-randomized comparison group. Prescribing (per national guidelines) was measured from 12 months pre-intervention through 36 months post-intervention. We gathered qualitative data from the randomized CHCs via on-site observations, interviews, and phone calls.

**Findings:** Statin prescribing increased pre- to post-intervention for all Arms; only Arm 2 demonstrated a statistically significant change relative to comparison CHCs. Prescribing rates improved more in the study CHCs (7%, 8%, and 5% for Arms 1, 2 and 3 respectively) than the comparison CHCs (3%). These differences were not additive – CHCs that received more intensive implementation support did not have greater improvements in prescribing rates. Qualitative data suggest numerous clinic- and intervention-level factors underlying these results. Implementation strategies were not always applied as planned: the Toolkit was infrequently used, webinar attendance was poor, staff turnover was substantial, and few Arm 3 clinics were able to fully benefit from the offered practice facilitation.

**Implications for D&I Research:** This is one of the first studies to directly compare implementation strategies. The strategies employed here were associated with small improvements in the study CHCs’ guideline-concordant prescribing. Level of implementation support was less impactful than clinic ability to make changes. Guideline dissemination efforts should evaluate adopters’ needs / preferences so that subsequently deployed implementation strategies are well-received

**Primary Funding Source**

National Institutes of Health

### S72 Determinants of evidence-based practice uptake in community ICUs: A mixed methods study

#### Katherine Sterba, Emily Johnson, Nandita Nadig, Simpson Annie, Andrew Goodwin, Kit Simpson, Rebecca Beeks, Emily Warr, Dee Ford

##### Medical University of South Carolina, Charleston, SC, USA

###### **Correspondence:** Katherine Sterba (sterba@musc.edu)

**Background:** Research has advanced evidence-based practices (EBPs) to promote quality care in intensive care units (ICUs) but evidence-to-practice gaps limit their reach. This study’s objective was to characterize key determinants of ICU EBP (e.g., sepsis management) uptake in the rural community setting.

**Methods:** A parallel convergent mixed methods design was used with 6 ICUs receiving a quality improvement (QI) intervention. Guided by the Exploration, Preparation, Implementation and Sustainability framework, we identified inner and outer context factors related to EBP uptake using surveys (N=90), key informant interviews (N=14) and a site tracking log. EBP uptake was defined as completion of a set of 8 protocol, medical record, training and order set steps within 12 months. After completing qualitative (content analysis) and quantitative (descriptive statistics) data analyses independently, site, staff and program factors were summarized within and across hospitals to identify patterns by uptake status.

**Findings:** At the site level, while structural characteristics (hospital size, staff turnover, intensivist availability) did not vary by EBP uptake status, interviews highlighted variability in ICU culture and health system challenges that impacted uptake. Sites with lower uptake described a culture where power dynamics sometimes impeded progress and often had technology/staffing challenges. At the staff level, organizational readiness (mean=4.6, 1-6 scale) and self-efficacy (mean=4.3, 1-5 scale) were consistently high and perceived barriers were low (mean=2.3 of 12 barriers) across sites before QI initiation. Time and financial resources were the most common barriers (>50%). However, interviews highlighted that as initiatives progressed, differences across sites in staff communication and team attitudes and ownership were key uptake influences. Sites with better communication whose staff perceived higher program value had more timely uptake. At the program delivery level, mixed methods data showed that variations in uptake were associated with differences in program engagement and leadership. Sites with higher uptake had better staff training attendance, more program activities, fewer barriers to reaching milestones and stable, engaged nurse and physician champions.

**Implications for D&I Research:** Results characterize multiple influences on EBP uptake in the community ICU setting and highlight the importance of developing supportive implementation strategies (e.g., leadership preparation, culture assessment, marketing tools, trust-building) to accelerate the uptake of EBPs.

**Primary Funding Source**

The Duke Endowment

### S73 Implementing immediate postpartum LARC in rural New Mexico

#### Hannah Palm^1^, James Degnan^2^, Sharla Biefeld^2^, Abigail Reese^2^, Eve Espey^1^, Lisa Hofler^1^

##### ^1^University of New Mexico Health System, Albuquerque, NM, USA; ^2^University of New Mexico, Albuquerque, NM, USA

###### **Correspondence:** Hannah Palm (hpalm@salud.unm.edu)

**Background:** Implementing an immediate postpartum (IPP) long-acting reversible contraception (LARC) hospital program is complex and requires a multidisciplinary team. After New Mexico Medicaid approved reimbursement for IPP LARC, the New Mexico Perinatal Collaborative (NMPC) developed an evidence-based implementation program. Seven rural New Mexico hospitals participated in the program. Our objective was to evaluate the implementation process. The primary study outcome was time from NMPC program component introduction to hospital completion of the step.

**Methods:** In this mixed-methods study, conducted from April 2017 to May 2018, we interviewed 20 key personnel from seven rural New Mexico hospitals that planned to implement IPP LARC programs. NMPC introduced program components in a stepped-wedge design. Participants contributed baseline and follow-up data at four time periods detailing the steps taken towards program implementation and the timing of step completion. Qualitative data were analyzed using directed content analysis principles based in the Consolidated Framework for Implementation Research.

**Findings:** Investigators conducted 43 interviews. The IPP LARC program components included patient education, provider training, nursing education, charge capture, available supplies, and protocols or guidelines. The median time from NMPC introduction of a component to completion by the hospital was 240 days (IQR 150.8–268.5 days). Alternatively, when hospitals requested specific program components, time to completion was a median of 7 days (IQR 3.5–34.5 days). Of the four hospitals that completed all NMPC program components, two currently offer IPP LARC and two do not. Three hospitals declined to complete the NMPC program because reimbursement for IPP LARC could not be confirmed. Cost was the overwhelming barrier to IPP LARC program implementation. Most rural hospitals reported financial vulnerability with an inability to take on additional risk, whereas hospital stability facilitated implementation.

**Implications for D&I Research:** Despite a robust implementation process and hospital engagement, IPP LARC was not implemented at most New Mexico hospitals. Significant flaws in the Medicaid policy were revealed through this process, leading to Medicaid revisions. Clinical champions and financial and administrative stability emerged as key predictors of hospital readiness for implementing IPP LARC. Interventions to improve IPP LARC access must begin at the state Medicaid level to ensure adequate payment for rural hospitals.

**Primary Funding Source**

University of New Mexico Seligman Research Fund

### S74 Audit and feedback to address prescribing of high-risk medications in long-term care: Theory-based process evaluation alongside a pragmatic, factorial, cluster-randomized trial

#### Nicola McCleary^1,2^, Justin Presseau^2^, Laura Desveaux^3^, Holly Witteman^4^, Catherine Reis^5^, Stefanie Linklater^2^, Monica Taljaard^2^, Kednapa Thavorn^2^, Jeremy Grimshaw^2^, Noah Ivers^5^

##### ^1^University of Ottawa, Ottawa, ON, Canada; ^2^Ottawa Hospital Research Institute, Ottawa, ON, Canada; ^3^Womens' College Hospital, Institute for Health System Solutions and Virtual Care, Toronto, ON, Canada; ^4^Université Laval, Québec City, QC, Canada; ^5^Women's College Hospital, Toronto, ON, Canada

###### **Correspondence:** Nicola McCleary (nmccleary@ohri.ca)

**Background:** Providing A&F to long-term care physicians may help them reduce prescribing of high-risk medications (including antipsychotics and benzodiazepines). However, how A&F is designed and presented impacts its effectiveness: how to optimize A&F is unclear. Partnering with a healthcare quality advisory organization, our team is conducting a pragmatic, 2×2 factorial, cluster-randomized trial to test the impact of variations in two factors on A&F effectiveness: A) the comparator (comparing prescribing to top performing peers vs. the provincial average); and B) framing (risk framing (reporting the number of patients prescribed high-risk medication) vs. benefit framing (reporting the number of patients not prescribed). Hypothesised mechanisms of effect were based on Goal Setting Theory and Social Cognitive Theory. We investigated these mechanisms, and the factors shaping A&F use.

**Methods:** In this mixed-methods theory-based process evaluation, physicians who downloaded their A&F were invited to complete an online questionnaire assessing theory-based constructs proposed as mechanisms (intention, self-efficacy, outcome expectations, descriptive norms, and goal prioritization) using 5-point Likert scales. We compared scores using t-tests. We also conducted semi-structured interviews and identified themes related to A&F use.

**Findings:** 33/89 physicians completed the questionnaire. We found a statistically significant difference in descriptive norms (agreement that colleagues are appropriately adjusting antipsychotic prescribing): those in the provincial average arm had higher mean agreement scores than those in the top performing peers arm (Mean(SD) 3.7(0.6) vs 3.0(0.7); p=.003). No other differences in proposed mechanisms were found. Across five interviews, physicians in the provincial average arm and those in the top performing peers arm (a higher target) reported aiming to achieve similar prescribing rates to the comparator. Physicians in the benefit framing arm reported not perceiving such feedback to be immediately actionable.

**Implications for D&I Research:** If the trial shows no effect, the limited influence on proposed mechanisms may explain why. If the trial shows an effect, other mechanisms may be involved. While randomised trials are essential for evaluating whether D&I interventions work, investigating theory-based mechanisms of effect in this way can help establish the ‘how’ and ‘why’ of intervention effectiveness. This supports efforts to refine interventions, replication, and application to other jurisdictions.

**Primary Funding Source**

CIHR

### S75 The perceived usability of an audit and feedback report aiming to improve quality in primary care

#### Catherine Reis^1^, Laura Desveaux^2^, Natasha Kithulegoda^1^, Beth Bosiak^1^, Holly Witteman^3^, Noah Ivers^1^

##### ^1^Women's College Hospital, Toronto, ON, Canada; ^2^University of Toronto, Toronto, ON, Canada; ^3^Université Laval, Québec City, QC, Canada

###### **Correspondence:** Noah Ivers (Noah.Ivers@wchospital.ca)

**Background:** Audit and feedback is a widely used quality improvement strategy, however evidence suggests that how it is designed may impact the effectiveness of the intervention. Health Quality Ontario (HQO), a provincial agency responsible for monitoring and reporting on the quality of health care in the province of Ontario, develops and delivers confidential audit and feedback reports for registered primary care physicians. Administrative data are used to assess a series of quality indicators reported at an aggregate, practice level. We sought to evaluate the perceived usability of this audit and feedback report following it’s redesign, in which user-centered design methodology was employed to optimize the user experience and amplify the effectiveness of the report as a quality improvement tool.

**Methods:** We conducted one-on-one, semi-structured interviews with recipients of the report, recruited by email from HQO and invited to participate in an interview with a member of the research team. Recruitment continued until data saturation was reached. Participants were asked about their overall impressions of the report, whether they felt the report was easy to navigate and what parts of the report they found most useful and why. Participants were also asked to describe what action(s) (if any) they took following their review of the report (e.g., conducted a chart review), and what feature(s) of the report informed those actions. Interviews were transcribed verbatim and inductively coded using thematic analysis.

**Findings:** A total of 17 interviews were completed. Participants struggled to interpret practice level data in an actionable way, but used the report as a source of reinforcement and validation. Three key themes emerged relating to the perceived usability of the redesigned report: 1) Improved clarity of messaging was appreciated but did not equate to usability; 2) Recipient goals and report goals were not aligned; and 3) Variation in how physicians think about audit and feedback influences perceived usability.

**Implications for D&I Research:** While the redesigned report was clear, the disconnect between recipients and the report impeded perceived usability and ultimately impacted the use of the report. Future work is needed to understand how feedback could be designed to align with different quality improvement objectives.

**Primary Funding Source**

CIHR

### S76 Understanding how physicians cognitively engage with audit and feedback

#### Laura Desveaux^1^, Kim Devotta^2^, Noah Ivers^3^, Tara Kiran^2^

##### ^1^Womens' College Hospital, Institute for Health System Solutions and Virtual Care, Toronto, ON, Canada; ^2^St. Michael's Hospital, Toronto, ON, Canada; ^3^Women's College Hospital, Toronto, ON, Canada

###### **Correspondence:** Laura Desveaux (laura.desveaux@wchospital.ca)

**Background:** Audit and feedback (A&F) influences intentions to improve care quality, yet recipients consistently report not acting on their data. While this discrepancy may be driven by contextual factors (e.g. limited resources), recipient factors likely account for some of the gap. To address this, the objective of the current study was to understand how primary care physicians (PCPs) engage with A&F.

**Methods:** Participating PCPs (n=73) received aggregate, practice, clinic, and provincial level data collected from the electronic medical record and other existing databases and were asked to review independently. Peer coaches (also PCPs) were nominated by and trained to support their colleagues in interpreting their data. One-on-one, semi-structured interviews were completed with PCPs who received an A&F report and a focus group was completed with peer coaches. Qualitative data was analyzed inductively using reflexive thematic analysis informed by a constructivist paradigm.

**Findings:** A total of 14 PCPs completed an interview and 8 peer coaches attended the focus group. Three key themes emerged. (1) PCPs (including peer coaches) have different mental models underlying the perceived utility of A&F. The majority of PCPs either believed that the data could provide valuable insights that could drive positive changes in practice (**Mixed Mental Model**) or they believed that the data could be used as a mechanism to monitor changes in practice that were driven by other insights (**Positive Mental Model**). Regardless of mental model, (2) it was hard for PCPs to identify modifiable drivers of performance, and (3) PCPs experience tensions that impact how they interact with data. These include adhering to guidelines versus truly patient-centered care and the importance of doing work versus taking time to reflect.

**Implications for D&I Research:** Recipient factors, including individual capabilities and mental models about feedback influence engagement with A&F. As health systems continue to invest in leveraging routinely collected data to provide A&F, identifying how best to tailor feedback may be a critical link to achieving population impact. A&F initiatives – even those that are well designed – must address recipient characteristics and context to optimize impact. There was a general belief that peer coaches could help address this need while successfully diffusing tensions.

**Primary Funding Source**

Academic Health Science Center Alternate Funding Plan

### S77 Assessing implementation strategies in VA’s national opioid risk management initiative

#### Shari Rogal^1^, Matthew Chinman^1,2,3^, Walid Gellad^1,4^, Maria Mor^1,5^, Hongwei Zhang^1^, Sharon McCarthy^1,3^, Genna Mauro^1^, Jennifer Hale^1^, Elizabeth Oliva^6,7^, Jodie Trafton^6,7,8^, Adam Gordon^9,10^, Leslie Hausmann^1,4^

##### ^1^Center for Health Equity Research and Promotion, VA Pittsburgh Healthcare System, Veterans Health Administration, Pittsburgh, PA, USA; ^2^RAND Corporation, Pittsburgh, PA, USA; ^3^Veterans Integrated Service Network 4 Mental Illness Research, Education and Clinical Center, VA Pittsburgh Healthcare System, Veterans Health Administration, Pittsburgh, PA, USA; ^4^Division of General Internal Medicine, University of Pittsburgh School of Medicine, Pittsburgh, PA, USA; ^5^Department of Biostatistics, University of Pittsburgh Graduate School of Public Health, Pittsburgh, PA, USA; ^6^VA Office of Mental Health and Suicide Prevention, VA Palo Alto Healthcare System, Veterans Health Administration, Menlo Park, CA, USA; ^7^VA Center for Innovation to Implementation, VA Palo Alto Healthcare System, Veterans Health Administration, Menlo Park, CA, USA; ^8^Department of Psychiatry and Behavioral Sciences, Stanford University School of Medicine, Palo Alto, CA, USA; ^9^Informatics, Decision-Enhancement, and Analytic Sciences Center, VA Salt Lake City Health Care System, Veterans Health Administration, Salt Lake City, UT, USA; ^10^Program for Addiction Research, Clinical Care, Knowledge, and Advocacy, University of Utah School of Medicine, Salt Lake City, UT, USA

###### **Correspondence:** Shari Rogal (rogalss@upmc.edu)

**Background:** In March 2018, the VA issued a policy notice requiring VA Medical Centers (VAMCs) to complete case reviews for Veterans identified in the Stratification Tool for Opioid Risk Management (STORM) report as being prescribed opioids and at very high risk for adverse outcomes. Half of VAMCs were randomly assigned to policy notices requiring additional oversight and support if VAMC case review completion rates were <97%. Our HSR&D-funded randomized program evaluation assessed: 1) strategies VAMCs used to implement case reviews, 2) whether those strategies differed in VAMCs receiving policy notices requiring additional oversight, and 3) the association between implementation strategies and case review completion rates.

**Methods:** VAMC points of contact for notice implementation were asked to complete an online survey assessing 1) their VAMCs’ use of 68 possible implementation strategies to ensure case reviews were completed and 2) whether each strategy was used in response to the policy notice. Frequency of strategy use was evaluated and differences in the number of strategies used between VAMCs in the two randomization arms (policy notice with oversight versus policy notice alone) were tested using the Wilcoxon rank sum test. Pearson correlation coefficients were computed to assess the correlation between total strategies used and case review completion rates.

**Findings:** Ninety-nine of 140 VAMCs completed the survey (71%). VAMCs used a median of 23 (IQR=15) implementation strategies, and this did not differ between VAMCs receiving policy notices requiring additional oversight versus policy notices alone (24.5 vs. 23.0, p=0.87). The most commonly used strategies were: using STORM report (90%); informing local opinion leaders of the need to complete case reviews (75%); and recruiting and cultivating relationships with local partners (74%). Overall, VAMCs reported that most (80%) implementation strategies were used because of the policy notice. There was no association between the total number of strategies used and the case review completion rate (r=0.11, p=0.31).

**Implications for D&I Research:** VAMCs used a variety of implementation strategies in response to the policy notice, regardless of whether additional oversight was described in the notice. Implementation strategy surveys can provide valuable information about how sites respond to VA policies.

**Primary Funding Source**

Department of Veterans Affairs

### S78 Determinants of implementation success in complex inter-organizational relationships: A mixed-methods evaluation of veteran directed care

#### Nina Sperber^1^, Edward Miech^2^, Alecia Clary^3^, Kathleen Perry^1^, Merle Edwards-Orr^4^, Courtney Van Houtven^5^, Kali Thomas^6^, James Rudolph^7^

##### ^1^Durham VA Health Care System, Veterans Health Administration, Durham, NC, USA; ^2^VA PRIS-M QUERI, Veterans Health Administration, Indianapolis, IN, USA; ^3^The University of North Carolina at Chapel Hill, Chapel Hill, NC, USA; ^4^Applied Self-Direction, Boston, MA, USA; ^5^Duke University, Durham, NC, USA; ^6^Providence VA Medical Center, Veterans Health Administration, Providence, RI, USA; ^7^Brown University School of Public Health, Providence, RI, USA

###### **Correspondence:** Nina Sperber (nina.sperber@duke.edu)

**Background:** Veteran Directed Care (VDC) provides Veterans at risk of nursing home placement a flexible budget for long-term care services. VDC is administered through Aging and Disability Network Agencies (ADNAs), community-based providers affiliated with the US Department of Health and Human Services Administration for Community Living. As part of an evaluation of VDC implementation that considers both sides of the partnership (e.g., Veterans Affairs (VA) medical centers (VAMCs) and ADNAs), we sought to identify determinants of successful VDC implementation.

**Methods:** Focusing on 5 different VAMCs that adopted VDC in 2017 or 2018, we interviewed coordinators from participating VAMCs and ADNA partners at two time points: two and eight months after starting. We systematically applied over 20 determinants from the Consolidated Framework for Implementation Research (CFIR) to interview transcripts and scored each construct for valence and magnitude. The 5-point scale ranged from +2 (“strong positive influence on implementation”) to -2 (“strong negative influence”). We scored each determinant at three levels of analysis: VAMC, ADNA, and VAMC-ADNA partnership, yielding over 400 data points. To find determinants of successful implementation, 6 or more Veterans enrolled at 8 months, we used Configurational Comparative Methods (CCMs), a mathematical approach for identifying necessary and sufficient conditions associated with an outcome of interest.

**Findings:** At the VAMC/ADNA partnership level, the determinants “External Change Agent +2” (e.g. family member advocating for a program) and “Networks & Communication +2” (e.g., regular scheduled meetings between partners) uniquely distinguished programs with higher enrollment. Both were sufficient for implementation success only at the +2 level (i.e., strong positive influence). The VAMC side of the VDC partnership appeared to drive the External Change Agent +2 score: while the ADNA score could be anywhere between 0 and +2, the VA score had to be +2 for implementation success to occur. Alternatively, Networks & Communication needed to present as +2 from the ADNA, whereas the VA score could be either +1 or +2 for implementation success to occur.

**Implications for D&I Research:** In inter-organizational implementation like VDC, each partner may require different conditions for overall implementation success; Configurational Comparative Methods offer new ways to identify necessity and sufficiency of specific implementation conditions.

**Primary Funding Source**

Department of Veterans Affairs

### S79 Comparing health system and physician practice influences on social needs screening

#### Jennifer Frehn^1^, Amanda Brewster^1^, Hector Rodriguez^1^, Stephen Shortell^2^

##### ^1^University of California, Berkeley, Berkeley, CA, USA; ^2^Health Policy and Management, University of California, Berkeley School of Public Health, Berkeley, CA, USA

###### **Correspondence:** Jennifer Frehn (jfrehn@berkeley.edu)

**Background:** System-owned physician practices are becoming increasingly prominent as health systems address the challenges of new value-based payment models. System-level policies and resources can support dissemination of innovations such as screening for social needs, but further evidence is needed to understand how health systems influence innovation implementation among their member practices. We examined how system versus practice characteristics influence whether practices implement screening for social needs, a care delivery innovation recommended to mitigate disparities.

**Methods:** The 2018 National Survey of Healthcare Organizations and Systems, a nationally representative survey of physician practices (N=818) and their health systems owners (N=253), was analyzed to examine whether practice or system characteristics explain more of the variance in a practice’s screening for social needs (food insecurity, housing instability, utility needs, interpersonal violence, transportation needs). Multilevel models with practices nested in systems were estimated.

**Findings:** Systems-owned practices screened for an average of 1.7 (34%) of the 5 social needs assessed. The intraclass correlation indicates 16% of the variation in practices’ social needs screening is attributable to differences among the health systems that own them, with 84% attributable to differences between practices. In multivariable analyses including both practice and system characteristics, only practice characteristics were significantly and positively associated with a practice’s social needs screening. These included a practice’s health information technology capacity (0.19, p = 0.001), innovation culture (0.25, p < 0.001), patient engagement strategies (0.54, p < 0.001), the percentage of a practice's revenue from Medicaid (0.26, p < 0.001), having a method for identifying complex, high-need patients (6.76, p = 0.015), and being an FQHC (8.59, p = 0.012). The R^2^ for the model containing only practice-level variables was more than 10 times larger than the R^2^ for the model with only systems-level variables (0.26 vs 0.02).

**Implications for D&I Research:** Practice-level characteristics explain more of the variance in a practice's screening for social needs than do systems-level characteristics. Efforts to expand social needs screening among system-owned practices should focus on strategies at the practice level, such as initiating improvement efforts to support dimensions of practices associated with screening, including increasing technology capacity and patient engagement strategies.

**Primary Funding Source**

Agency for Healthcare Research and Quality

### S80 Interpreting variation in community-based care coordination efforts to reduce state and national Medicare hospital readmissions using the consolidated framework for implementation research (CFIR)

#### Jane Brock^1^, Alaina Brothersen^1^, Brianna Gass^1^, Lacey McFall^1^, Kati Ollom^2^, Christina Goatee^3^, Amy Huebschmann^4^, Jodi Holtrop^4^

##### ^1^Telligen, Greenwood Village, CO, USA; ^2^Telligen, West Des Moines, IA, USA; ^3^Centers for Medicare & Medicaid Services, Baltimore, MD, USA; ^4^University of Colorado School of Medicine, Aurora, CO, USA

###### **Correspondence:** Jane Brock (jbrock@telligen.com)

**Background:** Quality Improvement Organizations (QIOs) developed community coalitions to align care coordination efforts for Medicare beneficiaries in order to reduce readmission rates within geographically defined communities. This CMS (Centers for Medicare & Medicaid Services) funded national quality improvement program worked with 380 coalitions from 2014-2019, facilitating a variety of interventions within each community. Baseline readmission rates among communities, calculated from claims data, varied from 17.7 to 112 readmissions/1000 beneficiaries. Program results through mid-2017 ranged from +35% (high performance) to -44.2% (low performance) relative improvement. We applied an implementation framework (CFIR) to the QIO efforts to define common characteristics of interventions, implementation strategies, and contexts in which improvement efforts took place. We identify features associated with successful intervention implementation, and with changes in readmission rates.

**Methods:** We used explanatory sequential mixed methods, beginning with a written survey, followed by qualitative interviews. A purposively selected sample of 22 communities represented a range of relative improvement, geographic characteristics and baseline readmissions rates. The survey measured the QIO’s perceived influence of individual CFIR constructs on community readmission rates over time; interviews elicited details and mechanisms. Two independent reviewers qualitatively coded transcribed interviews. Final ratings for the influence of each CFIR construct on community performance were assigned by consensus, ranging from -2 (strong negative influence) to +2 (strong positive influence).

**Findings:** Early findings indicate that high performers used interventions with relative advantage (33% vs. 25%), and adaptability (66% vs. 25%); developed coalitions with networking and communication strength (50% vs. 38%); and were less likely to have negative implementation climates (0% vs 50%), or struggle with engaging others in the work (0% vs. 25%). Neither strength of intervention evidence, nor elements of coalition structure differentiated performance. Outer setting constructs were modified through inductive coding. Next steps will compare domain ratings between high and low performing communities using chi-square tests.

**Implications for D&I Research:** Communities effectively reducing readmissions had coalitions with more favorable implementation climates, more robust stakeholder engagement strategies, and interventions aligned with local concerns and capabilities. The CFIR can help guide, monitor and evaluate community-based improvement initiatives, although further development of outer setting constructs is needed.

**Primary Funding Source**

Centers for Medicare and Medicaid Services

### S81 Using correlational and configurational comparative methods to identify implementation strategies associated with successful uptake of evidence-based practices for advanced liver disease in the veterans health administration

#### Vera Yakovchenko^1^, Timothy Morgan^2^, Rachel Gonzalez^3^, Angela Park^4^, Maggie Chartier^5^, David Ross^5^, Emily Comstock^6^, Jasmohan Bajaj^7^

##### ^1^Center for Healthcare Organization and Implementation Research, VA, BridgeQUERI & CHOIR, Veterans Health Administration, Bedford, MA, USA; ^2^Department of Gastroenterology, VA Long Beach Healthcare System, Veterans Health Administration, Long Beach, CA, USA; ^3^Department of Research, VA Long Beach Healthcare System, Veterans Health Administration, Long Beach, CA, USA; ^4^Boston VA Healthcare System, New England Veterans Engineering Resource Center, Veterans Health Administration, Boston, MA, USA; ^5^Office of Patient Care Services; HIV, Hepatitis, and Public Health Pathogens Programs, Veterans Healthcare Administration, Washington, DC, USA; ^6^Baltimore VA Medical Center, Veterans Health Administration, Baltimore, MD, USA; ^7^Hunter Holmes McGuire VA Medical Center, Veterans Health Administration, Richmond, VA, USA

###### **Correspondence:** Vera Yakovchenko (vera.yakovchenko@va.gov)

**Background:** The Department of Veterans Affairs (VA) established a national collaborative (HIT) composed of regional teams of interdisciplinary and interspecialty providers, leaders, and staff tasked with supporting local implementation strategies to increase evidence-based practices (EBPs) for ALD. The aim of this evaluation was to assess how site-level implementation strategies were associated with measures of quality of two EPBs: esophageal varices screening via esophagogastroduodenoscopy (EGD) and hepatocellular carcinoma (HCC) surveillance.

**Methods**: A provider at each VA site was invited to complete an online survey (N=63, response rate 48%) examining the use of 73 implementation strategies as defined by the Expert Recommendations for Implementing Change (ERIC) study. HCC and EGD data was obtained from the VA’s Corporate Data Warehouse. We used descriptive statistics, correlational analyses, and comparative configurational methods (CCMs) to assess the relationships between ERIC strategy use and provision of EBP ALD care. For CCM analyses, we set the consistency to 100% and defined higher-performing sites as those above the median for ALD care (composite metric of EGD and HCC screening).

**Findings:** Sites used a median of 12 (IQR 7-27) strategies to improve ALD care. The most commonly endorsed strategies were data warehousing techniques (73%), change physical structure and equipment (67%), and change record systems (62%). Among the 73 strategies, 12 strategies were individually associated with better performance in EGD screening and two in HCC surveillance. CCMs identified six strategies in combination with one another that were linked to ALD outcomes in 72% of higher-performing sites. Three strategies overlapped across methodologies: 1) provide clinical supervision, 2) organize clinician teams and provide time to support one another’s learning, and 3) build on existing high-quality working relationships and networks to promote information sharing and problem solving.

**Implications for D&I Research:** This evaluation from the first year of a national quality improvement initiative identified 12 strategies associated with improved EBP’s for ALD using correlational analyses and 6 using CCM. Three strategies identified by CCM were not identified by correlational analyses. This innovative, multi-method approach to identifying key implementation strategies associated with improved performance on quality measures will help to inform future intervention and policy development.

**Primary Funding Source**

Department of Veterans Affairs

### S82 How variation in health system implementation affects the success of colorectal cancer screening outreach

#### Amanda Petrik^1^, Edward Miech^2^, Jennifer Coury^3^, Beverly Green^4^, Laura-Mae Baldwin^5^, Nicole Merrithew^3^, Kelly Coates^3^, Gloria Coronado^1^

##### ^1^Kaiser Permanente Center For Health Research, Portland, OR, USA; ^2^Regenstrief Institute, Indianapolis, IN, USA; ^3^CareOregon, Portland, OR, USA; ^4^Kaiser Permanente Washington Health Research Institute, Seattle, WA, USA; ^5^University of Washington School of Medicine, Seattle, WA, USA

###### **Correspondence:** Amanda Petrik (Amanda.F.Petrik@kpchr.org)

**Background:** Colorectal cancer (CRC) screening remains an underused preventive health measure despite its effectiveness at reducing CRC incidence and mortality. State and federal programs, health care systems, and insurance plans have successfully improved CRC screening rates using mailed fecal immunochemical test (FIT) outreach. Nevertheless, challenges remain with implementing such programs into practice. BeneFIT is a collaboration between research institutions and health insurance plans to understand the implementation of a health plan-driven mailed FIT program. The health plan in Oregon used an implementation model that involved close collaboration with health centers to implement this program.

**Methods:** The program facilitated the centralized mailing of FIT tests to patients (through a mail vendor); health centers engaged in various activities to support the program, including reviewing patient lists (‘scrub’) and removing names of patients who had not yet established care at the health center or were not candidates for screening, delivering patient reminders by phone, and offering patient incentives. We categorized health centers’ level of implementation (high, low) based on FIT completion rates and applied Configurational Comparative Methods (CCMs) to identify combinations of organizational-level factors that distinguished among high and low implementing health centers during the first year of participation in the program.

**Findings:** The health plan mailed FIT tests to over 8,300 patients at 17 clinics from 2016-2017. FIT return rates varied by health center (range = 3.9 - 31.4%). Eleven of the 17 health centers had FIT completion rates of ≥ 19% and were categorized as high implementing health centers. CCM analysis identified four solution paths that uniquely distinguished between high and low implementation with 100% consistency and 82% coverage. The combinations were: scrubbing lists and offering a mail return option to patients; providing gift cards and offering a mail return option to patients; scrubbing lists and providing gift cards; or solely offering a mail return option and implementing in two or more locations within the same organization.

**Implications for D&I Research:** Our findings from CCM analysis of a Medicaid health plan-initiated mailed outreach program can inform effects to optimize health center activities to support optimal implementation.

**Primary Funding Source**

Centers for Disease Control and Prevention

### S83 PrEP implementation during community-based HIV testing in Florida: Application of the consolidated framework for implementation research

#### DeAnne Turner^1,2^, Elizabeth Lockhart^2^, Wei Wang^2^, Robert Shore^3^, Ellen Daley^2^, Stephanie Marhefka^2^

##### ^1^Yale University, New Haven, CT, USA; ^2^University of South Florida, Tampa, FL, USA; ^3^Midway Specialty Care Center, Wilton Manors, FL, USA

###### **Correspondence:** DeAnne Turner (deanne.turner@yale.edu)

**Background:** HIV testing/counseling is a critical point during which non-clinical staff could intervene, discuss and/or refer clients for pre-exposure prophylaxis (PrEP; a once a day pill that significantly lowers a person’s risk of acquiring HIV). The goals of this study were to identify underlying subgroups of PrEP implementers among staff who provide HIV testing and to understand the contextual factors affecting PrEP implementation within HIV testing environments.

**Methods:** Latent Class Analyses (LCA) were performed using MPlus.v.8 on a sample of 144 HIV testing/counseling staff in Florida. LCA groups participants based on similarities in how they answer a predetermined set of questions (here, five items related to PrEP-implementation behaviors). The final LCA and corresponding latent classes were determined based upon fit indices and theoretical interpretation. Two generalized linear mixed models were conducted in SPSS to estimate PrEP implementation as a function of constructs from the Consolidated Framework for Implementation Research (CFIR). Qualitative interviews were conducted with 30 participants and analyzed thematically. Mixed methods data were analyzed via joint analysis and triangulation. This study was approved by the IRB at the University of South Florida.

**Findings:** Based on consideration of fit statistics and theoretical relevance, a 3-class LCA was selected. Class one (“Universal”) included participants who often spoke with clients about PrEP, regardless of client eligibility, and often shared written information about PrEP. Class two (“Eligibility Dependent”) included staff who were likely to discuss PrEP if they felt their client was eligible. Staff in Class 3 (“Limited”) spoke to clients about PrEP inconsistently. These latent groups also emerged within qualitative interviews. Several CFIR variables were associated with PrEP implementation, including relative priority and available resources. Characteristics of HIV testing staff, including race and sexual orientation also predicted implementation group membership; participants identifying as a sexual or racial minority were less likely to be in the “Limited” implementation group than their peers.

**Implications for D&I Research:** This study provides a mixed-methods application of the CFIR to a novel method of HIV prevention – PrEP. Addressing the contextual and organizational factors that affect PrEP implementation may help HIV testing staff to more seamlessly implement PrEP education and referrals.

### S84 Exploring the heterogeneity of factors that may influence implementation of PrEP in family planning clinics: A latent profile analysis

#### Kaitlin Piper, Regine Haardörfer, Cam Escoffery, Anandi Sheth, Jessica Sales

##### Emory University, Atlanta, GA, USA

###### **Correspondence:** Kaitlin Piper (kaitlin.piper@emory.edu)

**Background:** Title X-funded family planning clinics have been identified as optimal sites for delivery of pre-exposure prophylaxis (PrEP) for HIV prevention. However, PrEP has not been widely integrated into family planning services, especially in the Southern US, and data suggest there may be significant implementation challenges in this setting. Because Title X clinics vary greatly in provider-, organizational-, and community-level characteristics, there is likely variation in capacity to implement PrEP across clinics.

**Methods:** We conducted a survey from February-June 2018 among providers and administrators of non-PrEP providing Title X-funded clinics across 18 southern states. Survey items were designed using the Consolidated Framework for Implementation Research (CFIR) to assess constructs relevant to PrEP implementation. To explore the heterogeneity of CFIR-related determinants and identify distinct segments of Title X clinics, a latent profile analysis was conducted using nine CFIR constructs: complexity, relative advantage, cost, attitudes, implementation climate, compatibility, leadership engagement, available resources, and cosmopolitanism. We then conducted a multi-level analysis (accounting for nesting of participants within clinics) to test whether class membership was associated with readiness for implementation of PrEP, controlling for key sociodemographic characteristics. The study was approved by the university affiliated Institutional Review Board.

**Findings:** 420 healthcare providers/administrators from 229 non-PrEP providing Title X clinics participated in the study. We identified six classes of participants that each had distinct patterns of perceptions across the CFIR-related determinants of readiness for PrEP implementation. Two classes of participants showed positive perceptions across CFIR factors (i.e. “high capacity”) (n=173), two classes held negative perceptions (i.e. “low capacity”) (n=87), and one class held neutral perceptions (i.e., “moderate”) (n=160). Class membership was related to numerous provider-level (i.e., years of experience, ability to prescribe medication), clinic-level (i.e., on-site insurance support), and community-level characteristics (i.e., DHHS region, county-level HIV prevalence). Compared to the “high capacity” class, the “moderate” (B=-0.45, SE=0.06, p<0.0001) and “low capacity” (B=-0.53, SE=0.07, p<0.0001) classes had significantly lower levels of implementation readiness.

**Implications for D&I Research:** Latent profile analyses can help researchers understand how context-specific implementation determinants vary across individuals and settings (such as clinics), allowing implementation planning to be tailored to the specific needs of each segment of the population.

**Primary Funding Source**

National Institutes of Health

## Global Dissemination and Implementation Science

### S85 Advancing a global agenda for implementation of integrated care

#### Nicole Stadnick^1^, Euan Sadler^2,3^, Jane Sandall^3^, Cristina Fernandez Turienzo^3^, Ian Bennett^4^, Jeffrey Borkan^5^, Bibilola Oladeji^6^, Oye Gureje^6^, Gregory Aarons^1,7^, Marisa Sklar^1,7^

##### ^1^University of California, San Diego, La Jolla, CA, USA; ^2^University of Southampton, Southhampton, United Kingdom; ^3^King’s College London, London, United Kingdom; ^4^University of Washington, Seattle, WA, USA; ^5^Brown University, Providence, RI, USA; ^6^University of Ibadan, Nigeria, Ibadan, Nigeria; ^7^Child and Adolescent Services Research Center, San Diego, CA, USA

###### **Correspondence:** Nicole Stadnick (nstadnick@ucsd.edu)

**Background**: Integrated care is the coordination of primary and specialty health care and it is a highly promising and pragmatic approach to improving delivery of health services and patient outcomes. While implementation of integrated care approaches is gaining strong interest and investment globally, there are no formal guidelines for this process applicable to diverse healthcare systems. Furthermore, there is a complex interplay of influences at multiple socioecological levels that are critical for successful and sustained integrated care implementation in health systems.

**Methods**: A multiple case study design was employed and informed by the Exploration, Preparation, Implementation, Sustainment (EPIS) framework (Aarons et al., 2011) to illuminate shared and unique implementation processes in contemporary integrated care implementation efforts. The study addressed three objectives: 1) To illustrate exemplar integrated care implementation efforts through seven case studies spanning five countries (England, Israel, Nigeria, USA, Vietnam) targeting a range of healthcare systems, patient populations (e.g., older adults with frailty, children with autism) and implementation strategies and outcomes; 2) To synthesize shared and unique barriers and facilitators across implementation efforts using the phases and factors of the EPIS framework; and 3) To develop a proposed agenda for future integrated care implementation research to generalize across countries and health service systems.

**Findings**: Qualitative synthesis of case study descriptions revealed common themes that informed our proposed agenda to advance integrated care implementation. Specifically, five outer context factors (e.g., leadership, inter-organizational environment and networks), five inner context factors (e.g., leadership, fidelity monitoring), one bridging factor (community-academic partnerships) and one innovation factor (fit between integrated care and the implementation context) were identified as shared across the case studies.

**Implications for D&I Research:** ased on these findings, we propose an agenda for advancing integrated care implementation efforts related to three broad goals: 1) consider the role of funding at multiple levels of implementation; 2) foster meaningful collaboration with stakeholders across phases of implementation; and 3) cultivate opportunities for clear communication about integrated care implementation. This agenda is an initial step towards prioritizing goals and implementation strategies for researchers, practitioners and policymakers across countries and healthcare systems involved in integrated care implementation.

**Primary Funding Source**

National Institutes of Health

### S86 Using behavioral economics approaches to design interventions to decrease cervical cancer mortality in Zambia: Results from a discrete choice experiment on preferences for screening services

#### Sujha Subramanian^1^, Madeliene Jones^2^, Sonja Hoover^3^

##### ^1^RTI International, Waltham, MA, USA; ^2^RTI International, RTP, NC, USA; ^3^Social Policy, Health and Economics Research, RTI International, North Waltham, MA, USA

###### **Correspondence:** Sonja Hoover (shoover@rti.org)

**Background:** Around the world a woman dies of cervical cancer about every 2 minutes and Sub-Saharan African countries experience the highest mortality rates. Even in this era of Human Papillomavirus vaccination, screening remains an essential approach to reduce the burden from cervical cancer in the immediate future. Zambia, which has one of the highest burdens of cervical cancer, is in the process of scaling up cervical cancer screening. The purpose of this study is to conduct a discrete choice experiment (DCE) to evaluate individual level preferences to tailor the screening program.

**Methods:** Based on extensive formative research, the final DCE attributes included distance to facility (5 levels), availability of free versus paid transportation, middle-aged versus young nurse provider, half day versus full day wait and cost (5 levels). Seven distinct groups of men and women (350 to 400 in each cohort) completed the DCEs and represented geographically diverse cohorts (rural and urban settings; clinic and community locations). Each respondent completed 8 choice decision sets based on well-balanced and near-orthogonal fractions of the full-choice design and a total of 2,670 individuals completed the DCEs. The main analysis was conducted using a mixed logit model.

**Findings:** Our results indicate that the preferences examined were broadly dichotomous. There was universal preference among women and men for the screenings to be performed by a middle-age nurse instead of a young nurse. There was strong preference (ranging from 76% to 100%) to receive free care rather than paying 100 Zambian Kwachas. Furthermore, rural residents had a stronger preference for shorter walking distance to the clinic than urban residents (81% versus 73% selected 1 hour compared to 3 hours of walking). Additional simulations to explore these findings are ongoing.

**Implications for D&I Research:** In Zambia, free provision of cervical cancer screening and the availability of older female nurse providers are essential for high uptake. Rural residents, both men and women, indicated strong preference for travel time of less than one hour to the clinic so wide geographic available of screening services is important. The successful completion of this study reveals that systematic, behavioral economics approaches can be used to inform program planning.

**Primary Funding Source**

National Institutes of Health

### S87 Costs of a diabetes prevention intervention for Caribbean and Caribbean-descent individuals: Preliminary results

#### Saria Hassan^1^, Marcella Nunez-Smith^1^, Oswald P. Adams^2^, Xiao Xu^1^

##### ^1^Yale University School of Medicine, New Haven, CT, USA; ^2^University of the West Indies, Faculty of Medical Sciences, Bridgetown, Barbados

###### **Correspondence:** Saria Hassan (saria.hassan@yale.edu)

**Background:** Understanding cost implications of interventions for chronic disease prevention is critical for identifying cost-effective and efficient approaches to care. Life-style Intervention with Metformin Escalation (LIME) is a multi-site pragmatic trial of a culturally appropriate diabetes prevention intervention that combines lifestyle change and metformin medication to reduce the incidence of diabetes among Caribbean and Caribbean-descent individuals. This study reports preliminary findings on the cost of the LIME intervention.

**Methods:** The LIME trial enrolls participants between the ages of 40 and 60 in New York, Puerto Rico, US Virgin Islands, Barbados and Trinidad. We used preliminary data from two sites (Barbados and Trinidad) for this analysis. Detailed monthly logs were completed by implementers at these sites to track resources used for delivery of the intervention (facility space, personnel time, transportation, supplies). Participant-related costs were collected through participant surveys (e.g. lost-time from work, transportation). Cost of metformin was based on participant’s actual utilization and national average wholesale price. All costs were reported in 2018 U.S. dollars.

**Findings:** Sixty-one participants were enrolled in the LIME study in Barbados and Trinidad by October 2018, each participant was enrolled in one workshop series (comprising of 6 weekly sessions). To accommodate all participants, 6 workshop series (36 weeks) were held in total. Participants returned for follow up with 90% retention at 6-months. Cost of supplies for all the workshops was $544.74, while cost of personnel time for conducting the workshops amounted to $3611.28. This resulted in an average of $68.13 per participant. Other cost data are being analyzed and will be completed by September 2019. We will report on direct and indirect costs of the LIME intervention in Barbados and Trinidad.

**Implications for D&I Research:** Our study demonstrated feasibility of collecting rigorous cost data in low resource settings. Understanding the cost of LIME, a culturally appropriate diabetes prevention intervention, will inform future scale-up and scale out. It will also facilitate future evaluation of the cost-effectiveness of the LIME intervention by additionally accounting for patient outcomes and longer-term consequences (e.g., prevention of diabetes).

**Primary Funding Source**

National Institutes of Health

### S88 Offering the choice of self-administered oral HIV testing (CHIVST) among long-distance truck drivers in Kenya: A trial-based cost-effectiveness analysis

#### Deo Mujwara^1^, Elizabeth Kelvin^2^, Gavin George^3^, Eva Mwai^4^, April Kimmel^1^

##### ^1^Virginia Commonwealth University School of Medicine, Richmond, VA, USA; ^2^City University of New York Graduate School of Public Health and Health Policy, New York, NY, USA^3^University of KwaZulu-Natal Westville Campus, Durban, South Africa; ^4^NorthStar Alliance, Nairobi, Kenya

###### **Correspondence:** Deo Mujwara (mujwarad@vcu.edu)

**Background:** Awareness of HIV status is critical for achieving UNAIDS targets, particularly for sub-populations at high risk of acquiring and transmitting HIV. These sub-populations require targeted, resource-intensive strategies for HIV test uptake, a challenge when HIV funding is limited. We conducted a trial-based cost-effectiveness of CHIVST in one high-risk population—long distance truck drivers—in Kenya.

**Methods:** We leveraged data from a randomized-controlled trial of CHIVST (intervention, n=150) versus provider-administered testing (control, n=155). Economic cost data (HIV test kits, medical supplies, labor, capital and overhead costs, patient time), including upper and lower bounds, came from the literature and reflected a societal perspective. Generalized Poisson and linear gamma regression models estimated the effectiveness (relative risk) and incremental costs (2017 I$), respectively, with incremental effectiveness calculated as the reciprocal of the absolute risk difference and reported as the number needing to receive CHIVST for an additional HIV test uptake. We reported incremental cost-effectiveness ratios (ICERs), with 95% confidence intervals (CIs) calculated using Fieller’s theorem. Deterministic sensitivity analysis identified key cost drivers; non-parametric bootstrapping generated cost-effectiveness acceptability curves to assess uncertainty in the ICER. The economic performance of CHIVST was evaluated using a willingness-to-pay threshold of 3x GDP per capita for Kenya (3*I$1594=I$4782).

**Findings:** HIV test uptake was 23% more likely for CHIVST versus provider-administered testing, with six individuals needing to be offered CHIVST for an additional HIV test uptake (6.25, 95% CI 5.00-8.33). The mean cost per patient was more than double for CHIVST (I$26.56 vs I$10.47). The incremental cost-effectiveness of CHIVST was I$97.21 [95% CI 65.74-120.98] per additional HIV test uptake compared to provider-administered HIV testing. Self-test kits and patient time were the main cost drivers of the ICER, with findings robust even in a worst-case scenario of all upper bound economic costs. The probability of CHIVST being cost-effective approached one at a willingness-to-pay threshold of I$140.

**Implications for D&I Research:** CHIVST is a highly efficient use of resources compared to provider-administered testing. Future work should consider the long-term health consequences, including new HIV infections, and economic consequences of this HIV testing approach, as well as its application in other high-HIV-risk populations.

**Primary Funding Source**

International Initiative for Impact Evaluation (3IE).

### S89 Implementing a diabetes self-management intervention in a Mexican regional health system: Testing a scalable unit

#### Benjamin Aceves^1^, Catalina Denman^2^, Maia Ingram^1^, Jill Guernsey de Zapien^1^, Tomas Nuno^1^, Elsa Cornejo^2^, David Garcia^1^, Cecilia Rosales^1^

##### ^1^University of Arizona, Tucson, AZ, USA; ^2^El Colegio de Sonora, Hermosillo, SO, Mexico

###### **Correspondence:** Benjamin Aceves (benjaminaceves@email.arizona.edu)

**Background:** Mexico has an epidemiological crisis related to type 2 diabetes mellitus (T2DM), reporting in 2016 almost 100,000 attributable deaths. This specifically affects low-income Mexican citizens who face several issues associated with T2DM, including lack of access to information and resources on self-management. Centros de Salud (health centers) are meant to serve those individuals by providing health services, including resources on chronic disease prevention. *Meta Salud Diabetes (MSD)*, a 13-session weekly self-management intervention developed to prevent cardiovascular complications and other health issues within the T2DM population, was tested for efficacy and designed for health centers to improve services.

**Methods:** Investigators conducted a test scale up study from February to June 2019 within Jurisdiction VI (Northwest Sonora) of the Sonora Ministry of Health using the Institute for Healthcare Improvement framework for scaling up health interventions. Initially, investigators trained 19 stakeholders within the regional health system at various social ecological levels on the intervention and implementation process. The intervention was implemented in all five T2DM support groups (GAMs) within the regional health system, and specifically designated a coordinator from the regional office to support the entire process.

**Findings:** A total of 72 participants were consented and behavioral and biological (HbA1c, pressure, BMI) measures taken at baseline; approximately 72% of participants completed the intervention and were present at post-intervention measurements. Implementation fidelity measures demonstrated comprehensive adherence to intervention curriculum and potential moderators were assessed for influences on implementation. Findings demonstrated that training stakeholders at every social ecological level provided those facilitating MSD with support by allotting time and resources to properly prepare for sessions, while also ensuring adherence to intervention curriculum. Additionally, designating a coordinator from the regional health office assisted facilitators with resolving barriers to intervention implementation and supported in working toward federal accreditation of GAMs. Initial statistical analysis demonstrates improved behavioral and biological measures when comparing baseline to post-intervention.

**Implications for D&I Research:** Overall, results provide evidence for using regional health systems as a scalable unit when implementing chronic disease self-management interventions state- and nationwide. This study will help inform future efforts to scale up the intervention in various states throughout Mexico.

**Primary Funding Source**

National Institutes of Health

### S90 Long-term sustainability of improvements in tobacco use treatment in health centers in Vietnam

#### Donna Shelley^1^, Nina Siman^1^, Nancy VanDevanter^1^, Trang Nguyen^2^, Charles Cleland^1^, Nam Nguyen^2^

##### ^1^New York University, New York, NY, USA; ^2^Institute of Social and Medical Sciences, Hanoi, Vietnam

###### **Correspondence:** Donna Shelley (dreffshelley@gmail.com)

**Background:** Little is known about how well or under what conditions advances in health care delivery, once adopted, are sustained and effects maintained in routine practice, particularly in low-middle income countries. We conducted an NCI-funded 2-arm cluster randomized study of the effectiveness of two strategies for increasing implementation of tobacco use treatment (TUT) guidelines in 26 health centers in Vietnam: Ask (screen for tobacco use), Advise (advise to quit), Assist (brief counseling), and in the intervention arm, refer to a village health worker (VHW) for 3 sessions of counseling (Refer) (i.e., 3As vs 3As+R). We found a 10-fold increase in clinician adherence to the 3As at 12-months (end of intervention) in both arms and high referral rates to VHWs in the 3As+R arm (60%). This study presents data on 24-month sustainability and factors that may have influenced sustainability of provider-delivered TUT.

**Methods:** We conducted post intervention qualitative interviews guided by CFIR (n=52), and 24-month surveys with health care providers (n=51) which assessed provider-delivered 3As+R using the same measures in the baseline, 6, 12, and 24-month surveys. Questions about factors influencing sustainability were adapted from the Program Sustainability Assessment Tool. We used a mixed methods analytic approach.

**Findings:** Adherence to all 3As (for half or more visits) significantly increased at 6 and again at 12-months, but by 24-months declined to 6-month levels or lower (e.g., Assist increased from 0% at baseline to 42% 6-months, 77% 12-months and 37% 24-months. Referral rates were 38% at 6, 70% at 12 and 18% at 24 months. Survey data were consistent with qualitative interviews noting that lack of ministry of health (MOH) prioritization of TUT, and therefore availability of ongoing funding and resources (e.g., ongoing training), and competing priorities, present barriers to sustainability. However, provider, VHW and community commitment to addressing this public health priority was noted as a factor motivating health centers to continue to deliver TUT.

**Implications for D&I Research:** In countries with a political and social context that is similar to Vietnam’s, early and ongoing stakeholder engagement with policymakers (i.e., MOH) is needed to align priorities and garner support to sustain effective strategies for implementing evidence-based TUT.

**Primary Funding Source**

National Institutes of Health

### S91 Organizational readiness to implement soil-transmitted helminth elimination programs: Results from a three-country hybrid study

#### Arianna Means^1^, Marie-Claire Gwayi-Chore^1^, Angel Titus^2^, Yesudoss Gnanapu^2^, Comlanvi Innocent Togbevi^3^, Félicien Chabi^3^, Euripide Avokpaho^3^, Adrian JF Luty^4^, Moudachirou Ibikounlé^5^, Sitara Ajjampur^2^, Dr. Bryan Weiner^1^, Judd Walson^1^, Kumudha Aruldas^2^

##### ^1^University of Washington, Seattle, WA, USA; ^2^Christian Medical College, Vellore, India; ^3^Institut de Recherche Clinique du Bénin, Abomey-Calavi, Benin; ^4^Université de Paris, Paris, France; ^5^Université d’Abomey-Calavi, Cotonou, Benin

###### **Correspondence:** Arianna Means (aerubin@u.washington.edu)

**Background:** Soil-transmitted helminths (STH), known as intestinal worms, affect over 1.5 billion people globally. STH control guidelines target school-age children with deworming drugs delivered via school-based mass drug administration (MDA). However, new evidence suggests that it may be possible to interrupt transmission of STH using community-based MDA that targets individuals of all ages. Launching community-wide MDA as standard practice would require substantial changes to national and global policies. We aimed to determine health system readiness to implement community-wide MDA in conjunction with the DeWorm3 project, a hybrid trial testing the feasibility of interrupting STH transmission in Benin, India, and Malawi.

**Methods:** We adapted the Organizational Readiness for Implementing Change tool by adding a ‘Capacity for Change’ module in which constructs for demonstrated capacity, organizational agility, and organizational structure were included. In each country, we sampled WHO officials, NGO partners, government personnel at all administrative levels, and community-level health workers who support delivery of the intervention. Survey questions were also organized by health system domains: policy environment, leadership structure, technical capacity, community-delivery infrastructure, financial, material, and human resources. Surveys utilized a 5-point Likert scale, with the addition of open-ended questions for follow-up. Mean readiness scores were summarized at module, construct, and domain levels, and disaggregated by stakeholder group.

**Findings:** Surveys were administered to 108, 74, and 69 stakeholders in Benin, India, and Malawi, respectively. In all countries, mean organizational readiness to implement community-wide MDA was high while items associated with organizational capacity to implement scored lower. The constructs ‘demonstrated capacity to deliver public health campaigns’ and ‘organizational agility’ scored particularly low. Across all sites, low mean readiness was observed in financial and human resources domains. However, within these two domains WHO and NGO-level stakeholders perceived higher readiness to implement compared to local government and health workers. These findings provide evidence regarding specific change management activities that could take place amongst targeted stakeholders prior to the roll out of STH elimination programs.

**Implications for D&I Research:** This study adapts a valid D&I tool for use in low-and-middle-income countries. This study also bridges research to policy by evaluating drivers of readiness ahead of potentially sweeping policy changes across STH-endemic countries.

**Primary Funding Source**

Bill & Melinda Gates Foundation

### S92 What makes an enabling context for mental health delivery? Differential implementation practices for task-shared delivery across education and health sectors in western Kenya

#### Prerna Martin^1^, Kathryn Whetten^2^, Christine Gray^2^, Rosemary Meza^1^, Caroline Soi^1^, Cyrilla Amanya^3^, Augustine Wasonga^3^, Shannon Dorsey^4^

##### ^1^University of Washington, Seattle, WA, USA; ^2^Duke University, Durham, NC, USA; ^3^ACE Africa Kenya, Bungoma, Kenya; ^4^Psychology, University of Washington, Seattle, WA, USA

###### **Correspondence:** Prerna Martin (prmartin@uw.edu)

**Background:** Research demonstrates effectiveness of mental health interventions delivered via task-sharing in low-resource settings; however, methods to scale and sustainably deliver interventions with minimal governmental funding are lacking. We examine implementation practices and policies (**IPPs**) associated with successful implementation of a cognitive behavioral therapy (**CBT**) in the health extension and education sectors in western Kenya.

**Methods:** As part of Step 1 of a large stepped-wedge cluster randomized trial, we implemented the CBT intervention in 10 sites in health extension and education (20 sites, 60 lay counselors; 257 youth). Teachers and Community Health Volunteers (**CHVs**) were trained and supervised by local, experienced lay counselors. Six sites per sector, selected on characteristics that could impact implementation (urban/rural, implementation leadership), participated in a mixed methods comparative case study using a rapid qualitative inquiry to understand IPPs associated with successful implementation (36 counselors; 12 leaders). Analyses would inform implementation coaching for subsequent sites (30 per sector).

**Findings:** Overlapping and sector-specific IPPs were identified. Both sectors highlighted the importance of training, supervision, resources, counselor recognition, and cross-sector collaboration. Counselors at sites with higher implementation leadership were more likely to report their own positive attitude and communication about CBT implementation. Workload adjustment was critical for education but not health (83% vs. 17%), where CHVs were less embedded in an organizational hierarchy. Teachers reporting more positive organizational climate were more likely to report receiving more implementation support from other teacher co-workers at their schools (75% vs. 33%). Incentives that facilitated CBT delivery were perceived differently across sectors. Teachers were more likely to report incentives of improved performance in their existing role (i.e., classroom-teacher) (72% vs. 44%) and receiving positive feedback from their leader (61% vs. 39%). CHVs—who are the health system’s extension into the community—highlighted incentives such as gifts/acknowledgment from community members more frequently (44% vs. 11%).

**Implications for D&I Research:** Implementation strategies for scale-up are needed. Our results provide information on differential IPPs related to implementation success in two child-relevant sectors. The rapid methods are replicable in other low-resource settings and can be used to inform tailored implementation support for task-shared interventions.

**Primary Funding Source**

National Institutes of Health

### S93 Ready for change? a cfir-driven mixed methods study of provider-level barriers and facilitators to adoption of evidence-based practice for cervical cancer screening in the Dominican Republic

#### Erica Liebermann (ejl472@nyu.edu)

##### New York University, New York, NY, USA

**Background:** High rates of cervical cancer persist in the Dominican Republic, despite the availability of Pap smear screening. Evidence-based guidelines suggest HPV testing for women over 30 every 5 years is the most effective screening strategy. Country-specific factors necessary for the successful integration of evidence-based guidelines in the Dominican Republic are not yet understood. In particular, it is not known what Dominican health care providers, who are women’s primary link to screening, know or think about current global recommendations for cervical cancer prevention. This study explored Dominican provider awareness of and attitudes towards evolving evidence-based practice (EBP) for cervical cancer screening.

**Methods:** This was a pre-adoption assessment guided by the Consolidated Framework for Implementation Research (CFIR) and the Theory of Planned Behavior (TPB). In this mixed methods study**,** 21 in-depth qualitative interviews were conducted and 202 surveys administered with providers in the Santo Domingo and Monte Plata provinces of the Dominican Republic. Qualitative and quantitative data were compared across CFIR constructs within the *inner setting*, *outer setting*, *characteristics of individuals* and *intervention characteristics* domains, and mixed methods findings categorized as provider-level barriers or facilitators to adoption of EBP for cervical cancer screening in the Dominican Republic.

**Findings:** Mixed methods findings indicated that barriers to adoption of EBP were limited provider knowledge of HPV testing for primary screening, and a strong belief that Dominican women needed to begin screening for cervical cancer at a younger age than is recommended by evidence-based guidelines. Facilitators to a change in screening practice were concern about high cervical cancer incidence and mortality in the Dominican Republic, a medical culture of evidence-driven practice, and provider receptivity to integration of HPV testing into all health sectors.

**Implications for D&I Research:** This study revealed important Dominican provider beliefs and practices that would affect the adoption of a change in screening practice. Findings suggest a demonstration project with HPV testing might generate necessary in-country data to gain acceptance from providers as well as health system leaders. Implementation frameworks and outcomes for such a study would elicit factors relevant to scale-up of a new screening strategy in the Dominican Republic and in low-and middle-income countries more broadly.

**Primary Funding Source**

New York University internal scholarships

### S94 Strengthening south African district hospitals with the implementation of a clostridioides difficile infection intervention: Understandings from the consolidated framework for implementation research

#### Laurel Legenza^1^, Renier Coetzee^2^, Kenneth Crombie^3^, Tasneem Esack^4^, Megan Mina^3^, Susanne Barnett^1^, Warren Rose^1^, Nasia Safdar^1^

##### ^1^University of Wisconsin-Madison, Madison, WI, USA; ^2^University of the Western Cape, Cape Town, South Africa; ^3^University of Cape Town, Cape Town, South Africa; ^4^Victoria Hospital, Wynberg, South Africa

###### **Correspondence:** Laurel Legenza (Legenza@wisc.edu)

**Background:***Clostridioides difficile* infection (CDI) is a global health threat and patients testing positive for *C. difficile* in South Africa have a high risk of mortality according to a recent epidemiology study. Substantial opportunities for improving CDI quality of care in low resourced hospitals exist. This study explores the implementation of a diarrhea alert CDI checklist intervention in South African government hospitals using the Consolidated Framework for Implementation Research (CFIR).

**Methods:** A CDI checklist was designed and implemented at three district level hospitals in the Western Cape, South Africa. CFIR was used as a framework to contextualize study findings, including a description of the implementation process and adaptations for each hospital. A mixed-methods approach was applied with quantitative outcomes data and qualitative interview and focus group data with front-line and administrative healthcare providers. Transcripts were coded to *a priori* workflow steps as well as to aspects of the CDI checklist and emerging themes.

**Findings:** While maintaining the intervention core elements, each hospital adapted the implementation process based on available resources. One hospital displayed high acceptance and uptake of the intervention compared to the two other hospitals. At the high-acceptance hospital, quality of care measures improved with use of the checklist compared to baseline data collected over one year (n=112 patients with positive *C. difficile* result). From qualitative analyses, factors that influenced acceptance and uptake included strong peer intervention champions, champions in influential leadership positions, strong managerial leadership, and consistent tertiary hospital and external academic affiliations. High staff turnover may have limited checklist uptake. Recommendations for refining the intervention are reducing checklist size, including additional risk factors, and coordination with concurrent programs and new staff training.

**Implications for D&I Research:** Further research is needed to explore readiness for adoption and implementation strategies in resource constrained settings without strong academic affiliation. Tools for assessing readiness for change should be evaluated for use in low-resource settings. The CDI checklist should be further adapted and evaluated for resource constrained settings, along with revisions from study findings in order to increase intervention sustainability and impact.

### S95 Promoting uptake of evidence-based care for acute coronary syndrome in Tanzania: A prospective mixed-methods study

#### Julian Hertz^1^, Francis Sakita^2^, Godfrey Kweka^3^, Gerald S. Bloomfield^1^, John Bartlett^4^, Janet Bettger^4^

##### ^1^Duke University School of Medicine, Durham, NC, USA; ^2^Kilimanjaro Christian Medical Centre, Moshi, Tanzania; ^3^Kilimanjaro Christian Research Institute, Moshi, Tanzania; ^4^Duke Global Health Institute, Duke University, Durham, NC, USA

###### **Correspondence:** Julian Hertz (julian.hertz@duke.edu)

**Background:** Acute coronary syndrome (ACS) is a leading cause of death worldwide. Despite the strength of evidence for global guidelines for ACS diagnosis and care, barriers to care remain, particularly in low-resource settings. The aims of this study were to describe current adoption of evidence-based practices for ACS care in northern Tanzania and to identify barriers to uptake.

**Methods:** An observational mixed-methods study was conducted in northern Tanzania. From August 2018 through January 2019, adult patients presenting to an emergency department (ED) with chest pain or shortness of breath were prospectively consecutively enrolled, and their diagnostic workups, treatments, and diagnoses were observed and recorded. Individual five-year risk of cardiovascular event was calculated using the Harvard National Health and Nutrition Examination Survey risk score. Semi-structured in-depth interviews were concurrently conducted with emergency and outpatient department providers in northern Tanzania to explore barriers to ACS diagnosis and care. Thematic analysis of interview transcripts was accomplished via an iterative cycle of coding and consensus building.

**Findings:** Of 339 patients presenting with chest pain or shortness of breath, median (IQR) age was 60 (46-72) years, and 222 (65.5%) had >10% five-year risk of a cardiovascular event. Of participants, 170 (50.1%) underwent ECG testing, 9 (2.7%) underwent cardiac biomarker testing, 6 (1.8%) were diagnosed with ACS, and 3 (0.9%) received aspirin. Patients with >10% five-year risk of cardiovascular event were not more likely to receive an ECG than lower-risk patients (OR 0.89, 95% CI 0.56-1.39, *p*=0.595). Thematic analysis of interview data revealed patient-related, provider-related, and systems-related barriers to ACS care. Patient-related barriers included poor knowledge of ACS and inappropriate healthcare-seeking behavior. Provider-related barriers included inadequate training regarding ACS and inconsistent application of knowledge to the clinical context. Systems-related barriers included lack of data about disease burden, lack of guidelines and protocols, cost of care, and insufficient diagnostic equipment.

**Implications for D&I Research:** In northern Tanzania, current uptake of evidence-based strategies for diagnosis and care of ACS is challenged by modifiable patient-, provider-, and systems-related factors. Ongoing research leverages data-driven and stakeholder-prioritized strategies in a hybrid implementation-effectiveness study to improve ACS care and outcomes.

**Primary Funding Source**

National Institutes of Health

### S96 The 4 youth by youth crowdsourcing contest: Using participatory design to reach and increase uptake of HIV self-testing among young people in Nigeria.

#### Juliet Iwelunmor^1^, Oliver Ezechi^2^, Chisom Obiezu-Umeh^1^, Titi Gbaja-biamila^2^, Ucheoma Nwaozuru^1^, David Oladele^2^, Florida Uzoaru^1^, Collins Airhihenbuwa^3^, Bill Kapogiannis^4^, Joseph Tucker^5^

##### ^1^Saint Louis University, St. Louis, MO, USA; ^2^Nigerian Institute of Medical Research, Yaba, Lagos, Nigeria; ^3^Georgia State University, Atlanta, GA, USA; ^4^*Eunice Kennedy Shriver* National Institute of Child Health and Human Development, National Institutes of Health, Bethesda, MD, USA; ^5^University of North Carolina at Chapel Hill, Chapel Hill,, NC, USA

###### **Correspondence:** Juliet Iwelunmor (iwelunmorj@slu.edu)

**Background:** Often conventional, expert-driven interventions are pushed out to youth populations, assuming behavior change will occur regardless of fit. Bottom-up, participatory approaches such as crowdsourcing may increase uptake of HIV self-testing. We used crowdsourcing to develop a youth-led HIV self-testing program for Nigerian youth.

**Methods:** We define crowdsourcing as having a group solve a problem and then sharing with the public. Our crowdsourcing project occurred in three phases. First, we conducted a nationwide open idea challenge contest where we solicited concepts and images promoting HIV self-testing over a 7-week period. Next, we conducted a 48 hour designathon in which youth teams were invited to create prototype solutions for increasing uptake of HIV self-testing. Finally, during our 4-week bootcamp, a select group of teams refined and finalized their prototypes for expanding HIV self-tests. All contests were judged by an expert panel of judges consisting of local professionals in public health, communications, civil society, and design. Evaluation criteria included the following: desirability to young people, feasibility to implement, and potential for impact.

**Findings:** During the idea challenge, a total of 903 entries were received. The top 42 entrants pitched their solutions to the judges with top three entries advancing to the designathon. During the designathon, a total of 127 team entries were received, of which 13 teams were invited to create prototype implementation strategies for increasing reach and uptake of HIV self-tests. The top seven teams advanced to the 4-week bootcamp where they learnt skills, received mentorship and tailored feedback, and finalized their prototype solutions and implementation strategies. The top three teams received monetary awards and HIV self-testing kits to support their project’s future implementation and evaluation.

**Implications for D&I Research:** Crowdsourcing contests are a useful and feasible methodology for engaging young people in the design and development of implementation strategies to increase uptake of HIV self-testing. The process generated significant engagement and tapped into the creativity and potential of young people to lead solutions and strategies for expanding HIV self-testing uptake in Nigeria. Further research evaluating ideas is underway and may pave way for implementation research targeting youth populations.

**Primary Funding Source**

National Institutes of Health

### S97 Use of human-centered design to adapt a novel tuberculosis digital adherence technology to the local context

#### Devika Patel^1^, Christopher Berger^1^, Joseph Ggita^2^, Alex Kityamuwesi^2^, Joshua Feler^3^, Kathryn Neville^4^, Lara Chehab^1^, Achilles Katamba^5^, Adithya Cattamanchi^6^, Amanda Sammann^1^

##### ^1^University of California, San Francisco, San Francisco, CA, USA; ^2^Uganda Tuberculosis Implementation Research Consortium, Kampala, Uganda; ^3^Yale University School of Medicine, New Haven, CT, USA; ^4^Stanford University, Stanford, CA, USA; ^5^Makerere College of Health Sciences, Kampala, Uganda; ^6^University of California San Francisco, San Francisco, CA, USA

###### **Correspondence:** Devika Patel (devika.patel@ucsf.edu)

**Background:** Tuberculosis (TB) treatment success rates in high burden countries remain far below the World Health Organization’s (WHO) 90% target. 99DOTS (Everwell Health Solutions, India) is a low-cost digital adherence technology where patients call toll-free phone numbers hidden underneath pills in blister packs to self-report medication dosing. The resulting real-time adherence data enables targeted patient follow-up. As part of its first deployment in sub-Saharan Africa, we used human-centered design (HCD) methodology to improve fit to the local context.

**Methods:** Utilizing HCD methodology, we performed 46 semi-structured interviews at eight TB treatment units in Uganda. Interviewees included TB patients, family members, clinicians, nurses, community health workers, and local community leaders. Key quotes and themes were elicited from transcribed interviews and translated into actionable insights that guided 99DOTS adaptation.

**Findings:** We discovered seven main insights: education about TB is an essential and strongly desired component; gamification, entertainment, celebrities and politicians are not motivators; health workers are trusted members of patients’ TB journeys; food was a major adherence barrier; pill packs had the potential to serve as adherence tools; the stigma surrounding TB is deeply pervasive; and patients want to feel gratitude and celebration. These insights led to the physical and visual redesign of the pill pack envelope transforming it into a booklet concealing the pills, space on the pack to record their health worker’s contact information, pictographic instructions on its use, and a selection of educational stickers to allow the patient to customize the pill pack. Text messages and audio recordings by local health workers were designed to emphasize TB education and motivational messages during patients’ daily phone calls.

**Implications for D&I Research:** The human-centered design methodology emphasizes empathy with end-users, which ensures that technology such as 99DOTS is adapted to fit the appropriate cultural and local context. This intervention is currently being implemented with over 600 patients in Uganda and its adoption and implementation are under evaluation. HCD is a powerful tool to bolster and complement dissemination and implementation research endeavors.

**Primary Funding Source**

TB Reach

### S98 Implementing mHealth interventions in a resource-constrained setting: A case study from Uganda

#### Amanda Meyer^1^, Mari Armstrong-Hough^2^, Diana Babirye^3^, David Mark^3^, Patricia Turimumahoro^3^, Irene Ayakaka^4^, Jessica Haberer^5^, Achilles Katamba^4^, J. Lucian Davis^1^

##### ^1^Yale School of Public Health, New Haven, CT, USA; ^2^New York University, New York, NY, USA; ^3^Uganda Tuberculosis Implementation Research Consortium, Kampala, Uganda; ^4^Makerere College of Health Sciences, Kampala, Uganda; ^5^Massachusetts General Hospital, Boston, MA, USA

###### **Correspondence:** Amanda Meyer (amanda.meyer@yale.edu)

**Background:** Mobile health (mHealth) interventions are becoming more common in low-income countries. Existing research often overlooks implementation challenges associated with technological requirements and design of mHealth interventions. We therefore sought to describe the process of implementing a complex mHealth intervention in Uganda, to classify its key challenges.

**Methods:** We created a mobile survey application to facilitate a household-randomized, controlled trial of home-based TB contact investigation. We incorporated digital fingerprinting for patient identification in both study arms, and automated SMS messages in the intervention arm only. A local research team systematically documented challenges to implementation in biweekly site visit reports, project management reports, and biweekly conference call minutes. We then used the Consolidated Framework for Implementation Research (CFIR) to identify *construct*s where key challenges to implementation were experienced.

**Findings:** Using CFIR, we identified challenges in three principal domains: (1) intervention characteristics, (2) inner setting, and (3) characteristics of individuals implementing the intervention. The *adaptability* of the application to the local setting was limited by software and hardware requirements. The *complexity* and logistics of implementing the intervention further hindered its adaptability. Study staff reported that health workers (HWs) were enthusiastic regarding the use of technology to enhance TB contact investigation during trainings and initial phases of intervention implementation. After experiencing technological issues, their trust in the technology declined along with their use of it. Finally, the development and execution of a data management plan that would allow for articulation of *goals* and provide timely *feedback* to study staff, HWs, and participants was impeded by complex data structures. Together, these issues made it difficult to measure fidelity in real-time.

**Implications for D&I Research:** mHealth technologies have potential to make delivery of public health interventions more direct and efficient. However, challenges uniquely posed by technology such as the lack of real-time adaptability, challenging feedback systems and complex data structures should be examined and addressed, especially in low-resource settings where IT services have not yet proliferated. Innovative ways of minimizing these challenges using implementation strategies are needed to ensure technological advancement ultimately improves patient care rather than disrupts it.

**Primary Funding Source**

National Institutes of Health

### S99 A feasibility and acceptability study of an mhealth platform and integrated wearable digital sensors to improve the implementation of manualized psychological treatment for underserved adolescent mothers

#### Ashley Hagaman^1^, Alastair van Heerdan^2^, Anubhuti Poudyal^3^, Prabin Byanjankar^4^, Sujen Man Maharjan^4^, Prasansa Subba^4^, Brandon Kohrt^3^

##### ^1^Yale University, New Haven, CT, USA; ^2^Human Sciences Research Council, Pietermaritzburg, South Africa; ^3^George Washington University, Washington DC, DC, USA; ^4^Transcultural Psychosocial Organization-Nepal, Kathmandu, Nepal

###### **Correspondence:** Ashley Hagaman (ashley.hagaman@yale.edu)

**Background:** There is high prevalence of untreated postpartum depression among adolescent mothers, and women in low- and middle-income countries (LMICs) have the least access to care. LMICs have begun to implement non-specialist delivered manualized interventions to treat depression, but the evidence for their sustainability and scalability is inconsistent. Passive sensing data generated from wearable digital devices has great potential to optimize effectiveness at scale and improve intervention outcomes for postpartum depression. We developed StandStrong, a platform that aggregates and visualizes passive sensing data (GPS, physical activity, machine-coded episodic audio recordings, proximity to infant) to enhance depression treatment delivery.

**Methods:** This implementation study explored (a) the feasibility and acceptability of using wearable digital sensors with young women and their families in an under-served LMIC setting and (b) the barriers and facilitators for non-specialist integration of this data into a phone-based application to personalize psychological treatment delivery. Leveraging a community-based participatory mixed-methods design and CFIR (consolidated framework for implementation research), in-depth interviews, focus group discussions, and observations were conducted among 46 mothers, 2 focus group discussions with non-specialist health care providers (n=13), and 4 community-counselors across 120 StandStrong delivered depression treatment sessions in rural Nepal.

**Findings:** Individual and family level barriers included difficulty carrying the phone throughout the day, family privacy concerns, fear of device loss/damage, and uncertainty about possible health effects of Bluetooth proximity monitoring. Mothers highlighted that continuous knowledge of their own behaviors (movement/activity) and receiving information about their infant were important facilitators. Counseling facilitators included the ability to: review passive data with patients during sessions to help recall, use data visualizations to encourage patient behavioral activation, and access data for detailed tracking of patient progress between sessions. Community-counselors noted inconsistent data capture as the primary barrier to implementation at scale.

**Implications for D&I Research:** StandStrong presents a novel, patient-centered implementation strategy for enhanced evidence-based manualized treatment for postpartum depression. This research strengthens our understanding of the role of context at multiple levels in LMIC to better adapt and enhance manualized evidence-based interventions to deliver more personalized care across cultural contexts. We also demonstrate the feasibility of mhealth technologies to improve global D&I work.

**Primary Funding Source**

Bill & Melinda Gates Foundation

## Health Policy Dissemination and Implementation Science

### S100 Mixed-methods evaluation of implementation of an opt-out tobacco treatment service at a safety-net hospital to improve tobacco treatment performance metrics

#### Renda Wiener^1,2^, Nicole Herbst^1^, Bhavna Seth^1^, Katia Oleinik^1^, Hasmeena Kathuria^1^

##### ^1^Boston University School of Medicine, Boston, MA, USA; ^2^Edith Nourse Rogers Memorial Veterans Hospital, Veterans Health Administration, Bedford, MA, USA

###### **Correspondence:** Renda Wiener (rwiener@bu.edu)

**Background:** In response to a state-level incentive program, our safety-net hospital sought to improve Joint Commission metrics for inpatient (TOB-2) and post-discharge (TOB-3) tobacco treatment. We developed an intervention consisting of an “opt-out” electronic health record-based Best Practice Alert (BPA)+order-set, which triggered consultation to the inpatient Tobacco Treatment Service (TTS) for all hospitalized smokers, regardless of motivation to quit.

**Methods:** We performed a sequential explanatory mixed methods study to evaluate implementation of the TTS. Using a quasi-experimental pragmatic design and multivariable logistic regression, we first examined effectiveness at improving performance metrics, comparing inpatient and post-discharge tobacco treatment among smokers seen (n=505) versus not seen by the TTS due to time constraints (n=680) in the 6 months after implementation (7/1/16-12/31/16). We next examined adoption of intervention components by clinicians over the first year (7/1/16-6/30/17), calculating the proportion of “current smoker” admissions whose clinicians accepted TTS consultation (“signed off on” order set), and follow-through of TTS recommendations for tobacco treatment. Finally, we conducted qualitative interviews with inpatient clinicians (n=25) to understand their rationale for adoption or non-use of intervention components.

**Findings:** Effectiveness in first 6 months: Smokers seen by the TTS had higher rates of tobacco treatment than those not seen, both in the inpatient (TOB-2: 51.5% [260/505] vs 35.9% [244/680], AOR 1.9, 95% CI 1.5-2.42) and post-discharge (TOB-3: 32.5% [164/505] vs 12.4% [84/680], AOR 3.38, 95% CI 2.51-4.57) settings. Adoption over first year: Among all current smoker admissions for whom the BPA fired, clinicians accepted TTS consultation on 62.1% (4100/6598), with acceptance ranging from 8% to 82.2% across clinical services (p<.00001). Among patients seen by the TTS who desired tobacco treatment, the primary inpatient team ordered treatment consistent with TTS recommendations for 82.5% during hospitalization, but for only 48.8% at discharge. Clinicians expressed that they valued the TTS, but that BPA fatigue, time constraints, competing priorities, and poor communication were barriers to consulting the TTS and following recommendations.

**Implications for D&I Research:** Implementing an “opt-out” inpatient tobacco treatment service at a safety-net hospital is feasible, acceptable, and effective at improving performance on quality metrics. Further adaptation to increase clinician adoption of team recommendations must address multiple clinician barriers.

**Primary Funding Source**

Local grant from Boston University Evans Center for Implementation & Improvement Sciences

### S101 A decision sciences approach to investigate evidence use in trauma screening for children entering foster care

#### Thomas Mackie^1^, Ana Cotroneo^1^, Brittany Fishman^1^, Laurel Leslie^2^, Justeen Hyde^3^, Radley Sheldrick^4^

##### ^1^Rutgers University School of Public Health, Piscataway, NJ, USA; ^2^Tufts Medical Center, Boston, MA, USA; ^3^VA HSR&D Center for Healthcare Organization and Implementation Research (CHOIR), Veterans Health Administration, Bedford, MA, USA; ^4^Boston University School of Public Health, Boston, MA, USA

###### **Correspondence:** Thomas Mackie (tmackie@ifh.rutgers.edu)

**Background:** Calls have been made for greater application of the decision sciences to expedite research evidence use by public sector decision-makers in mental health policy. The decision sciences draw particular attention to the “bounded rationality” of decision-makers, investigating the consequences of imperfect information, limited human information processing abilities, and heuristics that decision-makers use to expedite decision-making. Our study investigates how decisions are made by mid-level managers in Medicaid, child welfare, and mental health systems who seek to develop protocols that routinely identify trauma among children entering foster care. This paper aims to identify case-specific insights into decision-making for screening protocols as well as to explore the utility of the decision sciences in studies of research evidence use.

**Methods:** Our study used a “decision sampling” approach, in which respondents (n=85) from 11 states identified a recent decision that sought to identify and address the trauma of children entering foster care. We anchored key informants’ responses on the recent index decision to minimize recall and response bias and potential desirability of evidence use. Informed by the "decision-making ecology," our study first investigated the decision-continuum specifically seeking to understand the series of related decisions that were necessary to address the index decision on trauma screening and assessment (n=23). We then inquired on the factors perceived as influential to the decision-making process.

**Findings:** The decision continuum identified by respondents included 17 decision points across five domains, including (1) the reach of the screening protocol, (2) content of the tool, (3) threshold for referral, (4) workforce development requirements, and the (5) system capacity to respond. Across the decision continuum, factors identified as influential to decision-making arose from four domains: (1) the sociopolitical context, (2) organizational absorptive capacity, (3) decision-making structure/decision-makers, and (4) the case, including clinical considerations and access to and content of diverse information sources, ranging from local knowledge to global evidence.

**Implications for D&I Research:** Opportunities exist to integrate decision sciences into studies of research evidence use. Our application, employing the decision-making ecology to policy decisions, facilitated an in-depth characterization of the decisions required and characterization of the factors influential and relevant trade-offs.

**Primary Funding Source**

W.T. Grant Foundation

### S102 A pragmatic method for costing implementation strategies using the time-driven activity-based costing

#### Zuleyha Cidav^1^, Jeff Pyne^2^, David Mandell^3^, Rinad Beidas^3^, Geoffrey Curran^4^, Jennifer Mautone^5^, Ricardo Eiraldi^5^, Steven Marcus^3^

##### ^1^University of Pennsylvania School of Medicine, Philadelphia, PA, USA; ^2^University of Arkansas for Medical Sciences, Little Rock, AR, USA; ^3^University of Pennsylvania, Philadelphia, PA, USA; ^4^Central Arkansas Veterans Healthcare System, Little Rock, AR, USA; ^5^The Children's Hospital of Philadelphia, Philadelphia, PA, USA

###### **Correspondence:** Zuleyha Cidav (zcidav@upenn.edu)

**Background:** Strategies to implement evidence-based practices consume scarce resources and incur costs. Although critical for decision makers with constrained budgets and limited resources, such resource use and cost information are not typically reported. This is at least partly due to a lack of clearly defined and standardized costing methods for use in implementation science. This study presents a pragmatic approach to systemically estimating resource use and costs of implementation strategies using a well-established business accounting system. The method is demonstrated by estimating the first-year implementation costs of a group-based cognitive behavioral therapy program for students with externalizing disorders in six Philadelphia schools.

**Methods:** Time-driven activity-based costing (TDABC) is combined with the existing guidelines forimplementation strategy specification and reporting. Implementation protocol, measures, project notes and key personnel interviews were used to map the implementation process by specifying the strategies with their actors, specific action steps, temporality, and dose. The dose is defined for each action step as the person-hours invested in its completion, and accounts both for frequency and intensity of the action step. Implementation strategy dose is the sum of person-hours on each action step that constitute the strategy. Project resources are identified, andthe price per unit person-hour is calculated as per the TDABC. Costs of action steps, strategies and implementation project is reported from a payer perspective.

**Findings:** Estimated total cost was $63,842; $10,640 per school. The largest cost incurred was for the communication efforts ($30,691), which involved in-person meetings, phone calls, and email exchanges. Next largest costs were for the stakeholder engagement, consultation/coaching, and supervision, which comprised 19%, 15%, 12% of total costs, respectively. Assessment/evaluation and training constituted the smallest costs, at 4% and 3% respectively.

**Implications for D&I Research:** This method allows for inclusion of implementation costs in the efforts of strategy specification, tracking and reporting. It serves as a pragmatic tool to operationalize the conduct of the implementation activities, track the resources consumed and estimate associated costs. It could facilitate the routine incorporation of cost analysis and economic evaluations into implementation research. It provides granular cost information which could be used to identify and address the inefficiencies in the implementation process.

### S103 Scaling beyond early adopters: A systematic review and key informant perspectives

#### Isomi Miake-Lye^1^, Selene Mak^2^, Christine Lam^3^, Anne Lambert-Kerzner^4^, Deborah Delevan^5^, Tanya Olmos^6^, Paul Shekelle^6^

##### ^1^Evidence-based Syntheisis Program, VA Greater Los Angeles Healthcare System, Veterans Health Administration, Los Angeles, CA, USA; ^2^VA Greater Los Angeles Healthcare System, Evidence-based Syntheisis Program, Veterans Health Administration, Los Angeles, CA, USA; ^3^Center for the Study of Healthcare Innovation, Implementation & Policy, VA Greater Los Angeles Healthcare System, Veterans Health Administration, Sepulveda, CA, USA; ^4^University of Colorado - Denver, Denver, CO, USA; ^5^Care Coordination QUERI, VA Greater Los Angeles Healthcare System, Veterans Health Administration, Los Angeles, CA, USA; ^6^VA Greater Los Angeles Healthcare System, Veterans Health Administration, Los Angeles, CA, USA

###### **Correspondence:** Isomi Miake-Lye (isomi.miake-lye@va.gov)

**Background:** While innovations and improvements in care delivery are continuously available, they are often not spread across all settings that would benefit from their uptake. We conducted a systematic review and key informant interviews to describe the process of scale up and spread clinical and administrative practices, with a focus on “hard-to-engage” sites.

**Methods:** For the systematic review component, we searched for literature relevant to large-magnitude scale up and spread. We augmented the traditional systematic review with key informant interviews. We identified eight leads of large-magnitude scale up and spread projects in VA. They participated in semi-structured interviews to share their perspectives on and experiences with strategies to scale up and spread clinical and administrative practices across healthcare systems. Drawing primarily on matrix analysis, each interview was analyzed by three members of the team and consistency of interpretation was regularly checked through team discussion.

**Findings:** Our searches identified 1,919 titles for review, of which 52 articles were included. Seven discussed strategies for hard-to-engage sites, 11 described hard-to-engage sites, and 34 discussed general scale up and spread strategies. These included publications were narratively synthesized and combined with interview findings to describe breaking down the national scale up or spread process, macro models of organization or infrastructure of spread efforts, common challenges for spreading to hard-to-engage sites, potential benefits of working with hard-to-engage sites, and useful strategies for hard-to-engage site.

**Implications for D&I Research:** Little published evidence has been identified that focuses on or provides discussion of strategies for reaching those sites that may be hard-to-engage. Future work could focus on better documentation of the later stages of spread efforts, including specific approaches and strategies used to engage hard-to-engage sites.

**Primary Funding Source**

Department of Veterans Affairs

### S104 Family impact seminars: A model for communicating research to state policymakers

#### Elizabeth Day^1^, Karen Bogenschneider^2^

##### ^1^Cornell University, Ithaca, NY, USA; ^2^University of Wisconsin-Madison, Madison, WI, USA

###### **Correspondence:** Elizabeth Day (ead255@cornell.edu)

**Background:** When it comes to public policy, university research is considered more trustworthy than research from advocacy groups and think tanks. Yet university research is widely perceived as playing too small a role in public policy. The state Family Impact Seminar model is an approach designed to increase respect for and use of research evidence in policymaking, provide nonpartisan opportunities for legislators to engage in open dialogue for fostering relationships and finding common ground, and encourage legislators to examine policies and programs through the lens of family impact.

**Methods:** Since 1999, over 225 seminars have been held in more than 20 states on family issues, broadly defined to include health care, jobs, education, and corrections. A common survey protocol is administered at every seminar, and several additional evaluations have assessed whether the seminars achieve their goals, including phone interviews after seminars.

**Findings:** On seminar evaluations, legislators report using information to share with colleagues, incorporate into speeches and discussions, and develop or evaluate legislation. For example, in Oregon, following the first seminar, a refundable child-care tax credit was adopted, which the legislative sponsor attributed, in part, to information provided by the seminar; in Nebraska, after a seminar on rising health-care costs, a state Children’s Health Insurance Program law was passed and in 2009, more than 48,000 children were served. Additionally, in follow-up evaluations (phone interviews with 18 legislators 3-5 months post-seminar; 69% response rate) of a Wisconsin seminar on the science of early brain development, 83% of legislators reported that because of the seminar, they were “quite a bit” more likely to see the practical value of research; 72% of legislators reported that because of the seminars they were “quite a bit” more likely to view researchers as approachable; and 50% said they were “quite a bit” more likely to get to know their colleagues on the other side of the aisle.

**Implications for D&I Research:** Several best practices will be discussed that make the Family Impact Seminars an effective model for communicating rigorous, nonpartisan research to policymakers.

**Primary Funding Source**

William T. Grant Foundation

### S105 Federal use of research evidence: The research-to-policy collaboration model

#### Lawrie Green^1^, Max Crowley^2^, Taylor Scott^1^

##### ^1^Pennsylvania State University, University Park, PA, USA; ^2^Pennsylvania State University, State College, PA, USA

###### **Correspondence:** Lawrie Green (lawrie.green@psu.edu)

**Background:** Although most social science research is intended to produce knowledge for public benefit, a persistent gap between empirical knowledge and decisions made in practice and policy involves a complex set of challenges for which there are no simple solutions. Effective research translation necessarily involves coordinating a comprehensive array of strategies that support research use by decision-makers.

**Methods:** We will present on a model known as the Research-to-Policy Collaboration for (1) identifying legislative needs for scientific research evidence, (2) building researcher networks capacity to rapidly respond to those needs, and (3) facilitate effective sustained collaboration between policy and research communities. This includes discussion of a completed pilot trial (2016-2018) and ongoing randomized controlled trial for evaluating the efficacy of this model at scale (2019). Lessons learned for conducting research on improving the use of research in legislative contexts will be shared.

**Findings:** Pilot findings revealed that this model can successfully mobilize prevention scientists, engage legislative offices, connect policymakers and experts in prevention, and elicit congressional requests for evidence on effective prevention strategies. On average, the RPC model costs $3,510 to implement per legislative office. The RPC can elicit requests for evidence at an average cost of $444 per request. Researcher participating in the model report greater efficacy for engaging with legislative audiences around translating research for use in policy.

**Implications for D&I Research:** This project signals that the use of scientific knowledge of prevention in policymaking can be greatly augmented through strategic investment in translational efforts. Ultimately, this model seeks to accelerate the dissemination of scientific evidence among legislative audiences. Opportunities for optimizing project elements, and plans for future work are discussed.

**Primary Funding Source**

William T. Grant Foundation

### S106 Building the capacity of patient advocacy organizations to broker research evidence in health policymaking

#### Itzhak Yanovitzky^1^, Matthew Weber^2^

##### ^1^Communication, Rutgers University, New Brunswick, NJ, USA; ^2^Hubbard School of Journalism and Mass Communication, University of Minnesota, Minneapolis, MN, USA

###### **Correspondence:** Itzhak Yanovitzky (itzhak@rutgers.edu)

**Background**: Patient advocacy groups are well-positioned to broker relevant research into state and federal health policymaking given their mission, resources, and established relationships with policymakers. While these organizations frequently use research to draw attention to problems affecting their constituents and advocating for sensible solutions, they can also be effective brokers of research that informs policy implementation. We report preliminary findings from a project that assesses this research brokering potential of mental health policy advocacy groups in the context of improving access to screening for adolescent depression in New Jersey’s public schools.

**Methods**: A combination of data sources is used to assess the knowledge brokering potential of mental health advocates statewide: (1) content analysis of policy documents (transcripts of legislative bills and hearings, state government documents, and taskforce reports on the topic of adolescent depression from 2010-2019); (2) content analysis of local and national news reports on the topic (2010-2019); (3) analysis of web and social media content produced by mental health advocacy groups; (4) key-informant interviews with policy directors of mental health advocacy organizations active on this issue; and (5) key-informant interviews with state legislators who are working towards universal access to adolescent depression screening.

**Findings**: Data analysis is ongoing, but preliminary findings suggest that brokering of research evidence by mental health advocacy groups is limited by the availability of relevant research. In particular, policymakers are interested in research regarding potential barriers and facilitators to implementation of universal screening for adolescent depression in schools to reduce their ambiguity regarding the feasibility of implementation and alleviate concerns about unintended effects, but such research is not readily available. Mental health advocates are frequently called upon to provide implementation-relevant insights, but they are mostly able to provide anecdotal evidence that cannot inform sound policy.

**Implications for D&I Research:** This 3-year project will test the hypothesis that equipping mental health advocates with research that is directly relevant to implementation of the proposed policy, will facilitate their ability to advocate for evidence-based policy solutions and subsequently enable policymakers to make progress regarding response to the growing problem of adolescent depression.

**Primary Funding Source**

William T. Grant Foundation

### S107 An implementation intervention for school sun safety policies increased parents’ reports of sun safety communication from schools and children’s sun protection.

#### David Buller^1^, Kim Reynolds^2^, Mary Buller^1^, Richard Meenan^3^, Jeff Ashley^4^, Julia Berteletti^1^, Kim Massie^2^

##### ^1^Klein Buendel, Golden, CO, USA; ^2^School of Community and Global Health, Claremont Graduate University School of Community and Global Health, Claremont, CA, USA; ^3^The Center for Health Research, Kaiser Permanente Northwest, Portland, OR, USA; ^4^Dermatology, Sun Safety for Kids, Burbank, CA, USA

###### **Correspondence:** David Buller (dbuller@kleinbuendel.com)

**Background:** The CDC and Surgeon General have called on the nation’s schools to help prevent skin cancer by implementing sun safety in educational settings and school policies. We conducted a trial evaluating an intervention for supporting implementation of school district sun safety policy. Parents were surveyed to test the hypothesis that parents will report more communication about sun safety at schools receiving the intervention than at control schools.

**Methods:** Elementary schools (n=118) were enrolled in a pretest-posttest randomized controlled design (mean [M] =564.6 students/school; M=54.5% Hispanic students). All schools were in California school districts that had adopted a sun safety policy, Board Policy 5141.7. Schools randomized to the Sun Safe Schools (SSS) intervention group (N=58) received support for implementing school sun safety practices by trained coaches over 20 months. Based on Diffusion of Innovations Theory, support and resources for implementation were tailored to school principals’ readiness to implement. Parents completed an online posttest survey through invitations sent by principals or while attending parent-oriented school events.

**Findings:** The SSS intervention appeared to increase communication on sun safety and the use of student sun protection. Parents in intervention schools were more likely to report receiving information about sun safety from the school (M=26.3%, sd=3.1%, p=0.017) and that their children wore sun-protective clothing (frequency rating M=2.93, sd=0.03, p=0.033) than in control schools (M=18.0%, sd=2.5%; M=2.83, sd=0.03, respectively). The improvement in sun safety appeared to result from policy implementation. In schools where principals reported implementing sun safety practices at posttest, parents reported that children spent less time outdoors (M=14.8 hours, sd=0.25, p=0.033) and had fewer sunburns (mean proportion=0.13, sd=0.01, p=0.009) than at non-implementing schools (M=16.3 hours, sd=0.67; M=0.21, sd=0.04, respectively). Further, parents who received information about sun safety from the school (M=3.08, sd=0.04, p=0.008) reported more sun protection for their child than parents not receiving the information (M=2.96, sd=0.02).

**Implications for D&I Research:** A school district-level policy, combined with active technical support for schools within the district, appears effective at increasing implementation of school sun safety practices to help protect children from solar ultraviolet radiation, the primary risk factor for skin cancer.

**Primary Funding Source**

National Institutes of Health

### S108 Implementation of financial incentives in the federal supplemental nutrition assistance program (SNAP): Comparison across settings

#### Pamela Hull^1^, Rebecca Selove^2^, Jaimie Shing^1^, Kemberlee Bonnet^1^, Joscelyn Silsby^3^, Caprice Brown^3^, David Schlundt^1^

##### ^1^Vanderbilt University, Nashville, TN, USA; ^2^Tennessee State University, Nashville, TN, USA; ^3^AARP Foundation, Washington, DC, USA

###### **Correspondence:** Pamela Hull (pam.hull@vumc.org)

**Background:** Financial incentives have been shown to increase the purchase of fresh fruits and vegetables (FFVs) among low-income adults, but little is known about the implementation of FFV incentive policies through the federal Supplemental Nutrition Assistance Program (SNAP) in diverse real-world settings. This study used RE-AIM to evaluate implementation of the Fresh Savings incentive program, a large-scale demonstration project funded by USDA, to compare two incentive models across four settings.

**Methods:** AARP Foundation implemented Fresh Savings for SNAP participants in Tennessee and Mississippi from September 2015 to March 2019 in 27 Kroger grocery stores, 44 farmers’ markets, 8 produce stands, and 4 small grocers. Both incentive models depended on a qualifying SNAP purchase with either: 1) Paper coupons offering a 50% discount on FFV at Kroger stores (valued up to $10), or 2) dollar-for-dollar matched tokens up to $20 in value per week, available for immediate use. Transaction data were analyzed using joinpoint and interrupted time series analysis to examine trends, in addition to qualitative assessment of program adaptations.

**Findings:** Fresh Savings reached approximately 250,000 unique SNAP households during 43 months. Comparing across settings, adoption decreased as reach increased. Specifically, adoption ranged from 97% redemption of 7,624 tokens worth $100,737 in produce stands, and 95% redemption of 18,834 tokens worth $241,982 in farmers’ markets, to 80% redemption of 22,781 tokens worth $326,983 in small grocers, and 18% redemption of 1,971,942 printed grocery coupons worth $2,150,489 in Kroger stores. For Kroger, repeated exposure increased significantly over time, from 0.14 to 3.83 coupons printed per SNAP shopper per month. In markets, 75% of SNAP shoppers redeeming tokens were repeat shoppers. Analyses identified significant improvements in implementation outcomes in response to program adaptations over time, in particular, changes to the qualifying purchase requirement.

**Implications for D&I Research:** Incentives offered in grocery stores reached more SNAP customers, although paper coupons that would typically be saved for subsequent shopping trips had lower redemption rates than immediately useable tokens in varied market settings. Future research should examine consumer behavior and burden of implementation strategy options when planning or evaluating SNAP financial incentives for FFVs in various settings.

**Primary Funding Source**

USDA

### S109 Implementation of evidence based practices for substance use disorders in juvenile justice settings: Evaluating costs across implementation phases

#### Kathryn McCollister^1^, Diana Bowser^2^

##### ^1^University of Miami Miller School of Medicine, Miami, FL, USA; ^2^Brandeis University, Waltham, MA, USA

###### **Correspondence:** Kathryn McCollister (kmccolli@miami.edu)

**Background:** The juvenile justice system remains siloed from the behavioral health system resulting in significant unmet need for treatment among justice involved youth. Implementation of integrated strategies for improving access to treatment requires evidence of the costs of changing existing practices, which could result from adding new services, discontinuing treatment protocols that are not effective, or expanding existing services. To promote integration across different systems, stakeholders need to be aware of what types of cost investments (e.g., personnel, facilities, data systems) lead to more efficient implementation and better outcomes.

**Methods:** A detailed cost analysis was conducted alongside the Juvenile Justice Translational Research on Interventions for Adolescents in the Legal System (JJ-TRIALS), a cooperative implementation research initiative funded by the National Institute on Drug Abuse. JJ-TRIALS features two randomly assigned implementation interventions, Core and Enhanced, which are focused on improving screening, assessment, referral, and linkage to behavioral health services among juvenile justice youth with SUD. Cost data were collected prospectively throughout the study, including a careful accounting of existing resources reallocation to support JJ-TRIALS activities during Baseline, Early Experiment, and Late Experiment implementation phases.

**Findings:** During Baseline (i.e., pre-implementation and pre-randomization), the average cost incurred by participating agencies was $9,490 (range: $3,245 to $18,029). Sites randomized to Enhanced had relatively higher baseline costs ($13,176 vs. $9,222 in the Core sites). During the Experiment phase, Enhanced sites continued to incur higher implementation costs relative to the Core sites, but these costs steadily declined and ultimately converged with Core sites as they entered the sustainment phase. When comparing Early and Late Experiment phases, Enhanced sites’ average cost was $5,885 during Early Experiment and $3,696 during Late Experiment. Core sites had an average cost of $3,523 during Early Experiment and $1,932 during Late Experiment.

**Implications for D&I Research:** In a funding climate where available resources are already stretched thin, recommending that agencies take on additional responsibilities to implement evidence based screening, referral, and treatment linkage services is a challenging proposition. Agencies need to be aware of what sort of investments will be required to prepare for implementation, during implementation, and to sustain newly adopted practices.

**Primary Funding Source**

National Institutes of Health

### S110 Implementation of a real-world value-based insurance design benefit program to improve access to necessary chronic medications for diabetes patients

#### Connie Trinacty^1^, Vanessa Simiola^1^, Timothy Frankland^1^, Alyce Adams^2^, Andrea Altschuler^2^, Deborah Taira^3^, Dennis Ross-Degnan^4^

##### ^1^Kaiser Permanente Hawaii, Honolulu, HI, USA; ^2^Kaiser Permanente Northern California, Oakland, CA, USA; ^3^Daniel K. Inouye College of Pharmacy; University of Hawaii at Hilo, Honolulu, HI, USA; ^4^Harvard Medical School and Harvard Pilgrim Health Care Institute, Boston, MA, USA

###### **Correspondence:** Connie Trinacty (connie.mah.trinacty@kp.org)

**Background:** Increasingly popular value-based insurance designs (VBIDs) have been shown to improve short-term adherence to medications for chronic illness with no increase in total spending. However, despite positive evidence, the uptake of VBID has been slow among commercial health plans. Systematic reviews report variation in implementation in different settings.

**Methods:** Kaiser Permanente Hawaii (KPHI) offered WellRx, a drug rider benefit that waives all copays for essential medications and supplies for diabetes, was offered on a pilot basis in 2012-2014 and later in 2015 as a purchased benefit for large employer accounts. Primary (employer interviews, patient surveys) and secondary (electronic medical and claims records) data were used to assess the impact of the program at the individual and organizational level using the RE-AIM framework and its five evaluation components: Reach, Effectiveness, Adoption, Implementation, and Maintenance.

**Findings:** During the pilot program, *reach* expanded from 46% to 92% users with diabetes (n~1700 members). This two-fold increase was mainly due to the change from opt-in to automatic enrollment, now the approach in the operational program. Program *effectiveness* was based on survey data collected among 890 WellRx utilizers. Over 50% of program participants reported increased positive health behaviors such as engaging in daily exercise, checking their blood sugar levels daily, and adjusting their diet for weight loss. Participants also self-reported high confidence in maintaining or improving their blood glucose levels and approximately 80% reported taking medications daily. Of the employers that were offered the operational program in 2014, all but one participated, indicating high *adoption*. *Implementation* was determined using program enrollment versus actual dispensing at the zero-dollar copay rate. Although it took four years to reach this level, over 90% of program participants receive the zero-dollar copay for qualifying medications and supplies.

**Implications for D&I Research:** This study examines a real-world VBID program in a setting that illustrates the challenges of implementing system-level interventions. The RE-AIM framework provides ways to improve program efficiency and impact at the organization-, employer- and patient-levels. Increasing reach, improving effectiveness of care, and expanding employer adoption are areas for future policy, practice, and research. Additional evaluation of program outcomes is needed to determine if expansion is feasible.

**Primary Funding Source**

National Institutes of Health

## Models, Measures, and Methods

### S111 Mapping knowledge brokers in media ecosystems to assess the use of research evidence

#### Matthew Weber^1^, Itzhak Yanovitzky^2^

##### ^1^Hubbard School of Journalism and Mass Communication, University of Minnesota, Minneapolis, MN, USA; ^2^Communication, Rutgers University, New Brunswick, NJ, USA

###### **Correspondence:** Matthew Weber (msw@umn.edu)

**Background:** Recent research on dissemination and implementation has sought to better understand the pathways by which policymakers and end users of research evidence engage with one another and exchange ideas. This work details the methods by which we map the use of research evidence at the state level. The research presented is part of a new research effort connecting the use research evidence to the broader public debates regarding adolescent depression treatment in schools within the state of New Jersey through knowledge brokerage mapping. We focus specifically on the use of news interviews and op-eds, social media posts, public events, social media, and local and regional journalism to map communication and evidence use.

**Methods:** Our approach to mapping focuses on joining disparate datasets by focusing on actors and evidence as key points of intersection. For social media data tracking, we implemented the open-source Social Feed Manager and used the Twitter API to automatically sample key Twitter feeds. Additional social media data were manually collected from Facebook for key actors and key news organization by tracking new posts made during the research period and recording the data. Accounts were identified by using the document analysis to identify a seed list of key actors and then identify their related social media accounts. Methods for other media collection will also be highlighted.

**Findings:** The knowledge brokerage mapping methodology reveals key network structure that connects four elements: (1) actors, (2) the way the actors are linked, (3) actors’ interests with regard to the policy considered, and (4) actors’ role regarding the flow and exchange of policy-relevant information within the network of actors (supplier, broker, or user). By aggregating links among actors the researchers were able to reproduce the network that connects actors to a particular body of research evidence.

**Implications for D&I Research:** The implications of this methodological research focus on the utility of key measures for identifying knowledge brokers. Our ongoing tracking and analysis of research evidence use by policymakers, brokers, and news and social media provides a methodological starting point for similar research around related policy issues and provides a roadmap for work in this area.

**Primary Funding Source**

William T. Grant Foundation

### S112 Comparison of generic vs. intervention-specific referents when measuring the inner organizational context

#### Aaron Lyon^1^, Clayton Cook^2^, Chayna Davis^3^, Eric Brown^4^, Elissa Picozzi^3^, Jill Locke^3^, Lindsay Frederick^3^, Mark Ehrhart^5^

##### ^1^Psychiatry and Behavioral Sciences, University of Washington, Seattle, WA, USA; ^2^University of Minnesota, Minneapolis, MN, USA; ^3^University of Washington, Seattle, WA, USA; ^4^University of Miami, Miami, FL, USA; ^5^University of Central Florida, Orlando, FL, USA

###### **Correspondence:** Aaron Lyon (lyona@uw.edu)

**Background:** Valid and pragmatic measures are a critical – but elusive – ingredient of contemporary implementation research. Studies are needed to “raise the bar” on the predictive validity of instruments to ensure that they demonstrate real-world utility. Instruments assessing the inner organizational context are among the most common in implementation research. Organizational factors are typically assessed with instruments that reference either generic intervention types (e.g., “evidence-based practices”) or specific interventions, but differences in predictive validity due to the referent of the items have never been evaluated. As a component of a federally-funded measurement development project, the current study assessed the associations of generic and intervention specific versions of organizational measures to implementation and service outcomes for two different universal behavioral health prevention programs, delivered in the education sector.

**Methods:** Participants were 441 educators (89% female; 84% non-Hispanic White) working in 54 elementary schools, who had received training in one of two universal, evidence-based behavioral health prevention practices (Schoolwide Positive Behavioral Interventions and Supports [SWPBIS]; Promoting Alternative Thinking Strategies [PATHS]). Participants within each school were randomized to complete school-adapted versions the Implementation Leadership Scale, Implementation Climate Scale, and Implementation Citizenship Behavior Scale that contained either generic “evidence-based practice” or intervention-specific referents. Objective SWBPIS and PATHS fidelity assessments were completed by independent observers. Educators also completed ratings of student behavioral outcomes (on task behavior, oppositional behavior, oppositional behavior).

**Findings:** Results indicated statistically significant associations (p<.01) with independent observations of intervention fidelity only when ratings were completed using intervention-specific versions of the instruments. Effect sizes for the intervention-specific versions (*r* = .205 - .255 for total scores) were of equal or greater magnitude than those that have typically been identified in previous organizational research. Relationships with student outcomes were apparent for each constructs, but no clear pattern emerged in terms of the relative strength of the two version of the scales in predicting student outcomes.

**Implications for D&I Research:** This research enhances the specificity with which organizational determinants of implementation should be assessed and provides preliminary evidence that their relationship to implementation outcomes is likely to be enhanced by the inclusion of intervention-specific referents in assessment instruments.

**Primary Funding Source**

Institute of Education Sciences

### S113 Predicting the sustainability of evidence-based interventions: A neural network approach to investigating links between contexts, mechanisms and outcomes

#### Tim Rappon, Alyssa Indar, Erica Bridge, Whitney Berta

##### Institute of Health Policy, Management and Evaluation, University of Toronto, Toronto, ON, Canada

###### **Correspondence:** Tim Rappon (tim.rappon@mail.utoronto.ca)

**Background:** This submission builds on work presented at last year’s D&I conference describing the use of a neural network to map the sustainability of quality improvement (QI) initiatives for the health care of older adults. While the model was able to produce some relevant insights, it suffered from two key limitations: 1) it was not generalizable to other populations or other types of evidence-based interventions (EBI), since the initial dataset came from a focused scoping review and 2) it performed poorly at predicting the sustainment and sustainability of QI interventions.

**Methods:** We created an expanded dataset by coding an additional 257 articles which were cited in one of four recent comprehensive reviews of sustainability for EBI (Stirman *et al*. (2012), Ament *et al.* (2015), Tricco *et al.* (2016) and Shelton *et al.* (2018)), bringing our total sample to 312 articles. In addition to extracting implementation and post-implementation strategies (based on Powell *et al.* (2015)), we also distinguished between sustainment (i.e. implementation outcomes) and sustainability (i.e. staff/patient outcomes) and coded for type of intervention, setting, intervention target (based on ICD10), model or framework of sustainability, costing and adaptations (Stirman *et al.* (2019). We used this data to train a Kohonen self-organizing map, which groups similar studies together based on these characteristics. We performed leave-one-out cross-validation using the extracted variables to assess the sensitivity and specificity of the map’s predictions of sustainment and sustainability.

**Findings:** The self-organizing map achieved a diagnostic odds ratio of 5.2 (p<0.05) for sustainability but was not significant for sustainment. We observed that omitting information on clinical targets, intervention setting, adaptations, or post-implementation strategies resulted in significant reductions in predictive power. Several key linkages for sustainability were apparent, such as the special importance of stakeholder interrelationships for community-based interventions.

**Implications for D&I Research:** Our study presents a novel method for investigating relationships between contexts, mechanisms and outcomes of sustainability. It supports the validity of realist, context-dependent models of sustainment/sustainability that emphasize the importance of adaptation (such as the Dynamic Sustainability Framework). We are also developing a web-based tool which allows implementers to get tailored sustainability recommendations for their projects based on our model.

### S114 Advancing our understanding of organizational constructs influencing the delivery of evidence-based practice across publicly-funded mental health agencies: A secondary data analysis using qualitative comparative analysis

#### Sapna Mendon^1^, Lawrence Palinkas^1^, Michael Hurlburt^1^, Rinad Beidas^2^

##### ^1^University of Southern California, Los Angeles, CA, USA; ^2^University of Pennsylvania, Philadelphia, PA, USA

###### **Correspondence:** Sapna Mendon (smendon@usc.edu)

**Background:** To maintain effectiveness of widely-implemented evidence-based practices (EBPs), it is crucial to have a strong understanding of what factors contribute to fidelity. Studies have found factors such as organizational culture, access to training and education, leadership engagement, and implementation climate to be associated with fidelity. However, there is little knowledge about how these constructs synergistically contribute to implementation outcomes; information that can strategically expedite implementation. The current study targets organizational complexities and applies an innovative analytic technique to identify combinations of characteristics necessary and sufficient for fidelity.

**Methods:** This mixed methods study was informed by data collected from multi-level stakeholders, including treatment developers, agency stakeholders, system-level leaders and key personnel in 15 publicly-funded mental health agencies. Fidelity was measured using the Therapy Procedures Checklist- Family Revised (TPC-FR), and inner setting characteristics were measured using the Implementation Leadership Scale (ILS), Implementation Climate Scale (ICS), and Organizational Social Context scale (OSC). Analyses were conducted using Qualitative Comparative Analysis (QCA), a set theoretic approach applying Boolean logic. This method allowed for a qualitative approach to a quantitative analytic technique, granting access to examine organizational complexities. Quantitative scores from all aforementioned measures informed the presence or absence of a condition for a given organization, and concrete examples from qualitative interviews were used to supplement data calibration. Within the organizational sub-domains outlined in the CFIR, there are 32 possible combinations of characteristics. QCA identified which conditions were necessary to yield strong fidelity, and which configurational pathways were sufficient.

**Findings:** Findings showed two conjunctural combinations sufficient for maintaining fidelity: 1) structural characteristics and implementation climate and 2) implementation climate and readiness for implementation and organizational culture. This finding indicates that strong fidelity will occur when these pathways are present.

**Implications for D&I Research:** This study moved beyond identifying individual facilitators and barriers to fidelity by using a configurational approach to identify how these known influencers work in combination. Such knowledge assigns value to CFIR characteristics and helps to prioritize which aspects can expedite implementation efforts in practice. These combinations highlight areas of organizational infrastructures that can be addressed to improve the use of EBP in community-based settings.

### S115 Quality improvement and implementation science in cancer care: Identifying areas of synergy and opportunities for further integration

#### Devon Check (devon.check@duke.edu)

##### Duke University School of Medicine, Durham, NC, USA

**Background**: Efforts to improve cancer care have been driven by two approaches: quality improvement (QI) and implementation science (IS). QI and IS have developed independently but have potential for synergy. To inform efforts to better align these fields, we examined 20 cancer-related QI and IS articles to identify differences and commonalities.

**Methods**: We searched PubMed for cancer care studies that used IS or QI methods and were published in the past 5 years in one of 17 leading journals selected by our interdisciplinary team. When possible, we categorized studies as QI or IS based on authors’ descriptions; otherwise, we categorized studies as QI if they evaluated efforts to improve the quality, value, or safety of care, or IS if they evaluated efforts to promote the adoption of evidence-based interventions into practice. We identified the 10 most frequently cited studies from each category (20 total studies), characterizing and comparing their objectives, methods – including use of theoretical frameworks and involvement of stakeholders – and approaches to practice change.

**Findings**: All IS studies (10/10) and most QI studies (6/10) addressed barriers to uptake of evidence-based interventions. The remaining 4 QI studies sought to improve clinical outcomes, reduce costs, and/or address logistical issues. QI and IS studies employed common approaches to change practice. For example, clinician and staff training was employed in 8/10 IS studies and 4/10 QI studies, and performance monitoring and feedback in 3/10 IS studies and 2/10 QI studies. However, the terminology used to describe these approaches was inconsistent between IS and QI studies. Additionally, few studies (2/10 IS, 1/10 QI) used a theoretical or conceptual framework and only 4/20 (2 from each category) consulted key stakeholders in developing their approach. Most studies (10/10 IS and 6/10 QI) were multi-site and observational, with a minority of studies (2 from each category) using a randomized design.

**Implications for D&I Research:** Cancer-related QI and IS studies had overlapping objectives and used similar approaches but inconsistent terminology. The impact of IS and QI on cancer care could be enhanced by greater harmonization of the disciplines and by promoting rigor through the use of theoretical frameworks and stakeholder input.

**Primary Funding Source**

Kaiser Permanente Washington Health Research Institute Development Fund

### S116 Integrating improvement and implementation science to address extreme heterogeneity: The case of HPV vaccination

#### Brian Mittman (brian.s.mittman@kp.org)

##### Kaiser Permanente Southern California, Pasadena, CA, USA

**Background**: Implementation scientists increasingly recognize the challenge of extreme heterogeneity across place and time in implementation barriers, facilitators and related influences on implementation strategy selection and effectiveness. Heterogeneity requires data-guided selection and ongoing tailoring of implementation strategy bundles to meet local circumstances. Unfortunately, methods derived from clinical research are optimized to study simple interventions (e.g., fixed pharmaceutical interventions) -- to determine whether an intervention is “effective” -- but generally unable to generate insights into how and why an intervention achieves its effects, and, more importantly, how to modify the intervention and its context to increase effectiveness. Improvement science offers numerous conceptual and methodological approaches for addressing heterogeneity and guiding site-specific tailoring. We integrated improvement science and implementation science methods to address gaps in HPV vaccination delivery, an implementation problem that has proven highly resistant to conventional intervention approaches, largely due to patterns of extreme heterogeneity in local barriers, facilitators and circumstances.

**Methods**: We integrated standard improvement science techniques (root cause analysis, local quality teams, time series analysis) with conventional clinical trial approaches to diagnose and address highly heterogeneous barriers to HPV vaccination performance improvement in Kaiser Permanente Southern California, a large integrated healthcare system serving over 4 million members. Implementation strategy selection and tailoring are guided by a menu of forms linked to core functions (Jolles et al 2019) to ensure internal validity and generalizability.

**Findings**: Pilot study findings confirm heterogeneity of barriers. A form-function menu derived from local barrier assessment data matches a set of core functions derived from published literature and offers a collection of implementation strategy forms for use by local quality teams developing site-tailored implementation approaches.

**Implications for D&I Research:** Implementation science addresses phenomena that differ dramatically from simple interventions for which clinical research methods are optimized. Implementation studies asking “Is intervention X effective” or “Is Intervention A superior to Intervention B” will generally produce answers such as “Sometimes” or “It Depends.” Implementation researchers must replace efforts to emulate clinical trialists with guidance and tools from the field of improvement science. This integration should facilitate dramatic improvements in our field’s ability to generate useful implementation insights, evidence and guidance.

**Primary Funding Source**

Kaiser Permanente Washington Health Research Institute Development Fund

### S117 Quality improvement vs. implementation research: Finding middle ground

#### Ann Sales (salesann@umich.edu)

##### University of Michigan, Ypsilanti, MI, USA

**Background:** Many people, including researchers who work in the fields of quality improvement (QI) and/or implementation research (IR), find it difficult to define whether an activity is best defined as QI, or IR. The distinction is important because of regulatory issues, such as human subjects protection, and because of status related to terms such as “science” and “research”. In this presentation I describe a project that straddles both areas, funded through the VA Quality Enhancement Research Initiative (QUERI).

**Methods:** The project focuses on supporting implementation of goals of care conversations in Veterans Health Administration long term care facilities. We developed and now send feedback reports to both nursing home and home based primary care teams in 19 VA facilities detailing their achievements in documenting goals of care conversations on a monthly basis. We now vary the reports systematically to test differences in response to the feedback reports when we provide enhancements—either tips for action planning, or information about comparison performances. We follow the feedback report distribution with surveys once a quarter to estimate uptake. The overall project including distribution of feedback reports and evaluation of their impact is deemed QI, while the administration of the survey to understand the uptake of the enhanced reports, is research.

**Findings:** Response to the feedback reports overall has been unclear. The evaluation design, as part of the QI evaluation, involves a cohort analysis, using a matched set of facilities, to see whether documentation of goals of care conversations is greater in facilities receiving the feedback reports. While this analysis is not yet complete, we have interim results showing graphical representation of differences in documentation. The results of the research component are still in process, but will be available in interim form by December.

**Implications for D&I Research:** Regulatory issues, as well as organizational perspectives on what is deemed QI vs. research, condition the work being done, and how it is regulated. These issues impose burden on individuals doing the work, resulting in competing pressures to get projects deemed one or the other. Middle ground approaches, such as the VA QUERI program, may offer solutions to some of these issues.

**Primary Funding Source**

Kaiser Permanente Washington Health Research Institute Development Fund

### S118 How epidemiology can strengthen both improvement and implementation science

#### Don Goldmann (DGOLDMANN@IHI.ORG)

##### Institute for Healthcare Improvement, Boston, MA, USA

**Background**: Both improvement and implementation science offer strong methods for adoption and spread of change concepts and best practices. Though there are important differences between these fields, both emphasize having a clear program/change theory, measuring processes and outcomes, and demonstrating a causal link between the improvement/implementation approach, changes made, and improved processes and outcomes. Credibility of improvement/implementation efforts depends on appropriate program design and evaluation so that causation can be differentiated from association, and observed improvements attributed to the program. Surprisingly, neither field seems to have thoroughly explored the contributions that epidemiology can make to inferring causality, especially dealing with bias, confounding, and the counterfactual.

**Methods**: We explored how adopting basic principles of epidemiology (adapted from criteria developed by Austin Bradford Hill) and causal inference (as promulgated by Judea Pearl and others), could strengthen the credibility of improvement and implementation programs. We examined the extent to which these principles were applied in published improvement collaboratives, as summarized by Wells, et. al. (1). We also examined whether these publications adhered to SQUIRE 2.0 and StarI guidance for describing methods and contextual factors, which would be critical in efforts to spread these programs.

**Findings**: Published results of improvement collaboratives, while purportedly showing that the changes implemented were effective in improving designated outcomes, generally did not adequately apply epidemiological principles, such as describing and mitigating bias and confounding. Nor did they adhere to SQUIRE or StarI publication standards. Therefore, results could not be attributed confidently to these programs. In-depth study of efforts to spread the two influential programs demonstrated lack of attention to important Hill criteria, limiting their credibility and generalizability; efforts to spread these programs also demonstrated insufficient attention to fidelity of methods and context.

**Implications for D&I Research:** Credibility of improvement projects would be strengthened if design (e.g., stepped wedge or cluster randomized trials) and evaluation incorporated basic epidemiological approaches to mitigating bias and confounding. Efforts to replicate apparently effective programs might be more successful if improvers and implementers described what they did and how they did it more rigorously and adapted their approach to local context when spreading their work.

**Primary Funding Source**

Kaiser Permanente Washington Health Research Institute Development Fund

**Reference**

1. Wells S, Tamir O, Gray J, Naidoo D, Bekhit M, Goldmann D (2018). “Are quality improvement collaboratives effective? A systematic review”. *BMJ Qual Saf*. 27: 226-240.

### S119 Embracing and guiding, rather than suppressing or ignoring, heterogeneity and tailoring: Core functions and forms in complex health interventions

#### Brian Mittman (brian.s.mittman@kp.org)

##### Kaiser Permanente Southern California, Pasadena, CA, USA

**Background:** Decades of development of clinical research methods have produced approaches optimized for answering clinical effectiveness questions such as “Is Intervention X effective?” or “Is Intervention A superior to Intervention B?” Conventional clinical research methods are well-suited to the study of simple interventions (pharmaceutical agents) and other robust interventions. For most complex health interventions (health promotion, delivery system interventions, implementation strategies), however, the dominant answers to effectiveness questions are “it depends” or “sometimes.” Complex health interventions (CHIs) are often effective in some settings but not others, and in specific settings during one time period but not another. CHIs show extreme heterogeneity and modifiability and their outcomes are often highly mediated, moderated and strongly influenced by contextual factors. Standard clinical research methods are often inadequate for studying CHIs, while conventional understanding and use of concepts such as “fidelity,” “manualized intervention,” “core components” and “adaptation” are either inapplicable or must be significantly redefined for CHIs. Many emerging approaches for studying CHIs are based on the concepts of core functions and forms. This presentation introduces, defines and discusses these concepts.

**Methods:** This presentation summarizes published literature (e.g., Hawe 2004, 2015) on core functions and forms and discusses their application in clinical and health services research and role in the PCORI Methods Standards for Studies of Complex Interventions.

**Findings:** Core functions (the intervention’s basic purpose or aims) are distinct from the detailed forms that operationalize or carry out core functions. Detailed examples are provided in the three presentations illustrating the function/form approach.

**Implications for D&I Research:** Implementation researchers are increasingly recognizing the limited validity of standard assumptions underlying clinical research when applied to implementation phenomena, accepting the need to study implementation strategy tailoring and mediators, moderators and mechanisms to understand “how,” “when” and “why” implementation strategies achieve their effects -- and how they can be modified to increase effectiveness. Conceptualizing implementation strategies as core functions (rather than core components) accompanied by a menu of forms that are detailed, specific and non-generalizable should significantly enhance the implementation science field’s success in generating valid implementation insights, evidence and guidance.

### S120 Using ethnographic methods to identify the core functions and forms of patient-centered medical homes (PCMH) within federally qualified health centers (FQHCs).

#### Monica Perez Jolles (mjolles@usc.edu)

##### University of Southern California, Los Angeles, CA, USA

**Background:** Despite significant investment in transforming primary care settings into patient-centered medical homes (PCMHs), guided by five principles (accessibility, coordination, comprehensiveness, patient-centeredness and commitment to quality and safety), evidence of PCMH effectiveness has been mixed. One reason may be extreme heterogeneity in PCMH features and contexts and our failure to capture unique sources of variation, a common challenge in studies of complex health interventions. This session describes a systematic approach to identifying PCMH core functions and creating a nationally applicable PCMH “functions and forms” matrix.

**Methods:** We employed ethnographic methods using semi-structured interviews, shadowing and document review to understand the full range of goals, purposes and features of PCMH care model features. We recruited a randomly selected sample of clinical providers, care coordinators, clinic managers, staff and patients (n=21 encounters) at three Southern California FQHCs. Additional information was gathered from managers at the network’s quality improvement and research departments (n=4). Data collection protocols used familiar language and visuals to depict PCMH in terms of functions and forms. Data were summarized into an interactive excel database and menu of local PCMH forms, the workforce assigned to carry out those forms, and aligned PCMH functions based on national standards from AHRQ and NCQA.

**Findings:** We found areas of misalignment between AHRQ and NCQA expectations in 3 PCMH principles: accessible care, patient-centered care and commitment of quality and safety. A few local forms did not align with their/ corresponding function(s). Interviews with professionals asked them to conceptualize their daily activities as forms that operationalize specific PCMH functions. For providers and staff, the concepts of forms were easier to grasp than the concept of core functions.

**Implications for D&I Research:** Conceptualizing and studying PCMH as a complex health intervention by distinguishing between its core functions and local forms can help clarify the intervention’s mechanisms of effect and better inform the selection and tailoring of implementation strategies. Our approach offers a valuable model for future efforts to identify core functions and forms of complex health interventions such implementation strategies and strategy bundles.

**Primary Funding Source**

Internal funding

### S121 Emergent functions and forms of emergency department-based peer support for opioid use disorders

#### Alan McGuire^1^, Kristin Gilmore Powell^2^, Peter C. Treitler^2^, Karla Wagner^3^, Krysti Smith^3^, Nina Cooperman^4^, Lisa Robinson^5^, Jessica Carter^6^, Bradley Ray^7^, Dennis Watson^5^

##### ^1^HSR&D Center for Health Information and Communication, Roudebush VA Medical Center, Veterans Health Administration, Indianapolis, IN, USA; ^2^Rutgers University, New Brunswick, NJ, USA; ^3^University of Nevada, Reno, Reno, NV, USA; ^4^Rutgers Robert Wood Johnson Medical School, New Brunswick, NJ, USA; ^5^University of Illinois at Chicago, Chicago, IL, USA; ^6^Richard L. Roudebush VA Medical Center, Indianapolis, IN, USA; ^7^Indiana University Purdue University Indianapolis, Indianapolis, IN, USA

###### **Correspondence:** Alan McGuire (abmcguir@iupui.edu)

**Background:** Emergency department (ED)-based peer support programs aimed at linking persons with opioid use disorder (OUD) to medication assisted treatment and other recovery services are a rapidly expanding approach to addressing the opioid crisis. Six states are already leveraging SAMHSA Opioid State Targeted Repose (STR) grants for multi-site rollouts. However, there is little evidence evaluating such programs’ effectiveness or clarifying the critical elements of the model. This project utilizes three states’ experiences in order to identify core functions of such programs and forms used to fulfill these functions.

**Methods:** Analogous to Jolles and colleagues “top-down” approach, evaluation teams from each state held ongoing macro-level discussions regarding their states’ rollouts. Eventually, core functions began to crystalize. Subsequently, consistent with a “bottom-up” approach, they used program-level data to chart key programmatic elements (forms). Data from 22 programs serving between 1 and 17 EDs included semi-structured interviews, site observations, and/or focus groups. Qualitative data were analyzed using a general inductive approach conducted in 3 steps.

**Findings:** Core functions include: Integration of peer supports in EDs; Alerting peers of eligible patients and making the patient aware of peer services; and Connecting patients with recovery services. There were several notable differences in forms. Peer integration differed in terms of peer’s physical location and who hired and supervised peers. In terms of alerting peers, peers often depend on ED staff to alert them to potential patients while people other than the peers often first introduce potential patients to programming. In terms of connecting patient with recovery services, programs generally schedule initial appointments for recovery services for patients, but some programs provide a range of other services aimed at supporting participation in recovery services.

**Implications for D&I Research:** Model clarification is a key factor in implementation science and the field requires an approach that lends itself to the synergy of model fidelity and adaptation. The forms and functions approach lends itself to productively organizing and comparing knowledge gained across multiple sites without precluding innovation by prescribing certain forms.

**Primary Funding Source**

Foundation

### S122 Identification of core forms and functions in the secondary stroke prevention by uniting community and chronic care model teams early to end disparities: The succeed trial

#### Michele Galatovich^1^, Brian Mittman^2^, Barbara Vickrey^3^, Monica Ayala-Rivera^4^, Amytis Towfighi^5^

##### ^1^University of Miami, Miami, FL, USA; ^2^Kaiser Permanente Southern California, Pasadena, CA, USA; ^3^Icahn School of Medicine at Mount Sinai, New York, NY, USA; ^4^University of Southern California, Los Angeles, CA, USA; ^5^Rancho Los Amigos National Rehabilitation Center, Downey, CA, USA

###### **Correspondence:** Amytis Towfighi (atowfighi@dhs.lacounty.gov)

**Background:** The SUCCEED trial tested the efficacy of a Chronic Care Model/community-based intervention to improve vascular risk factor control among individuals with recent stroke (ischemic or hemorrhagic) or transient ischemic attack, enrolled from 4 safety-net hospitals of the 2^nd^ largest municipal health system in the United States. Subjects randomized to intervention were managed by an advanced practice practitioner (APP)-community health worker (CHW) team, following evidence-based protocols. Within the framework of a minimum of three clinic visits, 3 home visits, and Chronic Disease Self-Management Program workshops, the care team tailored the intervention to meet the patient’s needs. In order to describe how the various components of the intervention were adapted to local circumstances and patients’ needs in this complex intervention, we used the framework of form vs. function to describe key components of the intervention.

**Methods:** Using our conceptual model of the intervention, we identified key domains, motivating needs, and functions of the intervention. Through semistructured interviews with the five CHWs and four APPs, we identified the various forms that were used to achieve the functions. Data were summarized into an Excel database and menu of forms. The care team members then rated each form with respect to frequency of use and utility.

**Findings:** The SUCCEED intervention targeted the following domains: medication adherence, stroke literacy, self-management skills, care coordination, healthcare system navigation, social support, and addressing barriers. Each domain had approximately 10 functions, which subsequently had numerous forms of achieving those functions. Although most forms were developed a priori as part of the intervention, some were developed during the course of the trial by the care team to address their patients’ unique needs.

**Implications for D&I Research:** The form vs. function framework is useful for describing real-world adaptations of complex interventions. The development of a menu of all the possible forms in which key functions can be addressed allows for a more nuanced understanding of how the intervention can succeed in real-world settings as well as provide guidance for implementation in various settings.

**Primary Funding Source**

National Institutes of Health

### S123 Necessary but not sufficient: A multimethod study of the role of champions in heathcare-related implementation

#### Edward Miech^1^, Nicholas Rattray^1,2^, Teresa Damush^2^

##### ^1^VA PRIS-M QUERI, Veterans Health Administration, Indianapolis, IN, USA; ^2^VA Roudebush Medical Center, Veterans Health Administration, Indianapolis, IN, USA

###### **Correspondence:** Edward Miech (edward.miech@va.gov)

**Background:** One factor often cited as crucial to effective implementation in healthcare is the presence of champions. Few studies, though, have described or explained how champions influence implementation outcomes. This analysis sought to identify specific causal paths by which champions influenced implementation success in a large, prospective and national study of VA acute stroke programs.

**Methods:** This multimethod analysis of champions integrated three discrete research methods: construct scoring, Configurational Comparative Methods (CCMs) and qualitative analysis. This research was based on the VA RE-INSPIRE study, which examined the implementation of acute stroke programs at VA medical centers across the United States between 2012-2015. The Consolidated Framework for Implementation Research (CFIR) served as the study's conceptual framework. Semi-structured interviews were conducted at three annual site visits at 11 VA medical centers; over 150 VA staff participated, resulting in over 300 interviews. Interview transcripts were qualitatively coded by study team members. Next, the project team systematically applied 20 CFIR constructs directly to the interview data for each of the 33 site visits, scoring each construct on a scale from +2 (strong positive influence on the implementation process) to -2 (strong negative influence). This dataset of over 600 CFIR scores was analyzed with CCMs to determine how Champions and other constructs influenced implementation outcomes. These cross-case solutions were then examined in detail using within-case qualitative analyses.

**Findings:** The presence of champions was found to be a "necessary but not sufficient" condition for healthcare-related implementation success. Effective champions alone were not consistently linked to implementation success, but were necessary for effective reflecting and evaluating, which in turn proved sufficient for implementation success: effective champions → effective reflecting & evaluating → implementation success. The presence of champions did not always translate into effective Reflecting & Evaluating; in these cases, implementation was not successful. Qualitative analyses specified the particular causal mechanisms linking Champions with Reflecting & Evaluating.

**Implications for D&I Research:** Multimethod research offers new ways to parse the necessity/sufficiency of specific conditions like the presence of champions for implementation success, as well as determine the causal pathways by which certain conditions work together to yield outcomes of interest.

**Primary Funding Source**

Department of Veterans Affairs

### S124 Using computational comparative methods to synthesize findings across studies using the consolidated framework for implementation research (CFIR)

#### Laura Damschroder^1^, Edward Miech^2^, Julie Lowery^3^

##### ^1^VA Ann Arbor Healthcare System, Veterans Health Administration, Ann Arbor, MI, USA; ^2^VA PRIS-M QUERI, Veterans Health Administration, Indianapolis, IN, USA; ^3^VA Ann Arbor Center for Clinical Management Research, Veterans Health Administration, Ann Arbor, MI, USA

###### **Correspondence:** Laura Damschroder (laura.damschroder@va.gov)

**Background:** The Consolidated Framework for Implementation Research (CFIR) provides a theory-based approach to identifying barriers and facilitators to implementing evidence-based innovations. The CFIR was used to identify determinants for seven different implementations of seven different programs within Veterans Health Administration (VHA). The goal of this study was to use computational comparative methods (CCMs) to identify pathways to successful and to unsuccessful implementation.

**Methods:** The seven evaluations collected qualitative data via semi-structured interviews from stakeholders (e.g., providers, staff) for 53 VHA settings. The CFIR is comprised of 39 constructs which were used to develop codebooks by which to code qualitative data. Quantitative ratings (-2 to +2) were assigned to each setting for each CFIR construct using published methods. Ratings indicated valence (barrier versus facilitator) and magnitude of influence on implementation (weak, strong). Implementation outcomes were tracked for each case. QCA methods were used to identify pathways to successful implementation and pathways to unsuccessful implementation. The QCAPro package within R was used for analyses.

**Findings:** Three pathways explained 92% of successful implementations. One pathway was the presence of a single facilitating condition: strong positive Leadership Engagement. Two pathways required the combination of multiple conditions: 1) weakly positive Leadership Engagement combined with positive (strong or weak) Access to Knowledge & Information; or 2) positive (strong or weak) Networks & Communication combined with Goals & Feedback and Reflecting & Evaluating. Likewise, three pathways explained 89% of failed implementations. Two pathways required the presence of a single barrier related to: 1) Leadership Engagement; or 2) Implementation Leader or Champion. One additional pathway involved combined barriers related to Access to Knowledge & Information with Networks & Communication. All solutions were 100% consistent.

**Implications for D&I Research:** When implementing evidence-based innovations, there is no one path to successful or to unsuccessful implementation. CCM, combined with consistent use of a theoretical framework like the CFIR, provides a powerful approach to building the necessary knowledge-base about what interventions work where and why.

**Primary Funding Source**

Department of Veterans Affairs

### S125 New methods enhancing the use of qualitative data in simulation modeling: A case in uncovering mechanisms for primary care transformation

#### Rod MacDonald^1^, Andrada Tomoaia-Cotisel^2^, Zaid Chalabi^3^, Samuel Allen^4^, Karl Blanchet^5^, Michael Magill^6^

##### ^1^James Madison University, Harrisonberg, VA, USA; ^2^RAND, Arlington, VA, USA; ^3^University College London, London, United Kingdom; ^4^Worchester Polytechnic Institute, Worcester, MA, USA; ^5^London School of Hygiene & Tropical Medicine, London, United Kingdom; ^6^University of Utah, School of Medicine, Salt Lake City, UT, USA

###### **Correspondence:** Rod MacDonald (macdonrh@jmu.edu)

**Background:** Primary care transformation (PCT) is a critical and elusive target. We lack sufficient understanding of the internal processes of change in primary care practices, and of the policies that can facilitate success. We sought to generate a grounded, dynamic, causal theory of PCT, so as to elucidate the mechanisms linking elements that facilitate or impede implementing the Patient Centered Medical Home (PCMH). In simulation modeling, qualitative data often go unused after model conceptualization and resolving diverse perspectives is not explicitly dealt with in most formal methodologies. Given the many disparate experiences and contexts in PCT, we also developed methods for system dynamics modeling that describe how to resolve diverse perspectives in conceptualization and how to use qualitative data *for validation* of both qualitative and simulation models.

**Methods:** We used a mixed methods approach to develop and validate a system dynamics simulation model of PCT. This model maps the journey of a primary care practice from traditional to PCMH care, as influenced by governmental policies, organizational and environmental contexts, and employee preferences. We conducted semi-structured interviews (n=82) and direct observations for 10 primary care clinics and collected operations and outcomes data (2003-2013). These data informed creation and validation of a simulation model showing how policies and preferences interact with each other and PCT. We also held stakeholder discussions and compared our findings to PCT literature to verify the model and assess the generalizability of our theory.

**Findings:** In the core structure of primary care service delivery, services are added to the care team’s docket, those services accumulate, until they are either completed or shed. Our model shows how PCT is structurally connected to this backbone in a complex feedback system which gives rise to tensions. Tensions are felt more acutely when system changes are implemented and the length of transformation is transformation is 5+ years.

**Implications for D&I Research:** This work provides a structural explanation for the various developmental pathways (and phases) observed in this case study and in wider PCT literature. Understanding this structure and leveraging policies and preferences that facilitate PCT may enable stakeholders to successfully shepherd transformation. Methods developed strengthen model building and validation.

**Primary Funding Source**

Agency for Healthcare Research and Quality

### S126 Implementation mapping: A promising and innovative method to design and select implementation strategies for firearm safety promotion in pediatric primary care

#### Rinad Beidas^1^, Shari Jager-Hyman^1^, Courtney Wolk^1^, Leopoldo J. Cabassa^2^, Maria Fernandez^3^

##### ^1^University of Pennsylvania, Philadelphia, PA, USA; ^2^Washington University in St. Louis, Saint Louis, MO, USA; ^3^The University of Texas Health Science Center at Houston School of Public Health, Houston, TX, USA

###### **Correspondence:** Rinad Beidas (rbeidas@upenn.edu)

**Background:** Implementation strategies are techniques designed to enhance the adoption, implementation, and sustainment of research-supported clinical interventions into practice—they are the interventions of implementation science. Over 70 distinct implementation strategies have been described in published taxonomies. Designing and selecting the most appropriate implementation strategies for use in practice is a complex and challenging task and one for which the literature has provided limited guidance. Further, the literature has been hampered by a lack of participatory approaches in designing and selecting implementation strategies and has not included the voice of stakeholders in an adequate fashion. Implementation mapping is a promising and innovative method to design and select implementation strategies because it is participatory (i.e., includes stakeholders), systematic, and contextually sensitive (i.e., tailored) approaches to assessing the needs of stakeholders and a particular implementation context.

**Methods:** We present on an innovative methodology to design and select implementation strategies using an NIMH funded study (R21 MH109878) as the exemplar. We applied implementation mapping with 210 stakeholders (including physicians, parents of youth, leaders of health systems, firearm experts) to design a menu of implementation strategies for health systems interested in implementing firearm safety promotion in pediatric primary care. We report on the feasibility and acceptability of this methodological approach, developed in other disciplines and applied to implementation strategy development.

**Findings:** We found that implementation mapping was feasible and acceptable in the design and selection of implementation strategies as demonstrated by the high levels of engagements and generation of implementation strategies. However, we also identified barriers to operationalizing the steps in a reproducible way and ensuring that the stakeholder voice was adequately represented throughout all stages of the process.

**Implications for D&I Research:** Implementation mapping is a promising methodological approach to the generation of implementation strategies. Research is needed to test whether strategies developed via these methods are more effective than strategies developed through competing approaches. These approaches may make it easier for designing and selecting the most appropriate implementation strategies for use in practice; we will provide concrete resources and steps to using these approaches in policy and practice.

**Primary Funding Source**

National Institutes of Health

### S127 A user-friendly resource to help select, adapt, and operationalize d&I models for proposal writing and research

#### Borsika Rabin^1,2^, Ross Brownson^3^, Russell Glasgow^2^, Rachel Tabak^3^

##### ^1^University of California, Veterans Health Administration, San Diego, La Jolla, CA, USA; ^2^University of Colorado School of Medicine, Aurora, CO, USA; ^3^Washington University in St. Louis, St. Louis, MO, USA

###### **Correspondence:** Borsika Rabin (Borsika.a.rabin@gmail.com)

**Background:** Selection, adaption, and application of theories, models, and frameworks (hereafter known as “models”) support rigorous and reproducible D&I science research. Though a number of D&I science literature reviews and tools exist, researchers still describe barriers to selection, adaptation, and integration of models into their work. This presentation will introduce a significantly updated and enhanced on-line tool to help investigators through these steps.

**Methods:** The authors have developed a searchable website for identifying and comparing existing models (http://www.dissemination-implementation.org/). The initial version of this website, containing information on 76 models has been widely used (185,000 visits for the past 12 months with an average of 500 visits per day and 44% of visits international), it was in need of updating and revision to make it more user-friendly and helpful for non-experts. We used existing web-based tools and other resources (e.g., criteria for selecting theories from Sarah Birken, recent comprehensive literature review by Sharon Straus, taxonomy by Per Nilsen) to expand on the existing D&I Models in Health website with additional information.

**Findings:** Additional website content will be presented including: 1) development and use of logic models to identify key constructs, 2) selection of D&I models and abstractions of 15 additional models to cover the vast majority of commonly used models; 3) more detailed guidance on the adaptation and integration of D&I models; 4) application of D&I models including using them for quantitative, qualitative, and/or mixed methods evaluation; and 5) an enhanced linkage to measures of key constructs in the various models. An easy to follow guide that participants can use (e.g., a fillable pdf) and examples from multiple setting/topic areas are provided for each step.

**Implications for D&I Research:** The presentation aims to enhance the rigor and scope of D&I science research by facilitating the processes of selecting, adapting, and applying models. This revised resource should help enhance decision making, rationale for model selection, and use of/measurement of theoretical constructs in D&I research.

**Primary Funding Source**

University of Colorado and Washington University in St. Louis

### S128 Enhancing implementation models, theories, and frameworks using intersectionality

#### Justin Presseau^1^, Lora Giangregorio^2^, Jenna Gibbs^3^, Alison Hoens^4^, Danielle Kasperavicius^5^, Christine Kelly^6^, Julia Moore^7^, Isabel Braganca Rodrigues^2^, Matteo Ponzano^2^, Kathryn Sibley^6^, Cheryl van-Daalen Smith^8^, Sharon Straus^5^

##### ^1^Ottawa Hospital Research Institute, Ottawa, ON, Canada; ^2^University of Waterloo, Waterloo, ON, Canada; ^3^McGill University, Montreal, QC, Canada; ^4^University of British Columbia, Vancouver, BC, Canada; ^5^Li Ka Shing Knowledge Institute, St. Michael's Hospital, Toronto, ON, Canada; ^6^University of Manitoba, Winnipeg, MB, Canada; ^7^The Centre for Implementation, Toronto, ON, Canada; ^8^York University, Toronto, ON, Canada

###### **Correspondence:** Danielle Kasperavicius (kasperavicid@smh.ca)

**Background:** Models, theories, and frameworks (MTFs) form the foundation of implementation science. There is increased recognition that intersecting social factors have significant impacts on implementation interventions. Intersectionality explores the complex nature of intersecting social categories (e.g., age, education) and their interaction with compounding power structures (e.g., education system) and forms of discrimination (e.g., sexism). Currently, implementation MTFs lack fulsome exploration of intersectionality considerations. Our objectives were to identify MTFs representing key stages (problem identification; assessing barriers/facilitators to knowledge use; and selecting/tailoring/implementing interventions) in the Knowledge-to-Action Model (KTA) and enhance them with an intersectional lens.

**Methods:** Seventeen MTF experts, implementation researchers/practitioners, and intersectionality experts considered 134 MTFs using a Delphi procedure to select one MTF for each of the three key KTA stages. In round 1, participants formed sub-groups, reviewed full-text articles describing a sub-set of MTFs, and rated each MTF’s overall importance (based on the MTFs’ acceptability, applicability, and usability) on a Likert scale from 1 (unimportant MTF) to 7 (important MTF). Medians and ranges for each MTF were shared. MTFs with a median <5 were excluded from subsequent rounds unless members voiced concerns about exclusion. In round 2, all participants reviewed and rated full-text articles on MTFs selected in round 1. Participants were provided with each MTF’s median, interquartile range, mean, and standard deviation, and if relevant, their previous responses. The same process for discussing and excluding MTFs was used. Participants completed two additional rounds to select final MTFs. Each selected MTF was then enhanced with intersectionality (e.g., by incorporating reflexivity) by experts using co-creation principles.

**Findings:** Experts selected: the Iowa Model of Evidence-Based Practice for the ‘problem identification’ stage; the Consolidated Framework for Implementation Research for the ‘assess barriers/facilitators to knowledge use’ stage; and the Theoretical Domains Framework/Behavior Change Wheel for the ‘select/tailor/implement interventions’ stage. Intersectionality-enhanced versions of the MTFs were then co-developed by the expert team.

**Implications for D&I Research:** Three MTFs were enhanced with intersectionality to support an intersectional approach to implementation intervention development. Future research will explore their usability and impact of considering intersectionality in implementation interventions. This approach could be used for enhancing other MTFs.

**Primary Funding Source**

Canadian Institutes of Health Research (CIHR)

### S129 Advancing the science of intervention adaptation: Integrating human-centered design and implementation science in the UW alacrity center

#### Aaron Lyon^1^, Sean Munson^1^, Emily Friedman^1^, Michael Pullmann^1^, Brenna Renn^1^, Kelly Koerner^2^, Julie Chung^2^, Patricia Arean^1^

##### ^1^University of Washington, Seattle, WA, USA; ^2^Evidence-Based Practice Institute, Seattle, WA, USA

###### **Correspondence:** Aaron Lyon (lyona@uw.edu)

**Background:** Despite the recent emergence of a “science of intervention adaptation,” few methods exist to inform systematic innovation redesign. Usability – the extent to which a product can be used by specified users to achieve specified goals – is a key “upstream” determinant of implementation and service outcomes. The mission of the NIMH-funded University of Washington ALACRITY Center (UWAC) is to study the utility of human-centered design (HCD) methods to enhance both evidence-based psychosocial intervention (EBPI) and implementation strategy usability and implementation outcomes in community settings. To accomplish this, UWAC developed the three-phase Discover, Design/Build, Test (DDBT) framework to drive iterative innovation redesign while retaining core functions.

**Methods:** The DDBT framework employs mixed-methods user testing techniques such as quantitative instruments (e.g., Intervention Usability Scale [IUS]), heuristic analysis by experts, and “lab-based” user testing to assess interventions and strategies. Examples of the application of these methods will be presented. For instance, in one project evaluating an exposure protocol for anxiety disorders, 10 clinicians with differing levels of experience (beginner, *n*=3; intermediate, *n*=4; advanced, *n*=3) participated in usability testing sessions during which they reviewed materials using “think aloud” methods, applied exposure procedures in a behavioral rehearsal, and rated the protocol with the IUS. Experts in EBPI design also completed evaluations using the Heuristic Evaluation Rubric for EBPIs (HERE), which rates EBPIs on six design principles.

**Findings:** Results from multiple projects will be presented. For the exposure protocol example, average IUS ratings (78.61) indicated acceptable usability, but also room for improvement. Ratings for beginner and intermediate users were lower (77.50) than advanced (82.50). High-priority usability issues included the protocol’s failure to block contraindicated behaviors (e.g., excessive reassurance), lack of feedback on the accuracy of the fear hierarchy, and inadequate guidance for backend “processing” of exposures. HERE ratings indicated high efficiency, but low incorporation of “natural constraints” (i.e., static properties of the target context).

**Implications for D&I Research:** HCD methods have strong potential to improve innovation usability and implementability and advance the science of intervention adaptation. Integration of data across UWAC projects will allow for identification of common EBPI and implementation strategy design problems and a matrix of linked redesign solutions.

**Primary Funding Source**

National Institutes of Health

### S130 Making implementation science more rapid: Iterative use of the RE-AIM framework

#### Russell Glasgow^1^, Catherine Battaglia^1,2^, Marina McCreight^2^, Borsika Rabin^3^

##### ^1^University of Colorado School of Medicine, Aurora, CO, USA; ^2^Denver-Seattle Center of Innovation, VA Eastern Colorado Healthcare System, Veterans Health Administration, Aurora, CO, USA; ^3^University of California, San Diego, La Jolla, CA, USA

###### **Correspondence:** Russell Glasgow (russell.glasgow@cuanschutz.edu)

**Background:** Implementation science (IS) models and methods advanced translation of research and understanding of multi-level complex outcomes. They have been widely used for planning and post-hoc evaluation, but seldom to inform mid-course adjustments to interventions or implementation strategies.

**Methods:** We employed the RE-AIM (Reach, Effectiveness, Adoption, Implementation, Maintenance) Framework to guide mid-course evaluation and, if relevant, adaptations across five diverse health services projects in the Department of Veterans Affairs (VA). Using a mixed methods approach, we developed an innovative methodology and new pragmatic tools to engage team members to: a) assess their perceptions of the most important RE-AIM dimension(s) at a given time point in the project, b) evaluate the teams’ satisfaction with progress across the RE-AIM dimensions, and c) inform adaptations as relevant. Based on rapid graphical feedback on the ‘gaps’ between importance and satisfaction with progress, teams developed action plans using Specific, Measurable, Achievable, Relevant and Time-bound (SMART) goals.

**Findings:** An average of seven team members with diverse roles participated in two project-specific assessments and team discussions for each project. Qualitative and descriptive data illustrated the process was feasible, understandable to participants, and useful to help teams reflect upon and adjust their interventions/implementation strategies. There was variability in the RE-AIM domains rated as most important across the different projects. After discussions, teams most often chose reach and adoption domains as needing improvement. Examples of reach action plans were re-engaging key stakeholders to solicit their ideas to reach more participants and revising participant exclusion criteria. An example of an adoption action plan was to conduct chart reviews to closely track adoption. Follow-up interviews revealed satisfaction with the iterative RE-AIM approach and pragmatic tools. Teams implemented action plans based on SMART goals.

**Implications for D&I Research:** The participatory process of employing RE-AIM to support rapid mid-course evaluations proved feasible for five different project teams in the VA. Adjustments to interventions/implementation strategies were undertaken based on progress on different RE-AIM dimensions. Future work is needed to replicate these methods and to determine ideal frequency and timing. Greater focus on rapid and iterative use of IS frameworks is encouraged to facilitate successful translation of research to practice.

**Primary Funding Source**

Department of Veterans Affairs

### S131 Integrating the consolidated framework for implementation research into a culturally responsive evaluation approach: Examples from mixed-methods evaluations of diabetes prevention and management programs reaching underserved populations

#### Sara Jacobs^1^, LaShawn Glasgow^2^, Peter Amico^3^, Kimberly Farris^4^, Gia Rutlege^4^, Bryce Smith^4^

##### ^1^RTI International, Highland Park, IL, USA; ^2^RTI International, Atlanta, GA, USA; ^3^Amico Consulting, Orlando, FL, USA; ^4^Centers for Disease Control and Prevention, Atlanta, GA, USA

###### **Correspondence:** Sara Jacobs (sjacobs@rti.org)

**Background:** Diabetes is a significant population health threat. Evidence-based interventions, such as the Centers for Disease Control and Prevention’s National Diabetes Prevention Program (the National DPP) and diabetes self-management education and support (DSMES) programs can help prevent, delay or manage the disease. However, participation in these programs is suboptimal, particularly among underserved populations. National DPP and DSMES programs specifically targeting underserved participants have not been previously evaluated. Evaluations of these programs can elucidate how to effectively tailor these evidence-based interventions for underserved populations, but evaluations must be conducted in a manner that accounts for cultural and contextual factors.

**Methods:** Culturally responsive evaluation (CRE) is a framework for centering an evaluation in the culture of the populations being evaluated. CRE provides overarching steps for organizing the evaluation and accounting for culture. We integrated CRE with implementation and outcome constructs from the Consolidated Framework for Implementation Research (CFIR) and adapted versions of CFIR (1) to ensure that the evaluation produced useful evidence for putting evidence-based diabetes interventions to use in real-world setting, reaching underserved populations. We conducted mixed-methods evaluations of two programs offering the National DPP lifestyle change program and two providing DSMES services using our integrated CRE-CFIR approach.

**Findings:** Specifically, we incorporated CFIR into three critical aspects of the CRE framework: framing research questions, developing and adapting data collection instruments, and analyzing the data. By including CFIR we were able to quickly develop evaluation questions and data collection instruments that both centered the evaluation in culture and ensured we collected practice-based evidence that supports the implementation of the National DPP lifestyle change and DSMES programs in real-world settings, particularly those reaching underserved populations and communities. The presentation provides example evaluation and interview questions, mapped to relevant CFIR constructs, as well as examples of how we analyzed the data.

**Implications for D&I Research:** As health disparities continue to rise, our integrated approach could be used in other evaluations designed to evaluate the implementation of evidence-based health interventions aimed at reaching underserved populations/communities. This integrated approach could be used within both the health care delivery and community-based settings.

**Primary Funding Source**

Centers for Disease Control and Prevention

**Reference**

1. Rojas Smith L, Ashok M, Morss Dy S, Wines RC, Teixeira-Poit S (2014). Contextual Frameworks for Research on the Implementation of Complex System Interventions. Rockville (MD): Agency for Healthcare Research and Quality (US); Report No.: 14-EHC014-EF.

### S132 Comparing modeling approaches for stepped-wedge cluster randomized trials as applied to d&I research in community and practice settings

#### Lance Ford^1^, Ann Chou^1^, Daniel Zhao^1^, Tabitha Garwe^1^, Juell Homco^2^, Daniel Duffy^2^, Julie Stoner^1^

##### ^1^University of Oklahoma Health Sciences Center, Oklahoma City, OK, USA; ^2^University of Oklahoma School of Community Medicine, Tulsa, OK, USA

###### **Correspondence:** Ann Chou (ann-chou@ouhsc.edu)

**Background:** Dissemination and implementation (D&I) research continues to explore and develop a variety of methods, such as pragmatic trials, effectiveness-implementation-hybrid trials, participatory action research, etc. to address implementation questions. Stepped-wedge design (SWD), a type of cluster-randomized trial, is increasingly being used to study community- and practice-based interventions. SWD is beneficial when the intent is to expose all communities or practices to an intervention but infeasible to roll out all at once. Given SWD is a relatively new design, this study identifies optimal analytic approaches to account for correlation and time effects applied to data from practice-based research settings.

**Methods:** Based on a statewide D&I of a quality improvement intervention bundle to 263 Oklahoma primary care practices, simulations were constructed to investigate how (1) levels of clustering and modeling approaches impact statistical efficiency of intervention effect estimation, (2) conclusions differ among modeling approaches by the extent of extraneous temporal effects, and (3) estimates vary across modeling approaches when the intervention effect is modified by time under the intervention. Programming simulations also examined estimate bias and precision from mixed effects models.

**Findings:** Discounting multiple levels of clustering in SWD can lead to an underestimation of model parameters and standard error, which in turn, may lead to an increase in false positive errors. For SWD trials that extend over long periods of time, potential confounding effect of calendar time must be adjusted. Failing to do so can lead to an overestimation of the treatment effect. When it is expected that there might be a delay in intervention uptake, a time on treatment variable in the modeling approach should be included to avoid obtaining an estimate of the overall treatment effect that overestimates the time-specific treatment effect at the beginning of the intervention, and underestimates the effect once the intervention effect is fully observed.

**Implications for D&I Research:** SWD trials can be used to evaluate the effect and timeliness of implementing evidence-based practices in community- and practice-based interventions, accounting for the nested nature of the data in particular. This study presents statistical modeling considerations that should be addressed to ensure accuracy of study results and conclusions.

**Primary Funding Source**

Agency for Healthcare Research and Quality

### S133 Advancing a methodology for engaging state health agency stakeholders in cost analysis of implementation strategies: An example of early childhood obesity prevention translation across four states

#### Rebekka Lee, Erica Kenney, Catherine Giles, Rebecca Mozaffarian, Angie Cradock, Steven Gortmaker

##### Harvard T.H. Chan School of Public Health, Boston, MA, USA

###### **Correspondence:** Rebekka Lee (rlee@hsph.harvard.edu)

**Background:** It is unclear how best to implement evidence-based interventions in real world community settings in order to maximize their reach and impact on health and minimize costs.

**Methods:** The CHOICES learning collaborative partnership is a training, technical assistance, and modeling initiative designed to build capacity among decision-makers to understand and use cost-effectiveness analysis to identify obesity prevention interventions that offer the best value for money. Four state health agency participants selected Nutrition and Physical Activity Self-Assessment for Child Care (NAP SACC) for potential future implementation or expansion. Two states already implemented the intervention on a limited scale, while one state was implementing alternative obesity prevention interventions that were not evidence-based. We engaged stakeholders from each state to develop plausible implementation strategies given their existing resources and infrastructure and to ensure intervention fidelity. We calculated projected implementation costs associated with the intervention and each implementation strategy using a standard costing protocol and used microsimulation modeling to estimate impact on health and costs of each approach over ten years.

**Findings:** The combined intervention and implementation strategy costs ranged between $56 and $97 per child per year. Major costs across states included labor and travel for statewide training. Reasons for cost variation included different additional components to the core intervention, different trainer salaries, and differences in whether existing programs would be replaced or new programs would be developed. Most states modeled an implementation strategy that utilized existing state quality rating and improvement systems (QRIS), a voluntary program that incentivizes training through increased reimbursement, as an implementation mechanism. The percentage of 2-5 year olds potentially reached in each state varied widely (3-21%) according to a) whether the QRIS system was highly utilized and had existing funding; b) whether the state could require implementation.

**Implications for D&I Research:** As evidence-based interventions are translated into practice in state and local health agencies, population impact and cost will not be uniform. The costing methods described could be a valuable resource for researchers to collect similar cost data across implementation research studies and for agencies to carefully consider how best to leverage existing resources for cost-effective implementation.

**Primary Funding Source**

JPB Foundation

### S134 Rapid cycle systems modeling to optimize implementation: A case example of family navigation for early identification of autism

#### Radley Sheldrick^1^, Nicole Stadnick^2^, Jocelyn Kuhn^3^, Thomas Mackie^4^, Marilyn Augustyn^3^, Sarabeth Broder-Fingert^5^

##### ^1^Boston University School of Public Health, Boston, MA, USA; ^2^University of California, San Diego, La Jolla, CA, USA; ^3^Boston Medical Center, Boston, MA, USA; ^4^Rutgers School of Public Health, Piscataway, NJ, USA; ^5^Boston University School of Medicine, Boston, MA, USA

###### **Correspondence:** Radley Sheldrick (rshldrck@bu.edu)

**Background**: Implementation of complex behavioral interventions is challenged by inconsistent fidelity and the need for contextual adaptations challenges. *Systems science* methods can incorporate prior theory, evidence, and stakeholder input to optimize interventions for specific contexts . We report a case example using *systems modeling* to optimize an evidence-based family navigation (FN) intervention to improve early detection of Autism Spectrum Disorder (ASD) in a safety net hospital.

**Methods**: We developed a *rapid cycle systems modeling* approach that includes four phases: (1) engage key stakeholders to identify goals; (2) gather relevant data; (3) develop prototypes of systems models; (4) share findings with stakeholders to inform further prioritization, modeling, and evaluation.

**Findings**: *Phase 1:* While previous FN research sought to improve early ASD detection by increasing screening and diagnostic referrals, stakeholders from primary care and developmental-behavioral pediatrics identified long wait-lists for diagnostic evaluations as a root cause of delays and target for intervention. *Phase 2:* Clinic policies were evaluated and administrative data (e.g., clinical referrals, workforce capacity) were collected to inform model structure and parameters. *Phase 3:* Previously-developed models were used to identify four candidate strategies to reduce waitlists. Specifically, prior system dynamics models of referral thresholds were used to consider (A) prescreening to “fast-track” children at high risk, and (B) re-assessing engagement before formal evaluation. Queueing models were used to consider (C) over-booking appointments. Linear optimization models were used to consider (D) enabling qualified nurse practitioners to diagnose ASD. Initial models suggest that these four strategies could yield maximal gains of 21%, 18%, 25%, and 33% in the number of available diagnostic appointments, respectively. *Phase 4:* we are now re-engaging with stakeholders to develop protocols to embed strategies within FN (A, B) and pediatric clinics (C, D), assess feasibility, and refine estimates of effect. To date, phases 1-3 required under three weeks.

**Implications for D&I Research:** Improving early ASD detection depends on optimizing local service capacity. For implementation to succeed, FN must contribute to this goal. *Systems modeling* complements standard inferential methods by synthesizing local data and research evidence, while *rapid cycle* iterative design mobilizes stakeholders to develop strategies to adapt evidence-based interventions for context within limited policy windows.

**Primary Funding Source**

National Institutes of Health

### S135 The use of network analysis to assess community partner collaboration in an early childhood system of care grant

#### Jessica Fry, Margo Candelaria, Roshelle Kades, Heather Whitty, Tania Hernandez-Baullosa, Kate Wasserman

##### University of Maryland Baltimore, Baltimore, MD, USA

###### **Correspondence:** Jessica Fry (jessica.fry@ssw.umaryland.edu)

**Background:** The SAMHSA-funded System of Care (SOC) in Southern Maryland aims to expand and sustain early childhood mental health (ECMH) community-based services for children ages 0-5. Over four years community organizations have collaborated to provide a wide array of evidence-based interventions. We examined organization collaboration over time. Based on SOC guiding principles it was hypothesized that through infusing SOC principles over time the organizations would increase collaboration leading to long-term sustainability of an EMHC service array in the region.

**Methods:** Data was collected quarterly from November 2017 through March 2019 using the “Levels of Collaboration Scale”, which asks organizations to rate their collaboration with all other organizations (0=no interaction at all, 1=networking, 2=cooperation, 3=coordination, 4=coalition, and 5=collaboration). R was used to create network collaboration maps. Tableau was used to create heat maps. Network and heat maps were shared with partners to show progress. Study was IRB approved by appropriate organizations. Data collection will continue until September 2019.

**Findings:** Overall, collaboration among all organizations (N=11 to 23) increased (*M*_Time1_=2.18 to M_Time9_=2.46; range=1.79-2.75). Among the 8 core-funded partners, collaboration increased from *M*=2.50 to *M*=3.49 (range= 2.5-3.49). Repeated-measures ANOVA results show there was a statistically significant effect of time on collaboration, *F*(3,102)=3.34, *p*=.022, and there was a significant linear trend *F*(1,34)=8.11, *p*=.007. The largest significant difference was between time point 4 and 9 (p=.018), and there was a clinically significant difference between time point 1 and 9 (p=.005). Overall this indicates primary organizations were “coordinating” with one another more over time, sharing information and resources, having frequent communication, shared decision making and demonstrating increased collaboration over time.

**Implications for D&I Research:** By quantifying collaboration we’ve taken a traditionally abstract idea and turned it into an indicator of project progress, community involvement and sustainability. Changes overall reflect qualitative changes in project goals and partnerships over time such change in project leadership and shared initiatives to create new programmatic content. When built into the project from the start, collaboration can be factored into logic models as an outcome, supporting use of implementation science methods within projects CQI processes to reflect collaboration growth.

**Primary Funding Source**

SAMHSA

## Prevention and Public Health

### S136 Protocol for a county-randomized comparative implementation trial of two delivery strategies for an evidence-based ehealth HIV prevention intervention

#### Brian Mustanski^1^, Nanette Benbow^1^, Patrick Janulis^1^, Dennis Li^1^, Benjamin Linas^2^, Kathryn Macapagal^1^, Sean Murphy^3^, Bruce Schackman^3^, J.D. Smith^1^, Hendricks Brown^1^

##### ^1^Northwestern University, Chicago, IL, USA; ^2^Boston Medical Center, Boston, MA, USA; ^3^Weill Cornell Medical College, New York, NY, USA

###### **Correspondence:** Brian Mustanski (brian@northwestern.edu)

**Background:** Despite substantial NIH investment in developing eHealth HIV interventions, little implementation research has examined strategies to effectively scale up these programs. To advance eHealth intervention implementation, we are conducting a county-randomized comparative implementation trial of two delivery approaches for Keep It Up!, an online, CDC-best-evidence HIV prevention program for young men who have sex with men (YMSM). Designed to be delivered by community-based organizations (CBOs) following an HIV test, a pilot direct-to-consumer (DTC) strategy with home-based HIV/STI testing was promising. This presentation describes the protocol for target county selection and outcome measures in the trial.

**Methods:** We reviewed geographic clustering of counties with 1,500+ YMSM. Standalone counties were automatically selected for inclusion. Among clustered counties, we selected the one with the most Black and Latino 18–29-year-olds and removed those directly adjacent, repeating this procedure until 64 counties were selected. Two adjacent counties were added based on topography. Selected counties were stratified and randomized 2:1 to receive KIU! via the CBO or DTC strategy. Funding proposals were solicited from CBOs in the 44 CBO-strategy counties. Counties with successful CBO applicants were checked against the DTC counties to ensure balance. Outcomes for this type III hybrid trial follow RE-AIM, with the primary outcomes being impact (reach x effectiveness) and cost per infection averted. We also capture various metrics of adoption and implementation from YMSM self-report, KIU! meta-data, and CBO and DTC staff reports and interviews.

**Findings:** The CBO-strategy arm selected 14 CBOs for funding in the first round. A second funding announcement will be released Fall 2019, with the goal of selecting another 8 CBOs, resulting in 22 counties per arm. The subsequent panel presentations will discuss the pragmatic design of each arm as well as the refreshing of the intervention technology to meet the demands of scalability.

**Implications for D&I Research:** Given the national urgency to end the HIV epidemic, understanding the best strategies to implement eHealth HIV interventions to reach the most people is critical to realizing the cost-effective scalability promised by such interventions. Careful selection of research targets and unintrusive measures is critical to maintaining scientific rigor while remaining pragmatic in these studies.

**Primary Funding Source**

National Institutes of Health

### S137 Keeping it up: Updating and upgrading an evidence-based ehealth HIV intervention across contexts and over time

#### Dennis Li, Rana Saber, Brian Mustanski

##### Northwestern University, Chicago, IL, USA

###### **Correspondence:** Dennis Li (dennis@northwestern.edu)

**Background:** eHealth interventions purport to provide advantages in cost-efficient dissemination over face-to-face programs. In practice, however, the technology can become a barrier to its own scalability. Their delivery does not conform to traditional community-based organization (CBO) HIV prevention service infrastructures, in which there is limited technical capacity. The technology must also be adaptable to local contexts while keeping up with technological progression, social expectations, and advancements in HIV prevention science. Little guidance exists on how to update and/or upgrade eHealth interventions over time. Here, we describe principles that eHealth researchers can use to remain agile and responsive to shifting local and global sociotechnical contexts while preserving intervention effectiveness. We draw on Keep It Up! (KIU!) 3.0, a web-based, CDC-best-evidence HIV prevention intervention for young men who have sex with men (YMSM) currently being refreshed from its efficacy trial version (2.0).

**Methods:** Potential adaptations came from several sources: A content review by the Content Team, feedback from YMSM end users, implementation needs suggested by a CBO advisory board, and software needs identified by the Technology Team. Utilizing Mohr’s intervention principles framework, we characterized KIU! 2.0 by its theoretical behavior change strategies (e.g., peer norms) and instantiation components (e.g., role model stories). Adaptations were deemed appropriate if they maintained fidelity to those principles rather than the strict original presentations of content.

**Findings:** KIU! 3.0 has substantially refreshed form and functionality but retains the intervention principles of 2.0. Following a software development model, we launch a minimally viable version in Fall 2019 and will make feature edits over time to ensure continued relevancy of both technology and content. We will monitor user feedback logs and regularly test usability across multiple devices and platforms. Decision rules around the intervention principles will guide these ongoing changes.

**Implications for D&I Research:** Achieving the promise of cost-efficient scalability of eHealth HIV interventions requires a paradigm shift toward treating interventions as a set of principles rather than a static product, coupled with proper planning in intervention design and ongoing usability monitoring. Novel methodologies, such as hybrid and optimization designs, can be used to continue assessing effectiveness.

**Primary Funding Source**

National Institutes of Health

### S138 Making it real: Approaches to ensure validity in community-based settings within a pragmatic implementation trial of an ehealth intervention for HIV prevention for young men who have sex with men (YMSM)

#### Nanette Benbow, Hendricks Brown, J.D. Smith, Brian Mustanski, Justin Jones

##### Northwestern University, Chicago, IL, USA

###### **Correspondence:** Nanette Benbow (nanette.benbow1@northwestern.edu)

**Background:** Little is known about how to scale-up eHealth interventions in community-based organizations (CBO) that provide HIV prevention services. Pragmatic implementation trials can inform scale-up of evidence-based interventions as they take place in settings where clients receive the intervention and compare different real-world alternatives. This talk describes the steps taken to ensure a valid and pragmatic implementation in CBOs.

**Methods:** The Keep It Up! (KIU!) study is a type Ill effectiveness-implementation hybrid design trial that uses a cluster-RCT to compare two implementation strategies: direct-to-consumer (DTC) vs. CBO-based. Formative research was conducted with CBOs and health departments to inform the CBO implementation strategy, including CBO selection process and integration of the intervention into standard practice. 66 counties with the largest YMSM populations were randomized 2:1 to either strategy.

**Findings:** We took multiple steps to resemble realistic implementation in CBOs. To select CBOs, we designed a Request for Proposal (RFP) process similar to that used by CDC and health departments who are the main HIV prevention funders for CBOs. We created an advisory board of CBO and health department representatives to review the RFP and inform the CBO selection process to help ensure similarity to standard practice. To broadly disseminate the RFP, we worked with CDC, national HIV organizations, and health departments to distribute to CBOs. We selected CBOs using an objective RFP review panel composed of people experienced in HIV funding and/or provision of HIV prevention services and modeled the review process after CDC and health department procedures. Our training and capacity building tools to prepare CBO staff for use of the intervention were informed by CDC capacity building provider tools.

**Implications for D&I Research:** In keeping with pragmatic implementation trials, we carried out a CBO selection process that faithfully resembled how CBOs typically apply for and receive funding for HIV prevention services. Mirroring real-world conditions led to complexities not typically encountered in more closely controlled research studies, such as reaching the number of CBOs needed to meet sample size requirements. Input and on-going involvement from stakeholders and practitioners who fund and carry out HIV prevention services were essential to ensuring a valid, pragmatic approach.

**Primary Funding Source**

National Institutes of Health

### S139 Considerations for implementing a direct-to-consumer model of keep it up!, an ehealth HIV prevention intervention for young men who have sex with men

#### Kathryn Macapagal, Krystal Madkins, Dennis Li, Brian Mustanski

##### Northwestern University, Chicago, IL, USA

###### **Correspondence:** Kathryn Macapagal (kathryn.macapagal@northwestern.edu)

**Background:** eHealth HIV prevention interventions are efficacious in reducing risk behavior and STI incidence, but we know little about how to implement them or how they perform in real-world settings. Keep It Up! (KIU!) is a CDC best evidence, brief, online HIV prevention program for young men who have sex with men (YMSM) ages 18-29. KIU! was originally designed to be delivered via community-based organizations (CBOs) after YMSM tested HIV-negative. The efficacy trial included a pilot of a direct-to-consumer (DTC) delivery model. Centralized staff recruited YMSM through online advertising, shipped them HIV/STI test kits, and granted them intervention access upon testing HIV-negative. Here, we describe considerations for adapting this delivery model to mimic real-world settings.

**Methods:** We are testing KIU! in a type III effectiveness-implementation hybrid RCT comparing CBO versus DTC delivery. Sixty-six U.S. counties with large YMSM populations were randomized 2:1, with 22 counties selected for DTC implementation. Preparations occurred in 2018-2019; enrollment begins in fall 2019. We consulted with implementation scientists and a YMSM Advisory Council (YAC) to inform DTC adaptations for this trial.

**Findings:** DTC adaptations focus on the advertising, recruitment, enrollment, and retention processes. We developed a county-by-county recruitment strategy including ZIP-code-based advertising and outreach to CBOs, colleagues, and stakeholders in DTC counties; we will monitor effectiveness of these methods. Per YAC preferences for confirming identity before enrollment, we implemented automated third-party identity verification (versus a phone call with study staff), which may reduce participation barriers while still deterring fake participants. The YAC suggested coupon codes and sexual health products to compensate for lack of monetary incentives to complete KIU!. Other factors that may affect implementation success include staff experience with engaging prospective YMSM participants exclusively online, shipping and processing HIV/STI test kits, and eHealth.

**Implications for D&I Research:** DTC delivery of eHealth HIV prevention interventions promises to reach YMSM more effectively than traditional in-person approaches, yet this has not been studied empirically. Being both KIU! service providers and researchers testing implementation of KIU! requires us to emulate real-world settings while maintaining scientific rigor for the RCT. We accomplish this with careful planning and decision-making together with YMSM and intervention and implementation scientists.

**Primary Funding Source**

National Institutes of Health

### S140 Examining the influence of client characteristics and contextual factors on fall prevention guideline implementation in home- and community-based service programs

#### Lisa Juckett^1^, Alicia Bunger^2^

##### ^1^The Ohio State University, Columbus, OH, USA; ^2^College of Social Work, Ohio State University, Columbus, OH, USA

###### **Correspondence:** Lisa Juckett (lisa.juckett@osumc.edu)

**Background:** The Centers for Disease Control and Prevention’s STEADI Toolkit compiles fall risk screening, assessment, and intervention/referral guidelines designed to help healthcare professionals prevent falls among older adults. Although developed for ambulatory care, STEADI may also be a valuable fall prevention tool in home- and community-based service (HCBS) programs (e.g. home-delivered meals). To inform strategies for implementing STEADI in HCBS programs, this study was guided by the Consolidated Framework for Implementation Research and examined 1) variations in STEADI implementation across three HCBS programs and 2) client-level and inner setting determinants of STEADI implementation.

**Methods:** A two-phase, explanatory sequential mixed-methods approach was used. In Phase 1, quantitative data were extracted from HCBS client charts to assess the extent to which HCBS professionals screened and assessed HCBS clients for fall risk and provided or referred follow-up fall prevention services according to STEADI guidelines. Quantitative data were analyzed using descriptive and logistic regression analyses. In Phase 2, interviews and focus groups were conducted with HCBS frontline staff and administrators to explicate Phase 1 findings. Qualitative data underwent directed content analysis. Quantitative and qualitative findings were triangulated to identify potential implementation strategies tailored to HCBS programs.

**Findings:** Fidelity to STEADI screening, assessment, and intervention/referral guidelines was generally low across the three programs. Screening guidelines were implemented in 46% (n = 291) of cases; implementation of assessment guidelines was omitted entirely; and intervention/referral guidelines were implemented for only 10% (n = 14) of clients identified as a fall risk. Implementation of STEADI screening guidelines was associated with physical impairments, mobility issues, cane use, walker use, and program location. Implementation of intervention/referral guidelines was associated with diabetes and mental health disorders. Qualitative data indicated that HCBS stakeholders attributed low rates of STEADI implementation to the complexity of STEADI guidelines and inner setting determinants (e.g. networks and communications).

**Implications for D&I Research:** Moving STEADI to HCBS settings may require simplifying components of STEADI guidelines. Additionally, implementation strategies should be tailored to establish internal networks within HCBS organizations that enable professionals to effectively coordinate follow-up fall prevention services for clients screened as being at risk for falling.

**Primary Funding Source**

Internal funding, The Ohio State University

### S141 The role of rural church context in participation and attendance in a cardiovascular risk-reduction intervention

#### Kristine Zimmermann, Naoko Muramatsu, Stacie Geller

##### University of Illinois at Chicago, Chicago, IL, USA

###### **Correspondence:** Kristine Zimmermann (kzimme3@uic.edu)

**Background:** Rural US adults experience higher levels of cardiovascular disease (CVD) incidence and mortality and practice health promoting activities less often than non-rural counterparts. Evidence-based health promotion interventions to improve CVD-related behaviors and outcomes have demonstrated limited effectiveness when implemented with rural populations. To promote the dissemination and implementation of effective interventions in rural settings and reduce rural health disparities, this study examined the role of context in a 12-week CVD risk-reduction intervention (Heart Smart for Women, HSFW) followed by a 24-month maintenance intervention (Heart Smart Maintenance, HSM), implemented in 12 rural Illinois churches.

**Methods:** Guided by the Consolidated Framework for Implementation Research (CFIR), we identified five constructs to describe intervention churches. We used linear and logistic regression modeling with generalized estimating equations and clustering participants by church to assess how the five CFIR-derived constructs were associated with person-level attendance and participation outcomes.

**Findings:** “Congregational support for the interventions” was associated with higher HSFW attendance (p < .001). “Church-based leader characteristics,” which referred to the health experience and level of engagement of the church-based lay leaders involved in the intervention, was associated with HSM participation among HSFW attendees (Odds ratio = 2.52, p = .023). Finally, “religious basis for health promotion” was associated with lower HSM attendance (p < .001), whereas “history of health activities in the church” (p = .045), “church-based leader characteristics” (p < .001), and “pastor involvement” (p = .010) were associated with higher HSM attendance.

**Implications for D&I Research:** Rural church context, particularly the attitudes, expertise, and involvement of individuals within churches, appear to play an important role in intervention participation. Results suggest that implementation of community-based health behavior interventions in rural settings may benefit from recruiting existing groups and particularly those with strong social connections, such as churches, worksites, and clubs. Further study of interventions are needed to to better understand the relationship between contextual factors and improved behavioral and clinical health outcomes as well as the generalizability of findings across rural settings.

**Primary Funding Source**

Funded in part by the Office on Women's Health, US Dept. of Health and Human Services

### S142 Community health networks: Capacity for evidence-based program implementation in the context of differing network structures

#### Renee Parks, Rebekah Jacob, Bobbi Carothers, Peg Allen, Stephanie Mazzucca, Rachel Tabak, Ross Brownson

##### Washington University in St. Louis, St. Louis, MO, USA

###### **Correspondence:** Renee Parks (renee.parks@wustl.edu)

**Background:** Mobilizing a network of collaborating organizations is key to addressing community health and an essential public health service. Community structure shapes the identification and implementation of evidence-based programs (EBPs). There is not an ideal structure “type” which posits effective implementation. We examined patterns of community structure, capacity for and delivery of EBPs by integrating social network data and survey data from local health department (LHDs) participating in a project aimed to build capacity for EBPs to control diabetes.

**Methods:** Stakeholders at 12 LHDs in Missouri identified partnering organizations within their jurisdictions to participate in a social network survey to measure collaboration and a perception survey to assess capacity for and delivery of EBPs. These organizations were partners in chronic disease control and community health. Network statistics were calculated for each jurisdiction with R package igraph. Measures of capacity for EBP implementation and EBP delivery were aggregated over jurisdiction. Correlations between network degree centralization (a measure of hierarchy), EBP capacity, and EBP delivery were calculated with R package Hmisc using jurisdiction as the level of analysis (n=12).

**Findings:** The number of organizations in each jurisdiction ranged from 11 to 32, and 26% to 68% of organizations were from non-health areas. The average number of collaboration ties for each organization within the networks ranged between 4.3 and 10. Networks were fairly connected with density ranging between .24 and .62. Degree centralization ranged from 0.29 to 0.69 suggesting that some networks have prominent organizations with many collaborations. More centralized networks tended to be in smaller communities (r=-.52, p=.08) with less LHD staff (r=-.53, p=.08), perceive less of an expectation to use and have skills for implementing EBPs (r=-.42, p=.18), perceive less partnership support for implementing EBPs (r=-.46, p=.14), yet report delivering more EBPs directly (by agency) or through collaboration with a partner (r=.42, p=.17).

**Implications for D&I Research:** Social network analysis provides useful information about the structure and the nature of the collaborations that facilitate EBP implementation. These results suggest smaller communities may collaborate more hierarchically to implement EBPs, highlighting the need for varying approaches to build capacity within networks of varying community structure.

**Primary Funding Source**

National Institutes of Health

### S143 Identifying and prioritizing infrastructure supports for a community-based child maltreatment prevention intervention

#### Leah Bartley, Allison Metz

##### National Implementation Research Network, Carrboro, NC, USA

###### **Correspondence:** Leah Bartley (leah.bartley@unc.edu)

**Background:** Developing and ensuring the necessary supports and conditions for implementation of a primary prevention program requires an understanding of current and potential conditions and structures available for implementation. Yet, there is very little in the literature on how public service systems assess and develop the infrastructure required for successful implementation. The purpose of this applied study is to detail a process in which an implementation team assessed and prioritized implementation infrastructure supports.

**Methods:** Semi-structured interviews were conducted with key informant stakeholders with knowledge and experience related to the current and past infrastructure elements available to support implementation of the primary prevention community-based intervention. An interview protocol was developed to assess implementation supports based on implementation drivers. Implementation drivers are the core components or building blocks of the infrastructure needed to support practice, organizational, and systems change in service to implementation of evidence-based models and innovations (Metz & Bartley, 2011). The semi-structured nature of the interviews ensured flexibility in the question and probing process. A diverse implementation team identified key informants for each driver. A total of 17 interviews were conducted with representation from the state department, community-based implementing agencies, intermediary organizations, the state training department and university partners. Interviews were recorded in order to corroborate detailed interview notes that were captured by the interviewers. Interview data were entered into an excel database and coded for themes across interviews. Results were categorized and shared with the team. A quick-wins facilitation methodology was used with the team to prioritize implementation actions.

**Findings:** For each of the seven implementation drivers, 2-3 critical actions were identified through the semi-structured interviews. Using the quick win methodology, the team prioritized the drivers into quick wins and long-term wins. This prioritization led to a stage-based implementation and data analysis plan. From the implementation plan, a multi-component implementation team structure was developed in order to support the development and installation of the required infrastructure for implementation.

**Implications for D&I Research:** This study provides insight into how an implementation team can lead efforts to understand and align infrastructure to promote successful implementation.

**Primary Funding Source**

NJ Department of Children and Families

### S144 Using implementation science principles to demonstrate improved access to services for pregnant and parenting youth

#### Margo Candelaria^1^, Jessica Fry^1^, Vira David-Rivera^2^, Ashley Cunningham^2^, Christine Johnson^3^

##### ^1^University of Maryland Baltimore, Baltimore, MD, USA; ^2^Baltimore City Health Department, Baltimore, MD, USA; ^3^Maryland Department of Health, Baltimore, MD, USA

###### **Correspondence:** Margo Candelaria (mcandelaria@ssw.umaryland.edu)

**Background:** The Health and Human Services Pregnancy Assistance Fund (PAF) provides funds to increase access to services for pregnant and parenting youth (PPY). In place of new programming, this PAF targeted increasing partnerships to strengthen referral networks and program engagement in a mid-Atlantic city. Consistent with implementation science theory and determinants framework, we anticipated that access to services would improve with increased knowledge of program activities, improved partner relationships, and collaborative strategizing for systems improvement, with at least 300 PPY accessing services in year 1.

**Methods:** PPY serving agencies jointly created a cohesive services system. New activities included monthly meetings, sharing about various programs/services and referral processes, and collective communications efforts. IRB data collection included tracking referrals, continuous quality improvement (CQI) interviews examining referral/engagement successes and barriers, and reported levels of agency collaboration. Analytic approach included descriptive analyses for referrals (using SPSS), content themes analysis across program CQI interviews, and network analyses using the self-report Levels of Collaboration scale (Frey, et al, 2006), (0=no interaction at all to 5=collaboration) creating network collaboration maps (using R Studio) and heat maps (using Tableau). All outcomes were shared with partners quarterly to inform implementation outcomes. Data collection continues through June 2020.

**Findings:** In year 1 (7/18-7/19), 333 PPY (40 fathers) were referred to services with increases over time (Q1=52, Q2=93, Q3=77, Q4=111). This surpassed goal numbers and reflected diverse youth served (20% Hispanic/Latino, 71% African-American). CQI interviews indicated that increased relationships yielded more direct referral networks and engagement, including “warm hands-offs” for PPY. This was supported by network analysis that indicated increased collaboration among all organizations (N=8) increased (*M*_Time1_=2.69 to M_Time2_=2.82), with average number of connections increasing from 5.44. to 8.00.

**Implications for D&I Research:** Findings support the determinants framework and indicate that existing programs had strengthened referrals and engagement with increases in community partnerships, supported by multiple methods of data collection. Rather than focus on implementing new services, the PAF aimed increase access to existing services for PPY, with access surpassing expected goals. This supports the notion that funding can target better access to services by strengthening community partnerships to improve service implementation for youth.

**Primary Funding Source**

Health and Human Services

### S145 How readiness relates to successful adoption and implementation of prevention orientated frameworks

#### Allison Dymnicki^1^, Sabrina Arredondo Mattson^2^, Elizabeth Spier^3^, Beverly Kingston^4^

##### ^1^American Institutes for Research, Washington, DC, USA; ^2^IBS Center for the Study & Prevention of Violence, University of Colorado Boulder, Boulder, CO, USA; ^3^American Institutes for Research, San Mateo, CA, USA; ^4^Institute of Behavioral Science, IBS Center for the Study and Prevention of Violence, Boulder, CO, USA

###### **Correspondence:** Allison Dymnicki (adymnicki@air.org)

**Background:** Researchers from the Center for the Study and Prevention of Violence are partnering with educators in 46 middle schools to implement Safe Communities Safe Schools (SCSS). SCSS seeks to prevent and reduce youth problem behavior, mental, and behavioral health concerns, and increase prosocial behavior by implementing a multi-level and multifaceted approach that includes three core program components: developing a functioning school-based team, building capacity around data use, and implementing evidence-based programs.

**Methods:** Researchers used a two-step readiness process to ensure that the schools had met a threshold of readiness prior to beginning the intervention. First, CSPV conducted readiness feasibility visits to assess indicators including leadership’s support of SCSS and the presence of a school-based champion. Second, school-based teams from 21 schools completed a 90-item readiness assessment annually to assess constructs such as the degree to which leadership has created structures that support SCSS implementation (Leadership support) and expect, reward, and support the SCSS model (Priority).

**Findings:** 46 of 60 schools met the readiness criteria and agreed to participate. Two years of readiness assessment data suggest that some changes in readiness occurred in the expected direction, with significant increases for 6 of 20 readiness subscales including staff capacity, *t*(215)= 2.26, *p* = .025, knowledge and skills related to SCSS, *t*(212)= 3.91, *p* < .001, leadership support for SCSS, *t*(213)= 3.44, *p* = .001, and priority to implement SCSS *t*(200)= 2.37, *p* = .182. The data also indicate that certain readiness components might not be as malleable or addressed within this type of comprehensive approach (such as general leadership: Means=6.00 in Year 1, 6.11 in Year 2; and resource utilization: Means =4.49 in Year 1, 4.38 in Year 2).

**Implications for D&I Research:** Two years of data from a randomized controlled trial of SCSS suggest a feasible approach to determine the readiness of schools interested in implementing a comprehensive evidence-based prevention focused framework. Future work should study the extent to which readiness components can be altered by continued training and technical assistance and explore the sustainability and dissemination of SCSS, to ensure continued engagement of school stakeholders and other key stakeholders.

**Primary Funding Source**

National Institutes of Health

### S146 Resources and school culture are associated with readiness for implementation of universal prevention programs in rural schools

#### Lindsey Turner, Hannah Calvert, Katie Bubak-Azevedo, Carl Siebert

##### Boise State University, Boise, ID, USA

###### **Correspondence:** Lindsey Turner (lindseyturner1@boisestate.edu)

**Background:** Organizational readiness for implementation of evidence-based best practices is an important predictor of successful implementation outcomes. Rural schools have been found to struggle with implementation of systems changes and evidence-based practices; however, organizational settings such as rural schools are generally under-studied.

**Methods:** We gathered survey data from 159 school staff who were part of the newly-formed implementation teams at 40 rural public schools, prior to the implementation of Tier 1 Positive Behavioral Interventions and Supports (PBIS), a universal prevention strategy. Goals of the current analyses were to examine how the availability of resources and structural supports were associated with organizational readiness for change. We used a 6-item measure of change based on the Organizational Readiness to Implement Change scale (alpha=.94). Analyses accounted for clustering within school.

**Findings:** Perceived school-wide readiness was significantly lower for PBIS team members who indicated they did not have adequate resources for implementing behavior interventions (P<.001), did not have adequate resources for monitoring student behavior (P<.01) and did not have enough time to implement positive behavior support systems (P<.001). Perceived organizational readiness was lower among respondents who indicated that their school’s behavior management process was bureaucratic and overly complex (P<.01), but readiness was higher among respondents who indicated that using evidence-based practices is consistent with the culture of their school (P<.001).

**Implications for D&I Research:** Results show the importance of assessing and considering structural resources, staff time and scheduling flexibility, and aspects of school culture when planning for the implementation of universal prevention programs such as PBIS.

**Primary Funding Source**

National Institute of Justice

### S147 Integrating evidence-based approaches for social and organizational risk management in a school setting: A randomized trial on implementation effectiveness

#### Lydia Kwak^1^, Christina Björklund^1^, Maria Boström^1^, Gunnar Bergström^1,2^, Lotta Nybergh^1^, Liselotte Schäfer Elinder^1,3^, Kjerstin Stigmar^4,5^, Charlotte Wåhlin^6^, Irene Jensen^1^

##### ^1^Karolinska Institute, Stockholm, Sweden; ^2^University of Gävle, Gävle, Sweden; ^3^Stockholm Region, Stockholm, Sweden; ^4^Lund University, Lund, Sweden; ^5^Skåne University Hospital, Lund, Sweden; ^6^Linköping University, Linköping, Sweden

###### **Correspondence:** Lydia Kwak (lydia.kwak@ki.se)

**Background:** The high prevalence of mental ill-health among teachers underscores a need for more sustainable working conditions for this group. One way to achieve this is by improving schools’ social and organizational risk management. Many schools in Sweden lack a structured approach to manage occupational risks. In 2015, we launched an occupational mental health guideline to support evidence-based management of these risks at the workplace. The aim of this cluster-RCT was to compare the effectiveness of two strategies for implementing the guideline within schools with high fidelity. Effectiveness is measured as changes in guideline adherence (implementation effectiveness) and changes in social and organizational risk factors for mental ill health (intervention effectiveness).

**Methods:** Twenty schools in two municipalities in Sweden participate in the trial. Control-schools received a one-day training. Intervention schools received the training, formation of implementation teams and workshops. Effectiveness is measured as guideline adherence. Process outcomes, and barriers and facilitators influencing the implementation were assessed. Data were collected at baseline, 6, 12, 18 and 24 months and analyzed by a mixed-methods approach (i.e. survey, interviews, observation; last measurement is scheduled for September 2019).

**Findings:** Results will be presented on the implementation effectiveness and contextual factors influencing implementation. The study participants (n=705) were 76 % women and 45.9 years of age (*SD* = 11.84). Survey results show that baseline adherence to the guideline was low; 18 % of participants acted in accordance to the schools’ work environment policies. Furthermore, 20 % reported that their principal has knowledge on how the work environment influences mental ill health and 16 % reported that an action-plan was developed based on the latest systematic work environment survey. Detailed data on guideline adherence will be presented for 12- and 24 months follow-up. In addition, results on process outcomes, barriers and facilitators will be shown.

**Implications for D&I Research:** The project will provide valuable knowledge for researcher, policy makers and schools on the impact of implementation strategies for integrating evidence-based approaches for social and organizational risk management in a workplace setting in order to prevent mental ill health. Moreover, knowledge is gained on contextual factors that influence implementation.

**Primary Funding Source**

AFA-insurance

### S148 How well are we doing? stakeholder perspectives on implementing evidence-based interventions to increase colorectal cancer screening.

#### Lauren Workman, Heather Brandt, Hiluv Johnson, Elijah Christian, Dave Murday

##### University of South Carolina, Columbia, SC, USA

###### **Correspondence:** Lauren Workman (WORKMANL@mailbox.sc.edu)

**Background:** The Colorectal Cancer Screening Program in South Carolina (CCSPSC) works with federally-qualified health centers (FQHCs) to implement evidence-based interventions (EBIs) to increase colorectal cancer screening (CRCS). The program’s primary implementation strategies include 1) an assessment of readiness and context to identify factors that may inhibit or promote implementation, 2) development of tailored implementation plans, and 3) provision of educational sessions and technical assistance using a “whole office” approach. We will describe our approach to understand stakeholder perceptions of how well those strategies are working to encourage use of EBIs to promote CRCS and integrate them into the culture of partner FQHCs.

**Methods:** Based on Proctor et al.’s (1) Implementation Outcomes framework, we assessed implementation outcomes on three dimensions: *acceptability, appropriateness, and feasibility.* We matched evaluation questions to each dimension and used stakeholder interviews and a provider survey as data sources. Interview data were coded using an inductive approach. We calculated descriptive data for provider survey items. A concerted focus on the primary implementation strategies was used to guide analysis in relation to the implementation outcomes.

**Findings:** Interview data revealed the whole office approach has high *acceptability*, as it generates buy in, awareness, and enthusiasm for the need to increase CRCS. In addition, stakeholders perceived the program as *appropriate* because readiness and implementation data were used to adapt implementation approaches and selection of EBIs to each FQHC. Further, provider survey data indicated that CRCS is an important priority and flexible implementation support from the CCSPSC is highly valuable. However, more than one-third of providers characterized the *feasibility* of using and maintaining their FQHC’s selected EBIs as somewhat hard or very hard. Providers cited several barriers including insufficient patient transportation, costs for direct visualization screenings, and long wait times for referral completion.

**Implications for D&I Research:** Given the large scale of the program and varied contexts of each partner FQHC, it is important to understand the acceptability, appropriateness, and feasibility of the program’s strategies and goals. These data allow us the opportunity to assess and enhance aspects of our program to promote the relevance, sustainability, and potential replication of strategies to improve CRCS.

**Primary Funding Source**

Centers for Disease Control and Prevention

**Reference**

1. Proctor E, Silmere H, Raghavan R, Hovmand P, Aarons G, Bunger A, Griffey R, Hensley M (2011). “Outcomes for implementation research: conceptual distinctions, measurement challenges, and research agenda”. *Adm Policy Ment Health*. 38: 65–76.

### S149 Why implementation fails: Perspectives of teachers who do not implement classroom based physical activity (and those who do)

#### Lindsey Turner, Julianne Wenner, Hannah Calvert

##### Boise State University, Boise, ID, USA

###### **Correspondence:** Lindsey Turner (lindseyturner1@boisestate.edu)

**Background:** Classroom-based physical activity (CBPA)—including brief instructional breaks for movement, or the integration of physical activity with lessons—can benefit children’s physical, social, emotional, and cognitive health. Teachers are key implementation agents. Despite CBPA being considered a best-practice, many classroom teachers do not regularly use it. Much research has explored teachers’ perspectives on CBPA, but almost none has explored why implementation fails. The perspectives of teachers who do not implement CBPA can contribute important information about potential strategies to promote implementation.

**Methods:** These analyses use 24 teacher interviews, gathered as part of a mixed-methods evaluation of a CBPA intervention at five elementary schools at which all classroom teachers received CBPA training. Implementation was monitored through weekly tracking logs completed by all teachers over 14 weeks. Many teachers tried CBPA initially, but some sustained it consistently whereas others did not. After the tracking period, interviews were conducted with a subset of 12 teachers who were consistently high implementers (HI) and 12 who were low implementers (LI). Interviews were transcribed and coded thematically.

**Findings:** While HI and LI both recognized the value of CBPA for student well-being and focus in the classroom, several key differences were noted. HI were more likely to write CBPA into their daily schedule and to have a cooldown routine for allowing students to settle and transition back to schoolwork. They had principals who were proactive and explicit supporters of CBPA, and they shared ideas about CBPA with their colleagues. In contrast, LI did not have these environmental supports. Lack of time is a common challenge for implementation of any classroom initiative; specific to CBPA, LI were more hindered by it as a barrier, and while HI had found solutions, LI had concerns but no solutions.

**Implications for D&I Research:** As with other innovations, the implementation of CBPA is facilitated by the presence of proactive leadership/champions. Knowledge of the “why” of behavior change is crucial, but a key barrier seems to be “how” to implement. Coaching teachers in specific strategies for time management (e.g., explicit scheduling) and behavior management (e.g., cooldown routines) may facilitate implementation among teachers who would not otherwise do so.

**Primary Funding Source**

Institute of Education Sciences

### S150 Individual-level factors related to the implementation of school-based physical activity approaches: Perspectives from multiple stakeholders

#### Jacob Szeszulski^1,2^, Michael Robertson^3^, Timothy Walker^1^

##### ^1^University of Texas Health Science Center at Houston, Houston, TX, USA; ^2^University of Texas Health Science Center at Austin, Austin, TX, USA; ^3^University of Texas MD Anderson Cancer Center, Houston, TX, USA

###### **Correspondence:** Jacob Szeszulski (Jacob.Szeszulski@uth.tmc.edu)

**Background:** Physical activity (PA) is a health-promoting and often required part of the elementary school day, but multiple stakeholders are responsible for implementing PA approaches (e.g., recess, physical education, active learning). Health fitness (HF) teachers generally view PA favorably, but other critical stakeholders may have less positive perceptions about implementing PA approaches. The purpose of this study was to compare different stakeholders’ perceptions of individual-level determinants related to implementation of school-based PA approaches.

**Methods:** School staff (*n*=139), including principals/assistant principals (*n*=21), HF teachers (*n*=41), and non-HF teachers (*n*=77), from public elementary schools (*n*=25), completed surveys measuring perceptions of individual-level determinants for implementing school-based PA approaches. Fourteen items (attitudes [4 items], barriers [3 items], knowledge [2 items], and outcome expectations [5 items]) were scored on 5-point Likert-type scales (strongly disagree to strongly agree) and averaged to ascertain determinant scores. Determinants were compared between job types using ANOVAs.

**Findings:** Principals (95.2%) and non-HF teachers (95.7%) were a higher proportion female than HF teachers (65.9%; *p*<.001). Mean age (43.4±12.0 years) was not significantly different between job types. Principals (22.0±7.1) had been in education more years than HF (15.0±10.7; *p*=.017) and non-HF (14.6±9.2; *p*=.005) teachers. HF (9.8±9.8) teachers were more likely to have held the same position longer than non-HF teachers (6.0±6.9; *p*=.029) and principals (4.5±3.5; *p*=.028). On average, non-HF teachers (2.3±0.8;*d*=0.40;*p<.*001) and principals (2.2±0.7;*d*=0.26;*p*=.005) reported more implementation barriers for PA promotion, compared to HF teachers (1.5±0.8). Non-HF teachers (4.6±0.6) also had lower outcome expectations for PA, compared to HF teachers (4.8±0.3*;d*=0.13;*p=.*006), which were not significantly lower than principals’ outcome expectations (4.7±0.4;*d*=0.05;*p*>.05). Non-HF teachers had lower PA knowledge (3.3±0.9) then both HF teachers (4.0±0.8;*d*=0.25;*p*<.001) and principals (4.0±0.7;*d*=0.27;*p*=.005). No differences in attitudes about PA existed between job types.

**Implications for D&I Research:** Stakeholder groups have unique perceptions of individual-level determinants related to implementation of PA approaches, which are less favorable for non-HF teachers. Differences in determinants suggest tailored implementation strategies are needed for different stakeholders’ job types, and this study provides information on which determinants tailored approaches can target. Future studies are needed to determine how differences in individual-level determinants impact implementation outcomes.

**Primary Funding Source**

National Institutes of Health

### S151 Stakeholder engagement in implementing a population health management intervention to address uncontrolled cardiovascular disease risk factors

#### Karen Goldstein^1^, Allison Lewinski^1^, Hayden Bosworth^1,2^, Jennifer Gierisch^2^, Courtney White-Clark^1^, Felicia McCant^1^, Leah Zullig^1,2^

##### ^1^Durham VA Health Care System, Veterans Health Administration, Durham, NC, USA; ^2^Duke University, Durham, NC, USA

###### **Correspondence:** Karen Goldstein (karen.goldstein@duke.edu)

**Background:** Implementing population-level interventions within a healthcare system requires longitudinal stakeholder involvement and multidisciplinary expertise. We sought to improve cardiovascular disease (CVD) risk among rural Veterans via an intervention addressing multi-level barriers including provider (competing demands), patient (risk awareness), and system (under-empowered teams).We describe a multidisciplinary team approach to intervention adaptation and implementation informed by structured consultation with key stakeholders.

**Methods:** Our team included a: behavioralist, clinical interventionist, primary care physician, implementation scientist, nurse scientist, project coordinators, and a clinical nurse. We developed an intervention to: 1) identify patients with uncontrolled blood pressure and/or with hyperlipidemia but not on statin therapy; 2) activate patients via personalized letters; 3) identify behavioral goals guided by patient preferences; and, 4) communicate patient goals and suggested CVD risk reduction plan to primary care. Stakeholder engagement activities included: input from existing CVD programs, feedback from Veterans Research Engagement Panel, and clinic-level debriefings. Our biweekly intervention debriefs were guided by the Stirman Adaptation Framework.

**Findings:** To date, 100 patients received activation letters. Of that number our population health manager was able to speak with 69; 38/46 Veterans kept scheduled appointments with their providers after receiving the intervention, and 33/54 Veterans that did not have a scheduled appointment with a provider made one. Notably, after the intervention, 54/100 Veterans initiated contact with a healthcare provider (ie, primary care physician or specialist) to have their blood pressure checked, medications modified, or obtain a referral for another provider or service (e.g., weight loss, smoking cessation). In addition, we identified critical adaptations to facilitate uptake within VA clinical practice including message tailoring, timing of activation letters, and alignment with ongoing primary care practices.

**Implications for D&I Research:** A collaborative team of multidisciplinary researchers can inform intervention adaptation, build stakeholder buy-in, and facilitate the development of a sustainable intervention within a clinical practice setting. The VA is an ideal venue for team-based implementation science and real-time adaptation of population-level interventions. Our project lays an important foundation for developing rigorous and relevant interventions that will assure that rural Veterans have access to effective, context-appropriate CVD risk management options.

**Primary Funding Source**

Department of Veterans Affairs

### S152 Understanding multilevel barriers and facilitators of cascade screening for lynch syndrome

#### Megan Roberts^1^, Swetha Srinivasan^2^, Alanna Kulchak Rahm^3^, Heather Hampel^4^, Daniel Reuland^5^, Amit Patel^6^, Jennifer Leeman^7^

##### ^1^University of North Carolina at Chapel Hill Eshelman School of Pharmacy, Chapel Hill, NC, USA; ^2^University of North Carolina at Chapel Hill, Chapel Hill, NC, USA; ^3^Genomic Medicine, Geisinger, Danville, PA, USA; ^4^Ohio State Wexner Medical Center, Columbus, OH, USA; ^5^The University of North Carolina School of Medicine, Chapel Hill, NC, USA; ^6^Medical Marketing Economics, LLC, Oxford, MS, USA; ^7^School of Nursing, University of North Carolina, Chapel Hill, NC, USA

###### **Correspondence:** Megan Roberts (megan.roberts@unc.edu)

**Background:** Cascade screening (i.e., genetic testing) among families affected by hereditary cancer disorders, such as Lynch syndrome (LS), is suboptimal and understudied. The overall objective of this project was to identify multilevel barriers and facilitators to the implementation of cascade screening for LS.

**Methods:** We conducted semi-structured, in-depth interviews to identify implementation barriers and facilitators for cascade screening among key stakeholders (n=60): patients (n=20); genetic counselors (n=10) and non-genetics providers (n=10) including oncologists, gastroenterologists, gynecologists, and general practitioners; genetic testing companies (n=5), advocacy organizations (n=5), managed-care payers (n=5), and genetics clinic administrators (n=5). The interview guide and analyses were informed by the Consolidated Framework for Implementation Research. Transcripts were double-coded (20% sample) using template analysis in Atlas.ti.

**Findings:** Barriers identified across all stakeholders included: (1) accessibility of genetic counseling services and workforce shortages, (2) emotional barriers impacting family communication such as stigma, guilt, avoidance, estrangement and dysfunction, (3) lack of education and awareness about LS by patients and providers, and (4) fear of a positive screening result impacting employment, career progression, and eligibility for health and life insurance. Patient and family members perceived cost as a barrier for pursuing genetic testing; however, other stakeholders highlighted the importance of communicating to patients that cost need not be a barrier, citing free genetic testing programs and broad insurance coverage. Additional clinic-level barriers were identified by providers in clinical workflows such as lack of processes to identify probands (initial relative diagnosed with LS) or regular follow-up with probands. Key facilitators identified across all stakeholders included: (1) free testing offered by genetic testing laboratories for family members of probands, (2) motivation to inform family members and undertake preventive health measures, and (3) connecting to online resources such as patient support groups.

**Implications for D&I Research:** These findings indicate that modifiable, multilevel barriers to the implementation of cascade screening are experienced across stakeholders. Findings from this study will inform an intervention to be tested in a future hybrid effectiveness implementation trial. As precision prevention takes hold, understanding implementation barriers to genetic testing will be imperative to ensure that these innovations are accessible to patients.

**Primary Funding Source**

National Institutes of Health

### S153 Identifying and applying implementation strategies to translating an evidence-based exercise intervention to a rural community cancer care organization

#### Laura Rogers^1^, Haiyan Qu^1^, Michelle Martin^2^, Jessica Adams^1^, Maria Pisu^1^, Richard Shewchuk^1^, Robert Oster^1^, Ana Baumann^3^

##### ^1^University of Alabama at Birmingham, Birmingham, AL, USA; ^2^University of Tennesse Health Science Center, Memphis, TN, USA; ^3^Washington University in St. Louis, St. Louis, MO, USA

###### **Correspondence:** Laura Rogers (rogersl@uab.edu)

**Background:** Rural cancer survivors are less physically active and suffer poorer health and quality of life, a disparity potentially reduced by exercise programming. Furthermore, available evidence-based interventions (EBIs) related to exercise and cancer survivorship are rarely translated and little is known about how to optimize this translation.

**Methods:** During development of an implementation toolkit for delivering an exercise EBI for women cancer survivors by non-research staff in a rural community cancer care organization, we collected multilevel focus group and nominal group technique (NGT) data from rural cancer survivors (n=19), potential interventionists (n=11), and community/organizational stakeholders (n=19). These data were coded to identify implementation strategies and Consolidated Framework for Implementation Research (CFIR) constructs important to the anticipated EBI implementation. Findings were integrated into the toolkit.

**Findings:** Integration of data across the participant types and the two different data collection methodologies, yielded multiple implementation strategies including but not limited to promoting program adaptability to accommodate rural resident and cancer survivor needs, obtaining primary care physician buy-in, building community coalitions, seeking philanthropic funds, providing EBI recipient transportation, etc. Moreover, we consistently identified 11 CFIR constructs as important along with relevant suggested strategies for targeting these constructs as demonstrated by the following examples: 1) Intervention Design Quality and Packaging (e.g., home-based options, exercise variety, etc.), 2) Implementation Readiness (“cheat sheets”, expert facilitation, etc.), and 3) Cosmopolitanism (e.g., donations from retailers, provide transportation by partnering with churches with vans, etc.). We also identified differences between participant types with regard to relative importance of CFIR constructs (e.g., Patient Needs and Resources was more important for cancer survivors, Evidence Strength and Quality was more important for interventionists, and Cosmopolitanism was more important for interventionists and stakeholders). Hence, suggestions for targeting these constructs using the identified strategies were included in the implementation toolkit.

**Implications for D&I Research:** The identified strategies for improving the translation of an exercise and cancer EBI also hold potential relevance to translation of other multicomponent, expertise-depending EBIs for rural cancer survivors. Further research is needed to determine the most effective implementation strategies, potential mediating role of identified CFIR constructs in implementation success, and applicability of these strategies to other similar EBIs.

**Primary Funding Source**

National Institutes of Health

### S154 Strength after breast cancer: An evidence-based rehabilitative exercise program for breast cancer survivors

#### Kathryn Schmitz^1^, Rinad Beidas^2^, Fran Barg^2^, William Calo^1^

##### ^1^Penn State College of Medicine, Hershey, PA, USA; ^2^University of Pennsylvania, Philadelphia, PA, USA

###### **Correspondence:** Kathryn Schmitz (Kzs95@psu.edu)

**Background:** Study 1. A hybrid Type 1 effectiveness-implementation trial sought to quantitatively assess whether an evidence-based exercise intervention for breast cancer survivors, Strength After Breast Cancer, was safe and effective in a new setting and to qualitatively assess barriers to implementation. Study 2. Upon completion of this trial, we created and evaluated a training to prepare clinicians to deliver the program.

**Methods:** Study 1. A cohort of 84 survivors completed measurements related to limb volume, muscle strength, and body image at baseline, 67 survivors completed measurements 12 months later. Qualitative methods were used to understand barriers to implementation experienced by referring oncology clinicians and physical therapists who delivered the program. Study 2. A total of 455 clinicians that had completed the training were sent an email inviting them to participate in a survey.

**Findings:** Study 1. Similar to the efficacy trial, the revised intervention demonstrated safety with regard to lymphedema, and improvements in lymphedema symptoms, muscular strength, and body image. Qualitative implementation data suggested significant implementation barriers around intervention characteristics, payment, eligibility criteria, the referral process, the need for champions (ie, advocates), and the need to adapt during implementation of the intervention, which should be considered in future dissemination and implementation efforts. Study 2. A total of 75 clinicians completed the online survey. All of these had attempted to implement the program in their clinics, 80% had succeeded in carrying out at least part of the program. Key barriers to implementation in practice was 3^rd^ party coverage and a difference in the way the program was enacted from the way their practice delivered care.

**Implications for D&I Research:** Study 1. This trial successfully demonstrated that Strength After Breast Cancer can be implemented in a community setting while retaining the effectiveness and safety. However, strategies to reduce barriers to implementation are required. This new program can inform larger scale dissemination and implementation efforts. Study 2. For Strength After Breast Cancer to be more robustly implemented, there is a need for more than just clinician training. Future research is needed to discern the implementation strategies that would be most effective.

**Primary Funding Source**

National Institutes of Health

### S155 Active living after cancer: Adaptation for community and clinic

#### Karen Basen-Engquist, Maria Swartz, Leticia Gatus

##### The University of Texas MD Anderson Cancer Center, Houston, TX, USA

###### **Correspondence:** Karen Basen-Engquist (kbasenen@mdanderson.org)

**Background:** Active Living after Cancer (ALAC) is a program to increase physical activity and improve quality of life in breast cancer survivors. It is based on an evidence-based program for the general public (Project Active) tested in a large RCT (Dunn et al, 1997) and was adapted for breast cancer survivors and subsequently implemented in community and clinical settings. In this presentation we will present three phases of program adaptation, using adaptation steps described in a scoping review of adaptation frameworks (Esoffery et al 2019), and the data driving adaptation decisions, and the effectiveness of the adapted intervention.

**Methods:** Phase 1 was the adaptation of the Project Active intervention for breast cancer survivors, guided by interview and questionnaire data from breast cancer survivors, and with effectiveness tested in a small RCT. Phase 2 was adaptation for broader community delivery of the program with a focus on medically underserved populations, guided by community stakeholder input, and tested in a pre-post evaluation. Phase 3 was the adaptation of the program for a clinical setting with the incorporation of a game-based element, evaluated in a small pilot test.

**Findings:** The Phase I adaptation steps included assessing the community, consulting with stakeholders, deciding what needed adaptation, adapting the original program, implementing and evaluating. Participants receiving the adapted program had significantly better physical functioning, and quality of life at the end of the program than a standard care control group. The Phase II adaptation included consulting with stakeholders, deciding what needed adaptation, adapting the original program, implementing and evaluating. In the pre-post evaluation, the 211 participants demonstrated significant increases in physical activity and improvements in physical functioning and quality of life, with high ratings of acceptability. In Phase 3 investigators consulted with stakeholders and adapted the intervention and pilot tested it. The pilot test identified technology implementation issues, but also demonstrated the high acceptability of the program and preliminary evidence of improvements in physical functioning.

**Implications for D&I Research:** Careful use and incorporation of information from cancer survivors and stakeholders in the adaptation process can positively influence program acceptability and feasibility, as well as outcomes.

**Primary Funding Source**

Cancer Prevention & Research Institute of Texas

## Promoting Health Equity and Eliminating Disparities

### S156 Assessing implementation of “style” for African American men with HIV: Using the consolidated framework for implementation research (CFIR) in a community participatory study

#### Janet Myers^1^, Greg Rebchook^1^, Rob Newells^2^, Marguerita Lightfoot^1^

##### ^1^University of California, San Francisco, San Francisco, CA, USA; ^2^AIDS Project of the East Bay (APEB), Oakland, CA, USA

###### **Correspondence:** Janet Myers (janet.myers@ucsf.edu)

**Background:** HIV diagnoses increased 87% among young African American gay and bisexual men from 2005-2014. CDC estimates that 1 in 2 African American gay and bisexual men will be diagnosed with HIV in their lifetime. Enhanced engagement in HIV healthcare among Black and Latino MSM living with HIV is vital to reduce disparities, improve health, and reduce HIV transmission. STYLE, a model program to improve engagement in healthcare among young MSM of color, has been successful. However, little work has been conducted to study the implementation of STYLE in real-world settings.

**Methods:** We adapted STYLE implementation materials for use in the local environment and population and assessed fidelity of STYLE to the original methods, while adapting it to the local context (process outcomes). We examined whether adapted STYLE achieved similar clinical outcomes as the original study, such as identifying previously undiagnosed cases of HIV, linkage to care, re-engagement in care, retention in care, and ART adherence (care outcomes). We also followed the Consolidated Framework for Implementation Research to investigate the domains relevant to the adaptation and implementation process.

**Findings:** With regard to the intervention, comparing STYLE's core components to current CBO programming resulted in the CBO: adopting new activities to implement and also some adaptation of STYLE’s core components to reflect the needs of the local population and HIV epidemic. Inner-setting issues affecting implementation included CBO challenges to managing staff turn-over and integrating a new EMR system into evaluation activities. Changing community demographics (outer setting) are driving the target population away from the catchment area, Oakland, where most services are currently provided. We found that intervention tailoring was necessary to address the needs of the local population's culture and broader age range.

**Implications for D&I Research:** Conducting community engaged research to adapt and implement an intervention must explicitly acknowledge the equal (though not identical) expertise of researchers and community providers. A process involving bi-directional exchange of information and ideas helps. Implementation frameworks can support community engaged research and once a productive working relationship between community and academic partners is established, benefits all aspects of implementation and dissemination.

**Primary Funding Source**

Centers for Disease Control and Prevention

### S157 Developing a multi-component strategy to scale-up a community health worker intervention: Findings from a hybrid effectiveness-implementation study

#### Carmen Samuel-Hodge^1^, Jennifer Leeman^2^, Sallie Allgood^3^, Samuel Cykert^1^

##### ^1^University of North Carolina, Chapel Hill, NC, USA; ^2^School of Nursing, University of North Carolina, Chapel Hill, NC, USA; ^3^Duke University, Durham, NC, USA

###### **Correspondence:** Jennifer Leeman (jleeman@email.unc.edu)

**Background:** Community Health Workers (CHW) are effective at reaching underserved populations. Evidence now is needed on implementation strategies that are effective at taking CHW interventions to scale in settings that reach those most affected by health disparities. To address this gap, we engaged public health and community clinic partners in the design of a multi-component strategy to implement a CHW-delivered lifestyle intervention in two predominantly African American, rural counties that rank 80^th^ and 98^th^ (of 100 NC counties) for healthy behaviors. We tested the strategy in one county, refined it, and then replicated it in the second county.

**Methods:** This 5-year study (2014-2019) applied a community-engaged, single-arm, hybrid effectiveness-implementation trial design. Within each county, CHWs in public health departments and community clinics delivered four in-home counseling sessions and three phone calls and referred participants to community resources and services. Implementation strategies included identifying barriers and facilitators, building a coalition, developing a formal implementation blueprint, adapting educational materials, and providing training and supervision. The RE-AIM framework guided measures of effectiveness and implementation. Data collection included biological measures, validated surveys, and an online system for tracking enrollment and intervention delivery.

**Findings:** The intervention reached 386 (30.5% of those invited) and retained 341 (88.3% of those enrolled). The intervention was effective, with significant mean improvements in dietary intake (↑0.88 servings fruits/vegetables, ↓0.3 servings soda per week), physical activity (↑43 minutes per week), weight (60% losing a mean of 2 pounds), and a 20% point reduction in participants with uncontrolled blood pressure. CHWs’ fidelity was mixed (content delivered, goals set, and referrals made) and was improved in the replication site. Although a mean of 1.3 referrals was made per visit, participants acted on only 40% of these referrals.

**Implications for D&I Research:** Findings and lessons learned from county one guided the refinement of implementation strategies used in the second county. A final product of this study is a replication toolkit that includes ready-to-use materials and detailed “how-to” guidance on both the intervention and the implementation strategies. Further research is underway to test scale-up across 20 health departments and community clinics, settings that reach those at greatest risk for poor health behaviors.

**Primary Funding Source**

Centers for Disease Control and Prevention

### S158 Implementing a multi-level electronic health record and community health worker intervention in immigrant-serving primary care practices to improve hypertension control among South Asian patients

#### Nadia Islam^1^, Radhika Gore^1^, Jennifer Zanowiak^1^, Laura Wyatt^1^, Sadia Mohaimin^1^, Priscilla Lopez^1^, Anna Divney^2^, Sahnah Lim^1^, Lorna Thorpe^1^

##### ^1^NYU School of Medicine, New York, NY, USA; ^2^CUNY School of Public Health, New York, NY, USA

###### **Correspondence:** Nadia Islam (nadia.islam@nyumc.org)

**Background:** Despite population-wide health efforts addressing hypertension (HTN), culturally-adapted multi-level strategies are needed for ethnic/minority communities facing HTN disparities. Project IMPACT tests the feasibility, adoption, and impact of integrating two evidence-based strategies - provider-level electronic health records (EHRs) interventions and patient-level community health worker (CHW) coaching - to improve HTN control among South Asians receiving care in 14 primary care practices in New York City.

**Methods:** Practices received EHR capacity-building support to identify and manage hypertensive patients. Patients with uncontrolled hypertension (n=304) were enrolled and randomized to treatment (n=159) or wait-list control groups (n=145) after completion of a CHW-led education session on HTN control. The treatment group participated in 4 additional in-language, culturally-adapted education sessions and 10 bi-monthly goal-setting phone calls. We conducted semi-structured interviews (n=36) with practice staff, CHWs, and a payer organization; directly observed clinic workflow; and administered practice-level surveys to examine barriers and facilitators to implementation processes and outcomes using Consolidated Framework for Implementation Research constructs.

**Findings:** Among the intervention group, there was a mean change of -4.5 mmHg in SBP (p<0.001) and -3.7 mmHg in DBP (p<0.001), while mean SBP and DBP increased in the control group (p>.05)). The intervention effect (adjusted for age and sex) for SBP and DBP was significant (p<0.001). In the intervention group, BP control (BP<140/90) increased from 37.3% at baseline to 69.7% at endpoint (p<0.001), while in the control group, BP control did not change. Fidelity to the culturally adapted CHW intervention was high; (88%) participated in at least 4 of the 5 sessions and an average of 7 goal-setting phone calls per participant were completed. A relationship between inner setting and outer setting factors influenced intervention impact. For example, EHR capacity-building led to new organizational practices, linked to CHW actions, that formalized clinics’ existing informal community knowledge and embeddedness. CHWs helped form connections between clinics, patients, and patients’ social networks, thus building social ties between clinic and community, though clinic staff limitations resulted in variability in extent of CHW integration.

**Implications for D&I Research:** We will discuss how study findings provide opportunities to translate and scale our model to address health disparities in ethnic/minority communities.

**Primary Funding Source**

Centers for Disease Control and Prevention

### S159 Predictors of 12-month implementation of the faith, activity, and nutrition (FAN) intervention in a statewide dissemination & implementation study: Application of the consolidated framework for implementation research (CFIR)

#### Sara Wilcox^1^, Danielle E. Jake-Schoffman^2^, Ruth P Saunders^1^, Brent Hutto^1^

##### ^1^University of South Carolina, Columbia, SC, USA; ^2^University of Florida, Gainesville, FL, USA

###### **Correspondence:** Sara Wilcox (wilcoxs@mailbox.sc.edu)

**Background:** Faith-based organizations, due to their broad reach and trust, are well-positioned to contribute to eliminating disparities. This D&I study examined 12-month implementation and predictors of implementation of FAN.

**Methods:** The university partnered with the SC Conference of the United Methodist Church to offer FAN to 986 churches; 115 churches (N=93 evaluated; 42% African American, 75% <500 members) adopted FAN and were trained by community health advisors. At baseline and 12 months, church FAN Coordinators completed implementation interviews regarding the four FAN components for physical activity (PA) and healthy eating (HE) (opportunities, policies, messages, pastor support). Potential predictors of implementation were selected based on CFIR guidance, and measured at baseline, immediate post-training, or 12 months, depending on training/program exposure needed for item relevance. Repeated measures ANOVAs tested change in PA and HE implementation composite scores, and Cohen’s *d* indicated magnitude of change. Mixed model linear regression tested whether CFIR items predicted 12-month implementation, controlling for baseline implementation.

**Findings:** PA and HE implementation increased significantly from baseline to 12 months (*d*=1.42 and 2.05, respectively). No constructs within the **intervention characteristics** domain predicted implementation. Within the **inner setting** domain, two structural characteristics predicted greater implementation: being an African American congregation (HE and PA) and having <500 members (HE). Networks & communication, relative priority, organizational rewards, readiness, and congregant needs also predicted PA and HE implementation. Culture and tension for change predicted PA but not HE implementation. Within the **characteristics of implementers** domain, identification with organization predicted PA and HE implementation. The personal attributes of self-efficacy and church membership duration predicted only PA implementation. Within the **implementation process** domain, engaging opinion leaders and engaging champions predicted PA and HE implementation. The directions of all associations were as expected.

**Implications for D&I Research:** The CFIR was used throughout the research process, with careful attention to timing of measures. Constructs within the inner setting and implementation process domains were most predictive of church-level implementation, and results will inform church trainings and technical assistance to minimize organizational barriers and promote enablers. Implementation of FAN was greater in African American churches, underscoring the potential of promoting health equity through this setting.

**Primary Funding Source**

Centers for Disease Control and Prevention

### S160 Utilizing implementation strategies to increase equity in classroom-based physical activity programming among low-resource schools

#### Rebecca Hasson, Andria Eisman, Daphne Watkins, Lexie Beemer, Tiwaloluwa Ajibewa

##### University of Michigan, Ann Arbor, MI, USA

###### **Correspondence:** Rebecca Hasson (hassonr@umich.edu)

**Background:** Proactively developing disparities-reducing implementation strategies are critical for the fidelity of classroom-based physical activity interventions in low-resource elementary schools. The purpose of this study was to determine the effectiveness of implementation strategies, guided by the Replicating Effective Programs (REP) framework, to motivate physical activity behavior change in students.

**Methods:** Nine, 3^rd^-6^th^ grade classrooms in one school in Detroit, Michigan (79% Latino; 80% qualified for free/reduced lunch) participated in Interrupting Prolonged sitting with ACTivity (InPACT), a 20-week intervention where teachers were asked to implement 5 x 4-minute moderate-to-vigorous physical activity (MVPA) breaks per day. A comprehensive set of strategies that targeted program packaging, teacher training, and technical assistance were developed: (1) a compendium of 200 MVPA breaks in compliance with health-enhancing exercise physiology principles; (2) evidence-based exercise videos that minimized out-of-school preparation time for teachers; (3) gamification of breaks to enhance student enjoyment; (4) teacher trainings to increase acceptability; (5) classroom management procedures to reduce transition time; and (6) floor plans that reorganized classroom furniture to reliably and safely increase children’s activity in classrooms. Teacher implementation was measured via a daily, weekly, and end-of-study questionnaire. Activity break duration and intensity was measured via direct observation.

**Findings:** Throughout the 20-week intervention period, 3^rd^-5^th^ grade teachers were able to implement 5 activity breaks per day at an average duration of 255 seconds*.* Sixth grade teachers were able to implement 2 activity breaks per day at an average duration of 249 seconds. These teachers noted that their rotating classroom vs. self-contained classroom was a primary barrier to implementation.

**Implications for D&I Research:** In order for evidence-based physical activity interventions to be successful, they must be complimented by effective implementation strategies. InPACT is the first classroom-based physical activity intervention tailored to meet the specific needs of low-resource schools by proactively developing disparities-reducing implementation strategies to motivate physical activity behavior change in students. Findings from this pilot study suggest, 5 x 4-minute MVPA breaks per day is a feasible dose of in-class activity that 3^rd^-5^th^ grade teachers can implement in low-resource school settings. Additional strategies are needed to maximize fidelity in 6th grade classrooms.

**Primary Funding Source**

University of Michigan MCubed Seed Funding Program

### S161 Identifying social barriers in the pre-implementation of physical activity interventions targeting latinos diagnosed with diabetes: Physical activity system of support (PASOS) pilot study

#### Sandra Echeverria^1^, Krista Kenney^2^, Jovanna Orozco^1^, Danusha Sanchez^1^, Towfiqul Alam^1^, Olu Jegede^2^, Dereck Deleon^2^

##### ^1^University of North Carolina Greensboro, Greensboro, NC, USA; ^2^Cone Health, Greensboro, NC, USA

###### **Correspondence:** Sandra Echeverria (seecheve@uncg.edu)

**Background:** Latinos living in the United States (U.S.) experience a disproportionate burden of diabetes and are less likely to adopt physical activity recommendations for proper diabetes management. We describe a pilot study integrating electronic health records (EHR), clinical referral systems, patient survey data, and community-based perspectives to identify pre-implementation barriers to physical activity adoption among an emerging Latino population living in the southern U.S.

**Methods:** This is a 3-stage pre-implementation study. First, we identified social determinants of health (SDOH) measures proposed in the field and determined how well these measures were integrated into EHR and patient referral systems of a large hospital system in North Carolina. Second, we surveyed Latino adults diagnosed with diabetes (n=75) receiving care within this hospital system to assess diabetes management stressors, financial barriers, housing stability, experiences of racial/ ethnic discrimination, healthcare access, work demands, neighborhood characteristics and preferences for a future physical activity intervention. We then examined associations between these SDOH measures and EHR-derived diabetes outcomes. Lastly, we will share our findings (projected October 2019) with local community stakeholders serving Latinos to determine how well these systems-based data sources (including patient surveys) correspond with their perceptions of implementation barriers to physical activity adoption.

**Findings:** The systems-based data and patient surveys revealed extensive social barriers in this emerging Latino population, with higher social disadvantage associated with poorer diabetes outcomes. We identified interpersonal, organizational (healthcare), and community-level barriers that must be considered as key pre-implementation barriers to intervention adoption. Community input will help establish the extent to which and how other measures not considered in this study (e.g. patient’s legal status, periodicity of data collection, family/ child barriers) need to be examined and adopted in intervention implementation.

**Implications for D&I Research:** The identification of social barriers in the pre-implementation phase of evidence-based interventions targeting Latinos remains understudied. This presentation demonstrates the feasibility of integrating various sources of data to design a pre-implementation ‘blueprint’ to address social determinants of health likely to influence intervention adoption and discusses next steps for full integration and implementation strategies.

**Primary Funding Source**

Internal university and hospital award

### S162 Adaptations to increase recruitment and retention in a multi-component heart disease prevention intervention for rural adults

#### Manorama Khare^1^, Kristine Zimmermann^2^, Craig Beintema^3^

##### ^1^University of Illinois College of Medicine, Rockford, Rockford, IL, USA; ^2^University of Illinois at Chicago, Chicago, IL, USA; ^3^Stephenson County Health Department, Freeport, IL, USA

###### **Correspondence:** Manorama Khare (mkhare1@uic.edu)

**Background:** Rural US residents experience disproportionately high heart disease incidence and mortality compared to non-rural, due in part to modifiable risk factors like poor diet and insufficient physical activity. Translational research on evidence-based practice to rural community settings to reduce heart disease risk is limited. The “Health Improvement Project” (HIP) is a multi-component initiative, implemented by a multi-sectoral coalition called Win With Wellness (WWW) in two rural, northwest Illinois counties. HIP aims to reduce Framingham Risk Scores through evidence-based, community-based interventions targeting adults age 30-72 with no heart disease history. During HIP’s early implementation, coalition and stakeholder input informed adaptations to increase recruitment, ensure reach across all at-risk adults, and facilitate participation.

**Methods:** HIP’s development was informed by lessons learned from a prior WWW intervention in the two counties. WWW’s community-based weight loss program (n = 297) was highly effective for completers; however, 70% dropped out and non-completers were disproportionately employed or of working age. To better reach this group, HIP targeted worksites with two program options: a weight-loss intervention and a theory-based health promotion program called Heart-to-Heart (HH). Optional text-messaging and physical activity tracker interventions were also offered to facilitate ongoing engagement.

**Findings:** Due to slow initial recruitment, HIP was modified during Year 1 to enhance recruitment and facilitate retention. To facilitate equity and promote community buy-in, WWW leveraged partner resources to include adults with a history of heart disease and those outside of the target age range in HIP. Currently, 130 adults have enrolled in HIP (85 in the weight-loss intervention, 45 in HH). Enrollment will continue through August 2019. To increase worksite enrollment and based on stakeholder feedback, we developed a HH web-based version to be launched in July 2019. Among HIP participants, 52 and 47 enrolled in the activity tracker and text-messaging interventions, respectively. Notably, the intervention team assists participants with limited technology access to facilitate their activity tracker participation.

**Implications for D&I Research:** Recruiting and retaining rural US populations in intervention research poses unique challenges. Successful implementation in rural community settings can be facilitated by working with a community coalition, incorporating stakeholder feedback, and adapting interventions to be accessible to target communities.

**Primary Funding Source**

Health Resources and Services Administration

### S163 Understanding determinants of physical activity adoption and maintenance in rural cancer survivors: A qualitative study to guide future implementation

#### Scherezade Mama^1^, Nishat Bhuiyan^1^, Anne Marie Melda^1^, Kathryn Schmitz^2^

##### ^1^The Pennsylvania State University, University Park, PA, USA; ^2^Public Health Science, Penn State College of Medicine, Hershey, PA, USA

###### **Correspondence:** Scherezade Mama (skmama@psu.edu)

**Background:** Rural breast cancer survivors (BCS) are less likely to meet physical activity (PA) recommendations than those residing in urban areas. Ecologic frameworks account for multilevel factors related to physical activity (PA) adoption and maintenance and may be used to contextually adapt interventions to meet the unique needs of underserved rural BCS. We explored ecologic determinants of leisure-time PA in rural BCS to inform PA intervention implementation efforts in rural communities.

**Methods:** An interview guide was developed using an ecologic framework and asked about individual, social, and environmental determinants of leisure-time PA adoption and maintenance. Semi-structured in-depth interviews were completed with 38 rural BCS in central Pennsylvania. Interview transcripts were analyzed by three independent coders using thematic content analysis and a constant comparison approach.

**Findings:** Participants were in their early 60s (*M* age=62.0±14.7 years) and overweight (*M* BMI=28.6±6.2 kg/m^2^); more than half completed college or more (65.8%) and reported an annual household income ≥$80,000. Most (87.9%) participants were at least 12 weeks but less than 5 years post-treatment, and 45.9% did not meet PA recommendations. Participants were largely aware of the importance of PA for cancer survivorship and discussed individual and social determinants that influenced PA adoption, including their history with PA, engagement in social activities or networks, and social support generally and for PA, specifically. Women described multilevel barriers for engaging in PA, including lack of support and physical and financial resources along with treatment-related barriers (e.g., pain, fatigue). Rural community characteristics that limited PA adoption and maintenance included a lack of places nearby to do PA and feeling isolated from people and places. Rural BCS highlighted the need for opportunities to socialize and connect with others locally while being active to improve survivorship outcomes.

**Implications for D&I Research:** Qualitative findings from this study highlight the multilevel, interactive complexities that influence PA in rural BCS, emphasizing the need for a more sophisticated, ecologic approach for increasing PA adoption and maintenance in this underserved population. Insights gleaned from this study aid our understanding of the unique needs of rural BCS and will guide adaptation of an evidence-based PA interventions for implementation within rural communities in central Pennsylvania.

**Primary Funding Source**

National Institutes of Health

### S164 Adapting, implementing, and evaluating an evidence-based community intervention to promote physical activity in a midsize rural town

#### Barbara Baquero^1^, Rebecca Bucklin^2^, Natoshia Askelson^2^, Nicole Novak^2^, Rima Afifi^2^, Christine Kava^1^, Sandy Berto^3^, Active Ottumwa Community Advisory Board (CAB)^4^, Edith Parker^2^

##### ^1^University of Washington, Seattle, WA, USA; ^2^University of Iowa, Iowa City, IA, USA; ^3^University of Iowa, Ottumwa, IA, USA; ^4^Active Ottumwa, Ottumwa, IA, USA

###### **Correspondence:** Barbara Baquero (bbaquero@uw.edu)

**Background:** There is a strong body of evidence-based interventions (EBIs) to increase physical activity (PA), but levels of PA at the population level are still low, with widening disparities among racial/ethnic, low-income, and rural communities. Most PA interventions are designed for urban contexts, therefore leaving the 60 million residents of the rural US with fewer effective EBIs to promote PA. To address these gaps we adapted, implemented, and evaluated an intervention for PA to a midsize rural (micropolitan, population core ≤50,000) community in Iowa.

**Methods:** We used a hybrid type I study design to adapt, implement, and evaluate the effectiveness of an intervention in a micropolitan community using a community-based participatory research approach. Community stakeholders served as advisors in the intervention, and we applied an adaptation-planning tool to guide the fit, acceptability, and importance of each adaptation. The intervention integrated strategies from informational, behavioral, social, and environmental change EBI approaches. Our evaluation followed the RE-AIM framework. The population-based evaluation comprised of a cohort of community residents followed over 24-months, a cross-sectional survey of community residents at baseline and at 24 months, and the systematic collection of process and implementation data.

**Findings:** We implemented informational, behavioral, social, and environmental strategies; for example, we distributed over 110 activity calendars, and held on average 37 free PA activities monthly. We reached over 4,000 individuals, and delivered a six-month communication campaign, including 425 radio spots, that made over 200,000 impressions on community members. We recruited and trained 53 lay health advisors (LHA), and on average, we had 12 LHA active each month of the intervention. Over 25 organizations adopted free PA activities classes led by LHA. The intervention was effective in reducing sedentary behavior and increasing light PA. We have maintained the intervention over 10 months beyond the study period.

**Implications for D&I Research:** This study demonstrated that adapting, implementing, and evaluating evidence-based strategies to promote PA and reduce health disparities in rural communities is feasible and effective. We argue that, participatory implementation science can advance and facilitate implementation of EBIs at the population level, while retaining the scientific integrity of the evidence and rigorously evaluating the implementation and its effectiveness.

**Primary Funding Source**

Centers for Disease Control and Prevention

### S165 Amplification of school-based strategies resulting from the application of the dynamic adaptation process to reduce sexual and gender minority youth suicide

#### Lara Gunderson^1^, Daniel Shattuck^1^, C. Ann Vitous^1^, Amy Green^2^, Mary Ramos^3^, Cathleen Willging^1^

##### ^1^Pacific Institute for Research and Evaluation, Albuquerque, NM, USA; ^2^The Trevor Project, Los Angeles, CA, USA; ^3^Department of Pediatrics, University of New Mexico, Albuquerque, NM, USA

###### **Correspondence:** Lara Gunderson (lgunderson@pire.org)

**Background:** Implementing evidence-based interventions (EBIs) is imperative to increase school safety for sexual and gender minority (SGM) students and their peers. Recently, the Expert Recommendations for Implementing Change (ERIC), a list of 73 discrete implementation strategies used in health care settings, was adapted for describing school-based interventions. The 2019 School Implementation Strategies, Translating the ERIC Resources (SISTER) compilation resulted in 75 discrete implementation strategies relevant to schools. This paper examines which of the SISTER strategies were used in a cluster randomized control trial focused on the uptake of six Centers for Disease Control and Prevention-recommended EBIs to reduce suicidality and risk behaviors among SGM adolescents and their peers. The study applies the Dynamic Adaptation Process (DAP), a phased, data-driven implementation planning process, that accounts for adaptation while ensuring fidelity to EBI core elements.

**Methods:** Qualitative data for this paper derive from transcripts among 38 school implementation leads and administrators, and 17 focus groups with school-level implementation resource teams (IRTs). These data were collected at intervention schools during the third year of a five-year study. We undertook iterative comparative analysis of qualitative data, mapping the codes to the relevant domains in the SISTER compilation. Authors synthesized findings by creating a descriptive matrix of the SISTER implementation strategies that IRTs applied in their schools.

**Findings:** Within the SISTER, we found that intervention schools implemented 21 strategies as encouraged under the study and 19 as part of their own efforts; 7 strategies were expanded independently by IRTs. We delineate which strategies the study structure supported and those that individual schools enacted on their own within the DAP.

**Implications for D&I Research:** This study offers insight into how and why schools select between and create new strategies. The DAP fosters freedom to expand beyond the study-supported strategies. Qualitative data illuminate motives for strategy diversification related to site-specific advancement and improving fit of EBIs with local contexts. Qualitative methods allow an in-depth illustration of strategies teams creatively enact in their efforts to advance EBIs. We discuss the use of the DAP to support the local implementation teams charged with advancing strategies to make schools safer for SGM students.

**Primary Funding Source**

National Institutes of Health

### S166 Coaching as an implementation strategy for successful school-based interventions to reduce health disparities among sexual and gender minority youth

#### Daniel Shattuck^1^, Sonnie Williams^1^, Janie Lee Hall^1^, Mary Ramos^2^, Cathleen Willging^1^

##### ^1^Pacific Institute for Research and Evaluation, Albuquerque, NM, USA; ^2^Department of Pediatrics, University of New Mexico, Albuquerque, NM, USA

###### **Correspondence:** Daniel Shattuck (dshattuck@pire.org)

**Background:** Research points to six evidence-based practices (EBPs) that make schools safer for sexual and gender minority (SGM) youth, reducing their elevated risk for adverse health and mental health outcomes. However, fewer than 12.2% of U.S. schools implement all six. To support the uptake of each EBP in high schools in New Mexico, our study applies coach-based technical assistance in the Dynamic Adaptation Process (DAP), an implementation framework that anticipates flexibility in responding to ongoing data-informed feedback. We critically examine the role of coaching as a key DAP component and the position of coaches in our wider implementation support structure.

**Methods:** Researchers conducted 88 semi-structured interviews and 33 focus groups spanning two cycles of annual data collection with school administrators, school nurses, teachers, implementation coaches, and other stakeholders across 19 implementation sites. Additional data were drawn from coaching debriefs and logs, and fidelity measurements. These qualitative data were analyzed qualitatively using iterative coding and thematic analysis techniques.

**Findings:** First, effective coaching within the DAP is facilitative and highly adaptable with coaches navigating inner contexts, or the internal workings, of schools, and outer contexts representing their broader socio-political and economic environments. Factors influencing implementation that coaches typically address include staff turnover, disengaged administrators, anti-SGM sentiment, public polices, and health service availability. Second, coaches serve as a two-way bridge in an implementation support structure enabling mutually-informing relationships among school-level delivery systems, external intermediaries, and researchers. Third, their positions in this structure frees coaches from constraints related to peer-based coaching or authoritative coaching characterizing supervisory and evaluative coaching models.

**Implications for D&I Research:** Schools are complex institutions that vary dramatically and are shaped by inner- and outer-context factors that affect their capacity to successfully implement EBPs. Per the DAP, coaching is vital to negotiating such factors and building this capacity by facilitating two-way communication. While other coaching models center largely on implementation and fidelity monitoring, the DAP fosters site-specific knowledge relevant to research translation and higher-order support systems. Such knowledge is indispensable to tailoring implementations for robust fits and sidestepping research-to-practice gaps that undermine efforts to enhance school safety and SGM student outcomes.

**Primary Funding Source**

National Institutes of Health

### S167 Improving implementation of primary care practice guidelines for sexual and gender minority patients: A mixed-methods study

#### Cathleen Willging^1^, Marisa Sklar^2^, Sonnie Williams^1^, Cameron Crandall^3^

##### ^1^Pacific Institute for Research and Evaluation, Albuquerque, NM, USA; ^2^University of California, San Diego, La Jolla, CA, USA; ^3^University of New Mexico Health Sciences Center, Albuquerque, NM, USA

###### **Correspondence:** Cathleen Willging (cwillging@pire.org)

**Background:** Compared to heterosexual, cisgender populations, sexual and gender minority (SGM) people are more likely to suffer from serious health conditions and insufficient access to health services. Primary care is at the frontlines of healthcare provision; yet, few clinics have mechanisms to meet SGM patient needs, contributing to patient dissatisfaction and subpar services. Use of SGM-inclusive practice guidelines at multiple levels of primary care delivery may decrease these disparities.

**Methods:** Guided by the Implementation of Change Model, we undertook surveys and qualitative interviews/focus groups with 32 providers/staff in four Federally-Qualified Health Centers (FQHCs) to examine knowledge, attitudes, clinical preparedness, evidence-based practice (EBP) implementation climate, and organizational readiness for SGM care. Descriptive analyses and between-subscale comparisons controlling for within-rater agreement were conducted to identify areas of greatest need. Interview/focus groups were iteratively coded and analyzed thematically.

**Findings:** Providers/staff preliminarily reported significantly more positive attitudes (*M*=5.56) regarding development of SGM clinical skills than clinical preparedness (*M*=4.04) and knowledge (*M*=3.95) combined (F(1,22)=23.06, *p*<.001); significantly greater focus (*M*=3.43) and selection for openness (*M*=3.22) to EBP implementation climate than educational support (*M*=2.47), recognition (*M*=2.40), and selection for EBP (*M*=2.19) combined (F(1,22)=27.00, *p*<.001); and significantly more positive senior leadership culture (*M*=3.52), staff culture (*M*=3.26), and opinion leaders (*M*=3.32) for organizational readiness to change than leadership behavior (*M*=2.98), leadership feedback (*M*=2.94), and general resources (*M*=2.23) combined (F(1,23)=17.65, *p*<.001). Qualitative findings corroborate these results. Participants lacked formal education in SGM care and familiarity with SGM-inclusive guidelines, while expressing receptivity to training. Reported impediments to improving SGM care included funding and bureaucratic restrictions, provider/staff reticence and technological constraints to collecting SGM data, and inadequate SGM patient outreach in socially-conservative catchment areas. Participants characterized senior leadership as favorably disposed to strengthening SGM care. Results will be finalized in August 2019.

**Implications for D&I Research:** Findings point to opportunities to bolster FQHCs to implement SGM-inclusive guidelines, including advancement of training, coaching, and community-engagement strategies that can be feasibly implemented in under-resourced primary care settings, and cultivation of leadership alignment across service delivery levels. These data also inform adaptation of a broader array of implementation strategies tailored to primary care to enhance guideline uptake and services for SGM patients.

**Primary Funding Source**

National Institutes of Health

### S168 Applying cfir to the implementation of a center of excellence in transgender health

#### Pamela Tinc^1^, Christopher Wolf-Gould^2^, Anne Gadomski^1^

##### ^1^Bassett Healthcare Network, Research Institute, Cooperstown, NY, USA; ^2^Bassett Healthcare Network, Oneonta, NY, USA

###### **Correspondence:** Pamela Tinc (pam.tinc@bassett.org)

**Background:** This study examines the implementation of a Center of Excellence in Transgender Health in a highly complex setting using the Consolidated Framework for Implementation Research (CFIR). The Center of Excellence is currently being implemented within the Gender Wellness Center (GWC), which is part of a large healthcare network in upstate New York. The purpose of the Center of Excellence is to provide a higher level of specialized care to transgender people residing in rural areas. Implementation of the Center of Excellence consists of several components, including: conducting a GAP analysis and needs assessment, developing strategic plans and logic models, expanding educational and outreach opportunities for staff and community organizations, upgrading medical records, and developing a patient registry.

**Methods:** An initial survey was distributed to GWC staff (n=6) in April 2018 at the implementation midpoint. This survey consisted of 74 open-ended questions that discussed 28 CFIR constructs. Survey data was analyzed qualitatively and results were shared with GWC staff for the purpose of quality improvement. The survey instrument will be refined using results of the baseline and distributed as a follow-up in October 2019 before grant funding ends in November 2018. Results of the follow-up surveys will again be analyzed using qualitative methods. This presentation will focus on how CFIR constructs changed from midterm assessment to the end of the grant period.

**Findings:** Results of the first survey indicated that Center staff viewed the implementation positively, but did face challenges. In particular, these challenges focused on knowledge and beliefs about the intervention, individual identification with the organization, complexity of the intervention, external engaging leadership, and many inner and outer setting constructs (and the interactions between them). Once analyzed, results of the follow-up surveys will be used to enhance these findings and make time-based comparisons.

**Implications for D&I Research:** The study describes an implementation evaluation in a highly complex setting. At the local level, results can be used directly to make improvements to the implementation, Center of Excellence, and healthcare network. As the study was evaluated using the CFIR, its results can also be compared with similar studies in other contexts.

**Primary Funding Source**

The Robert Wood Johnson Foundation

### S169 A systems-based approach for childhood obesity treatment across clinical and community contexts

#### Paul Estabrooks (Paul.estabrooks@unmc.edu)

##### University of Nebraska Medical Center, Omaha, NE, USA

**Background:** There has been limited success in the adoption, implementation, and sustainment of evidence-based childhood obesity treatment interventions (EBCOTIs) outside of well-resourced medical centers. Further, interprofessional resources recommended for EBCOTI implementation are scarce across micropolitan and rural communities where infrastructure for EBCOTI implementation may be spread across a broad geographic area and community/clinical organizations.

**Methods:** We applied a systems-based approach that acknowledges that the horizontal and vertical system components across, and within, local organizations that identify and interact with obese children may contribute to addressing the reach, adoption, implementation, and sustainability of an EBCOTI in under-resources regions. Horizontal components of the systems-based approach included engaging several sectors within a local region with the potential to address childhood obesity as well as an integration of research and practice partnerships. Vertical components of the approach were multi-leveled and were operationalized to include, at a minimum, representatives of the staff that interact with the potential beneficiaries of a childhood obesity program and the organizational decision-makers with the authority to approve EBCOTI adoption, implementation, and sustainability

**Findings:** Results across a series of case studies resulted in 4 propositions—that that (1) the integration of scientific and community/clinical systems to address questions that are scientifically innovative *and* have practical implications for stakeholders will result in an increased likelihood of sustained local implementation and evidence of ongoing reach and effectiveness of the evidence-based intervention, (2) the development of sustainable program, practice, or policy approaches is best achieved using a vertical and horizontal systems approach, (3) research synthesis that focuses on evidence-based principles (i.e., core mechanisms of change) rather than products (i.e., evidence-based programs) has a higher likelihood of achieving wide adoption and high-quality implementation, and (4) there is a higher likelihood of scale-up and sustainability when organizational or system governance, values, resources, strategies and structure are leveraged to design for dissemination.

**Implications for D&I Research:** A systems-based approach moves beyond considering dissemination and implementation outcomes in isolation and provides a blueprint for strategic partnerships, mission alignment, and ongoing focus on system capabilities to adapt, adopt, implement, and sustain EBCOTIs.

**Primary Funding Source**

National Institutes of Health

### S170 Building healthy families: A community-based program aimed at decreasing childhood obesity in rural communities

#### Kate Heelan (heelanka@unk.edu)

##### University of Nebraska at Kearney, Kearney, NE, USA

**Background:** The Kearney Micropolitan Statistical Area is anchored by the city of Kearney (population ~31,000). Approximately 33% of households in this region have children and local data from area pediatricians indicate that the prevalence of childhood obesity among 6-12 year olds is 19% and 25% among low income children in the region. As with most rural communities, the resources for interdisciplinary family-based childhood obesity programs are limited. For example, there are only two registered dietitians in the area and it is a state-designated shortage area for general pediatric services, along with counties to the north, south and west of the city of Kearney. **Methods:** A horizontal systems-based approach led to the development and sustained implementation of Building Healthy Families, a family-based childhood obesity treatment program. This presentation will highlight the approach used to engage an interdisciplinary team across organizations to address childhood obesity in the Kearney Micropolitan region. The goal of the 12-week program was to replicate the magnitude of BMI *z*-score reduction found in efficacy trials.

**Findings:** The team was comprised of a university professor, local pediatrician, dietitian and behavioral psychologist and they began by adapting Epstein and colleague’s efficacious Traffic Light intervention for implementation. Over an 8- year period, ninety-one families initiated the program and 67 (74% retention) families successfully completed the 12-week program (83% attendance). Child participants decreased BMI *z*-score (-0.27±0.22) at a level consistent with previous efficacy trials. Children also had significant decreases in fat mass (-2.89±3.90 kg) and increases in fat free mass (0.70±1.36 kg).

**Implications for D&I Research:** Our effectiveness data demonstrates adaptations for rural implementation were appropriate. Our presentation will discuss challenges experienced while enhancing reach, recruitment efforts using schools and medical clinics, and sustainability within rural populations. Identifying and creating collaborative system-based approaches to address these issues has potential for generalizability in rural communities.

**Primary Funding Source**

Nebraska Rural Futures Institute

### S171 Applying a CBPR approach to address capacity and sustainability of childhood obesity treatment interventions across and within different community organizations in a rural region

#### Jamie Zoellner (jz9q@virginia.edu)

##### University of Virginia, Christiansburg, VA, USA

**Background:** To address the research-practice gapof translating evidence-based childhood obesity treatment interventions (EBCOTI) into rural regions, this research is guided by a systems-based and community-based participatory research (CBPR) approach.

**Methods:** This research focused on the rural Dan River Region, a medically underserved area with high rates of childhood obesity. A CBPR process led to an NIH-funded planning grant (2013-2016) and subsequent PCORI-funded type 1 hybrid effectiveness-implementation trial (2017-2020). Both projects addressed building community capacity and evaluating the feasibility of adapting, implementing, and sustaining an EBCOTI using a horizontal and vertical systems-based approach. Acommunity advisory board (CAB) was formed with representation from local pediatrician groups, state department of health, and community partners (n=18) that could contribute to EBCOTI reach, adoption, implementation, and sustainability. Each organization also included participation from an organizational administrator and service provider (i.e., vertical representation). A Parent Advisory Team (PAT, n=6) was formed following the planning grant to assist with recruitment and engagement. The evaluation was guided by RE-AIM (reach, effectiveness, adoption, implementation, maintenance) and annual mixed-methods capacity surveys/interviews with CAB/PAT members.

**Findings:** Six cohorts of families (n=240 children between 5 & 12 years) were enrolled over 6 years. Key findings from this established CBPR process include: (1) Need for substantial adaptation of evidence-based approach for culture, economic status, and geographic region, (2) Success with training and implementation quality (e.g., community implementation fidelity averages 85-96%), (3) Challenges with maintenance of improved BMI z-scores, (4) Challenges with reach, including decreasing proportional reach with each additional cohort (e.g., Cohort 1=28%, Cohort 6=6%), (5) Continued difficulties with family engagement, despite adding a PAT safety net, and (6) Opportunities and challenges to engage CAB and PAT members in all phases of research and sustain community-academic partnerships necessary for this type of systems-based approach.

**Implications for D&I Research:** CBPR can be used to initiate and sustain a systems-based approach, including optimizing collaborations within clinical and community settings that can improve the translation of EBCOTI interventions into typical practice. However, additional research is needed to enhance reach and effectiveness and to continue EBCOTI sustainability planning for rural, medically-underserved regions.

**Primary Funding Source**

Patient-Centered Outcomes Research Institute

### S172 Facilitating systems-based approaches in multiple rural communities to translate evidence-based childhood obesity interventions into practice

#### Jennie Hill (jennie.hill@unmc.edu)

##### University of Nebraska Medical Center, Omaha, NE, USA

**Background:** A systems-based approach to translate evidence-based childhood obesity interventions (EBCOTIs) appears to be a promising implementation strategy. However, to achieve a public health impact across a large rural region, multiple communities will need be engaged in a systems-based approach. This presentation will describe a CDC funded project to test the utility of packaged EBCOTI with and without a systems-based learning collaborative implementation strategy across 8 communities in Nebraska. We will also present operational definitions across the potential mechanisms of the utility of the systems-based approach using Promoting Action on Research in Health Services (PARIHS).

**Methods:** The study is a mixed-methods, hybrid effectiveness-implementation type 3 research design. We will test an implementation strategy focused on determining local demand and capacity to implement EBCOTIs and test a learning collaborative implementation strategy on its utility to support program adoption, implementation, and sustainability when compared to receiving access to the packaged program and training materials alone. We will also explore potential relationships between PARIHS constructs and RE-AIM outcomes related to reach, adoption, implementation, and sustainability.

**Findings:** We hypothesize that rural communities that adopt the EBCOTI will be characterized by the presence of an organization that (1) can support recruitment of families for participation (e.g., clinic, school), (2) can support EBCOTI implementation (e.g., cooperative extension; YMCA), and (3) have administrators and service providers that align in their perceptions of the trialability, observability of outcomes, and local compatibility of the EBCOTI. We also hypothesize that implementation and sustainability will be characterized by (1) administrators and service providers that share positive perceptions of EBCOTI relative advantage and complexity, (2) contextual factors related to adaptive leadership and structural resources for EBCOTI implementation, and (3) facilitation through learning collaborative strategies that include knowledge provision, system-reflection on progress and outcomes, implementation goal setting, and regular feedback.

**Implications for D&I Research:** This study uses theoretical approaches to test implementation strategies essential to understanding the scale-up of effective interventions among at risk rural populations. We will highlight methods to operationalize and complete preliminary tests on mechanisms of reach, adoption, implementation, and sustainability.

**Primary Funding Source**

Centers for Disease Control and Prevention

### S173 Implementation strategies to advance an organization-wide equity initiative: Are some strategies more effective than others?

#### Sivan Spitzer-Shohat^1,2^, James Williams^1^, Brenda Battle^1^, Jelena Todic^1,3^, Scott Cook^1^, Marshall Chin^1^

##### ^1^University of Chicago, Chicago, IL, USA; ^2^Bar-Ilan University, Safed, Israel; ^3^The University of Texas at San Antonio, San Antonio, TX, USA

###### **Correspondence:** Sivan Spitzer-Shohat (spitzershohat@medicine.bsd.uchicago.edu)

**Background:** In 2013, UChicago Medicine launched its organization-wide Diversity, Inclusion and Equity Initiative, employing various strategies to promote change and transform the organization. We aimed to map the different implementation strategies employed and understand how and to what extent they assisted in advancing the organization’s equity goal.

**Methods:** We employed a convergent mixed-methods retrospective design. Semi-structured interviews conducted with key informants from eight departments (n=42) were recorded and transcribed. We elicited implementation strategies used and categorized them according to the 9 domains identified by Expert Recommendations for Implementing Change (ERIC). Data on social network analysis and perceived innovation fit, including knowledge, skills and obstacles were collected in a survey administered to equity committee members (n=40) and mid-management (n=105).

**Findings:** The organization employed 10 different strategies across 6 different ERIC domains, not including financial, consumer engagement and changes to infrastructure. Three central domains were identified: Developing stakeholder interrelationships by creating a governance structure; Stakeholder Training through in-house developed cultural competence training modalities in which 8,545 hours of training were delivered; and Interactive Assistance provided by the Diversity, Inclusion and Equity department team across the organization. We found that training increased stakeholders’ awareness and willingness to act: “*you are just aware of the different cultures, different ways of thinking about things so that you can accommodate as needed“,*and they perceived implementation of equity as important for the organization (x=4.28 (1 [low] – 5 [high] scale), sd=0.63). Creating a governance structure was not as successful of a strategy for promoting interrelations, as the equity committee’s average network density was low (x=1.227, sd=1.5). Albeit training and assistance provided, employees felt they still faced many implementation obstacles (x=2.68 (1 [low] – 5 [high] scale), sd=0.83) and lacked the knowledge to translate equity into practice: “…there’s a lot of theoretical knowledge we’ve learned but no so much what do we do with it.”

**Implications for D&I Research:** The organization was successful in advancing awareness and creating a willingness to act but, strategies implemented were less successful in bridging silos and implementing equity into everyday practices. Equity Implementation requires specific strategies focused on translating equity across organizational departments and levels into specific tasks.

### S174 Meeting health information technology needs in the management of social determinants of health

#### Joshua Colasurdo^1^, Christie Pizzimenti^1^, David Dorr^1^, Tamar Wyte-Lake^1^, Richard Holden^2^, Rachel Gold^3^, Richelle Koopman^4^, Deborah Cohen^1^

##### ^1^Oregon Health & Science University, Portland, OR, USA; ^2^Indiana University School of Medicine, Indianapolis, IN, USA; ^3^Science Program, Kaiser Permanente Center for Health Research, Portland, OR, USA; ^4^University of Missouri, Columbia, MO, USA

###### **Correspondence:** Christie Pizzimenti (pizzimen@ohsu.edu)

**Background:** Federal initiatives recommend primary care practices systematically collect information about the unmet social needs of their patients in an effort to illuminate social determinants of health (SDH). Most primary care practices use an electronic health record (EHR) for documentation of this patient information. Modifying EHRs to support collection of SDH information could support the widespread implementation and dissemination of SDH screening recommendations.

**Methods:** We collected qualitative data (observation, interviews) in 9 Community Health Centers (CHCs) located in Oregon and Washington. Data were analyzed using an immersion-crystallization process to identify the unmet information needs related to collecting and acting on SDH data. We then engaged with informatics experts to identify functional requirements that would address these needs. We used this information to develop an SDH prototype and recruited stakeholders to perform user acceptance testing and utilized the System Usability Scale (SUS) and semi-structured interviews to gather feedback on and refine the prototype.

**Findings:** The SDH dashboard prototype we developed addressed the identified functional requirements from four categories of unmet information needs: Communication, Consistency, Patient Prioritization, and Referrals. The dashboard prototype summarized information about SDH over time, flagged abnormal results and patient readiness to address them, and incorporated external referral information. It also allowed expansion into more detail upon request and the ability to assign priorities to different SDH domains based on clinical judgement. User acceptance testing results revealed a mean SUS score of 75.1, indicating the tool has higher perceived usability than 73% of current certified EHRs based on global benchmarks. Users reported higher satisfaction with the functionality of the prototype than with their current EHR platform and felt this tool would effectively support the management of social needs in their patients.

**Implications for D&I Research:** EHRs do not currently meet the information needs required by primary care clinical teams to implement and expand SDH documentation and management workflows. The SDH dashboard we developed is EHR-agnostic, addresses unmet information needs, and could – if adopted - support widespread implementation of SDH screening recommendations.

**Primary Funding Source**

Agency for Healthcare Research and Quality

### S175 Using intervention mapping to plan, develop and test implementation feasibility of an integrated health and social services intervention in two diverse communities

#### Andrew Knighton^1^, Kimberly Brunisholz^2^, Lisa Nichols^1^, Russ Elbel^3^, Gene Smith^1^, Angela Choberka^1^, Amber Rich^1^, Thomas Belnap^2^, Todd Allen^1^, Mikelle Moore^1^, Raj Srivastava^4^

##### ^1^Intermountain Healthcare, Salt Lake City, UT, USA; ^2^Intermountain Healthcare, Murray, UT, USA; ^3^SelectHealth, Murray, UT, USA; ^4^University of Utah School of Medicine, Salt Lake City, UT, USA

###### **Correspondence:** Andrew Knighton (andrewknighton@live.com)

**Background:** Poor population health is not randomly distributed but follows a social gradient with adversity clustering in specific communities. Recent federal policy efforts are testing interventions to address health inequities yet, little is known about how to effectively implement these complex, highly-contextual interventions. Our objective was to study implementation feasibility of the Alliance for Determinants of Health (Alliance) program using an innovative implementation science framework. The Alliance is a 3-year demonstration project led by Intermountain Healthcare (Intermountain) designed to improve population health outcomes through integrating health and social services.

**Methods:** Intervention Mapping (IM) using mixed methods analyses was used to plan, develop and test initial implementation feasibility. The steps taken included: a needs assessment using local health needs and actuarial claims data, ethnographic field results and key informant interviews; definition of change and performance objectives; theory and methods selection; program plan and production; implementation planning; and evaluation planning.

**Findings:** The needs assessment identified meaningful variation in local needs and available infrastructure across zip-code-based areas in Utah that guided selection of two communities. Program change objectives were defined, including improved local health and social services integration across sectors involving more than 20 service partners. Given the need for strong local program engagement, a collective impact approach grounded in complex adaptive systems theory was selected to guide development. An adapted Accountable Health Communities model was developed including local community-led steering committees to provide strategic oversight. Intermountain provided $6 million in funding to each community distributed by local community-led finance committees to address local social service gaps. Weekly program huddles with Alliance partners are used to report results and to continuously improve and resolve local barriers. During the first 6 months, 444 eligible Medicaid members were identified; 125 members underwent screening and 66 were navigated to various service partners demonstrating local integration feasibility.

**Implications for D&I Research:** Local communities vary considerably in social service needs, capacity and existing healthcare infrastructure. Implementing programs that integrate health and social services is complex and multi-faceted and should be designed with the local implementation in mind. Rigorous implementation science methods may reduce the risk of implementation failure and strengthen the potential generalizability of results.

**Primary Funding Source**

Intramural research funds of Intermountain Healthcare

### S176 Implementation potential of a text message-delivered medication adherence intervention in federally qualified health centers

#### Lyndsay Nelson^1^, Erin Bergner^1^, Sarah Williamson^1^, Sunil Kripalani^1^, Pamela Hull^1^, Jesus Gonzalez^2^, Lindsay Mayberry^1^

##### ^1^Vanderbilt University Medical Center, Nashville, TN, USA; ^2^University of Illinois College of Medicine, Chicago, IL, USA

###### **Correspondence:** Lyndsay Nelson (lyndsay.a.nelson@vanderbilt.edu)

**Background:** Text message-delivered interventions provide an opportune approach for improving chronic disease self-management and reducing health disparities. Texts can deliver tailored content to patients in their daily lives, do not require Internet access, and are used equally across racial/ethnic and socioeconomic groups. Despite evidence of efficacy, limited research has explored implementation of such interventions in clinical care. As part of a 15-month trial, we partnered with 13 Federally Qualified Health Center (FQHC) sites to evaluate an automated text messaging diabetes support program using a hybrid type 1 effectiveness-implementation design. Following the trial, we collected feedback from patients and clinic staff on implementation potential.

**Methods:** We recruited trial participants and staff (e.g., physicians, nurse practitioners, pharmacists, CMOs, and CEOs) from partnering clinics for a post-study interview. We used the Consolidated Framework for Implementation Research to develop separate interview guides for patients and clinic staff; questions assessed stakeholder readiness and barriers and facilitators to implementation. All interviews were recorded and transcribed verbatim. We coded transcripts using thematic analyses to identify, organize, and interpret themes in the data.

**Findings:** Patient participants (n=36) were 56% female, 58% Black, and 86% had annual incomes <$35K. Patients found the texts helpful and expressed regret the text messages had ended after the trial. They identified potential barriers to patients signing up in an implementation effort to include limited cell phone experience, diabetes stigma, and privacy concerns; they identified a major facilitator to be providers presenting/recommending the program to patients. All clinic staff (n=12) (50% providers, 50% administrators) expressed strong interest in implementing the program based on its capability to meet patients’ needs (e.g., individualization and ease of communication) and alignment with organizational goals. Factors affecting implementation included having staff to provide oversight of the program, integration into workflow, and available funding.

**Implications for D&I Research:** FQHC patients and clinic staff shared positive perceptions of implementing a text messaging support program. Next steps include developing and testing tailored implementation strategies at FQHCs. Findings indicate successful implementation will depend largely on identifying and preparing a clinic champion. We anticipate automated text messaging programs supporting chronic disease self-care may be readily implemented in under-resourced settings.

**Primary Funding Source**

National Institutes of Health

### S177 A prospective comparative case study Examining sustainability outcomes and determinants among lay health advisor programs in low-resource settings

#### Rachel Shelton^1^, Laura Brotzman^1^, Detric Johnson^2^, Alexis Smith^1^, Deborah Erwin^2^

##### ^1^Columbia University Mailman School of Public Health, New York, NY, USA; ^2^Roswell Park Comprehensive Cancer Center, Buffalo, NY, USA

###### **Correspondence:** Rachel Shelton (rs3108@cumc.columbia.edu)

**Background:** Sustainability is a critically important but understudied area within implementation science. While there is conceptual agreement among researchers that sustainability is the continued use of program components for the sustained achievement of health behaviors and outcomes, there is also growing recognition that sustainability is dynamic and programs may require adaptations in response to changing evidence, contexts, and needs. Empirical work on sustainability, particularly in low-resource settings, is needed to inform and advance research and existing conceptual frameworks in this area. The National Witness Project (NWP), an evidence-based Lay Health Advisor (LHA) program, is an ideal platform for examining sustainability and adaptation. NWP is a Research Tested Intervention Program from National Cancer Institute for cancer screening that has been nationally disseminated and implemented for 25 years among African American women in under-resourced settings.

**Methods:** We used a prospective mixed-methods (in-depth interviews and surveys) comparative case study design over three years among ten sites to: 1) examine patterns in program sustainability over time across and within sites; and 2) identify multi-level determinants from the LHA Sustainability Framework that are similar and different across high, moderate, and low sustained NWP sites.

**Findings:** We identified potential determinants at multiple levels across the LHA Sustainability Framework, but found that contextual factors (e.g. relationships with funders; partnerships with national leadership/academic centers) and organizational factors (e.g. diversity of funding, organizational stability and space; leadership/staff turnover, innovation of leadership, incentives for LHAs, non-profit status) were particularly important in understanding patterns of sustainability over time. Further, we found that sustainability within sites was dynamic and not static in terms of continued program delivery and gaps in programming.

**Implications for D&I Research:** Study findings: 1) provide empirical insight into the dynamic nature of sustainability over time and reinforce the importance of studying sustainability longitudinally across multiple time points; and 2) suggest organizational factors may be particularly important in understanding patterns of sustainability in community-based settings. Findings can be used to inform and plan for the sustainability of disparity and health equity-focused programs in community settings, and advance work that identifies and operationalizes key aspects of context that are meaningful for studying sustainability in low-resource settings.

**Primary Funding Source**

American Cancer Society

## Poster Slam

### S-178 Integrating implementation into a conceptual framework for academic detailing’s impact on providers’ prescribing behavior

#### Mark Bounthavong^1,2^, Mark McGovern^3^, Melissa Christopher^4^, Amanda Midboe^5,6^

##### ^1^Pharmacy Benefits Management Academic Detailing Service, Veterans Health Administration, Palo Alto, CA, USA; ^2^University of Washington, Seattle, CA, USA; ^3^Stanford University School of Medicine, Stanford, CA, USA; ^4^Veterans Health Administration, Washington, DC, USA; ^5^Stanford University, Stanford, CA, USA; ^6^Center for Innovation to Implementation, Veterans Health Administration, Menlo Park, CA, USA

###### **Correspondence:** Mark Bounthavong (mbounth@uw.edu)

**Background:** Academic detailing is an evidence-based, educational outreach intervention designed to address provider prescribing practices with evidence and is in use in a variety of health care systems across the US. It relies on population-level data, audit and feedback techniques, and practice facilitation to influence a provider's prescribing behavior. Theoretical frameworks on providers’ prescribing behavior exist but do not consider academic detailing’s implementation process and its link to modifying provider prescribing behavior. The objective was to develop a conceptual framework that would incorporate implementation process into the mechanism of academic detailing on influencing provider prescribing behavior.

**Methods:** A literature review in addition to consultation with academic detailing experts was performed to identify existing theoretical frameworks on provider’s prescribing behavior. Implementation frameworks were also identified and reviewed for compatibility with the current frameworks on prescribing behavior.

**Findings:** The Social Marketing Framework (SMF) and the Theory of Planned Behavior (TPB) were identified as key to explaining provider prescribing behavior. More importantly, we identified an ecological framework for effective implementation that incorporated the Framework for Effective Implementation with Interactive Systems Framework for Dissemination and Implementation. The ecological framework for implementation was incorporated with the SMF and TPB to provide a comprehensive conceptual framework that integrated implementation into the mechanisms for explaining academic detailing’s impact on provider prescribing behavior. Altogether, effective implementation is influenced by five categories: innovations, providers, communities, the prevention delivery system, and the prevention support system. The quality of implementation directly can directly affect the outcomes from academic detailing, which is measured by the changing prescribing patterns of providers. If implementation is not executed appropriately, the factors that decision makers use to judge the effectiveness of academic detailing will be invariable biased. Integrating implementation into this conceptual framework addresses the gap left by current frameworks on academic detailing’s impact on provider prescribing behavior.

**Implications for D&I Research:** By integrating implementation as part of the conceptual framework to understanding academic detailing’s impact on provider prescribing behavior, we address an important feature of academic detailing that can impact its effectiveness on influencing provider prescribing behavior. Future analysis will need to empirically test this conceptual framework.

**Primary Funding Source**

Department of Veterans Affairs

### S-179 Adaptation of evidence-based interventions and implementation strategies: An intervention mapping approach for optimizing impact

#### Maria Fernandez^1^, Cam Escoffery^2^, Maya Foster^1^, Laura Savas^1^, Patricia Dolan Mullen^1^

##### ^1^University of Texas Health Science Center at Houston, Houston, TX, USA; ^2^Rollins School of Public Health, Emory University, Atlanta, GA, USA

###### **Correspondence:** Maria Fernandez (maria.e.fernandez@uth.tmc.edu)

**Background:** Adaptation of evidence-based interventions (EBIs) and implementation strategies is an essential process for implementation and dissemination research and practice. Our team recently summarized 13 adaptation frameworks and identified common steps to guide the adaptation process. While adaptation models exist, they provide only limited guidance on what should change and what should remain the same and how to integrate participatory processes throughout the adaptation process.

**Methods:** Intervention Mapping for adaptation (IM ADAPT) provides step-by-step guidance on how to adapt EBIs and implementation strategies. The process includes the development of a logic model of change (LMC) based on the community/clinical assessment as an initial step to describe the intervention goals for influencing determinants, behavior and environment. The IM Adapt process also guides the review and documentation of the internal logic of the EBI including the targets of the original EBI, determinants addresses, change methods and/ or strategies used and implementation strategies. Planners then compare the EBI content and features to the needs of the new population or setting to determine what needed adaptation and core elements that should remain intact. These may include change methods/techniques used in the original intervention since these often represent the intervention’s core elements.

**Findings:** We describe the steps of IM Adapt and how it can serve to optimize intervention reach and impact. We also provide examples of adaptations using this this process including the adaptation of a breast and cervical cancer screening intervention and an HPV vaccination intervention. We will then describe the development and testing of IM Adapt online, a tool for finding and adapting evidence based interventions for cancer control and how it can be used to improve the use of EBIs including interventions from the National Cancer Institute’s Research Tested Intervention Programs (RTIPs) resource.

**Implications for D&I Research:** Implementation science research and practice requires systematic methods for adapting interventions and implementation strategies. IM Adapt has been used to successfully adapt EBI and implementation strategies. It can advance the field by providing a detailed process to inform adaptation and guide research efforts to improve the process.

### S-180 Advancing the capacity of practice implementers: An educational training model from the occupational therapy profession

#### Lisa Juckett (lisa.juckett@osumc.edu)

##### The Ohio State University, Columbus, OH, USA

**Background:** Clinical doctorate programs in allied health are becoming increasingly prevalent in academic institutions, resulting in the production of healthcare practitioners who have been trained to value and implement evidence-based practices (EBPs). Although such programs often require the completion of “Capstone” projects where students must assess EBP implementation at actual healthcare sites, seldom have allied health students received guidance on implementation measurement. This study examined the final Capstone projects of two cohorts of occupational therapy doctorate (OTD) students to determine the extent to which implementation outcomes were measured before and after students and faculty advisors were trained on Proctor and colleagues’ implementation outcomes taxonomy.

**Methods:** Final Capstone projects from two OTD cohorts were examined by means of descriptive and chi-square analyses to identify and compare differences in students’ measurement of implementation outcomes at their respective Capstone sites. Cohort 1 did not receive outcomes taxonomy training; Cohort 2 was trained on the taxonomy as were faculty advisors. Descriptive analyses were adopted to determine the rate of students that measured implementation outcomes as well as the types of outcomes being assessed. Chi-square analyses indicated differences in outcome measurement between the two student cohorts.

**Findings:** A total of 57 Capstone projects were included in our analysis of the two OTD cohorts. Of the cohort that received taxonomy training, 38% of students measured implementation outcomes as part of their final Capstone, yielding a significant difference in taxonomy use between cohorts, *x*^2^(1, *N* = 57) = 6.46, *p* = .012. Of the eight implementation outcomes, feasibility (*n* = 16), appropriateness (*n* = 12), and acceptability (*n* = 7) were the most frequently measured outcomes which were assessed through semi-structured interviews with Capstone site stakeholders.

**Implications for D&I Research:** Clinical doctorate programs have a responsibility to train future allied health practitioners not only in the importance of EBPs but in the complexities of EBP implementation. Our study presents one educational training model of how clinical doctoral programs can begin to infuse implementation science concepts into their curricula with the goal of producing practice implementers who can draw from these concepts throughout their careers as allied health professionals.

### S-181 Evaluating the implementation of a peer recovery coach model to reach underserved, minority individuals not engaged in substance use treatment in Baltimore City.

#### Mary B. Kleinman^1^, Kelly Doran^2^, Julia M. Felton^3^, Emily N. Satinsky^1^, Dwayne Dean^4^, Valerie Bradley^5^, Jessica F. Magidson^1^

##### ^1^University of Maryland College Park, College Park, MD, USA; ^2^University of Maryland Baltimore, Baltimore, MD, USA; ^3^Michigan State University, Flint, MI, USA; ^4^University Maryland Medical Center, Baltimore, MD, USA; ^5^University of Maryland, College Park, MD, USA

###### **Correspondence:** Mary B. Kleinman (mkleinm@umd.edu)

**Background:** Low-income, racial/ethnic minority individuals face disparities in access to substance use (SU) treatment. Peer recovery coaches, individuals with lived experience of SU, present a unique opportunity to assist those encountering barriers to treatment. Peers, embedded in community rather than clinical settings, can help reach those not engaged in treatment to promote harm reduction and linkage-to-care.

**Methods:** Using RE-AIM as a guide, we piloted a peer-delivered linkage-to-care intervention in a community resource center serving homeless and low-income residents of Baltimore, which has one of the highest opioid overdose fatality rates in the US. We examined the reach, effectiveness, adoption and implementation of this model.

**Findings:** Of 101 clients approached by or referred to the peer, 37 were interested in addressing their SU. Of those, the peer linked 67.5% (*n*=25) to treatment. Barriers to care addressed included risk factors for relapse, housing, and case management support. Forty-eight percent of clients linked to SU treatment remained in treatment at 30 days post-linkage. Table 1 further details results by component of RE-AIM.

**Implications for D&I Research:** These findings demonstrate that a peer integrated into a community setting can successfully link people to treatment for SU and support recovery through regular follow-up and addressing barriers to care. This process required adaptation based on individual goals and fluctuation in readiness for treatment. The RE-AIM framework provided an effective model to evaluate this work and should be considered for further research in the context of SU among underserved populations, especially focused on retention in treatment.

**Primary Funding Source**

UMB-UMCP Research Innovation Seed Grant

**Table 1 (abstract S-181).** RE-AIM framework to evaluate a peer-delivered linkage to SU treatment intervention

	O*	I*	C*	Outcome	Result
**Reach**			X	Proportion clients interested in working with the peer.	37/101 (36.6%)
**Effectiveness**			X	Linkage and retention outcomes.	Intake: 37 (100%) Initiated treatment: 23 (62.2%) 14-day retention: 16 (43.2%) 30-day retention: 12 (32.4%)
**Adoption**	X			Of clients with full intake data, proportion referred to the peer by organization staff.	5/28 (17.8%)
**Implementation**		X	X	Proportion clients working with the peer: -who had complete follow-up documentation. -who were linked to treatment.	Documentation: 16 (57.1%) Linkage: 25 (67.5%)
**Maintenance**	X			Sustained peer role following completion of pilot.	Trial ongoing.

*O=organization; I=interventionist; C=client

### S-182 Improving implementation of tobacco cessation policies in Medicaid managed care plans in California

#### Sara McMenamin^1^, Sara Yoeun^2^

##### ^1^University of California, San Diego, La Jolla, CA, USA; ^2^University of California San Diego, La Jolla, CA, USA

###### **Correspondence:** Sara McMenamin (smcmenamin@ucsd.edu)

**Background:** In November of 2016, the California Department of Health Care Services (DHCS) issued an All Plan Letter (APL 16-014) to the California Medi-Cal Managed Care Programs (MCPs) to provide information and guidance on MCP requirements for comprehensive tobacco cessation services. Researchers at UC, San Diego (UCSD) set out to examine Medi-Cal’s MCPs’ progress in implementing each section of APL 16-014, identify factors associated with higher levels of implementation, and to make recommendations as to where further guidance may be warranted from DHCS to reach full implementation.

**Methods:** From September 2018 through February 2019, UCSD researchers surveyed health educators and relevant personnel within California’s 25 Medi-Cal MCPs to document each MCP’s smoking cessation services and policies in 2018. Data were collected via three methods: (1) a web-based survey, (2) an in-depth phone interview, and (3) researcher collection of MCP smoking cessation relevant-documents. Data were collected for 24 of the 25 Medi-Cal MCPs (i.e., 96% response rate).

**Findings:** Two years after the release of the APL, MCPs demonstrate low levels of full implementation. On average, MCPs fully implemented three of eight sections of the APL. MCPs had highest implementation rates for APL-items related to coverage of cessation medications (55%), requirements for primary care providers to institute tobacco user identification systems (55%), and coverage for children and adolescents (59%). Lowest implementation was reported for items that required MCPs to ensure that contracted providers were referring members to a telephone quitline (18%); to ensure that contracted providers conduct tobacco use assessments (29%); and to ensure that pregnant tobacco users were referred to a telephone quitline (36%). Overall, only one MCP (5%) had fully implemented all eight sections of the APL.

**Implications for D&I Research:** These results indicate that although APL 16-014 was successful in creating more consistent benefits across MCPs, 95% of MCPs have not fully implemented all eight sections of the APL. Future research will examine organizational and environmental factors that may either promote or prevent full adoption of the APL requirements. Further guidance from DHCS may be needed to achieve full implementation of APL 16-014 among all 25 Medi-Cal MCPs.

**Primary Funding Source**

Tobacco-Related Disease Research Program (TRDRP)

### S-183 Implementing a trauma informed care model at New Jersey HIV care and treatment sites

#### Lindsay Senter^1^, Beth Hurley^2^, Sabrina Baumgartner^2^, Mary Dino^2^, Tony Jimenez^2^, Gricel Arredondo^2^, Barbara Cicatelli^2^

##### ^1^Research & Evaluation, CAI (Cicatelli Associates), New York, NY, USA; ^2^CAI (Cicatelli Associates), New York, NY, USA

###### **Correspondence:** Beth Hurley (bhurley@caiglobal.org)

**Background:** With the New Jersey Department of Health, Division of HIV/STD/TB Services, CAI is implementing the NJ Trauma Informed Care (NJTIC) Project among 31 funded agencies statewide over five years. Trauma can impact PLWHs’ ability to seek care, remain in care, adhere to treatment, and achieve viral suppression. This strengths-based service delivery model uses implementation science frameworks and approaches and works with all levels of staff integrating TIC into the agency culture and service delivery to improve HIV care continuum outcomes and client-level experiences.

**Methods:** NJTIC model includes four implementation phases; 1) understanding need and context through agency cultural and physical environment self-assessments on TIC integration capacity, which is completed by all levels of staff; 2) action planning using TA logs and action plan templates; 3) service delivery by first training all staff in TIC education, psychoeducation, skills-based training, and use of the Brief Trauma Screening Tool (BTS); and 4) monitoring progress/sustainability using a Client Encounter Form (CEF) and real-time data review to inform progress (e.g., unique clients served, TIC service provision, viral load).

**Findings:** To date, agencies have made substantive physical and cultural changes (e.g., rearranging furniture in waiting room to create more peaceful registration). Staff who have received foundational Trauma Education report significant changes in self-efficacy to provide TIC services and changes in knowledge and attitudes related to TIC. Since March 2019, agencies have recorded 804 client encounters (754 unique clients). Nearly 93% of clients who were offered TIC services were screened at their first visit using the BTS, and 85% of clients who were screened received TIC General Education. Nearly 25% of clients screened 3 or above on the BTS, indicating the incidence of a traumatic event in the past month. Of those with a positive screen, 89% received TIC General Education and 4% received at least one Psychoeducation session.

**Implications for D&I Research:** This initiative offers promising evaluation tools and real-world capacity building approaches to support staff and providers in their ability to integrate and implement TIC in their clinical and community-based organizational settings.

**Primary Funding Source**

New Jersey Department of Health and Hyacinth AIDS Foundation

### S-184 Implementation science for depression interventions in low-and middle-income countries: A systematic review

#### Bradley Wagenaar^1^, Wilson Hammett^1^, Courtney Jackson^1^, Dana Atkins^1^, Jennifer Belus^2^, Christopher Kemp^1^

##### ^1^University of Washington, Seattle, WA, USA; ^2^University of Maryland, College Mark, MD, USA

###### **Correspondence:** Christopher Kemp (kempc@uw.edu)

**Background:** Significant investments are being made to decrease the gap between growing evidence on the effectiveness of depression interventions in low-and middle-income countries (LMICs) and the application of these interventions at scale. Our objectives were to systematically review implementation research targeting depression interventions in LMICs and critically assess coverage and scientific gaps.

**Methods:** PubMed, CINAHL, PsycINFO, and EMBASE were searched for evaluations of depression interventions in LMICs reporting at least one implementation outcome and published through March 2019.

**Findings:** A total of 8,714 studies were screened, 759 were assessed for eligibility, and 81 studies published between 2003 and 2019 met inclusion criteria. Most studies were conducted in Sub-Saharan Africa (n=30; 37.0%), followed by South Asia (n=24; 29.6%), and Latin America and the Caribbean (n=13; 16.1%). The majority of studies (n=61; 75.3%) reported outcomes for a depression intervention that was implemented at the pilot/research phase. The majority of studies (n=47; 58.0%) focused on depressive interventions delivered at the facility level, with 32 (39.5%) delivered in the community. Primary depression intervention modalities were individual psychotherapy (n=30; 37.0%) and multicomponent interventions (n=27; 33.3%). Only 21 studies (25.9%) tested an implementation strategy, with the most common implementation strategy being revising professional roles (n=10; 47.6%). Common study designs were mixed methods (n=29; 35.8%), quasi-experimental uncontrolled pre-post (n=17; 21.0%), and individual randomized trials (n=16; 19.8). The most common implementation outcomes reported were acceptability (n=52; 64.2%), followed by feasibility (n=29; 35.8%), fidelity (n=18; 22.2%), cost (n=14; 17.3%), and appropriateness (n=14; 17.3%). Only 4 studies (4.9%) reported adoption, 3 (3.7%) reported sustainability, and no study reported penetration.

**Implications for D&I Research:** Implementation research for depression interventions in LMICs has focused largely on early-stage implementation outcomes, with the primary aim of testing interventions under pilot researcher-controlled implementation. Future implementation science should focus on testing implementation strategies for depression interventions being delivered in routine care. This would include increased consideration of contextual factors, as well as later-stage implementation outcomes such as cost, penetration, and sustainability. Certain LMIC regions, such as Middle East and North Africa and Europe and Central Asia could be prioritized for investments given the paucity of existing studies.

**Primary Funding Source**

National Institutes of Health

### S-185 Results from piloting an innovative model to engage multiple key stakeholders in learning about suicide prevention research for strategic action in under-resourced rural indigenous communities

#### Lisa Wexler^1^, Lauren White^1^, Panganga Pungowiyi^2^, Roberta Moto^3^

##### ^1^University of Michigan, Ann Arbor, MI, USA; ^2^Kawerak, Nome, AK, USA; ^3^Maniilaq Association, Kotzebue, AK, USA

###### **Correspondence:** Lauren White (lawhi@umich.edu)

**Background:** Studies focused on understanding how, why, and under what conditions community members and practitioners learn about, interpret and utilize scientific research are relatively rare. Among these, little is known about how members of marginalized groups understand and translate research to practice in ways that align with their epistemologies and fit within the constraints of their daily lives. Importantly, to date, few studies examine the applied learning about research evidence among non-professional end users who live and work within complex, community-based settings. The presentation reports on the results from a 3-year, NIMH pilot of PC CARES (Promoting Community Conversations About Research to End Suicide). Through a series of 9 sessions, PC CARES synthesizes actionable research evidence for community audiences, supports a process of community education and mobilization, and intentionally develops a community of practice across community groups and institutions.

**Methods:** Piloted in 10 rural and remote Alaska Native (AN) communities, the mixed methods study used process measures, which included participation and audio recorded community learning circles led by local facilitators, and analyzed discussions taking place within these community sessions. Outcome indicators track within person-change in perceived knowledge, skills, attitudes toward prevention, and community of practice; and, self-reported behavioral outcomes before and after participating.

**Findings:** Local PC CARES facilitators hosted 59 learning circles with 535 participants (376 unique) between 2015-2017. They followed the protocol with fidelity (80%), and interpreted the scientific research accurately 81% of the time. Discussions showed participants’ understood the research evidence and applied it to their lives and work. Linked participant surveys indicate positive changes in knowledge, beliefs, collaborative relationships and prevention actions.

**Implications for D&I Research:** The presentation will highlight implementation factors: 1) the process of planning, engaging, executing and evaluating PC CARES with Alaska Native partners, 2) intervention characteristics designed to promote uptake and use of the research evidence by a diverse group of stakeholders in learning circles, 3) tracking implementation of locally-run learning circles, and 4) the promising results in terms of feasibility, local facilitators’ fidelity and accuracy, and learning and behavioral outcomes.

**Primary Funding Source**

National Institutes of Health

